# Recipient-focused interventions to increase vaccine uptake in high and upper-middle income countries: a systematic review and network meta-analysis

**DOI:** 10.1016/j.eclinm.2025.103643

**Published:** 2025-11-18

**Authors:** Sarah R. Davies, Annabel L. Davies, Julian P.T. Higgins, Deborah M. Caldwell, Zak A. Thornton, Elisabeth Aiton, Ifra Ali, Sarah Dawson, Carmel McGrath, Thomas Parkhouse, Lucy Yardley, Julie Yates, Louise Letley, Sharif A. Ismail, Clare E. French

**Affiliations:** a Population Health Sciences, Bristol Medical School, University of Bristol, Bristol, UK; b NIHR Health Protection Research Unit in Behavioural Science and Evaluation at University of Bristol, Bristol, UK; c NIHR Applied Research Collaboration West (ARC West) at University Hospitals Bristol and Weston NHS Foundation Trust, Bristol, UK; d MRC Integrative Epidemiology Unit, University of Bristol, Bristol, UK; e NIHR Health Protection Research Unit in Vaccines and Immunisation, Department of Global Health and Development, Faculty of Public Health and Policy, London School of Hygiene and Tropical Medicine, London, UK; f Faculty of Health and Applied Sciences, School of Health and Social Wellbeing, University of West England, Bristol, UK; g School of Psychology, University of Southampton, Southampton, UK; h UK Health Security Agency, London, UK; i Department of Global Health and Development, Faculty of Public Health and Policy, London School of Hygiene and Tropical Medicine, London, UK

**Keywords:** Vaccine uptake, Vaccinations, Network meta-analysis, Systematic review

## Abstract

**Background:**

Vaccination is a highly effective public health tool. However, uptake of many vaccines has declined globally over the past decade, exacerbated by the COVID-19 pandemic, with coverage often falling short of critical thresholds. There is a large evidence-base of vaccine uptake interventions targeting vaccine recipients. Here we aim to synthesise evidence on the comparative effectiveness of these interventions, assess variations by age, and evaluate the impact of the COVID-19 pandemic on intervention effectiveness.

**Methods:**

We conducted a systematic review, searching nine databases and grey literature for randomised controlled trials from high and upper-middle income countries, published from 2000 to April 2024. We included interventions targeted at vaccine recipients of any age (or their caregivers) living in the community, aimed at increasing uptake of routine or selective/targeted vaccinations on the UK schedule and categorised them into six intervention categories (access, affordability, education, reminder, education and reminder, and multi-component). Our outcome was number vaccinated. We estimated odds ratios (ORs) using Bayesian random effects network meta-analysis (NMA) combining all direct and indirect evidence on relative intervention effects in a single coherent analysis. Risk of bias was assessed using the Cochrane RoB 2 tool and certainty in the evidence using CINeMA. This review is registered with PROPSERO (CRD42022369139).

**Findings:**

We included 268 studies (6,243,118 participants; 39% male, 61% female) with 223 eligible for analysis. Studies not eligible for analysis are narratively reported. Of those eligible for analysis, we assessed 105 studies as low risk of bias, 87 as some concerns, and 31 as high risk. Our NMA produced ORs and 95% credible intervals (CrI) > 1 for all six intervention categories, indicating favourable vaccine uptake compared to control (standard practice, no intervention, or attention placebo), with multicomponent (OR: 2.09, 95% CrI: 1.66–2.63), access (OR: 1.74, 95% CrI: 1.35–2.26), and affordability (OR: 1.87, 95% CrI: 1.47–2.40) interventions appearing most effective followed by education and reminder (OR: 1.47, 95% CrI: 1.29–1.67), reminder (OR: 1.36, 95% CrI: 1.22–1.50), and education (OR: 1.33, 95% CrI: 1.19–1.49). We judged the evidence for all six interventions as moderate certainty. Subgroup analyses exploring the effect of the COVID-19 pandemic suggest that affordability interventions may be less effective post-2020 (OR: 1.35, 95% CrI: 0.94–1.94) than pre-2020 (OR: 2.32, 95% CrI:1.72–3.19), similarly multicomponent interventions appear less effective post-2020 (OR: 1.45, 95% CrI: 1.00–2.10) than pre-2020 (OR: 2.50, 95% CrI: 1.89–3.33) whilst access, education, reminder, and education and reminder intervention effects remained stable.

**Interpretation:**

Vaccination programmes in high and upper-middle income countries should prioritise ensuring vaccines are accessible, convenient, and affordable for the recipient. Efforts should be directed towards further investigating and optimising access and affordability interventions. The possible negative impacts of the COVID-19 pandemic on the effectiveness of affordability and multicomponent interventions should be monitored.

**Funding:**

10.13039/501100001921NIHR Public Health Research Programme (NIHR135130).

**Research in context**

**Evidence before this study**

We searched the Cochrane Database of Systematic Reviews (CDSR) on the Cochrane Library, Ovid MEDLINE, NIHR Journals Library, DoPHER, Health Evidence, and Epistemonikos for reviews of interventions to increase vaccine uptake published from the inception of each database to September 2022, prior to registering our review protocol. The vast majority of existing reviews focused on specific populations, vaccine types and/or interventions. A broader set of evidence reviews on vaccine uptake in the general population, published in 2022, excluded seasonal vaccinations such as influenza and COVID-19. Whilst two broader reviews have been published more recently these did not apply rigorous systematic review methods—one used a basic search strategy identifying just 88 trials from 17 countries, the other included a large number of studies on behavioural interventions for vaccine uptake but information on inclusion criteria was unclear, searches were completed on a single database, and no study characteristics were provided. None of the existing reviews assessed the comparative effectiveness of the multiple competing intervention types using all available direct and indirect evidence.

**Added value of this study**

To our knowledge this is the first network meta-analysis of interventions aimed at vaccine recipients to increase vaccine uptake. Our review provides a significant addition to the growing body of evidence on this high priority topic. Our broad scope and very comprehensive searches resulted in a large dataset of 268 trials with 6,243,118 participants. Uniquely, our analyses utilised both direct and indirect evidence to produce estimates of the comparative effectiveness of interventions and non-active controls. Our review is the first to explore the impact of the COVID-19 pandemic on intervention effectiveness, and additionally provides subgroup analyses by age.

**Implications of all the available evidence**

Our analysis shows that interventions to enhance access, improve affordability, and those incorporating multiple components are most effective at increasing vaccine uptake. However, these intervention types contributed the least data. Evidence from trials conducted since the start of the COVID-19 pandemic suggests that the effectiveness of some of these interventions may have been affected. Future research should prioritize these types of interventions, including examining whether this change is long-term or specific to COVID-19 vaccines.

## Introduction

Vaccines are an extremely powerful and cost-effective public health tool for preventing the spread of infectious diseases.^1^ Achieving high vaccine uptake is crucial, however, for many vaccinations critical coverage thresholds are failing to be met.^2^ Global declines have been reported across many programmes, particularly since 2019, linked to major disruption to routine immunisation delivery during the COVID-19 pandemic.^3^ Lower vaccination coverage increases the risk of vaccine preventable disease outbreaks; worryingly measles cases rose by 3000% in Europe and Central Asia in 2023.^4^ Optimising global immunisation by identifying and addressing gaps in immunisation coverage is a strategic priority of the World Health Organisations (WHO's) Immunisation Agenda 2030.^5^

Barriers to vaccine uptake may include difficulty accessing healthcare, lack of awareness around vaccine availability, low confidence in the vaccine, distrust, lack of endorsement, lack of communication and recommendation from trusted providers and community leaders,^6^ and more recently vaccine fatigue related to the COVID-19 pandemic.^7^ Numerous, diverse, interventions aimed at vaccine recipients (or their caregivers) have been explored to address these barriers, including interventions that provide education or information, those that improve access to vaccines, provide reminders, and offer financial incentives or cover costs involved in getting vaccinated. Systematic reviews of intervention effectiveness tend to address specific populations, vaccine types and/or interventions.^8^^,^^9^ A recent set of evidence reviews on vaccine uptake in the general population included all vaccines on the UK immunisation schedule but excluded seasonal vaccinations such as influenza (included in a prior evidence review incorporating evidence to 2016 only) and COVID-19.^10^ Where reviews do include a broader range of the evidence,^11^^,^^12^ these have methodological limitations and syntheses have been limited to pairwise meta-analyses of intervention categories (e.g. education or reminders) compared with controls, which do not provide evidence on the comparative effectiveness of interventions.

Network meta-analysis (NMA) is an extension of pairwise meta-analysis that allows for the simultaneous comparison of multiple interventions. Uniquely, NMA makes use of ‘indirect evidence’ to draw comparisons between interventions that have not been directly tested against each other in a trial. Indirect evidence refers to the idea that, if two treatments A and B have not been compared in a trial, then we can infer their relationship via comparisons to some common third treatment C. That is, if we know that A is better than C (from trials comparing A to C) and that C is better than B (from trials comparing B to C), then we can infer that A is better than B, and by how much. By combining both direct and indirect evidence from all relevant trials, NMA produces a set of coherent comparisons between every pair of interventions.^13^ This makes efficient use of the evidence base and increases the precision of the relative intervention effect estimates. NMA is therefore an important method for presenting evidence about competing intervention options to support informed decision-making in clinical and policy contexts.^14^

Given the importance of reaching critical thresholds of vaccine coverage, it is crucial that policymakers have access to the most accurate and comprehensive evidence on which interventions are most effective at increasing vaccination rates. This ensures that resources are allocated effectively. We aim to address the gap between existing evidence and our understanding of the comparative effectiveness of different recipient-focused strategies in high and upper middle-income countries by conducting a comprehensive review and NMA of the evidence. We will explore variations in intervention effectiveness according to age, and evaluate whether the COVID-19 pandemic has influenced the effectiveness of these interventions.

## Methods

We conducted a comprehensive systematic review and NMA. The results reported in this paper are part of a larger project, which includes the development of a detailed intervention coding framework, a review of the evidence on intervention costs and economic, the protocol for which is registered with PROSPERO (https://www.crd.york.ac.uk/PROSPERO/view/CRD42022369139) and on the NIHR website.^15^ Results are reported in accordance with the PRISMA extension statement for network meta-analysis.^16^

### Selection criteria

Eligible studies were randomised controlled trials (RCTs; cluster or individually randomised) published since 2000 and conducted in high and upper-middle income countries, as defined by the World Bank as of July 2022.^17^ Studies had to randomise at least 100 participants, and cluster RCTs (CRCTs) required at least three clusters per arm. We restricted to large RCTs—the most robust evidence on intervention effectiveness—because of the substantial number of large primary studies conducted on this topic area.

### Participants

We included studies targeting all population groups living in the community in high and upper-middle income countries and eligible for vaccination (or carers of those eligible), including parents of young children, adolescents, and adults. Studies targeting hospital inpatients, prisoners, and residents of care/nursing homes were excluded. These populations may require different strategies to those living freely in the community. Studies focusing on increasing vaccinations in healthcare workers and in specific clinical risk groups were ineligible (Appendix A).

### Interventions

All routine and selective/targeted vaccinations on the UK immunisation schedule, including seasonal vaccinations were eligible for inclusion. There is a large overlap between international (e.g. WHO) and UK immunisation recommendations. Immunisations on the WHO schedule but not on the UK schedule are those for diseases typically seen in low income settings (e.g. cholera). The only other exception is the varicella vaccine which was not included on the UK immunisation schedule at the time of this review. We included varicella as a number of high income countries routinely offer the vaccination to children. See Appendix B.

We included any type of intervention targeted at the vaccine recipient or their caregiver that aimed to increase demand for, or access to, vaccination.

We excluded interventions aimed at the provider or healthcare system (e.g. provider training or incentives). Interventions aimed at both the intended vaccine recipients and providers or healthcare systems were also excluded, unless effectiveness data was available for the component targeting the intended recipients of vaccines alone.

### Comparator

Eligible studies used control groups of no intervention, usual care, waitlist or attention placebo. We also included ‘head-to-head’ trials which used an alternative eligible intervention (as defined above) as the comparator.

### Outcomes

Only studies that reported data on vaccine uptake were included. The outcome of interest was the number or proportion of people who received a vaccination. Some studies reported vaccination uptake for multiple different vaccines and/or for multiple different doses of vaccines. To ensure we used the data in a systematic way we developed the following hierarchy: 1. completion of a course of vaccinations (series completion); 2. having an up-to-date vaccination status (e.g. for childhood pre-school vaccinations the proportion of children who had received all the recommended vaccinations by a certain age); and 3. any dose (this could be part one dose of a course of vaccines, or single dose vaccines such as influenza (see Appendix B and C).

### Search strategy and screening

Searches were developed and run by an information specialist (SD). We searched MEDLINE-ALL (Ovid); PsycINFO (Ovid); Embase (Ovid); Cumulative Index to Nursing and Allied Health (CINAHL) (EBSCOHost); British Education Index (BEI) (EBSCOhost); Australian Education Index (AEI) (EBSCOhost); Educational Resources Information Center (ERIC); (EBSCOHost); Web of Science: Social Science Citation Index (SSCI) (Clarivate); Cochrane Central Register of Controlled Trials (Wiley) for RCTs conducted in higher and upper-middle income countries published from 2000 to 12th April 2024. A pragmatic search was conducted for grey literature, namely international trial registry protocols (ClinicalTrials.gov) on 18th April 2024 and theses and dissertations (Proquest Dissertations and Theses Global) on 24th June 2024. For full details see Appendix D.

Following the deduplication of search hits in Endnote, references were imported into Covidence (https://www.covidence.org/). Eligibility was independently assessed by pairs of reviewers with disagreements resolved by a third (SRD, CEF, IA, ZAT, or EA).

### Data extraction

Key data items (intervention characteristics, demographic data, and numeric outcome data) were independently extracted by two out of the following reviewers (SRD, ZAT, IA, EA, CEF) using a pre-defined, piloted Microsoft Access database. Other items (country, setting, vaccine) were extracted by one reviewer and checked for accuracy by a second. Data items that were documented within existing high-quality systematic reviews (e.g. Cochrane reviews—see Appendix E) were extracted from the primary study report(s) by one reviewer and then compared with the data presented within the existing review. Anywhere discrepancies were identified, the data item(s) were checked by a second reviewer. Where necessary, study authors were contacted for missing outcome or demographic data (sex, ethnicity, or age).

We used raw data extracted from the studies (e.g. number or proportion of participants receiving vaccinations) or effect estimates if no raw data were provided. Where outcome data were not provided in a useable form (e.g. no raw data or effect estimates provided), and the authors did not provide the data when contacted, the study was included in the review, but not the analyses.

Intervention classification was informed by the broad categories of the ‘5As’ model for the determinants of vaccine uptake,^18^ immunisation specialists at the UK Health Security Agency (UKHSA), and public contributors. Interventions were grouped as follows: access (interventions aimed at increasing access to vaccinations e.g. vaccines being provided at different locations, extended times e.g. weekends and early morning, opportunistic vaccinations, or providing accelerated dosing schedules); affordability (e.g. payment to cover costs, or offers of a financial incentive); education (education or information provided specifically to intended vaccine recipients or their caregivers in the form of letters, leaflets, educational campaigns, mainstream and social media campaigns, or face-to-face etc); reminders (delivered by any means, e.g. phone calls, text messages, letters); education and reminders (where both were equally prominent); multicomponent (interventions with features from three or more of the aforementioned categories; and interventions which incorporate features of two intervention categories where a prominent feature could not be identified–we assessed which was the most prominent feature by both assessing which aspect of the intervention the author highlighted in the title or methods of the study report(s) and assessing the content of the interventions to determine if one of the features held more weight. Where a prominent feature could be distinguished we categorised the intervention as such, where neither feature was prominent we categorised as multicomponent); and control (standard practices, no intervention, or attention placebo). Detailed definitions are provided in Appendix F.

### Public involvement

To integrate public involvement into this review we recruited 12 members of the public from diverse and underserved backgrounds.^19^ Meeting eight times, the group was highly engaged and contributed to multiple aspects of the review such as categorisation of interventions and interpretation of findings (see Appendix G).

### Risk of bias

We assessed risk of bias at the outcome level for each study included in the analyses using the Cochrane risk of bias 2 (RoB 2) tool.^20^ Studies were assessed independently by two of the following reviewers (SRD, ZAT, EA, CEF, TP), with discrepancies resolved through discussion, and involvement of a third reviewer as necessary. Where a study was included in a high-quality review, one reviewer assessed risk of bias and then compared their judgements to those presented in the existing review. Disagreements were resolved through discussion with a second reviewer.

### Statistical analyses

For synthesis, data were the numbers of events (and sample size) in each arm of each trial. For CRCTs we accounted for clustering by adjusting the number of events and participants per arm by the design effect (see Section 23.1.4 in^21^). Where possible, we specified the design effect in terms of the intraclass correlation coefficients (ICCs) reported in each trial. For studies that did not report an ICC we used an external estimate based on other CRCTs in this review. (Appendix H).

### Network meta-analysis

Our pre-specified primary analysis was an NMA combining all available direct and indirect evidence from the trials to estimate the relative treatment effects between every pair of interventions (see Appendix I). To do so, we assumed consistency between the relative treatment effects. Consistency means that, for each pair of treatments AB, the relative effect estimated from direct comparisons of A and B (in head-to-head trials) is equal to the relative effect estimated via indirect comparisons (from trials comparing each of A and B to some common third treatment C). Our pre-specified primary analysis was a random-effects model that allowed treatment effects to vary between trials and assumed a common heterogeneity parameter across effects. We fitted a fixed-effect model as a sensitivity analysis. We assumed a binomial likelihood for the number of events and modelled relative treatment effects as log odds ratios (LORs). For multi-arm trials, we specified a multivariate normal distribution for the relative treatment effects to account for correlations. We implemented the models in a Bayesian framework using JAGS,^22^ with non-informative prior distributions on all parameters. Further details of the model and its implementation are provided in Appendix I.

We assessed the NMA consistency assumption in three ways. First, we examined the plausibility of the assumption by comparing key effect modifiers across comparisons, focusing on the age of the vaccine recipients and whether the trial was conducted before or after the COVID-19 pandemic. Second, we examined empirical evidence of inconsistency via comparison with an unrelated mean effects model.^23^ Third, where direct comparisons were available, we conducted pairwise meta-analyses. Small-study effects were investigated using funnel plots^24^ and we performed sensitivity analyses; first, excluding studies judged to be at high risk of bias; second, excluding any studies with strongly outlying results; and thirdly without cluster adjustments (ICC = 0) and with larger ‘conservative’ ICC values (1 for households and 0.3 otherwise).

In case of inconsistency arising as a result of the two effect modifiers we conducted subgroup analyses to explore the impact of these differences. For the COVID-19 pandemic, we compared studies conducted prior to the pandemic (those with an end date of 2019 or before [‘pre-2020’]) with studies conducted during or after the pandemic (those studies with an end date of 2020 onwards [‘2020 onwards]) (Appendix I). For age, we compared studies conducted in different age groups (Young children–routine vaccinations, children—seasonal vaccines, adolescents/young adults, adults, pregnant women, older adults—see Appendix B for further details of age groups).

### Confidence in the evidence

We assessed confidence in the evidence using the Confidence In Network Meta-Analysis (CINeMA) approach,^25^ implemented using the web-based application which enables semi-automated assessments.^26^ Members of the review team (CEF, SRD, JPTH) evaluated the evidence for each intervention vs. control comparison considering within study bias, reporting bias, indirectness, imprecision, heterogeneity, and incoherence.

### Role of the funding source

The funder of the study had no role in study design, data collection, data analysis, data interpretation, or writing of the manuscript and the decision to submit.

## Results

### Results of the search

**Fig. 1:**
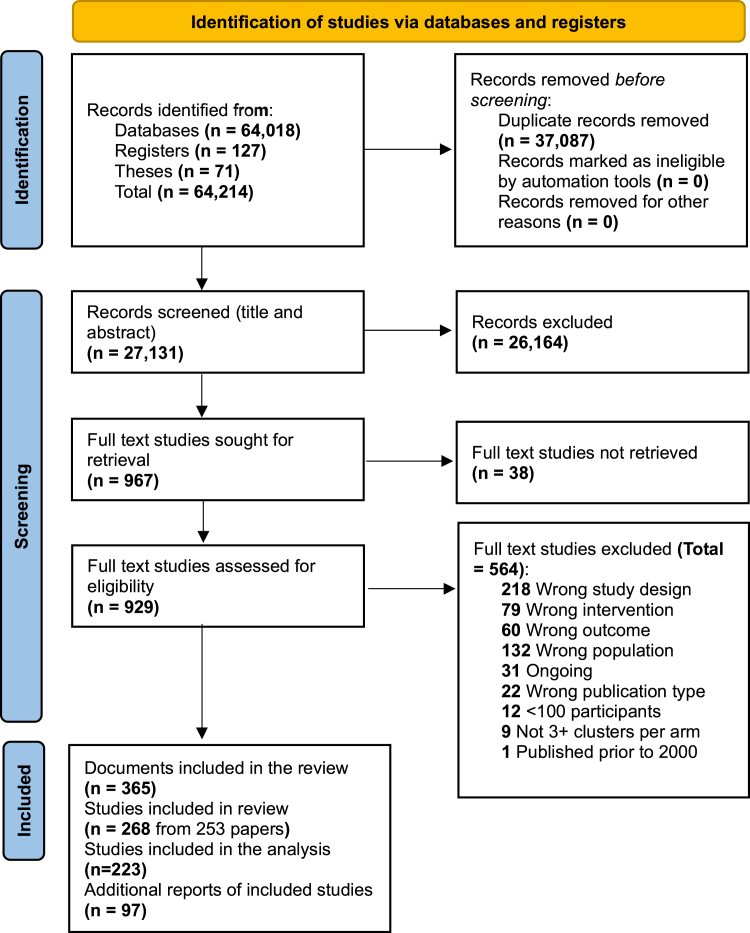
**PRISMA flow chart of systematic review process**.

We screened 27,131 titles and abstracts and assessed 967 full-text articles for inclusion (Fig. 1). Two-hundred and sixty-eight studies (from 253 papers) including 6,243,118 participants (male = 2,434,816 (39%), female = 3,808,302 (61%)) met our eligibility criteria. We were unable to include forty-five of these studies in the analyses, either because the outcome data were not in a useable format, or because all intervention arms were categorised as the same intervention type. The characteristics of these studies and reasons for non-inclusion in the analyses are in Appendix J.

### Study characteristics

Full study characteristics for the 223 studies included in the analyses are in Appendix K and summarised in Table 1. The included studies were published between 2000 to 2024 and included between 100 and 964,870 participants. Information on ethnicity of trial participants was reported in 39% of studies, religion in 5%, education in 45% and socio-economic data (in various formats) in 71% (see Appendix L). The most commonly reported vaccine for each age group was as follows: for young children–routine vaccinations all 34 trials reported on uptake of childhood vaccines; for children-seasonal vaccines 24/25 trials (96%) reported on influenza; for adolescents 43/54 trials (80%) reported on HPV; for adults 26/65 trials (40%) reported on influenza and 18/65 trials (28%) reported COVID-19; for pregnant women 10/11 trials (91%) reported on influenza; and for older adults 26/34 trials (77%) reported on influenza.

**Table 1:** Summary of study characteristics.

	N (%) of studies included in NMA (n = 223)
**Study type**	
Individually randomised controlled trials	170 (76%)
Cluster randomised controlled trials	53 (24%)
**Participants**	4,335,827
Male	1,972,618 (45.5%)
Female	2,363,208 (54.5%)
**Country**	
**High income**	**208 (93%)**
Australia	9 (4%)
Canada	3 (1.3%)
European countries	37 (16.6%)
Hong Kong Special Administrative Region (SAR), China	7 (1.8%)
Israel	1 (0.5%)
Japan	3 (1.3%)
New Zealand	1 (0.5%)
Singapore	1 (0.5%)
Taiwan, China	1 (0.5%)
United States	145 (65%)
**Upper middle income**	**15 (7%)**
Brazil	1 (0.5%)
China	12 (5%)
Georgia	1 (0.5%)
Guatemala	1 (0.5%)
**Age/vaccination group**	
Young children–routine vaccinations (0–5 years)	34 (15%)
Children–seasonal vaccinations	25 (11%)
Adolescents/young adults	54 (24%)
Adults	65 (29%)
Pregnant women	11 (5%)
Older adults	34 (15%)
**Setting**	
Healthcare	147 (66%)
Community/other	31 (14%)
Education	24 (10%)
Online	21 (10%)
**Intervention arms**	
Total no. of arms N = 260	
Access	14 (5%)
Affordability	14 (5%)
Education	85 (33%)
Reminders	78 (30%)
Education and reminders	54 (21%)
Multicomponent^a^	15 (6%)
**Outcome**	
Series completion	56 (25%)
Up-to-date vaccination	7 (3%)
First dose	12 (6%)
Any dose	148 (66%)
**Timepoint**	
Pre- 2020	165 (74%)
2020-onwards	58 (26%)

Footnote: young children—routine childhood vaccinations' captures routine childhood vaccinations given to pre-school aged children (0–5 years); ‘children—seasonal vaccinations’ pertains to children of any age offered seasonal vaccinations; ‘adolescents/young adults’ captures immunisations aimed at individuals aged 10–19 years but includes catch up and other campaigns for HPV vaccines that included young adults up to their mid-20s; ‘pregnant women’ captures vaccinations offered during pregnancy; ‘adults’ captures seasonal and selective vaccinations aimed at the general adult population; ‘older adults’ captures immunisations aimed at those 65 and above. We did not adhere to strict age cut-offs but were guided by the vaccination types being offered to the trial participants.

a Further details on the content of the multicomponent interventions are provided in Appendix M.

We judged 105 studies to be at low risk of overall bias, 87 to have some concerns, and 31 to be at high risk of bias, mainly due to issues with randomisation and measurement of the outcome (Appendix N).

**Fig. 2:**
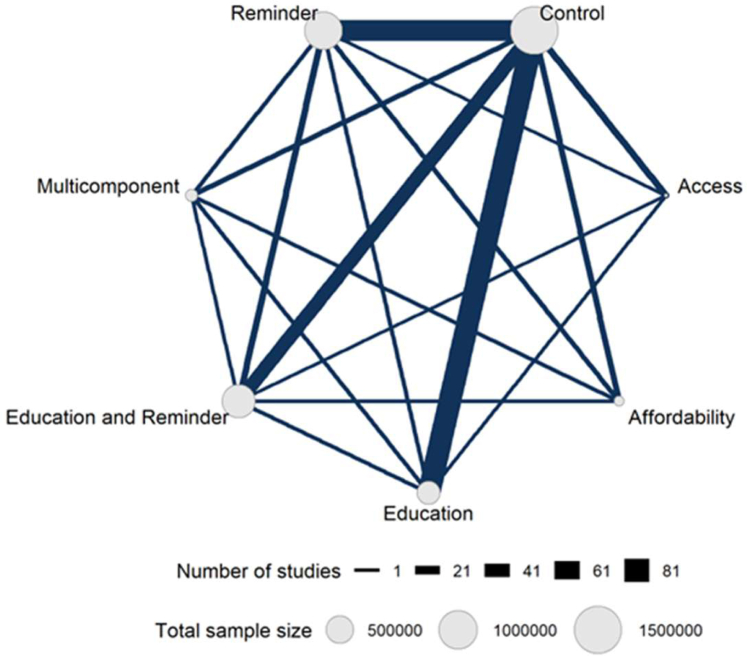
**Network plot visualising the Network meta-analysis of eligible interventions to increase vaccine uptake. Network graph created using the multinma package. Nodes(circles) represent interventions, edges (links between nodes) represent direct comparisons between treatments in trials. The thickness of an edge connecting two nodes is proportional to the number of trials comparing those two interventions. The size of node indicates the number of randomly assigned participants receiving that intervention.**

**Fig. 3:**
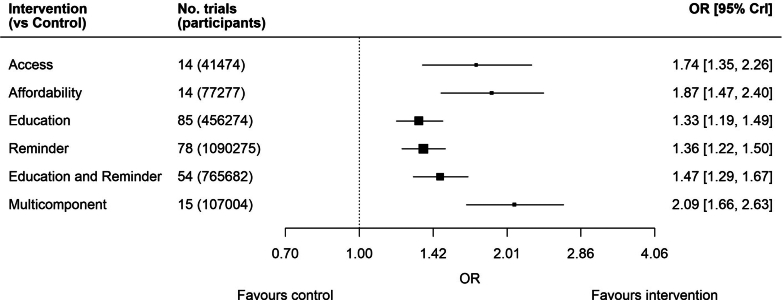
**Vaccine uptake by intervention type relative to control**.

Our network meta-analysis is represented visually in a network plot in Fig. 2. Results of the network meta-analysis show that the odds ratios (ORs) and 95% credible intervals (CrI) for all six intervention categories were above 1, indicating that all six intervention types were effective at increasing vaccine uptake compared with control. The interventions multicomponent (OR: 2.09, 95% CrI: 1.66–2.63), access (OR: 1.74, 95% CrI: 1.35–2.26), and affordability (OR: 1.87, 95% CrI: 1.47–2.40) appeared to be most effective at increasing uptake, followed by education and reminder (OR: 1.47, 95% CrI: 1.29–1.67), reminder (OR:1.36, 95% CrI:1.22–1.50), and education (OR: 1.33, 95% CrI: 1.19–1.49) interventions (Fig. 3).

### Confidence in the evidence.

We rated the confidence in all six effect estimates comparing intervention with control as ‘Moderate’. We downgraded by one level due to a combination of some concerns about heterogeneity (for all six interventions) and some concerns about within study bias (access and affordability interventions) or incoherence (affordability, education and reminder interventions). Details in Appendix O.

The heterogeneity parameter (median and 95% confidence interval (CI)) on the LOR scale was estimated as τ = 0.42 [0.37, 0.49]. This means that approximately 95% of the trial specific ORs lie within a multiplicative factor of exp(1.96×0.42)=2.27 of the NMA OR estimates. Based on comparison with an unrelated mean effects model, we found no evidence of inconsistency in the data (Appendix P).

The results of the pairwise meta-analyses support those of the NMA, with all six interventions showing favourable uptake compared with control, and multicomponent, access and affordability showing the largest effects (see forest plots in Appendix Q). Funnel plots for all pairwise meta-analyses with more than five trials are shown in Appendix R. In tests for asymmetry, all meta-analyses of interventions vs. control (except multi-component) showed evidence (p < 0.0001) of small-study effects. However, the asymmetry cannot be assumed to arise from publication bias since asymmetry may arise for other reasons.^27^ In particular, the funnel plots are consistent with intervention effects being truly smaller in larger studies.

### Subgroup analyses

**Fig. 4:**
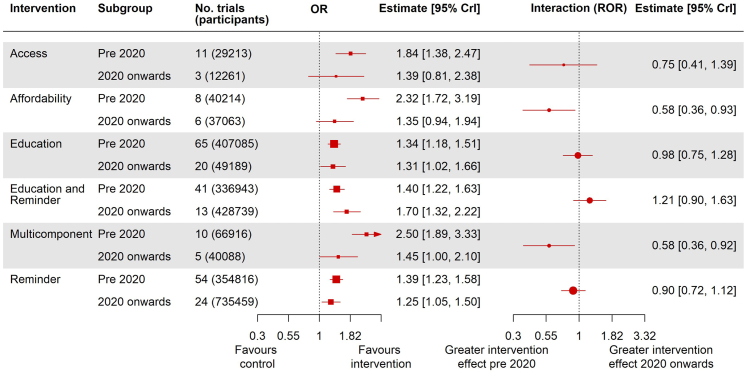
**Subgroup analysis exploring the impact of the COVID-19 pandemic comparing studies conducted pre-2020 and those conducted from 2020 onwards on intervention effectiveness compared to control. The left-hand plot shows the individual subgroup effects on the OR scale and the right-hand plot shows the interactions. On this scale, each interaction is a ratio of odds ratios (ROR) of 2020 onwards versus pre-2020**.

Results of our COVID-19 subgroup analysis show that the effects of access, education, reminder, and education and reminder interventions remained stable following the onset of the pandemic, however, there was an interaction effect (interaction estimate and credible intervals that do not pass the line of no-effect) between time period and affordability (ratio of odds ratios (ROR): 0.58, 95% CrI: 0.36–0.93) with affordability interventions being more effective pre-2020 (OR: 2.32, 95% CrI: 1.72–3.19) than 2020 onwards (OR: 1.35, 95% CrI: 0.94–1.94). We also observed an interaction in the same direction for multicomponent interventions (ROR: 0.58, 95% CrI: 0.36–0.92), with multicomponent interventions being more effective relative to control pre-2020 (OR: 2.50, 95% CrI: 1.89–3.33) than 2020 onwards (OR: 1.45, 95% CrI: 1.00–2.10) (Fig. 4).

**Fig. 5:**
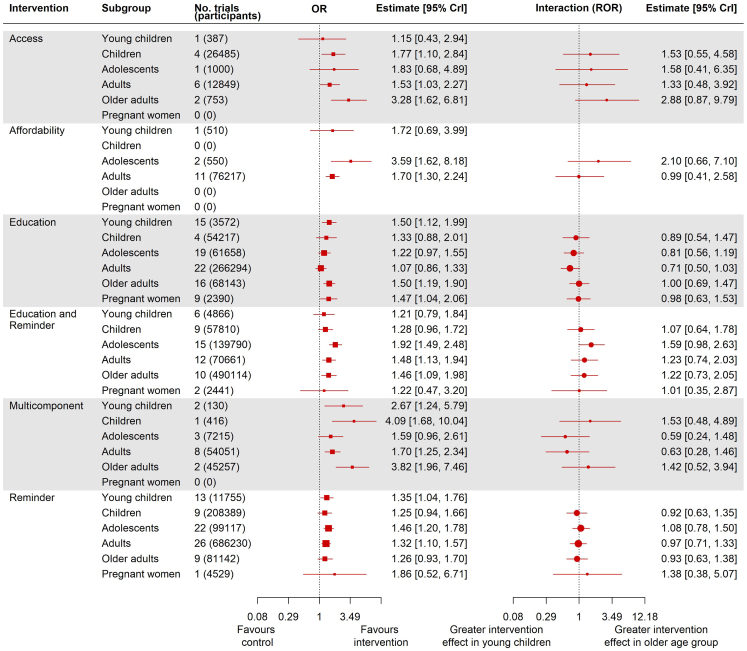
**Subgroup analysis exploring intervention effectiveness across age groups**.

Fig. 5 shows our post-hoc subgroup analysis exploring whether intervention effects varied according to age group. The strongest evidence in favour of education was for young children and older adults. Evidence for multicomponent interventions was limited, but they appear effective across age groups. For reminder, and education and reminder interventions adolescents and adults have the most contributing evidence and the strongest effects. For access and affordability interventions there were insufficient data to draw clear conclusions across age groups.

### Sensitivity analyses

Results for the fixed-effect model followed a similar pattern to the random-effects model, though the model exhibited a much poorer fit to the data (a deviance information criterion (DIC) of 2680 compared with 698 for the random-effects model), providing evidence for heterogeneity between trials (Appendix S).

When studies at high risk of bias were excluded, the results were similar to the primary analysis. In this analysis, all interventions were more effective than control, with the same three intervention types appearing most effective: multicomponent (OR: 2.06, 95% CrI: 1.60–2.67), affordability (OR: 1.88, 95% CrI: 1.47–2.41), and access (OR: 1.85, 95% CrI: 1.36–2.54) (Appendix S). After removing five studies with strongly outlying results, the findings remained stable (Appendix S). Findings were also robust to sensitivity analyses exploring assumed ICC values, showing the same pattern both without cluster adjustment and with large ICCs (Appendix S).

## Discussion

This network meta-analysis represents the most comprehensive synthesis of trials of vaccine uptake interventions targeting the intended recipients or their caregivers in high and upper-middle income countries. Moderate certainty evidence showed that all six intervention types (access, affordability, education, education and reminders, and multicomponent) were effective at increasing vaccine uptake compared with control. Interventions aimed at tackling the barriers of affordability (covering costs or providing incentives), accessibility/convenience of vaccinations, and interventions incorporating two or more prominent intervention strategies were the most effective, although these categories had the fewest studies contributing direct comparisons to the analysis. The effect of multicomponent interventions is hard to interpret, although they might be particularly effective due to them tackling multiple barriers to vaccination. These findings were robust to sensitivity analyses (removal of studies at high risk of bias, of strong outliers, and explorations of assumed ICC).

These findings provide an important addition to the growing body of evidence examining interventions to increase vaccine uptake. Previous syntheses tend to be highly focussed, examining one vaccine (e.g. influenza)^8^; intervention (e.g. reminders)^9^; or population (e.g. adolescents).^28^ Our findings are consistent with two recent broader reviews with similar criteria to ours. One review that also includes lower income countries and provider focused interventions, found that interventions to increase access and provide incentives are the most effective strategies.^11^ However, the searches were not comprehensive with no grey literature searches conducted and only 88 studies were included compared with our 268, despite their broader scope. The other review similarly found that all included interventions were more effective than control, with on-site vaccinations being the most effective.^12^ Information on inclusion criteria was however unclear, searches were completed on a single database, and no study characteristics were provided. A recent suite of reviews also found that access interventions were more effective than controls. Incentives were also found to be particularly effective to support the HPV vaccination programme, though this was based on only two studies. Education was effective in this suite of reviews, but this was only apparent in the over-65s.^10^

The timing of this review allowed us to conduct some early examinations of the impact of the COVID-19 pandemic on the effectiveness of vaccine uptake interventions. Subgroup analyses comparing studies conducted pre-pandemic to those conducted from 2020 onward provide some preliminary evidence that the pandemic may have impacted affordability and multicomponent interventions, with both appearing less effective since the onset of the pandemic. However, this could be specific to COVID-19 vaccines, as half of the affordability data from 2020 onwards relates specifically to COVID-19 vaccine uptake. Affordability includes incentives and payments to cover costs and at a time when people are struggling with a cost of living crisis, it might be expected that affordability interventions may be particularly effective. Whilst some studies have reported small positive effects of financial incentives for COVID-19 vaccines, others suggest that financial incentives for COVID-19 vaccines may have backfired.^29^^,^^30^ Our public involvement group, made up of individuals from underserved communities, further suggested that they may be likely to distrust incentives—seeing them as potential bribes. The impact on multicomponent interventions is harder to interpret. These subgroup analyses provide an early exploration of the impact of the pandemic on efforts to increase vaccine uptake. The findings should be interpreted with caution, due to the small number of studies post-2020 and the fact that the pandemic was a unique time. Nevertheless, they highlight the need for the situation to be monitored over the coming years to assess the potential for long-term impacts and allow delivery programmes to be adjusted accordingly.

Exploration of age-group effects showed generally positive effects for education, with the strongest evidence being for young children and older adults. For reminder, and education and reminder interventions, adolescents and adults had the most contributing evidence and the strongest effects. These interventions may be particularly effective in adolescents due to the vaccinations recommended for this age group. The majority of adolescent studies were on the HPV vaccine, for which lack of awareness and information are some of the most critical known barriers to uptake.^31^ It follows that education and information-based interventions may work to remove this barrier. For access and affordability interventions there were insufficient data to draw strong conclusions across age groups, highlighting the need for more investigation of these interventions.

Our findings should be considered in context of several strengths and limitations. Our search was very comprehensive and included 10 studies identified only through grey literature sources. Our inclusion criteria were broad ensuring the findings are widely generalisable. Whilst we included studies reporting on immunisations on the UK immunisation schedule, there is a large overlap between international (e.g. WHO) and UK immunisation recommendations for high and upper-middle income countries. Using the WHO schedule would make no difference to the studies included in this review.

We included studies conducted in high and upper middle-income countries, the geographical distribution studies was reasonable representing 27 countries, however a limitation of the available data is that 65% (N = 145) of studies were from the United States which may be relevant when considering the findings, particularly in countries with different healthcare systems and healthcare funding. Our exclusion of studies from lower income countries means these findings are not applicable to these settings. Such settings differ from higher income countries on several dimensions, including infrastructure, funding, accessibility, disease focus, and public trust and subsequently may require different strategies and would be better examined separately.

We extracted and report data on PROGRESS items (place of residence, race/ethnicity, gender/sex, education, and socio-economic status) as available in the trials. There were, however, significant shortcomings in included studies in terms of quality, completeness, and consistency of reporting of these characteristics that are known to be associated with inequalities in vaccination uptake.^32^ It was not possible to stratify the analyses by gender/sex or race/ethnicity as disaggregated data was insufficiently reported across the trials.

Our intervention categories grouped similar strategies together e.g. different ways of improving access to vaccinations, or different educational strategies. As would be expected given the diversity of interventions, our CINeMA assessment identified ‘some concerns’ regarding heterogeneity within intervention categories. Additionally, a proportion of interventions were categorised as multicomponent–including prominent strategies from two or more intervention categories–and are therefore difficult to interpret. A limitation of this approach is that we cannot parse the individual effects of different features of these broad intervention types, for example we cannot state whether financial incentives are more or less effective than payments to cover costs involved in receiving vaccinations, nor can we state which type (e.g. lottery or guaranteed) or amount of incentive is best. Future work will explore this heterogeneity in-depth by examining effective components of interventions.

Uniquely, our analytical approach allowed us to simultaneously compare each intervention type in a single model using both direct and indirect comparisons. We restricted our review to the strongest evidence available, RCTs, given the large number available studies. However, this may have led to some evidence–particularly for some types of interventions or population groups—being excluded. All interventions included in NMAs should be ‘jointly randomizable’, and it can be a limitation of the approach if this is not the case, however we believe that all the interventions in this NMA are genuine options for any population group.

We observed a relationship between magnitudes of effect and study sizes in our funnel plots. Although publication bias could be operating, such a relationship can also be explained by smaller studies implementing more intensive interventions with larger impacts on vaccine uptake.

In conclusion, our findings indicate that vaccination programmes should prioritise ensuring vaccines are accessible, convenient, and affordable for the recipient. Efforts should be directed towards further investigating and optimising access and affordability interventions. Future trials should incorporate analyses by ethnicity and socio-economic factors which are currently lacking but would inform decision making and help address inequities in vaccination. The possible negative impacts of the COVID-19 pandemic on the effectiveness of affordability and multicomponent interventions should be monitored. Our findings are of value for those developing and optimising interventions focused on vaccine recipients and should be considered within the context of the wider healthcare systems in which immunisation programmes operate.

## Contributors

CEF, JPTH, DMC, LY, LL and JY conceived and designed the study. SD developed and ran all searches. SRD, ZAT, IA, EA, and CEF selected the articles and extracted data. SRD, TP, ZAT, EA and CEF completed risk of bias assessments. CM led on public involvement. ZAT created and maintained the Access database. SRD, CEF and ALD directly accessed and verified the underlying data. ALD conducted the statistical analyses with input from JPTH, DMC, CEF and SRD. All authors interpreted the results. SRD wrote the first draft of the manuscript. All authors provided comments and critical revisions on drafts of the manuscript and approved on the final text. All authors had full access to all the data in the study and had final responsibility for the decision to submit for publication.

## Data sharing statement

All data and codes for analysis are available on the GitHub repository: https://github.com/AnnieDavies/VaccineReview.

## Declaration of interests

CEF was an invited speaker at the UK Clinical Vaccine Network Conference 2024 receiving travel expenses from the conference organising committee. JPTH received grant funding from the World Health Organisation to examine observational evidence on COVID-19 vaccine efficacy (July 2021 to June 2022), paid to University of Bristol. The remaining authors declare no competing interests.
