# Supplementary material for: Recipient-focused interventions to increase vaccine uptake in high and upper-middle income countries: a systematic review and network meta-analysis
Source: eClinicalMedicine. 2025 Nov 18;90:103643. doi: 10.1016/j.eclinm.2025.103643 (PMC12666792; doi:10.1016/j.eclinm.2025.103643)
Supplement: Supplementary Figure and Tables [file mmc1.docx]

**Contents**

[**A: Post-hoc clarifications to the protocol** 2](#_Toc213846007)

[**B: Age group/vaccination and vaccine outcome hierarchy** 2](#_Toc213846008)

[**C: Decision rule for selection of outcome data** 3](#_Toc213846009)

[**D: Search Strategy** 3](#_Toc213846010)

[*Ovid MEDLINE search* 4](#_Toc213846011)

[**E: List of high-quality reviews used for data checking** 13](#_Toc213846012)

[**F: Intervention and control categories used in this review** 13](#_Toc213846013)

[**G: Public involvement group** 14](#_Toc213846014)

[**H: Cluster adjustment** 15](#_Toc213846015)

[*Choice of ICC* 15](#_Toc213846016)

[*Cluster size assumptions* 15](#_Toc213846017)

[**I: Statistical model** 15](#_Toc213846018)

[*Subgroup analyses* 16](#_Toc213846019)

[*Model implementation* 16](#_Toc213846020)

[**J: Study characteristics of descriptive only studies** 17](#_Toc213846021)

[**K: Study characteristics of studies included in analyses** 26](#_Toc213846022)

[**L: Socio-demographic and socio-economic characteristics of study participants** 36](#_Toc213846023)

[**M: Multicomponent interventions included in the NMA** 54](#_Toc213846024)

[**N: Risk of bias assessment for included studies** 59](#_Toc213846025)

[**O: Confidence in the evidence** 65](#_Toc213846026)

[**P: Checks for inconsistency** 66](#_Toc213846027)

[**Q: Pairwise meta-analyses: results** 67](#_Toc213846028)

[**R: Funnel plots to look for small study effects from pairwise MAs with five or more studies** 75](#_Toc213846029)

[**S: Sensitivity analyses** 79](#_Toc213846030)

[*Sensitivity analyses: Fixed effects* 79](#_Toc213846031)

[*Sensitivity analyses: Removal of studies at high risk of bias* 79](#_Toc213846032)

[*Sensitivity analyses: Removal of outliers* 79](#_Toc213846033)

[*Sensitivity analysis: No cluster adjustments* 80](#_Toc213846034)

[*Sensitivity analysis: High ICC - larger ICC values (1 for households and 0.3 otherwise)* 81](#_Toc213846035)

[**T: References to included studies** 81](#_Toc213846036)

[**U: List of excluded studies at full text with brief reasons** 91](#_Toc213846037)

## **A: Post-hoc clarifications to the protocol**

In our eligibility criteria we clarify that we excluded studies carried out on specific clinical risk groups. This decision was made following discussion within the project team, including immunisation experts, and was based on the fact that interventions to increase uptake in clinical risk groups are likely to differ from those conducted in healthy populations in a number of ways, for example, they may frequently be carried out in secondary care settings and may not be applicable to the wider population. It is also possible that the barriers to vaccination are somewhat different in at-risk groups, with disease-specific factors such as concerns around the risk of adverse events or exacerbation of the underlying conditions potentially playing a role.

## **B: Age group/vaccination and vaccine outcome hierarchy**

| **Age group** | **Age due** | **Vaccines recommended** | **Vaccine hierarchy** |
| --- | --- | --- | --- |
| Young children – routine childhood vaccinations | Eight weeks | DTaP/IPV/Hib/HepB MenB  Rotavirus | Up-to-date vaccination data for the whole schedule of vaccines was selected where available over individual vaccines. Where individual vaccine data was used, we extracted the primary vaccination data as specified by the study authors. |
|  | Twelve weeks | DTaP/IPV/Hib/HepB  Pneumococcal conjugate vaccine (PCV)  Rotavirus |  |
|  | Sixteen weeks | DTaP/IPV/Hib/HepB  Rotavirus  MenB |  |
|  | One year | Hib/MenC  PCV booster  MMR  MenB booster |  |
|  | Three years, four months old | MMR  dTaP/IPV |  |
| Children – seasonal vaccinations | Eligible paediatric age groups | Influenza  COVID-19 | Influenza was selected over COVID-19. |
| Adolescents/young adults | Boys and girls aged twelve to thirteen years | HPV | HPV chosen where available. If not available, then authors’ primary outcome was chosen. |
|  | Fourteen years old | Td/IPV (check MMR status)  MenACWY |  |
| Adults |  | COVID-19  Influenza  Selective vaccinations such as Hepatitis A/B | Influenza was chosen first, followed by COVID-19. For selective vaccinations Hepatitis B was chosen over A. |
| Pregnant women | At any stage of pregnancy during flu season | Influenza | Influenza chosen where available. If not available, then Pertussis. |
|  | From 16 weeks' gestation | Pertussis |  |
| Older adults | 65 years old | Pneumococcal Polysaccharide Vaccine (PPV23) | Influenza was chosen first where available. Where not available we used the author’s primary outcome. |
|  | 65 years and older | Inactivated influenza vaccine |  |
|  |  | Shingles vaccine |  |

Footnotes:

Abbreviations:

DTaP/IPV/Hib/HepB - Diphtheria, tetanus, pertussis (whooping cough), polio, haemophilus influenzae type b (Hib) and hepatitis B

MenB - Meningococcal group B

MMR – Measles, mumps and rubella

MenACWY **-** Meningococcal groups A, C, W and Y

1. This review refers to the UK routine vaccination schedule. The November 2019 schedule was in use when this review was initiated and is available with the current version of the complete routine immunisation schedule (<https://www.gov.uk/government/publications/immunisation-schedule-the-green-book-chapter-11>). We used the schedule as a guide for the types of vaccinations to include in the review and to inform the development of our age groupings. However, since this review incorporates studies from all high and upper-middle income countries, which may use differing immunisation schedules, we did not strictly adhere to it. For example, the United States immunisation schedule includes an annual influenza vaccination for all adults – trials on influenza vaccination uptake among adults were not excluded. Also, noting that a number of high-income countries routinely offer varicella vaccination to children, trials reporting on interventions to increase uptake of varicella vaccination were eligible for inclusion.

2. In this review, the term pregnant woman is used to include women who are pregnant as well as transgender or non-binary people who are pregnant. This terminology is used to maintain consistency with NHS websites.

3. Age categories were ‘young children – routine childhood vaccinations’ which captures routine childhood vaccinations given to pre-school aged children (0-5 years); ‘children – seasonal vaccinations’ pertains to children of any age offered seasonal vaccinations; ‘adolescents and young adults’ captures immunisations aimed at individuals aged 10 to 19 years but includes catch up and other campaigns for HPV vaccines that included young adults up to their mid-20s; ‘pregnant women’ captures vaccinations offered during pregnancy; ‘adults’ captures seasonal and selective vaccinations aimed at the general adult population; ‘older adults’ captures immunisations aimed at those 65 and above. We did not adhere to strict age cut-offs but were guided by the vaccination types being offered to the trial participants.

## **C: Decision rule for selection of outcome data**

For our network meta-analysis model, we needed to extract one data point for each intervention arm. Some studies reported vaccination uptake for multiple different vaccines and/or for multiple different doses of vaccines. To ensure we extracted the data in a systematic way we developed the following hierarchy. The primary outcome of interest in the trials was the number of people who received a vaccination. Vaccinations are given in either single-dose or multi-dose schedules. Vaccination can therefore refer to having had at least one vaccination (any dose), having completed a course of vaccinations (series completion) or having an up-to-date vaccination status (timely) for example with childhood vaccinations timely vaccination might be the number/percentage of children who had received all recommended vaccinations by the age of 2 years. We used the trial authors definitions of timely vaccinations. For single-dose vaccines such as influenza, ‘any dose’ is the same as ‘series completion’. Where more than one of these outcomes were reported in a study, we used the following hierarchy to extract data and include in our analysis:

1. Series completion
2. Timely
3. Any dose

## **D: Search Strategy**

*Search Methods*

A comprehensive two-stage search strategy was developed, built around existing high-quality systematic reviews on interventions to increase vaccine uptake. This included existing Cochrane and NICE evidence reviews captured from scoping searches of the following resources: National Institute for Health and Care Excellence (NICE) website; NIHR Journals Library; Cochrane Database of Systematic Reviews; Epistemonikos; Database of Promoting Health Effectiveness Reviews (DoPHER); Health Evidence (McMaster University); and Ovid MEDLINE, from 2014 to 20 September 2022. We checked search strategies and extracted studies included in relevant systematic reviews or guidelines. We organised these studies into high level themes/intervention types, then built bespoke search strategies to (at least) re-capture these studies and hence other similar ones, published from 2000 onwards. A study publication date limit of 2000 onwards was used for this review because this will focus on studies published after the MMR scandal of 1998 when attitudes to vaccinations changed and the numbers of vaccination related studies increased greatly.

The search for primary studies was conducted across nine bibliographic databases. Where appropriate the search was structured around the concepts of vaccination and types of intervention to increase uptake, organised by themes: access; reminders; education; infrastructure. A parallel search strand including types of intervention combined with vaccines of interest was also conducted. The latest search was conducted on the 12 April 2024 on the following databases, limited to RCTs in high and upper-middle income countries:

• MEDLINE-ALL (Ovid);

• PsycINFO (Ovid);

• Embase (Ovid);

• Cumulative Index to Nursing and Allied Health (CINAHL) (EBSCOHost);

• British Education Index (BEI) (EBSCOhost);

• Australian Education Index (AEI) (EBSCOhost);

• Educational Resources Information Center (ERIC) (EBSCOHost);

• Web of Science: Social Science Citation Index (SSCI) (Clarivate);

• Cochrane Central Register of Controlled Trials (Wiley).

The search for primary studies was first initiated in October 2022, and at this time, the search was broad, aiming to capture experimental studies beyond RCTs, including quasi-experimental studies, controlled before and after and interrupted time series. Due to the scale of the task (lines of search code, database processing speeds, number of records retrieved etc), the searches were conducted and results screened on a theme-by-theme basis (four themes x nine databases). However, once the decision was made to only include RCTs in the review (see amendment to protocol on PROSPERO CDR42022369139), all themes were consolidated into one search per database. See below for the Ovid MEDLINE search.

A separate, pragmatic search was conducted for grey literature items, namely international trial registry protocols (ClinicalTrials.gov) (18-April-2024) and theses and dissertations (Proquest Dissertations and Theses Global) (24-June-2024).

### *Ovid MEDLINE search*

**Ovid MEDLINE(R) ALL** <1946 to April 12, 2024>

Update – RCTs – All Interventions

[*Vaccination*]

1 exp Vaccination/ 113921

2 immunization/ 54143

3 immunization schedule/ 11551

4 Immunization programs/ 13050

5 (immuni#ation? or vaccination? or revaccination? or reimmuni#ation?).tw,kf. 295082

6 (vaccin* adj (uptake? or coverage)).tw,kf. 18822

7 or/1-6 349611

[*Access Interventions*]

8 (*Vaccination/ or *Immunization/) and "Organization & Administration".fs. 1551

9 *Preventive Health Services/ 9142

10 Health Services Accessibility/ 86791

11 ("after hours" or "out of hours" or (extend* adj2 hours)).tw,kf. 4792

12 ((extend* or weekend? or early or earlier or late or later or evening? or commuter?) adj4 (clinic? or service? or appointment? or session?)).tw,kf. 13860

13 Mobile Health Units/ 3943

14 ((mobile adj4 (clinic? or health unit? or health care or healthcare or health van? or hospital? or site or sites)) or field hospital?).tw,kf. 4940

15 (temporary adj4 (centre? or center? or clinic? or facility or facilities or health care or healthcare or health unit? or service? or site or sites)).tw,kf. 979

16 (walkin or "walk in" or popup or pop-up or popin or "pop in" or "pop by" or "drop by").tw,kf. 5196

17 ((dropin or "drop in") adj4 (centre? or center? or clinic? or facility or facilities or health unit? or service? or site or sites)).tw,kf. 748

18 (drive-in or drive-through).tw,kf. 2710

19 (supermarket? or grocery store? or drug store? or instore? or in-store? or shopping cent* or retail unit? or mall or malls or out-of-town).tw,kf. 12959

20 (sporting venue? or stadium? or stadia or arenas).tw,kf. 4944

21 ((urban or rural) adj4 (access* or availability)).tw,kf. 4404

22 (escort* or group visit*).tw,kf. 2260

23 (health* adj (visit* or supervis*)).tw,kf. 6301

24 Safety-net Providers/ 1391

25 ((safety-net or safetynet) adj3 (clinic? or hospital? or provider? or system? or health care or healthcare)).tw,kf. 3581

26 exp Community Participation/ 48588

27 Community Health Centers/ or Community Health Services/ or exp Community Health Nursing/ or Community Mental Health Services/ or Community Networks/ 84585

28 Public Health Nursing/ 10260

29 ((community or public or civic or communal or municipal) adj4 (facility or facilities or provider? or setting? or venue* or located or location? or building* or regional* or clinic? or hall or halls or centre* or center* or space or spaces or site or sites)).tw,kf. 105266

30 (communit* adj4 (advoc* or awareness or champion* or compliance or educat* or engag* or involv* or participat* or uptake?)).tw,kf. 56020

31 (community group? or (communit* adj4 (activ* or campaign* or implement* or intervention or policy or policies or program* or pilot or strategy or strategies or workshop?))).tw,kf. 67151

32 ((community or community health*) adj (care or unit? or nurs* or service? or volunteer*)).tw,kf. 16300

33 (community adj4 (dwelling or based or delivered or located or location? or led or run or set or setting?)).tw,kf. 149067

34 (communit* adj4 (rural or urban or inner city)).tw,kf. 30674

35 Substance Abuse Treatment Centers/ 5427

36 ((drug? or substance?) adj4 (abuse or treat* or rehab*) adj4 (centre? or center? or clinic? or facility or facilities or health care or healthcare or health unit? or service? or site or sites)).tw,kf. 6298

37 ((family or families or carer? or parent* or office or work*) adj (based or delivered)).tw,kf. 23284

38 Community Pharmacy Services/ or Pharmacy/ 16242

39 (pharmacy or pharmacies or pharmacist?).tw,kf. 91295

40 *General Practice/ or *Family Practice/ 52346

41 (((general practi* or family practi* or primary care) adj7 (intervention? or program* or project? or service? or study or trial)) and (access* or uptake? or cover* or rate or rates or visit*)).tw,kf. 23627

42 Family Nursing/ or Maternal-Child Nursing/ 3512

43 Prenatal Care/ or Perinatal care/ or Maternal Child Health centers/ 40181

44 Peripartum Period/ or Postpartum Period/ 33712

45 ((prenatal or pre-natal or antenatal or ante-natal or pregnan* or perinatal or peri-natal or postpartum or post-partum) adj4 (care or centre? or center? or clinic? or facility or facilities or health care or healthcare or health unit? or service? or site or sites)).tw,kf. 60062

46 (mother? adj4 (baby or babies) adj4 (care or centre? or center? or clinic? or facility or facilities or health care or healthcare or health unit? or service? or site or sites)).tw,kf. 613

47 ((midwif* or widwiv*) adj4 (based or delivered or led or run or care or centre? or center? or clinic? or facility or facilities or health care or healthcare or health unit? or service? or site or sites)).tw,kf. 4021

48 (mother? adj4 program*).tw,kf. 2264

49 (well-child adj (care or clinic)).tw,kf. 794

50 ((women adj2 Infants adj2 children) or (WIC adj3 (program* or service? or support*))).tw,kf. 1731

51 exp Child Day Care Centers/ 6326

52 ((child or children* or day or daycare) adj4 (centre? or center?)).tw,kf. 19604

53 Schools, Nursery/ 1512

54 ((nursery or nurseries or kindergarten* or preschool* or pre-school*) adj4 (based or delivered or located or location? or led or run or set or setting?)).tw,kf. 2247

55 ((nursery or nurseries or kindergarten* or preschool* or pre-school*) adj4 (clinic or clinics or health* or nurs* or program* or vaccin* or immuni#ation?)).tw,kf. 17683

56 ((nursery or nurseries or kindergarten* or preschool* or pre-school*) adj7 (accessibility or "access to" or uptake? or cover* or rate or rates)).tw,kf. 1551

57 (play school* or play group?).tw,kf. 179

58 School Health Services/ 18419

59 Schools/ and (exp vaccines/ or Vaccination/) 978

60 ((school? or highschool?) adj4 (based or delivered or located or location? or led or run or set or setting?)).tw,kf. 34598

61 (school? adj4 (clinic? or health care or healthcare or health unit? or nurs* or vaccin* or immuni#ation?)).tw,kf. 22557

62 (school? adj7 (accessibility or "access to" or uptake? or cover* or rate or rates)).tw,kf. 9776

63 ((universit* or colleg*) adj4 (based or delivered or located or location? or led or run or set or setting? or students)).tw,kf. 107150

64 ((universit* or colleg*) adj4 (clinic? or health care or healthcare or health unit? or nurs* or program* or vaccin* or immuni#ation?)).tw,kf. 46716

65 (library adj4 (based or delivered or located or location? or led or run or set or setting?)).tw,kf. 5011

66 (public library or (library adj4 (clinic? or health care or healthcare or health unit? or nurs* or program* or based or delivered or setting? or vaccin* or immuni#ation?))).tw,kf. 5710

67 ((leisure or recreation or sports) adj4 (centre? or center? or facility or facilities)).tw,kf. 1947

68 (sports hall? or gym or gyms or gymnasium?).tw,kf. 2812

69 (faith adj2 organi#ation?).tw,kf. 619

70 ((religion or religious) adj4 (building? or centre? or center? or facility or facilities or delivered or located or location? or led or run or set or setting?)).tw,kf. 705

71 ((faith or church* or mosque? or synagogue?) adj4 (clinic? or health care or healthcare or nurs* or program* or based or delivered or setting? or vaccin* or immunis* or immuniz*)).tw,kf. 3355

72 Transcultural Nursing/ 3466

73 "Religion and Medicine"/ 11672

74 home care services/ or home health nursing/ or home nursing/ 44595

75 House Calls/ 4224

76 (housecall? or house call? or outreach or out-reach or (door adj2 door) or ((home or homes) adj4 visit*) or (home adj4 (immuniz* or immunis* or vaccinat*))).tw,kf. 56508

77 adult day care centers/ or Senior Centers/ 255

78 Homes for the Aged/ or Housing for the Elderly/ 16383

79 assisted living facilities/ or group homes/ or exp nursing homes/ 47346

80 residential facilities/ 5761

81 (assisted living or ((residential or nursing) adj4 home*)).tw,kf. 46448

82 (care home? or rest home? or long* term care facilit*).tw,kf. 13322

83 orphanages/ or Foster Home Care/ 4366

84 (orphange? or foster care or foster home* or kinship care or children* home*).tw,kf. 4399

85 exp Education, Special/ 15644

86 (special adj4 (education or school*)).tw,kf. 7131

87 halfway houses/ 1071

88 exp Homeless Persons/ 11428

89 (homeless* or home less* or hostel? or ((halfway or half-way) adj (house? or home?))).tw,kf. 15871

90 Prisons/ 11486

91 (prison? or ((custody or correctional or detention* or reformato* or remand) adj4 (centre* or center* or facilit* or institut*))).tw,kf. 17275

92 Ambulatory Care Facilities/ 22899

93 Emergency Service, Hospital/ 89858

94 Outpatient Clinics, Hospital/ 15853

95 ((outpatient? or out-patient? or out-of-hospital) adj7 (intervention? or program* or project? or service? or study or trial)).tw,kf. 47402

96 ((outpatient? or out-patient?) adj4 (centre? or center? or clinic? or unit? or visit*)).tw,kf. 72397

97 (ambulatory care or ambulance? or paramed* or para-med* or paraprofessional? or para-professional? or emergency department? or (accident* adj2 emergenc*) or ED visit*).tw,kf. 173176

98 (ED immunis* or ED immuniz* or ED vaccinat*).tw,kf. 20

99 (case management or case manager? or case worker?).mp. 21135

100 (interpreter* or translator*).tw,kf. 6938

101 (social adj (support or work*)).mp. 140996

102 Vaccination/nu or vaccin* manager?.tw,kf. 174

103 Occupational Health/ 36885

104 ((work adj2 (based or delivered or place? or site or sites)) or workbased or workplace? or worksite?).tw,kf. 73718

105 ((vaccinat* or immuni#ation? or reimmuni#ation?) adj4 (occupational health or employee? or employer? or frontline or front-line or personnel or provider? or staff or worker? or work force or workforce or "at work")).tw,kf. 5886

106 (opportun* adj (vaccination? or immuni#ation?)).tw,kf. 60

107 (opportun* adj4 (catch-up or catchup or boost* or revaccinat* or reimmunis* or reimmuniz* or re-vaccinati* or re-immunis* or re-immuniz*)).tw,kf. 193

108 or/8-107 1767514

109 7 and 108 36055

[*Reminder Interventions*]

110 Reminder Systems/ 3818

111 ((remind* or recall* or messag* or alert*) adj4 system?).tw,kf. 6606

112 (reminder? or prompts or prompting or prompted or nudge?).tw,kf. 89967

113 ((immuni* or vaccin*) adj4 (recall* or remind*)).tw,kf. 1261

114 (recall* adj4 (alert* or appointment? or based or boost* or calendar or campaign* or central* or correspond* or initiative? or intervention? or invit* or letter? or mail or email or message? or notice? or phone? or postal or "by post" or program* or prompt* or regist* or remind*)).tw,kf. 4485

115 (remind* adj4 (alert* or appointment? or based or boost* or calendar or campaign* or central* or correspond* or initiative? or intervention? or invit* or letter? or mail or email or message? or notice? or phone? or postal or "by post" or program* or prompt* or regist* or recall*)).tw,kf. 5185

116 ((recall* or remind*) adj4 (app* or auto* or computer* or digital* or electronic or tele* or SMS or text*)).tw,kf. 7335

117 ((recall* or remind*) adj4 (adults or patients or individuals or parent* or carer? or mothers or fathers or family or families or men or women)).tw,kf. 9106

118 ((recall* or remind*) adj4 (employee? or employer? or frontline or front-line or personnel or provider? or staff or worker? or work force or workforce)).tw,kf. 1097

119 (remind* or recall*).ti. 13432

120 ((auto* or computer*) adj4 (alert* or messag*)).tw,kf. 2378

121 (autodial* or (auto* adj4 (call* or dial* or tele*))).tw,kf. 6505

122 correspondence as topic/ 2331

123 postal service/ 2443

124 letter/ 1248841

125 (letter? or correspondence).tw,kf. 160105

126 invitation?.tw,kf. 8068

127 ((immuni* or vaccin*) adj4 letter?).tw,kf. 301

128 (communication? and (vaccin* or Immuni#ation*)).ti,kf,hw. 2880

129 (mail* out? or circulars).tw,kf. 779

130 electronic mail/ or text messaging/ 7513

131 posters as topic/ or poster?.tw,kf. 12479

132 pamphlets/ or (pamphlet? or leaflet? or brochure?).tw,kf. 34588

133 (recei* adj4 (vaccin* or immuni#ation?) adj4 (alert* or information or messag*)).tw,kf. 307

134 decision support system?.mp. 16605

135 (decision adj4 making adj4 (auto* or system? or technique?)).tw,kf. 5671

136 ((activat* or auto* or check* or review or update or up-to-date) adj3 (health* or medical or patient?) adj3 record?).tw,kf. 18860

137 (immunosurveillance or immuno-surveillance).mp. 3949

138 (postcard* or post* card*).tw,kf. 12302

139 or/110-138 1611825

140 7 and 139 18015

141 (vaccin* adj4 (text? or tele* or recall* or remind* or prompt* or postcard* or post* card* or postal or nudg* or messag* or mail or email? or letter? or invit* or autodial* or auto* dial* or alert*)).tw,kf. 3013

142 Reminder Systems/ and (Primary Prevention/ or Preventive Health Services/) 118

143 140 or 141 or 142 19495

[*Educational Interventions*]

144 HEALTH PROMOTION/ or (public health adj (campaign? or message?)).mp. 84772

145 Health Education/ 64190

146 (health adj (education or promotion)).tw,kf. 86325

147 Knowledge/ or Patient Medication Knowledge/ 15327

148 Health Knowledge, Attitudes, Practice/ and (uptake? or coverage).mp. 6475

149 Patient Acceptance of Health Care/ and (uptake? or coverage).mp. 6262

150 Education/ 21535

151 Health Literacy/ 9858

152 Patient Education as Topic/ 88464

153 consumer health information/ 4336

154 National Health Programs/ 33710

155 Government Publications as Topic/ 698

156 Health Fairs/ or health fair?.tw,kf. 1026

157 (education* adj4 (campaign? or information* or intervention? or initiative? or message? or program* or promotion* or strateg* or tailor* or tool* or target* or study or trial)).tw,kf. 153771

158 curriculum/ or curricul*.tw,kf. 128862

159 (literacy or illiterat*).tw,kf. 37992

160 (campaign or education*).ti. 192646

161 ((client? or consumer? or patient? or public) adj4 (educat* or teach* or train*)).tw,kf. 120780

162 (educat* and inform*).ti,kf,hw. or "educat* and inform*".ab. 33558

163 (fact sheet? or factual information or informational).tw,kf. 13211

164 Information Seeking Behavior/ 3292

165 Information Dissemination/ 19559

166 persuasive communication/ 4078

167 ((tailor* or personali#ed or individuali#ed) adj2 (campaign? or education* or information* or intervention? or initiative? or message? or program* or promotion* or strateg* or study or trial)).tw,kf. 38225

168 motivational interviewing/ or motivational interview*.tw,kf. 6476

169 (psychoeducat* or psycho-educat*).tw,kf. 9413

170 message framing.tw,kf. 553

171 Sex Education/ 9397

172 Prenatal Education/ 362

173 Early Intervention, Educational/ 3585

174 ((women* or mother* or maternal* or prenatal* or pre-natal* or antenatal* or ante-natal* or postnatal* or post-natal* or postpartum* or post-partum*) adj4 (educat* or teach* or train*)).tw,kf. 42946

175 (child health services/ or maternal-child health services/) and pc.fs. 2152

176 (well adj (baby or infant or child) adj care).tw,kf. 824

177 INSERVICE TRAINING/ or staff development/ or communication skill?.tw,kf. 45198

178 Education, Professional/mt [Methods] 841

179 ((educat* or teach or teaching or train*) adj4 (inservice or in-service or personnel* or employe* or staff or worker* or pharmacist* or clinician* or doctor* or physician* or practitioner* or geriatrician* or p?ediatrician* or nurse* or nursing or midwife* or midwive* or health visitor* or health worker? or paramedic* or para-medic* or paraprofessional* or para-professional? or therapist* or counsellor* or counselor* or assistant* or technician* or teacher* or trainer* or leader* or volunteer* or lay or frontline or front line or patient facing)).tw,kf,hw. 286726

180 ((staff or professional) adj development).tw,kf. 16621

181 PUBLIC RELATIONS/ 7190

182 Community-Institutional Relations/ 11003

183 exp Interpersonal Relations/ 354531

184 (community adj4 (mobili#ation or outreach or relation*)).tw,kf. 10676

185 (outreach or out-reach).tw,kf. and educat*.mp. 7422

186 (rais* adj2 awareness adj4 (promotion* or campaign? or intervention* or tool* or strateg*)).tw,kf. 1559

187 Community Networks/ or social support/ or community support/ or psychosocial support systems/ 87495

188 ((communit* or social) adj4 (network* or support)).tw,kf. 109878

189 peer influence/ 821

190 ((advice or advise* or promot* or support* or advocat* or influence* or pressure* or recommend* or led) adj4 (peer* or family or families or friend* or professional* or clinician* or doctor* or physician* or practitioner* or geriatrician* or p?ediatrician* or nurse* or nursing or midwife* or midwive* or health visitor* or health worker* or paramedic* or para-medic* or paraprofessional* or para professional* or therapist* or counsellor* or counselor* or social worker* or leader* or community or communities or teacher* or faith or lay)).tw,kf,hw. 240083

191 Mentors/ 13502

192 (mentor* or role model* or counsel?or?).tw,kf. 42249

193 hotlines/ 2974

194 (champion* or hotline*).tw,kf. 12285

195 Self-Help Groups/ 9674

196 ((group* adj2 support*) or self-help*).tw,kf. 25286

197 *communication/ or (communication.mp. and (mt or pc or px).fs.) 157117

198 DECISION MAKING/ or Decision Making, Shared/ 107245

199 exp Informed Consent/ 44005

200 Choice Behavior/ 35165

201 Consumer Advocacy/ 3481

202 Decision Support Techniques/ 22632

203 (decision* adj2 (aid? or support or tool*)).tw,kf. 43786

204 (toolkit? or tool kit?).tw,kf. 13250

205 (informed adj4 (consent or choice* or decision*)).tw,kf. 69074

206 ((individual* or secondparty or second party or thirdparty or third party or parent* or guardian* or mother* or father* or family or families) adj4 consent*).tw,kf. 7267

207 ((behavio* adj3 chang*) and (campaign? or intervention or program* or strateg* or technique?)).tw,kf. 36276

208 ((tele* or phone*) adj4 (contact* or followup or follow up)).tw,kf. 14642

209 INFORMATION SYSTEMS/ 19540

210 Communications Media/ 1830

211 exp mass media/ 48240

212 Social Marketing/ or public service announcement?.mp. 2965

213 Advertising/ or advertising as topic/ or direct-to-consumer advertising/ 16198

214 advert*.tw,kf. 23473

215 (((print or written or digital) adj (media or material)) or broadside?).tw,kf. 3302

216 *Internet/ 39646

217 Internet-Based Intervention/ 1215

218 Social Media/ or (social media and (campaign or intervention or initiative or message* or promotion* or strateg* or study or trial)).tw,kf. 33412

219 Mobile Applications/ 12380

220 exp Cell Phone/ 23455

221 exp Computers, Handheld/ 13795

222 Medical Informatics Applications/ 2552

223 (radio or television* or tv or broadcast* or podcast* or newspaper* or magazine*).tw,kf. 96752

224 ((health or media) adj4 campaign?).tw,kf. 8819

225 (campaign? adj4 (advert* or banner* or flyer* or handout* or hand-out? or information* or intervention* or leaflet? or letter* or mail* or email or material or messag* or online or pamphlet? or presentation* or program* or promotion* or strateg* or video*)).tw,kf. 8048

226 (promotion* adj4 (advert* or banner* or campaign? or flyer* or handout* or hand-out? or information* or intervention* or leaflet? or letter* or mail* or email or material or messag* or pamphlet? or presentation* or program* or strateg* or video*)).tw,kf. 20912

227 ((universal or population based or national* or nationwide* or statewide* or countrywide* or citywide* or national* or nation wide* or state wide* or country wide* or city wide* or government*) adj4 (campaign or intervention)).tw,kf. 6201

228 (education* adj2 (advert* or banner? or brochure? or campaign? or comm* or flyer* or handout? or hand-out? or information* or intervention* or leaflet? or letter* or mail* or email or material or messag* or online or pamphlet? or poster* or presentation* or program* or promotion* or strateg* or tele* or text* or tool* or video* or www or web or website)).tw,kf. 122937

229 (phone* or telephone* or smartphone* or cellphone* or smartwatch*).ti. 28674

230 ((phone* or tele* or smartphone* or cellphone* or smartwatch) adj3 (based or app* or campaign? or information* or intervention* or messag* or program*)).ab. 40853

231 (mobile* adj3 (based or app* or intervention* or device* or technolog*)).tw,kf. 30541

232 exp video-audio media/ 42504

233 (webinar or webcast or web cast or webconferenc* or web conferenc* or videoconferenc* or video conferenc* or broadcast*).tw,kf. 12719

234 ((app or apps or online or web or website* or internet or digital*) not survey).ti. 157065

235 ((app or apps or online or web or www or website* or internet or digital*) adj3 (based or campaign? or information* or intervention* or messag* or presentation* or program* or tool*)).ab. 113477

236 (twitter or tweet* or blog* or vlog* or pinterest or instagram or facebook or snapchat or tiktok or whatsapp* or chatbot?).tw,kf. 22003

237 (mobile health or mhealth or m-health or ehealth or e-health).ti,kf. 20456

238 ((mobile health or mhealth or m-health or ehealth or e-health) adj3 (based or application* or campaign? or information* or intervention* or messag* or program* or tool*)).ab. 7314

239 Remote Consultation/ 5833

240 remote* consult*.tw,kf. 1248

241 (econsult* or e-consult* or teleconsult* or tele-consult*).tw,kf. 3202

242 (zoom or skype or facetime or face time or digital first or Attend Anywhere or ACCURX or SystmOne).tw,kf. 4888

243 ((complex or factorial or interdisciplinary or inter-disciplinary or multi* component? or multicomponent? or multidisciplin* or multi* disciplin* or multidimension* or multi* dimension* or multifactor* or multi* factor* or multifacet* or multi* facet* or multilevel* or multi* level* or multimodal* or multi* modal* or multiparamet* or multi* paramet* or multiecological or multi* ecological or multistrateg* or multi* strateg*) adj4 (campaign* or intervention? or program* or strateg* or study or system? or trial)).tw,kf. 142359

244 or/144-243 2579899

245 7 and 244 34656

246 ((vaccin* or vaccination? or immuni#ation?) adj4 (educat* or teach* or train*)).tw,kf. 4191

247 ((vaccine? or vaccination? or immuni#ation?) adj4 (communic* or messag* or dialogu* or conversation* or discussion* or negotiation*)).tw,kf. 3476

248 vaccin* information.tw,kf. 1092

249 ((vaccine? or vaccination? or immuni#ation?) adj4 (information* or informed)).tw,kf. and (acceptance or acceptability or attitude? or awareness or (behavi* adj2 chang*) or beliefs or choice? or compliance or consent* or intent* or knowledge or perception? or seeking or trust* or understanding or willingness or campaign or champion* or communication or educat* or encourage* or endorse* or influenc* or nudg* or persua* or promot*).mp. 4462

250 245 or 246 or 247 or 248 or 249 38935

[*Infrastructure Interventions*]

251 "Delivery of Health Care"/og 22540

252 organization.fx. and (immuni#ation? or vaccination?).ti,kf,hw. 4992

253 ((service* or system* or team* or practice* or provider*) adj4 (administ* or organis* or organiz* or coordin* or co ordin* or co-ordin* or logistic* or plan* or structur*)).tw,kf. 243533

254 "Appointments and Schedules"/ 9926

255 appointment*.tw,kf. 34895

256 ((immuni#ation? or vaccination? or revaccination? or reimmuni#ation?) adj4 (scheduling or book* or rebook*)).tw,kf. 169

257 (booking system? or digital registration?).tw,kf. 197

258 "treatment adherence and compliance"/ or patient compliance/ 61915

259 Motivation/ 82009

260 motivat*.ti,kf. or ((motivat* or encourage*) adj4 (vaccination* or immunis* or immuniz*)).ab. 34956

261 ((motivat* or encourage*) adj4 (consumer* or client* or patient* or participant* or individual* or parent* or guardian* or mother* or father* or family or families or adolescent* or teen* or youth* or young* or adult* or old* or elderly or male? or female* or men or women)).tw,kf. 43157

262 Reinforcement, Psychology/ or Reinforcement Schedule/ 24761

263 Reward/ or Token Economy/ 27330

264 Reimbursement, Incentive/ 4820

265 (incentive* or disincentive*).tw,kf. 39437

266 (reward* or token? or voucher?).tw,kf. 76470

267 ((immuni#ation? or vaccination? or revaccination? or reimmuni#ation?) adj4 (reimburs* or pay or payment* or paid)).tw,kf. 416

268 exp Public Assistance/ 88198

269 (social insurance or ((insurance* or social or socio* or tax or welfare) adj4 (allowance* or benefit* or assistance or support or subsidies or claim or claims))).tw,kf. 89730

270 (((cash or financ* or money or monetary or pay or payment* or paid) adj4 (allowance* or benefit* or assistance or support or subsidies or transfer* or claim or claims)) or prepaid or pre-paid or prepayment* or pre-payment*).tw,kf. 24150

271 (punish* or fines or fined or penal* or sanction* or deter or deterred or discourage*).tw,kf. 60360

272 "No Jab No Pay".tw,kf. 25

273 ((document* or proof or prov* or record*) adj4 (immuni#ation? or vaccination?) adj4 (status or up-to-date)).tw,kf. 456

274 ((block* or remov* or take away or withdraw? or with-draw? or withhold* or with-hold* or withheld* or with-held*) adj4 (allowance? or benefit? or ((cash or income or financial or monetary or education* or employment or housing or food) adj (assistance or support)) or social or socio* or subsidies or tax or welfare or (health adj (claim? or insurance?)))).tw,kf. 5008

275 ((block* or remov* or take away or withdraw? or with-draw? or withhold* or with-hold* or withheld* or with-held*) adj4 (childcare or child care or nursery or kindergarten or school?)).tw,kf. 412

276 Mandatory Programs/ 2908

277 ((immuni#ation? or vaccination? or revaccination? or reimmuni#ation?) adj4 (mandat* or compulsory or obligat*)).tw,kf. 2487

278 ((consumer* or client* or patient*) adj4 rights).tw,kf. 5062

279 Choice Behavior/ or Freedom/ 41285

280 or/251-279 880710

281 7 and 280 18540

[*RCT Filter*]

282 exp Randomized Controlled Trial/ 612277

283 Randomized Controlled Trial.pt. 610719

284 Random Allocation/ 107079

285 Controlled Clinical Trial.pt. 95511

286 (randomis* or randomiz*).tw,kf. 857925

287 (RCT or cRCT or "at random" or (random* adj3 (administ* or allocat* or assign* or class* or cluster or crossover or cross-over or control* or determine* or divide* or division or distribut* or expose* or fashion or number* or place* or pragmatic or quasi or recruit* or split or substitut* or treat*))).tw,kf. 765562

288 (intervention or trial).ti,kf. 469365

289 ((intervention? or control* or compar*) adj4 (group? or trial)).ab. 1294769

290 or/282-289 2403570

291 exp Animals/ not Humans/ 5211377

292 290 not 291 2090747

293 limit 292 to yr="2000 -Current" 1736334

294 109 and 293 2936

295 143 and 293 1318

296 250 and 293 3226

297 281 and 293 1262

298 294 or 295 or 296 or 297 5325

[*Countries/topics to remove*]

299 (low income countr* or Afghanistan or Burundi or Burkina Faso or Central African Republic or Eritrea or Ethiopia or Guinea or Gambia or Guinea-Bissau or Liberia or Madagascar or Mali or Mozambique or Malawi or Niger or North Korea or Korean Democratic Republic or Rwanda or Sudan or Sierra Leone or Somalia or South Sudan or Syria or Syrian Arab Republic or Chad or Togo or Uganda or Yemen or Zambia or Algeria or Angola or Bangladesh or Benin or Bhutan or Bolivia or Cote d?Ivoire or Ivory Coast or Cambodia or Cameroon or Congo or Comoros or Cabo Verde or Djibouti or Egypt or Eswatini or El Salvador or Ghana or Haiti or Honduras or India or Indonesia or Iran or Kenya or Kyrgyz Republic or Kiribati or Lao or Lebanon or Lesotho or Mauritania or Micronesia or Mongolia or Morocco or Myanmar or Nepal or Nicaragua or Nigeria or Pakistan or Papua New Guinea or Philippines or Samoa or (Sao Tome and Principe) or Senegal or Solomon Islands or Sri Lanka or Tajikistan or Tanzania or Timor-Leste or Tunisia or Ukraine or Uzbekistan or Vanuatu or Vietnam or (West Bank and Gaza) or Zimbabwe or (Africa and sub-sahara*)).ti. 434074

300 (immunogenic* or immun* genic* or seroepidemiolog* or seroprevalen* or serorespon* or (sero* adj1 (epidemiolog* or prevalen* or respon*))).ti. 36105

301 299 or 300 466821

302 298 not 301 4550

[*Types of Vaccine*]

303 Diphtheria-Tetanus Vaccine/ or Diphtheria-Tetanus-Pertussis Vaccine/ or Diphtheria-Tetanus-Acellular Pertussis Vaccines/ 4593

304 Pertussis Vaccine/ 5523

305 (((diphtheri* or diptheri* or antidiphtheri* or antidiptheri* or whooping cough or pertussis or antipertussis or tetanus or tetani* or antitetanus or antitetani*) adj vaccin*) or ((dpt or dtp or dtwp or di te per or dtap) adj vaccin*)).tw,kf. 7633

306 Measles Vaccine/ or Mumps Vaccine/ or Rubella Vaccine/ or Measles-Mumps-Rubella Vaccine/ 12387

307 ((measles or mumps or rubella or antimeasles or antimumps or antirubella or MMR) adj4 vaccin*).tw,kf. 12450

308 Influenza Vaccines/ 27512

309 (((flu or influenza or antiflu or antiinfluenza) adj4 vaccin*) or (LAIV and vaccin*)).tw,kf. 30114

310 Haemophilus Vaccines/ 3188

311 (((h?emophilus or antih?emophilus) adj4 vaccin*) or (Hib adj vaccin*)).tw,kf. 2511

312 Parainfluenza vaccines/ 57

313 ((parainfluenza or para-influenza) adj4 vaccin*).tw,kf. 216

314 Respiratory Syncytial Virus Vaccines/ 988

315 ((respiratory syncytial virus or rsv) adj4 vaccin*).tw,kf. 1871

316 *Streptococcal Vaccines/ or Pneumococcal Vaccines/ or Heptavalent Pneumococcal Conjugate Vaccine/ 9690

317 (((pneumoni* or pneumococ* or antipneumoni* or antipneumococ*) adj4 vaccin*) or ((PCV* or PPV*) and pneum* and vaccin*) or pneumovax).tw,kf. 13998

318 exp Viral Hepatitis Vaccines/ 13446

319 ((hep* or antihep*) adj2 vaccin*).tw,kf. 11963

320 Meningococcal Vaccines/ 3982

321 ((meningiti* or meningococ* or MenB* or antimeningiti* or antimeningococ* or antiMenB*) adj vaccin*).tw,kf. 2015

322 Poliovirus Vaccines/ or Poliovirus Vaccine, Inactivated/ or Poliovirus Vaccine, Oral/ 7980

323 (((polio* or antipolio*) adj vaccin*) or ((IPV or OPV) and polio* and vaccin*)).tw,kf. 6478

324 Tuberculosis Vaccines/ or BCG Vaccine/ 22665

325 (TB vaccin* or BCG vaccin* or calmette* vaccin* or (tubercul* adj4 vaccin*)).tw,kf. 14134

326 Rotavirus Vaccines/ 2906

327 ((rotavir* or rota-vir* or antirotavir* or antirota-vir*) adj4 vaccin*).tw,kf. 4564

328 Papillomavirus Vaccines/ or Human Papillomavirus Recombinant Vaccine Quadrivalent, Types 6, 11, 16, 18/ 10597

329 (((HPV* or human papillomavir* or antiHPV or antipapillomavir*) adj7 vaccin*) or ((cervical cancer or anticervical cancer) adj vaccin*) or (papillomavir* and cancer and vaccin*) or (Pap* and smear? and vaccin*)).tw,kf. 16412

330 Herpesvirus Vaccines/ or Chickenpox Vaccine/ or Herpes Zoster Vaccine/ 3795

331 (((chickenpox or chicken-pox or varicella or zoster or herpeszoster or shingles) adj4 vaccin*) or ((antichickenpox or antichicken-pox or antivaricella or antizoster or antiherpeszoster or antiherpes-zoster or antishingles) adj4 vaccin*) or ZVL vaccin* or RZV vaccin*).tw,kf. 4539

332 Smallpox Vaccine/ 4107

333 ((smallpox or small pox or variola or antismallpox or antismall-pox or antivariola) adj4 vaccin*).tw,kf. 4452

334 Covid-19 Vaccines/ or chadox1 ncov-19/ or ad26covs1/ or 2019-ncov vaccine mrna-1273/ or bnt162 vaccine/ 25981

335 (((coronavir* or COVID or COVID19 or COVID2019 or 2019-nCoV or 2019nCoV or nCoV-2019 or nCoV2019 or HCoV-19 or HCoV19 or SARS-CoV-2 or SARSCo-V2 or SARS-CoV2 or SARSCoV2 or SARSCoV-2 or SARS2 or severe acute respiratory syndrome) adj4 vaccin*) or mRNA vaccines).tw,kf. 45027

336 (Monkeypox/ or Monkeypox virus/) and Vaccines/ 19

337 ((monkey pox or monkeypox or antimonkey pox or antimonkeypox) adj4 vaccin*).tw,kf. 412

338 or/303-337 211463

339 (108 and 338 and 293) not 298 234

340 (139 and 338 and 293) not 298 106

341 (244 and 338 and 293) not 298 218

342 (280 and 338 and 293) not 298 71

343 339 or 340 or 341 or 342 497

344 343 not 301 433

345 302 or 344 4983

[*Date limited:* Sept-2023 to 8-Apr-2024 (initial searches from 2000 onwards)]

346 (202309* or 202310* or 202311* or 202312* or 2024*).ep. 787571

347 (2023 09* or 2023 10* or 2023 11* or 2023 12* or 2024*).dp. 711986

348 ("2023 Sep*" or "2023 Oct*" or "2023 Nov*" or "2023 Dec*" or "2024 Jan*" or "2024 Feb*" or "2024 Mar*" or "2024 Apr*").dp. 814982

349 ("2023/09*" or "2023/10*" or "2023/11*" or "2023/12*" or "2024*").ez,dt. 466889

350 346 or 347 or 348 or 349 1119567

351 345 and 350 412

352 2024*.yr. 575851

353 346 or 347 or 348 or 349 or 352 1119567

354 345 and 353 412

355 (2022* or 2023* or 2024*).yr,dp,dt,ep,ez. 3882600

356 345 and 355 1451

Key to date fields:

dp: date of publication

dt: create date

ep: electronic date of publication

ez: Entrez date

yr: year of publication

## **E: List of high-quality reviews used for data checking**

Where studies were included in high-quality reviews we used the information in the review to double check our data extractions. We classified the following Cochrane reviews and evidence reviews that informed the National Institute for Care Excellence (NICE) guidelines for vaccine uptake in the general population as being high-quality as both types of reviews followed rigorous methodologies.

NICE guidelines

- NICE guideline [NG218]: Vaccine uptake in the general population evidence reviews including Acceptability, Access, Education, Infrastructure, Reminders, and pregnant women.
- NICE guideline [NG103]: Flu vaccination: increasing uptake in children.

Cochrane reviews

- Jacobson Vann JC, Jacobson RM, Coyne-Beasley T, Asafu-Adjei JK, Szilagyi PG. Patient reminder and recall interventions to improve immunization rates. *Cochrane Database of Systematic Reviews* 2018, Issue 1.
- Kaufman J, Ryan R, Walsh L, Horey D, Leask J, Robinson P, Hill S. Face-to-face interventions for informing or educating parents about early childhood vaccination. *Cochrane Database of Systematic Reviews* 2018.
- Thomas RE, Lorenzetti DL. Interventions to increase influenza vaccination rates of those 60 years and older in the community. *Cochrane Database of Systematic Reviews* 2018, Issue 5.

## **F: Intervention and control categories used in this review**

| **Intervention** | **Definition used in this review to categorise interventions** |
| --- | --- |
| Access | Interventions aimed at improving access to vaccinations. Access interventions included those providing vaccines in different community locations (e.g. schools, pharmacies, specialist clinics, community venues, or at home), interventions where vaccines were available at extended times (e.g. at the weekend, in the evenings, or early morning), interventions that offered opportunistic vaccinations (e.g. during routine visits to primary care, at the emergency room, or at the pharmacy), walk-in vaccinations where an appointment was not required, interventions where recipients were given help to make appointments (e.g. staff making an appointment on the phone), online appointment booking systems, or where interventions offered vaccine series using an accelerated dosing schedule (e.g. for hepatitis B). |
| Education | Interventions that aim to increase awareness and understanding about the vaccine/s. In this review education will refer to interventions that provide information and/or education. This may include providing information about the recommended schedule, vaccine safety/side effects and the effectiveness of vaccines, information about the disease that the vaccine is aimed at - what are the perceived risks? what is the severity of the disease? Information about social responsibility regarding vaccination, peer support, and input. Information/education may be delivered in various different formats including through leaflets, face-to-face sessions, apps or websites, via phone calls or text messages. It may also be provided though the media of a decision aid, or via motivational interviewing. Education could be delivered by different healthcare professionals, community leaders, or peers. |
| Reminders | Interventions that aim to increase vaccine uptake via the provision of reminders. Reminders include both an initial invitation to be vaccinated when a routine vaccination becomes due, as well as any additional reminders/recall before the vaccination is due or once it becomes overdue. Reminders could come from primary care, community pharmacists, health or social workers, or from other healthcare professionals. Reminders could be delivered in various different forms including letters, phone calls, text messages, emails, in app notifications etc. |
| Affordability | Interventions that focused on increasing the affordability of vaccination. This included interventions that provide a financial incentive to get vaccinated: either directly, through a lottery system or via rewards schemes. Affordability also covered interventions that aim to cover costs by either providing free or reduced price vaccines, or by providing payments that cover financial or time costs involved in getting a vaccinations e.g. payment to cover transport costs. |
| Education and reminders | Any intervention that provides information or education about the vaccine (as above) and also has a reminder component (as above). |
| Multicomponent | Interventions with features from three or more intervention categories will be classed as multicomponent. Where interventions incorporate features of two of the other intervention categories listed here (e.g. access and education), we assessed which was the most prominent feature by both assessing which aspect of the intervention the author highlighted in the title or methods of the study report(s) and assessing the content of the interventions to determine if one of the features held more weight. Where a prominent feature could be established, we categorised the intervention as such. Where a prominent feature could not be identified with each feature appearing equally important, we categorised the intervention as multicomponent. |
| Control | Control interventions included usual care where participants in the control group continued to receive standard vaccination practices, No intervention, where participants in the control group received no intervention, and attention placebo where participants received an intervention similar in design to the active intervention group, but where the content is not vaccine-based e.g. general health education provided rather than vaccine education. |

## **G: Public involvement group**

We recruited a diverse group of twelve adults from under-served communities and facilitated engagement throughout the duration of the project. We held regular group meetings where we employed flexible approaches to ensure easy access for all members. This included offering remote or in-person attendance; hosting meetings in easy-to-reach community settings; and providing reimbursements for time and travel.

We held eight meetings where we updated the group on project progress and facilitated activities where important features of interventions to increase vaccine uptake were explored within small groups. Members reported liking the small group activity set-up as it was *‘engaging – [and] people felt comfortable to contribute’.* Through these activities the group played an integral role in helping us to understand perceived barriers and motivators to vaccinate. We also provided a number of training opportunities for the group and the contributors reported that there were reciprocal benefits, for instance one contributor stated that “*Before involvement I thought research was for people with masters and PhDs. I feel I have a lot to contribute. We all have a lot of discussions and talking about your experiences and community experiences about vaccination…you feel you can contribute a lot!”* (public contributor reflections, session six, July 2024)

We have published a paper describing the involvement of the public in this review, including details of the demographics of the public contributors: McGrath, C., Davies, S.R., Ali, I., Dick, B., Dewani, P. and French, C.E. (2025), Public Involvement in a Systematic Review Project: Reporting Our Approach Using the ACTIVE Framework. Health Expectations, 28: e70323. <http://dx.doi.org/10.1111/hex.70323>

## **H: Cluster adjustment**

### *Choice of ICC*

Most CRCTs did not report the relevant ICC required for cluster adjustment. To choose a value to use for these studies we collected all the ICCs reported across the trials. None of the studies that reported this information were randomised at the household level. For other trials, the ICC did not appear to depend on the unit of randomization. The median across all these values was 0.05. Based on these observations we chose to use ICC=0.05 for all CRCTs that did not report their own ICC and were not randomised at the household level.

Some studies assumed an ICC value in their sample size/power calculations. These values were usually based on external evidence. For studies that were not randomised by household, assumed ICCs ranged from 0.01 to 0.32 (median 0.1). This suggested that a sensitivity analysis using ICC=0.3 was sensible.  We also performed a sensitivity analysis with ICC=0 (i.e. no cluster adjustment). Only one study (Saaksvuori 2022) had information about power calculations for households, with an assumed ICC of 0.7. Therefore, we chose ICC=0.7 for CRCTs randomised at the household level with sensitivity analyses using the maximum and minimum possible values (ICC=1 and ICC=0).

### *Cluster size assumptions*

Seven CRCTs did not provide information about the size of clusters. Four of these studies (Szilagyi 2019, Dini 2000, Dombkowski 2017b, Arthur 2002) were randomised at the household level, two (Si 2022, Esposito 2018) were randomised by school class and one (Nyamathi 2009) did not report the unit of randomization. For studies randomised by household, we assumed a mean cluster size of 2 based on the typical number of children in a family. For school class we used the approximate mean class size in the country in which the trial was conducted. Based on data from the Organisation for Economic Co-operation and Development (OECD), we used a mean cluster size of 40 for China (trial Si 2022) and 20 for Italy (trial Esposito 2018). Where there was no information on the unit of randomization (Nyamathi 2009), we used the mean design effect across all other studies which was 13.02.

## **I: Statistical model**

We pre-specified a statistical analysis plan Davies, A. L., Caldwell, D. M., Higgins, J. P. T., Davies, S. R., & French, C. E. (2024). Analysis plan for a synthesis of interventions aimed at increasing vaccine uptake. Unpublished. <https://research-information.bris.ac.uk/en/publications/analysis-plan-for-a-synthesis-of-interventions-aimed-at-increasin> The plan was approved 29^th^ August 2024 by the project group.

We assumed a binomial likelihood for the number of events and modelled relative treatment effects as log odds ratios (LORs). For multi-arm trials, we specified a multivariate normal distribution for the relative treatment effects to account for correlations.

We consider a model of $N$ trials, $i=1,\ldots,N,$ and $M$ interventions, $T_{a}=T_{1},\ldots,T_{M}$. Each trial $i$ is associated with $A_{i}$ arms labelled $k=1,\ldots,A_{i}$. The intervention in arm $k$ of trial$i$ is labelled $t_{i,k}\in\{T_{a};a=1,\ldots,N\}$. We write $n_{i,k}$ for the number of participants (sample size) in this arm and $r_{i,k}$ for the number of events. We model the number of events as following a binomial distribution,

$$r_{i,k}\sim\text{Bin}\left( n_{i,k},p_{i,k} \right).$$

We define $\delta_{i,1k}$ as the trial-specific relative treatment effect between $t_{i,k}$ and $t_{i,1}$ in trial$i$ measured as a log odds ratio (LOR),

$$\mathrm{logit}\left( p_{i,k} \right)=\mu_{i}+\delta_{i,1k},$$

$$\mu_{i}=\mathrm{logit}\left( p_{i,1} \right).$$

As our primary analysis, we assume random effects on the relative treatment effects,

$$\boldsymbol{\delta}_{\boldsymbol{i}}\sim\mathcal{N}\left( \boldsymbol{\theta}_{\boldsymbol{i}},\boldsymbol{\Sigma}_{\boldsymbol{i}} \right),$$

$$\theta_{i,k}=d_{T_{1}t_{ik}}-d_{T_{1}t_{i1}},$$

where $d_{T_{1}T_{a}}$ is the basic parameter representing the average relative effect of intervention $T_{a}$ compared to the baseline intervention $T_{1}$. The vectors $\boldsymbol{\delta}_{\boldsymbol{i}}$ and $\boldsymbol{\theta}_{\boldsymbol{i}}$ have dimension $A_{i}-1$ where each element represents a relative effect between intervention $t_{i,k}$ and a trial specific reference arm $t_{i,1}$. We assume a common between trial heterogeneity variance, $\tau^{2}$, for all pairwise comparisons. Therefore, each between-study covariance matrix $\boldsymbol{\Sigma}_{\boldsymbol{i}}$ has dimensions $\left( A_{i}-1 \right)\times\left( A_{i}-1 \right)$ with diagonal entries equal to $\tau^{2}$ and off diagonal entries equal to $\tau^{2}/2$.

### *Subgroup analyses*

A categorical variable with $G$ categories can be represented by $G-1$ binary indicators $x_{1},\ldots,x_{G-1}$. To perform a subgroup analysis based on this variable, we place $G-1$ interaction terms on each of the basic parameters,

$$\theta_{i,k}=d_{T_{1}t_{ik}}-d_{T_{1}t_{i1}}+\sum_{j=1}^{G-1} {(\beta}_{j,T_{1}t_{ik}}-\beta_{j,T_{1}t_{i1}})x_{j}.$$

The rest of the model takes the same form as the primary model.

### *Model implementation*

We implemented our models in a Bayesian framework using JAGS ^1^. We used four chains and assessed convergence by inspecting MCMC trace plots and using the Brooks-Gelman-Rubin $\hat{R}$ statistic ^2,3^. We assigned non-informative prior distributions to all parameters. For heterogeneity, we specified a half-normal prior $\tau\sim HN\left( 0.25 \right)$. For the relative treatment effects (and subgroup interaction terms) we used $N\left( 0,{100}^{2} \right)$. We used an adaptive phase of 10,000 iterations, a burn in of an additional 10,000, and a further 10,000 iterations from which we drew our posterior samples.

## **J: Study characteristics of descriptive only studies**

| **Ref** | **Study** | **Country** | **COVID-19** | **Setting** | **Int** | **Design** | **N** | **Vaccine type** | **Age group** | **Findings (as reported by study authors)** | **Reason study is descriptive** |
| --- | --- | --- | --- | --- | --- | --- | --- | --- | --- | --- | --- |
| 147 | Alsan 2024 | USA | Pre- | Online | E | I | 2893 | Influenza | Adults | Although race-concordant expert senders and race-discordant expert senders acknowledging past medical injustice earned higher ratings from Black individuals, we find that signals on vaccination delivered by a race-concordant layperson led to the greatest increases in intent to be vaccinated against influenza and COVID-19 as well as take-up of the flu vaccine. The effects of nonexpert senders were concentrated among respondents with no prior experience with flu vaccination, a group that may be particularly difficult to persuade, whereas experts move vaccination intent most among those immunized in recent years. | Interventions not codable |
| 4 | Aref 2022 | USA | Pre- | Education | E | I | 133 | MenB | Adults | At three months follow up only three participants reported having obtained the MenB vaccine. Two of them reported that this study influenced them to get the vaccine. | Outcome data not in usable format |
| 16 | Berset 2023 | USA | Post- | Healthcare | R | I | 1312 | CV | Young children | 1 automated message plus personal contact attempts (group 3) (30.2%) and 2 automated messages plus personal contact attempts (group 4) (31.8%) had significantly higher rates of receiving the MMR vaccine compared to group 2 (2 automated messages: 3.5%) but not group 1 (1 automated message: 28.0%) | Interventions were coded as the same category |
| 29 | Cameron 2013 | USA | Pre- | Healthcare | E | I | 125 | Influenza | Adults | Uptake in groups was as follows: Fact only: 4 out of 31 participants; Fact and Myth: 1 out of 31 participants; fact, myth, and why: 3 out of 32 participants; and CDC facts and myths: 4 out of 31 participants. | Intervention categories are identical - 4 groups with differently framed messages |
| 37 | Clayton 2021 | USA | Pre- | Healthcare | ER | C | 815 | CV | Young children | There was no difference in vaccine uptake between the conditions. | Interventions were coded as the same category |
| 40 | Cox 2012 | USA | Pre- | Healthcare | ER | I | 1175 | HBV | Adults | Patients asked to form intentions were slightly more likely to accept the first vaccine dose (55.2%) than patients not asked intentions (52.1%) | Interventions were coded as the same category |
| 58 | Ernsting 2013 | Germany | Pre- | Community | E | I | 2033 | Influenza | Adults | The intervention generated an indirect effect via planning on vaccination. Compensatory health beliefs mediated between intention and behaviour. An interaction between intervention group and compensatory health beliefs on behaviour transpired. At low compensatory health belief levels, the intervention group resulted in more vaccinations than the standard group. | Outcome data not in usable format |
| 64 | Frew 2014 | USA | Pre- | Community | E | I | 126 | Influenza | Adults | Neither gain- [OR = 0.5176, (95% CI: 0.203,1.322)] nor loss-framed [OR = 0.5000, 95% CI: (0.192,1.304)] messages were significantly associated with increased likelihood of immunization during pregnancy. | Outcome data not in usable format |
| 66 | Galasso 2023 | Australia, Austria, France, Germany, Italy, New Zealand, Sweden, United Kingdom, United States | Post- | Online | E | I | 6379 | C-19 | Adults | The effects on vaccination rates are increases of 2.6 ppts for protecting others (95% CI −0.007, 0.058), 3.8 ppts for protecting health (95% CI 0.004, 0.071) and 2.9 ppts for protecting the economy (95% CI −0.004, 0.063), corresponding to increases relative to the mean in the control group of 3.9%, 5.7% and 4.3%, respectively | too many countries that may have too many confounding factors. |
| 68 | Gerend 2012 | USA | Pre- | Education | E | I | 739 | HPV | Adults | The message framing interventions did not have a direct effect on HPV vaccine uptake. | Intervention categories are identical - differently framed messages |
| 74 | Green 2020 | USA | Post- | Community | A | I | 2727 | CV | Young children | Children who were enrolled for longer had fewer gaps in health insurance coverage and received more well-baby visits and immunizations compared to those with less service. | Outcome data not in usable format |
| 75 | Gurfinkel 2021 | USA | Pre- | Healthcare | R | I | 32283 | HPV | Adolescents |  |  |
| 89 | Hu 2021 | Singapore | Post- | Healthcare | E | I | 320 | Influenza | Older adults | At three months, 16 (10%) patients in the intervention group and 20 (12.5%) patients in the control group had completed influenza vaccination (P = 0.48). | Interventions were coded as the same category |
| 98 | Hutchinson 2018 | USA | Pre- | Healthcare | E | I | 235 | Influenza | Young children, Adolescents | 0% of the control participants received the vaccine in clinic vs 39% in the interventions group. | This study was unusual in that the control group had no chance to get a vaccine and so it was an outlier in comparison to other control arms. |
| 103 | Jacobson 2022 | USA | Post- | Healthcare | Aff | I | 58,308 | C-19 | Adults | While messages increased vaccination intentions, none of the interventions increased vaccination rates. Estimates for financial incentives rule out even relatively small increases in vaccination rates. | Outcome data is not in a usable format |
| 112 | Juraskova 2011 | Australia | Pre- | Education | E | I | 157 | HPV | Adolescents | Receipt of the vaccine at the 2-month follow-up was 37%, and was also not influenced by information framing. | Interventions were coded as the same category |
| 113 | Kasting 2019 | USA | Pre- | Healthcare | E | I | 1751 | HBV | Adults | 14.1% received all 3 recommended doses. There was not a significant interaction between message framing and HCP recommendation (p =.59). | Interventions were coded as the same category |
| 121 | Kim 2020 | USA | Pre- | Online | E | I | 104 | HPV | Adults | At the 2-month follow-up, the experimental group was twice as likely to receive the HPV vaccine compared to the comparison group. | Interventions were coded as the same category |
| 124 | Larsen 2023 | USA | Post- | Online | E | C | N/A | C-19 | Adults | The results are statistically indistinguishable across the two groups, demonstrating that the results are not sensitive to how we control for the growth rate of vaccinations in different sized counties. In column 1, we observe an increase of 10 vaccines, with a standard error of 78.74, implying a P value from a one-tailed test of 0.097. Thus, at a 95% confidence level, we cannot reject a null effect. At a 90% confidence level, we do reject zero, although we still cannot rule out small effects (as low as 1.81 vaccines per county). | Outcome data not in usable format |
| 136 | Marra 2014 | Canada | Pre- | Healthcare | A | C | 14,815 | Influenza | Older adults | In 2009, the mean rate of immunization in the 14 intervention communities was 83.8% (SD 16.3) compared with 85.6% (SD 16.6) in the 10 control communities, p = 0.79 (difference of 1.8%, 95% CI –12.3% to 15.9%). | Outcome data not in usable format |
| 136 | Marra 2014 | Canada | Pre- | Healthcare | A | C | 14,815 | Influenza | Older adults | In 2010, the mean influenza immunization rate was 56.9% (SD 28.0) in the control communities (n = 15) and 80.1% (SD 18.4) in the intervention communities (n = 14) (p = 0.01) for those ≥65 years of age. | Outcome data not in usable format |
| 141 | Mehta 2022 | USA | Post- | Healthcare | R | I | 16045 | C-19 | Adults | Among the 15 655 patients receiving text messaging, 118 of 3889 patients (3.0% [95% CI, 2.5%-3.6%]) in the standard messaging group completed 1 vaccine dose, as did 135 of 3920 patients (3.4% [95% CI, 2.9%-4.0%]) in the clinician endorsement group (absolute difference, 0.4% [95% CI, −0.4% to 1.2%]; P = .31), 100 of 3911 patients (2.6% [95% CI,2.1%-3.1%]) in the scarcity group (absolute difference, −0.5% [95% CI, −1.2% to 0.3%]; P = .20), and 143 of 3935 patients (3.6% [95% CI, 3.0%-4.2%]) in the endowment group (absolute difference, 0.6% [95% CI, −0.2% to 1.4%]; P = .14). | Interventions were coded as the same category |
| 143 | Milkman 2022 | USA | Post- | Community | Aff | I | 587,508 | C-19 | Adults, Older Adults | The first treated zip code, which drew considerable media attention, may have experienced a small bump in vaccinations compared with the control zip codes: average weekly vaccinations rose by an estimated 61 per 100,000 people per week (+11%). After pooling the results from all three zip codes treated during our six-week experiment, however, we do not detect evidence of any overall benefits. Furthermore, our 95% confidence interval provides a 9% upper bound on the net benefits of treatment in our study. | intervention not codable - financial incentives with different chances of winning. |
| 144 | Milkman 2022 | USA | Post- | Healthcare | R | I | 689,693 | Influenza | Adults | We found that the reminder texts that we tested increased pharmacy vaccination rates by an average of 2.0 percentage points, or 6.8%, over a 3-mo follow-up period. The most effective messages reminded patients that a flu shot was waiting for them and delivered reminders on multiple days. The top performing intervention included two texts delivered 3 days apart and communicated to patients that a vaccine was “waiting for you.” | Intervention categories are identical – different reminders |
| 149 | NCT 2022 | France, USA | Post- | Online | E | I | N/A | C-19 | Adults | We can reject very small effects of any intervention on new first doses (0.16 pp, United States; 0.021 pp, France), with similar results for second doses and boosters (United States). | Outcome data not in usable format |
| 155 | Nyamathi 2009 | USA | Pre- | Community/Other | M | C | 865 | HAV,HBV | Adults | Sixty-eight percent of the NCMIT participants completed the three-series vaccine at 6 months, compared with 61% of SIT participants and 54% of SI participants | Interventions were coded as the same category |
| 156 | Nyamathi 2010 | USA | Pre- | Healthcare | ER | I | 256 | HAV,HBV | Adults | A total of 148 participants completed the vaccine. Groups did not differ in rate of vaccination completion (73.6%, HHP group, versus 65% and 69% for the MI-Single, and MI-Group, respectively). | Interventions were coded as the same category |
| 165 | Patel 2012 | USA | Pre- | Healthcare | ER | I | 256 | HPV | Adolescents, Adults | HPV vaccine uptake was low (5.5%) and did not differ by study group. | Interventions not codable - multiple messages with slight differences in framing. |
| 167 | Payakachat 2016 | USA | Pre- | Healthcare | E | I | 291 | CV | Pregnant women | There was no significant difference in vaccination rates between the sVIS and mVIS groups (45% vs. 49%). | Interventions were indistinguishable |
| 172 | Rabb 2022 | USA | Pre- | Online | ER | I | 14248 | C-19 | Adults | No evidence of differences in vaccination rates (however measured) between the control and an aggregated ‘any message’ condition (estimated difference in proportions vaccinated −0.001, 95% confidence interval (CI) −0.004 to 0.001, CMH test, P = 0.27), nor between the arms taken all together (CMH test for 9 × 2 × 13 table, P = 0.12). | Outcome data not in usable format |
| 179 | Rickert 2015 | USA | Pre- | Education | E | I | 445 | HPV | Adolescents | The rhetorical question component of the intervention increased intention to vaccinate (RR = 1.45; 95% CI1.16,1.81), but not first dose uptake or series completion. The 1-sided and 2-sided messages had no effect. | Intervention arms couldn’t be coded. |
| 183 | Ronzani 2022 | Italy | Post- | Online | ER | I | 2277 | C-19 | Adults | There was no difference in vaccine uptake between the messages that included expert endorsement and those that did not. | Interventions were coded as the same category |
| 186 | Saitoh 2017 | Japan | Pre- | Healthcare | E | C | 188 | CV | Young children | The overall completion rates for all five vaccines did not differ significantly between the intervention (43.0%) and control groups (45.5%) at the 6-month follow-up (p = 0.77) | Interventions were coded as the same category |
| 189 | Schwarz 2008 | USA | Pre- | Community | E | C | 104 | HBV | Young children, Adolescents | Of the 104 children and adolescents who needed HBV vaccine at the beginning of the study, 62.3% (33/53) of the HBV video group completed the HBV vaccine series by the end of the study vs. 45.1% (23/51) of the Smoking video group, p=0.16. | This study didn't fit into any of our age/vaccine categories. |
| 196 | Skinner 2000 | Australia | Pre- | Education | E | C | 17411 | HBV | Adolescents | The difference in mean school uptake between intervention and control was small at 12% per dose. | Outcome data not in usable format |
| 197 | Song 2000 | Korea | Pre- | Healthcare | R | I | 2017 | Influenza | Older adults | The vaccination rate was 46.7% in the control group, 56.3% in the postcard reminder group, and 63.3% in the telephone reminder group. | Not enough data to use - paper in Korean, data extracted only from the abstract. |
| 199 | Stockwell 2012 | USA | Pre- | Healthcare | R | I | 174 | CV | Young children | Significantly more adolescents with intervention parents received either or both MCV4 and Tdap at weeks 4 (15.4% vs 4.2%; P<.001). | Interventions were coded as the same category |
| 212 | Szilagyi 2022 | USA | Pre- | Healthcare | R | I | 196,486 | Influenza | Older adults | Influenza vaccination rates were as follows: for young adults 36.8%, for older adults 55.6%. On unadjusted and adjusted (for age, gender, insurance, race, ethnicity, and prior influenza vaccine history) analyses, influenza vaccination rates were not statistically different for any study group versus control. | Outcome data not usable |
| 214 | Szilagyi 2023 | USA | Post- | Healthcare | R | I | 213,773 | Influenza | Young Children, Adolescents, Adults, Older Adults | Influenza vaccination rates overall were low (39.0%). Vaccination rates for study arms did not differ: Control (38.9%), commitment vs no commitment (39.2%/38.9%), direct appointment scheduling yes/no (39.1%/39.1%), appointment reminders yes/no (39.1%/39.1%); p > 0.017 for all comparisons (p value cut-off adjusted for multiple comparisons). | Outcome data not in usable format |
| 228 | Underwood 2015 | USA | Pre- | Education | E | C | 686 | HPV | Adolescents | Parental attitude score was the strongest predictor of HPV vaccine initiation among adolescents (adjusted odds ratio (aOR): 2.08; 95% confidence interval (CI): 1.80, 2.39). | Outcome data not in usable format |
| 232 | Wagner 2021 | USA | Pre- | Healthcare | R | I | 250 | CV | Young Children | There were no significant differences in vaccination uptake between those enrolled in UC or the VFB intervention (0.51%, p=0.86). | Outcome data not usable |
| 237 | Wilcox 2001 | USA | Pre- | Community | R | I | 1752 | CV | Young Children | Children in the outreach group were more likely to receive an immunization during the study observation period than children in the control group (61% vs 43%; P<.001). | Outcome data not usable – days unvaccinated. |
| 241 | Wootton 2018 | USA | Pre- | Healthcare | R | I | 280 | Influenza | Adults | Vaccination rates with opting-in and opting out were similar among all (83 vs. 84%), UTHealth (87 vs. 93%), and UTMB patients (79 vs.76%) (p > 0.05). | intervention not codable using our categories. |
| 249 | Yue 2020 | Singapore | Pre- | Community | Aff | I | 3000 | Influenza | Older adults | Increasing the total incentive for vaccination from 10 to 20 Singapore dollars (SGD) increased participation in vaccination from 4.5% to 7.5% (P < .001). Increasing the total incentive from 20 to 30 SGD increased the participation rate to 9.2%, but this was not statistically significantly different from a 20-SGD incentive. | Intervention categories are identical - different levels of financial incentive. |
| 251 | Zhang 2018a | USA | Pre- | Community/Other | E | I | 451 | HAV,HBV | Adults | 85% of eligible participants in both groups completed the three-dose series during the intervention periods. Neither statistically significant nor substantive differences were found in the breakdowns of either the number of participants or vaccines received between the two groups. | Interventions were coded as the same category |

**Footnotes**

Key: C: Cluster RCT, I: Individually randomised RCT,

Pre-: Pre- COVID-19, Post-: After-COVID-19

CV: Childhood vaccines, C-19: COVID-19, Flu: Influemza, HPV: Human papilloma vaccine, HBV: HBV vaccine,

Con: Control, Ac: Access, Af: Affordability, E: Education, ER: Education and reminder, R: Reminder, M: Multicomponent.

## **K: Study characteristics of studies included in analyses**

| **Ref No.** | **Study** | **Design** | **Country*** | **N** | **No Clusters** | **Int 1** | **Int 2** | **Int 3** | **Int 4** | **Outcome** | **Age** | **Vaccine** | **COVID-19** | **Setting** | **% Male** | **% Female** | **% White** | **Control proportion vaccinated** |
| --- | --- | --- | --- | --- | --- | --- | --- | --- | --- | --- | --- | --- | --- | --- | --- | --- | --- | --- |
| 1 | Abroms 2023 | RCT | United States | 719 | NA | Con | E |  |  | A | Adults | COVID-19 | Post- | Online | 25.7 | 74.3 | 79.9 | 0.05 |
| 2 | Alonge 2023 | RCT | United States | 4296 | NA | Con | R |  |  | A | Adolescents | COVID-19 | Post- | Healthcare | 50.7 | 49.3 | 77.7 | 0.08 |
| 3 | Anraad 2023 | RCT | Netherlands | 1236 | NA | Con | E |  |  | A | Pregnant women | CV | Post- | Online | 0.0 | 100.0 | NA | 0.92 |
| 5 | Arnold 2022 | RCT | Germany | 506 | NA | Con | R |  |  | A | Adults | BCG Tuberculosis, CV, Hepatitis A, Hepatitis B, Influenza | Pre- | Community/Other | 92.7 | 7.3 | NA | 0.31 |
| 6 | Arthur 2002 | CRCT | United Kingdom | 2052 | NA | Ac | ER |  |  | A | Older adults | Influenza | Pre- | Community/Other | 39.2 | 60.8 | NA | NA |
| 7 | Bartos 2022 | RCT | Czech Republic | 2101 | NA | Con | E |  |  | A | Adults | COVID-19 | Post- | Online | 49.9 | 50.1 | 100 | 0.77 |
| 8 | Bartu 2006 | RCT | Australia | 152 | NA | Con | E |  |  | A | Young children | CV | Pre- | Community/Other | 0.0 | 100.0 | 88.8 | 0.20 |
| 9 | Baskin 2018 | RCT | United States | 30748 | NA | R | Aff |  |  | A | Adults | Influenza | Pre- | Education | 50.0 | 50.0 | NA | NA |
| 10 | Bastani 2022 | RCT | United States | 238 | NA | M | E |  |  | S | Adolescents | HPV | Pre- | Healthcare | 7.1 | 92.9 | 2.1 | NA |
| 11 | Bennett 2015 | RCT | United States | 661 | NA | Con | E |  |  | S | Adults | HPV | Pre- | Education | 0.0 | 100.0 | 67.3 | 0.01 |
| 13 | Berg 2004 | CRCT | United States | 181590 | 181590 | Con | E |  |  | A | Adults | Influenza | Pre- | Healthcare | 46.1 | 53.9 | NA | 0.16 |
| 12 | Berg 2008 | CRCT | United States | NA | 134791 | Con | E | E |  | A | Older adults | Influenza | Pre- | Healthcare | 50.0 | 50.0 | NA | 0.21 |
| 14 | Berkhout 2018 | CRCT | France | 10597 | 75 | Con | E |  |  | A | Older adults | Influenza | Pre- | Healthcare | 42.0 | 58.0 | NA | 0.49 |
| 15 | Bernard-Genest 2021 | RCT | Canada | 130 | NA | Con | ER |  |  | A | Adults | HPV | Post- | Healthcare | 0.0 | 100.0 | NA | 0.12 |
| 16 | Berset 2022 | RCT | United States | 945 | NA | Con | R | R |  | A | Children | COVID-19 | Post- | Healthcare | 47.8 | 52.2 | 23.9 | 0.04 |
| 18 | Bethke 2024 | CRCT | Germany | 6512 | 25 | E | Ac |  |  | A | Young children | CV | Pre- | Education | 54.3 | 45.7 | NA | NA |
| 19 | Bian 2023 | CRCT | China* | 375 | 202 | Con | ER |  |  | A | Older adults | COVID-19 | Post- | Education | 46.7 | 53.3 | NA | 0.17 |
| 21 | Borg 2018 | CRCT | Australia | 6619 | 5534 | Con | ER | ER |  | A | Children | Influenza | Pre- | Healthcare | 51.4 | 48.6 | NA | 0.04 |
| 22 | Bourgeois 2008 | CRCT | United States | 144 | 8 | Con | ER |  |  | A | Adults | Influenza | Pre- | Community/Other | 51.2 | 48.8 | NA | 0.19 |
| 23 | Bowman, 2014 | RCT | United States | 460 | NA | Con | Ac |  |  | S | Adults | HBV | Pre- | Community/Other | 50.0 | 50.0 | NA | 0.68 |
| 24 | Brigham 2012 | RCT | United States | 424 | NA | Con | ER | ER |  | A | Adolescents | CV, MenACWY | Pre- | Healthcare | 45.0 | 55.0 | 12.5 | 0.07 |
| 25 | Bronchetti 2015 | RCT | United States | 9358 | NA | M | Aff | M | ER | A | Adults | Influenza | Pre- | Education | 42.7 | 57.3 | NA | NA |
| 27 | Burkhardt 2023 | RCT | United States | 1235 | NA | Con | R |  |  | A | Adolescents | COVID-19 | Post- | Healthcare | 51.6 | 48.4 | 15.7 | 0.02 |
| 28 | Buttenheim 2022 | RCT | United States | 11188 | NA | Con | R | R |  | A | Adults | Influenza | Post- | Healthcare | 44.6 | 55.4 | 69.2 | 0.31 |
| 30 | Campos-Mercade 2021 | RCT | Sweden | 9560 | NA | Con | Aff |  |  | A | Adults | COVID-19 | Post- | Online | 50.0 | 50.0 | NA | 0.71 |
| 31 | Cataldi 2024 | CRCT | United States | 49403 | 8 | Con | E |  |  | S | Adolescents | HPV | Post- | Community/Other | 51.1 | 48.9 | 67.1 | 0.41 |
| 32 | Centers for Disease 2012 | RCT | United States | 878 | NA | Con | R |  |  | S | Young children | CV | Pre- | Healthcare | 52.8 | 47.2 | NA | 0.28 |
| 33 | Chai 2013 | RCT | China* | 1992 | NA | Con | ER |  |  | A | Adults | Influenza | Pre- | Healthcare | 48.4 | 51.6 | NA | 0.18 |
| 34 | Chang 2023 | RCT | United States | 57893 | NA | Con | R | M |  | A | Adults | COVID-19 | Post- | Healthcare | 42.3 | 57.7 | 20.7 | 0.03 |
| 35 | Chao 2015 | RCT | United States | 12255 | NA | Con | R |  |  | S | Adolescents | HPV | Pre- | Healthcare | 0.0 | 100.0 | 23.9 | 0.47 |
| 36 | Chodick 2021 | RCT | Israel | 21592 | NA | Con | E |  |  | A | Adolescents | HPV | Pre- | Online | 0.0 | 100.0 | NA | 0.55 |
| 37 | Clayton 2021 | CRCT | United States | 678 | 678 | Con | ER | ER | ER | S | Young children | CV | Pre- | Online | 13.6 | 86.4 | 97.2 | 0.80 |
| 38 | Coley 2018 | RCT | United States | 303965 | NA | Con | ER |  |  | S | Adolescents | HPV | Pre- | Healthcare | 50.0 | 50.0 | NA | 0.00 |
| 39 | Conner 2017 | RCT | United Kingdom | 13806 | NA | Con | R |  |  | A | Older adults | Influenza | Pre- | Healthcare | 43.7 | 56.3 | 96.8 | 0.75 |
| 41 | Cutrona 2018 | RCT | United States | 30000 | NA | Con | R | ER | E | A | Adults | Influenza | Pre- | Healthcare | 41.3 | 58.7 | 77.9 | 0.10 |
| 42 | Dai 2021a | RCT | United States | 93354 | NA | Con | R | ER |  | A | Adults | COVID-19 | Post- | Healthcare | 43.3 | 56.7 | NA | 0.14 |
| 42 | Dai 2021b | RCT | United States | 67092 | NA | Con | R | R |  | A | Adults | COVID-19 | Post- | Healthcare | 43.5 | 56.5 | NA | 0.06 |
| 43 | Dalby 2000 | RCT | Canada | 142 | NA | Con | Ac |  |  | A | Older adults | Influenza | Pre- | Community/Other | 33.1 | 66.9 | NA | 0.54 |
| 45 | Daley 2002 | RCT | United States | 1234 | NA | Con | ER |  |  | A | Young children | CV | Pre- | Healthcare | 52.9 | 47.1 | NA | 0.20 |
| 44 | Daley 2014 | CRCT | United States | NA | 16 | Con | Ac |  |  | A | Adolescents | CV | Pre- | Education | 50.0 | 50.0 | NA | 0.26 |
| 148 | Daly, 2023 | RCT | Georgia* | 55176 | NA | Con | R | R |  | A | Adolescents | HPV | Post- | Healthcare | 0.0 | 100.0 | NA | 0.02 |
| 46 | Daniels 2007 | RCT | United States | 186 | NA | M | ER |  |  | A | Older adults | Influenza | Pre- | Community/Other | 25.3 | 74.7 | 8 | NA |
| 47 | Dapp 2011 | RCT | Germany | 2580 | NA | Con | E |  |  | A | Older adults | Influenza | Pre- | Healthcare | 37.3 | 62.7 | 100 | 0.57 |
| 48 | Davies 2017 | CRCT | Australia | 6967 | 40 | Con | E |  |  | S | Adolescents | HPV | Pre- | Education | 54.6 | 45.4 | NA | 0.79 |
| 49 | DeCamp 2020 | RCT | United States | 157 | NA | Con | ER |  |  | A | Children | Influenza | Pre- | Healthcare | 0.0 | 100.0 | NA | 0.79 |
| 50 | Dempsey 2019 | RCT | United States | 1294 | NA | Con | E | E |  | S | Adolescents | HPV | Pre- | Healthcare | 34.5 | 65.5 | 8.5 | 0.09 |
| 51 | DiClemente 2015 | RCT | United States | 216 | NA | Con | ER |  |  | S | Adolescents | HPV | Pre- | Healthcare | 50.0 | 50.0 | NA | 0.02 |
| 52 | Dini 2000 | CRCT | United States | 1227 | NA | Con | R | R | R | U | Young children | CV | Pre- | Healthcare | 50.0 | 50.0 | NA | 0.41 |
| 53 | Dombkowski 2014 | RCT | United States | 10175 | NA | Con | R |  |  | S | Young children | CV | Pre- | Healthcare | 50.0 | 50.0 | NA | 0.17 |
| 54 | Dombkowski 2017a | RCT | United States | 2348 | NA | Con | R |  |  | A | Adolescents | Influenza | Pre- | Healthcare | 49.4 | 50.6 | NA | 0.29 |
| 54 | Dombkowski 2017b | CRCT | United States | 2048 | NA | Con | R |  |  | A | Adolescents | Influenza | Pre- | Healthcare | 48.9 | 51.1 | NA | 0.37 |
| 55 | Domek 2019 | RCT | Guatemala* | 720 | NA | Con | R |  |  | F | Young children | CV | Pre- | Healthcare | 0.0 | 100.0 | NA | 0.92 |
| 56 | Doyle 2015 | RCT | Ireland | 233 | NA | Con | E |  |  | S | Young children | CV | Pre- | Community/Other | 0.0 | 100.0 | 99 | 0.73 |
| 57 | El-Mohandes, 2003 | RCT | United States | 286 | NA | Con | E |  |  | S | Young children | CV | Pre- | Community/Other | 0.0 | 100.0 | NA | 0.35 |
| 59 | Esposito 2018 | CRCT | Italy | 917 | NA | Con | E | E |  | A | Adolescents | HPV | Pre- | Education | 42.4 | 57.6 | NA | 0.09 |
| 60 | Fernandez 2022 | RCT | United States | 1554 | NA | Con | E |  |  | A | Adolescents | HPV | Pre- | Community/Other | 6.5 | 93.5 | 7.7 | 0.30 |
| 61 | Ferreira 2022 | CRCT | Brazil* | 238 | 6 | Con | E |  |  | A | Adolescents | HPV | Post- | Education | 0.0 | 100.0 | NA | 0.24 |
| 62 | Fiks 2013 | RCT | United States | 11434 | NA | Con | ER |  |  | S | Adolescents | HPV | Pre- | Healthcare | 0.0 | 100.0 | 55.7 | 0.65 |
| 63 | Fitzpatrick 2018 | RCT | China* | 556 | NA | Con | E |  |  | A | Adults | Hepatitis B | Pre- | Online | 100.0 | 0.0 | NA | 0.08 |
| 64 | Frew 2016 | RCT | United States | 106 | NA | Con | E | E |  | A | Pregnant women | Influenza | Pre- | Healthcare | 50.0 | 50.0 | NA | 0.12 |
| 67 | Gerend 2021 | RCT | United States | 150 | NA | Con | ER |  |  | F | Adults | HPV | Pre- | Healthcare | 100.0 | 0.0 | NA | 0.07 |
| 69 | Glanz 2017 | RCT | United States | 1093 | NA | Con | E | E |  | U | Young children | CV | Pre- | Online | 0.0 | 100.0 | 86.9 | 0.87 |
| 70 | Glanz 2020 | RCT | United States | 824 | NA | Con | E | E |  | U | Young children | CV | Pre- | Online | 0.4 | 99.6 | 85.3 | 0.92 |
| 71 | Goodman 2015 | RCT | United States | 105 | NA | Con | E |  |  | A | Pregnant women | Influenza | Pre- | Healthcare | 0.0 | 100.0 | 0.8 | 0.25 |
| 72 | Goodyear-Smith 2012 | CRCT | New Zealand | 5256 | 63 | Con | ER |  |  | F | Young children | CV | Pre- | Healthcare | 50.0 | 50.0 | NA | 0.99 |
| 73 | Grandahl 2016 | CRCT | Sweden | 751 | 18 | Con | E |  |  | A | Adolescents | HPV | Pre- | Education | 48.0 | 52.0 | NA | 0.61 |
| 75 | Gurfinkel 2021 | RCT | United States | 37003 | NA | Con | R | R |  | S | Adolescents | HPV | Pre- | Healthcare | 52.1 | 47.9 | NA | 0.17 |
| 76 | Haff 2023 | RCT | United States | 3671 | NA | Con | Ac | Ac |  | A | Adults | COVID-19 | Post- | Healthcare | 41.0 | 59.0 | 60.1 | 0.14 |
| 77 | Hambidge 2009 | RCT | United States | 811 | NA | Con | ER |  |  | U | Young children | CV | Pre- | Healthcare | 0.0 | 100.0 | NA | 0.33 |
| 78 | Hanley 2023 | RCT | United States | 7408 | NA | Con | E |  |  | S | Adolescents | HPV | Post- | Healthcare | 45.2 | 54.8 | 24.4 | 0.06 |
| 79 | Hannan 2013 | RCT | United States | 139 | NA | Con | E |  |  | A | Young children | CV | Pre- | Healthcare | 0.0 | 100.0 | NA | 0.84 |
| 80 | Harari 2008 | CRCT | United Kingdom | 2503 | 2050 | Con | E |  |  | A | Older adults | Influenza | Pre- | Healthcare | 45.1 | 54.9 | NA | 0.86 |
| 81 | Henrikson 2018 | RCT | United States | 1805 | NA | Con | ER |  |  | A | Adolescents | HPV | Pre- | Healthcare | 51.7 | 48.3 | 58.1 | 0.46 |
| 82 | Hess 2013 | CRCT | United States | 11982 | 16 | Con | R |  |  | A | Older adults | Shingles | Pre- | Healthcare | 50.0 | 50.0 | NA | 0.01 |
| 83 | Higginbotham 2012 | RCT | United States | 101 | NA | Con | Ac | R |  | A | Adults | Influenza | Pre- | Healthcare | 41.6 | 58.4 | NA | 0.05 |
| 84 | Ho 2019 | CRCT | Singapore | 8837 | 22 | Con | E |  |  | A | Older adults | Influenza | Pre- | Healthcare | 45.1 | 54.9 | NA | 0.05 |
| 85 | Hofstetter 2015a | RCT | United States | 2054 | NA | Con | R | R |  | A | Young children | CV | Pre- | Healthcare | 51.2 | 48.8 | NA | 0.61 |
| 85 | Hofstetter 2015b | RCT | United States | 5462 | NA | Con | ER | ER |  | A | Children | Influenza | Pre- | Healthcare | 51.4 | 48.6 | NA | 0.35 |
| 88 | Howell-Jones 2023a | CRCT | United Kingdom | 21786 | 257 | Con | E |  |  | A | Children | Influenza | Pre- | Healthcare | 51.1 | 48.9 | NA | 0.23 |
| 88 | Howell-Jones 2023b | CRCT | United Kingdom | NA | 2994 | Con | E | R | ER | F | Children | Influenza | Pre- | Education | 50.0 | 50.0 | 84.5 | 0.62 |
| 90 | Hu 2017 | RCT | China* | 1252 | NA | Con | E |  |  | S | Young children | CV | Pre- | Healthcare | 0.0 | 100.0 | NA | 0.83 |
| 91 | Hu 2018 | RCT | China* | 204 | NA | Con | E | E |  | A | Young children | CV | Pre- | Healthcare | 0.0 | 100.0 | NA | 0.57 |
| 92 | Huf 2024 | RCT | United Kingdom | 69805 | NA | Con | R | ER |  | A | Adults | COVID-19 | Post- | Healthcare | 49.0 | 51.0 | 45.1 | 0.27 |
| 93 | Hull 2002 | CRCT | United Kingdom | 1318 | 1206 | Con | R |  |  | A | Older adults | Influenza | Pre- | Healthcare | 45.4 | 54.6 | NA | 0.44 |
| 94 | Humiston 2014a | CRCT | United States | 12490 | 31 | Con | Ac |  |  | A | Children | Influenza | Pre- | Education | 50.0 | 50.0 | 43.4 | 0.30 |
| 94 | Humiston 2014b | CRCT | United States | 12876 | 32 | Con | Ac |  |  | A | Children | Influenza | Pre- | Education | 50.0 | 50.0 | 42.4 | 0.29 |
| 95 | Hurley 2018a | RCT | United States | 25039 | NA | Con | R |  |  | A | Adults | Influenza | Pre- | Healthcare | 28.7 | 71.3 | NA | 0.18 |
| 95 | Hurley 2018b | RCT | United Kingdom | 5332 | NA | Con | R |  |  | A | Older adults | Influenza | Pre- | Healthcare | 38.5 | 61.5 | NA | 0.30 |
| 96 | Hurley 2019 | RCT | United States | 616 | NA | Con | R |  |  | A | Older adults | CV, Influenza, Pneumo | Pre- | Healthcare | 36.0 | 64.0 | NA | 0.39 |
| 97 | Hurtaud 2023 | CRCT | France | 1975 | 19 | Con | E |  |  | A | Adults | CV | Post- | Healthcare | 50.0 | 50.0 | NA | 0.15 |
| 98 | Hwang 2010a | RCT | United States | 630 | NA | Con | Ac |  |  | S | Adults | HBV | Pre- | Community/Other | 75.1 | 24.9 | 9 | 0.73 |
| 99 | Hwang 2010b | RCT | United States | 630 | NA | E | M |  |  | S | Adults | HBV | Pre- | Community/Other | 78.9 | 21.1 | 12 | NA |
| 100 | Irigoyen 2006 | RCT | United States | 1662 | NA | Con | R | R |  | A | Young children | CV | Pre- | Healthcare | 50.0 | 50.0 | NA | 0.54 |
| 101 | Isrctn, 2021 | CRCT | Germany | 41548 | 10032 | Con | Aff |  |  | A | Adults | COVID-19 | Post- | Healthcare | 48.7 | 51.3 | NA | 0.02 |
| 102 | Jackson 2011 | CRCT | United Kingdom | 142 | 12 | Con | E |  |  | A | Young children | CV | Pre- | Healthcare | 5.6 | 94.4 | NA | 0.73 |
| 104 | Janitz 2023 | RCT | United States | 312 | NA | Con | E |  |  | A | Adults | COVID-19 | Post- | Community/Other | 21.4 | 78.6 | 76 | 0.29 |
| 105 | Jiang 2022 | RCT | China* | 350 | NA | Con | E |  |  | A | Older adults | Influenza | Post- | Healthcare | 45.1 | 54.9 | NA | 0.03 |
| 106 | Johansen 2023 | CRCT | Denmark | 964870 | 691820 | Con | ER |  |  | A | Older adults | Influenza | Post- | Healthcare | 48.5 | 51.5 | NA | 0.80 |
| 107 | Johnson 2003 | RCT | United States | 32698 | NA | ER | R |  |  | A | Older adults | Pneumo | Pre- | Community/Other | 50.0 | 50.0 | NA | NA |
| 108 | Jordan 2015 | RCT | United States | 18186 | NA | ER | R |  |  | A | Pregnant women | Influenza | Pre- | Healthcare | 0.0 | 100.0 | NA | NA |
| 109 | Joseph 2016 | RCT | United States | 200 | NA | Con | E |  |  | F | Adolescents | HPV | Pre- | Healthcare | 0.0 | 100.0 | NA | 0.54 |
| 110 | Ju, 2024 | RCT | China* | 220 | NA | Con | E |  |  | A | Adults | COVID-19 | Post- | Online | 34.1 | 65.9 | NA | 0.09 |
| 111 | Juon 2016 | RCT | United States | 232 | NA | Con | R |  |  | S | Adults | Hepatitis B | Pre- | Community/Other | 43.1 | 56.9 | NA | 0.17 |
| 116 | Kempe 2001 | RCT | United States | 603 | NA | Con | R |  |  | S | Young children | CV | Post- | Healthcare | 66.0 | 34.0 | 15 | 0.07 |
| 115 | Kempe 2005 | RCT | United States | 5193 | NA | Con | R |  |  | A | Children | Influenza | Pre- | Healthcare | 50.4 | 49.6 | NA | 0.58 |
| 114 | Kempe 2012 | RCT | United States | 264 | NA | Con | R |  |  | A | Adolescents | CV, HPV, MenACWY | Pre- | Education | 100.0 | 0.0 | NA | 0.45 |
| 117 | Kempe 2016 | CRCT | United States | 929 | 7 | Con | R |  |  | S | Adolescents | HPV | Pre- | Healthcare | 65.2 | 34.8 | NA | 0.38 |
| 118 | Kempe 2020 | CRCT | United States | 120130 | 188 | Con | R | R | R | A | Children | Influenza | Pre- | Healthcare | 50.0 | 50.0 | NA | 0.22 |
| 119 | Kerpelman 2000 | RCT | United States | 4150 | 2500 | Con | Aff |  |  | S | Young children | CV | Pre- | Community/Other | 49.5 | 50.5 | 14.2 | 0.61 |
| 120 | Khan 2023 | RCT | United States | 7411 | NA | Con | R | R |  | A | Adolescents | HPV | Post- | Healthcare | 52.0 | 48.0 | 24.9 | 0.03 |
| 87 | Kopfer 2012 | RCT | United States | 404 | NA | Con | E | E | E | F | Adults | HPV | Pre- | Education | 0.0 | 100.0 | 71.8 | 0.12 |
| 122 | Krieger 2000 | RCT | United States | 1246 | NA | Con | ER |  |  | A | Older adults | Influenza | Pre- | Community/Other | 54.7 | 45.3 | NA | 0.82 |
| 123 | Kulle, 2024 | CRCT | Switzerland | 20414 | 20 | Con | Ac |  |  | A | Adults | COVID-19 | Post- | Community/Other | 49.7 | 50.3 | NA | 0.04 |
| 125 | Lau 2012 | RCT | Australia | 742 | NA | Con | ER |  |  | A | Adults | Influenza | Pre- | Education | 43.0 | 57.0 | NA | 0.05 |
| 126 | LeBaron 2004 | RCT | United States | 3050 | NA | Con | R | R | R | S | Young children | CV | Pre- | Community/Other | 49.0 | 51.0 | 7 | 0.34 |
| 127 | Lee 2020 | RCT | United States | 50286 | NA | Con | Aff | R |  | A | Adults | Influenza | Pre- | Online | 37.9 | 62.1 | NA | 0.22 |
| 128 | Lerner 2021a | RCT | United States | 22046 | NA | Con | ER |  |  | A | Children | Influenza | Post- | Healthcare | 50.8 | 49.2 | 41.3 | 0.57 |
| 128 | Lerner 2021b | RCT | United States | 22046 | NA | Con | E |  |  | A | Children | Influenza | Post- | Healthcare | 50.8 | 49.2 | 41.3 | 0.57 |
| 128 | Lerner 2021c | RCT | United States | 689 | NA | Con | R |  |  | S | Children | Influenza | Post- | Healthcare | 50.8 | 49.2 | 41.3 | 0.44 |
| 129 | Leung 2017 | RCT | Hong Kong SAR, China | 529 | NA | Con | E |  |  | A | Older adults | Influenza | Pre- | Healthcare | 47.4 | 52.6 | NA | 0.25 |
| 130 | Liao 2020 | RCT | Hong Kong SAR, China | 365 | NA | Con | ER |  |  | A | Children | Influenza | Pre- | Online | 0.0 | 100.0 | NA | 0.38 |
| 131 | Lieu 2022 | RCT | United States | 8287 | NA | Con | ER | ER |  | A | Older adults | COVID-19 | Post- | Healthcare | 43.7 | 56.3 | NA | 0.22 |
| 132 | Lin 2020 | RCT | Taiwan, China | 282 | NA | Con | E |  |  | A | Young children | CV | Pre- | Healthcare | 22.8 | 77.2 | NA | 0.71 |
| 133 | Ma 2018 | CRCT | United States | 1834 | 32 | Con | E |  |  | S | Adults | Hepatitis B | Pre- | Community/Other | 41.3 | 58.7 | NA | 0.18 |
| 134 | Ma 2021 | RCT | United States | 180 | NA | Con | ER |  |  | S | Adolescents | HPV | Post- | Healthcare | 35.6 | 64.4 | NA | 0.00 |
| 135 | Mantzari 2015 | RCT | United Kingdom | 1000 | NA | Con | Aff |  |  | S | Adolescents | HPV | Pre- | Healthcare | 0.0 | 100.0 | NA | 0.08 |
| 137 | Mason 2000 | RCT | United Kingdom | 511 | NA | Con | R |  |  | A | Young children | CV | Pre- | Healthcare | 50.0 | 50.0 | NA | 0.07 |
| 138 | Masson 2013 | RCT | United States | 489 | NA | E | M |  |  | S | Adults | HBV | Pre- | Healthcare | 68.3 | 31.7 | 36 | NA |
| 139 | McCaul 2002 | CRCT | United States | 23733 | 49 | Con | R | ER |  | F | Older adults | Influenza | Pre- | Healthcare | 50.0 | 50.0 | NA | 0.20 |
| 140 | Meharry 2014 | RCT | United States | 135 | NA | Con | E | E |  | A | Pregnant women | Influenza | Pre- | Healthcare | 50.0 | 50.0 | 30.7 | 0.47 |
| 141 | Menzies 2020 | RCT | Australia | 1594 | NA | Con | R | R | R | U | Young children | CV | Pre- | Healthcare | 50.0 | 50.0 | NA | 0.74 |
| 145 | Moniz 2013 | RCT | United States | 216 | NA | Con | E |  |  | F | Pregnant women | Influenza | Pre- | Healthcare | 50.0 | 50.0 | 25.9 | 0.31 |
| 146 | Munoz-Miralles 2022 | CRCT | Spain | 210 | 57 | Con | E |  |  | S | Older adults | Influenza | Pre- | Healthcare | 50.0 | 50.0 | NA | 0.15 |
| 148 | NCT05012163 2024 | RCT | United States | 57581 | NA | Con | M | Aff | R | A | Adults | Influenza | Post- | Healthcare | 42.1 | 57.9 | 93.5 | 0.25 |
| 150 | NCT05248399, 2022 | RCT | United States | 199 | NA | Con | E |  |  | A | Adults | COVID-19 | Post- | Community/Other | 99.5 | 0.5 | NA | 0.10 |
| 151 | NCT05534061 2022 | RCT | United States | 349 | NA | M | Aff |  |  | A | Adults | COVID-19 | Post- | Community/Other | 63.0 | 37.0 | 63.6 | NA |
| 152 | NCT05537441 2022 | RCT | United States | 124793 | NA | Con | R |  |  | A | Adults | Influenza | Post- | Healthcare | 53.2 | 46.8 | NA | 0.27 |
| 153 | Nehme 2019 | RCT | United States | 25649 | NA | Con | M | M |  | A | Adults | Influenza | Pre- | Healthcare | 49.3 | 50.7 | NA | 0.14 |
| 154 | Nowalk 2010 | CRCT | United States | 12222 | 54 | Con | R | Aff |  | A | Adults | Influenza | Pre- | Community/Other | 57.2 | 42.8 | NA | 0.05 |
| 158 | O'Grady 2022 | RCT | Australia | 196 | NA | Con | R | M |  | S | Young children | CV | Pre- | Healthcare | 0.0 | 100.0 | NA | 0.67 |
| 159 | O'Leary 2015 | RCT | United States | 4587 | NA | Con | R |  |  | A | Adolescents | CV, HPV, MenACWY | Pre- | Healthcare | 53.5 | 46.5 | NA | 0.15 |
| 157 | O'Leary 2019 | RCT | United States | 1093 | NA | Con | E | E |  | A | Pregnant women | Influenza | Pre- | Online | 0.0 | 100.0 | 88.2 | 0.36 |
| 160 | Omer 2022 | RCT | United States | 1045 | NA | Con | E |  |  | F | Pregnant women | Influenza | Pre- | Healthcare | 0.0 | 100.0 | 55 | 0.61 |
| 161 | Osborne, 2023 | RCT | United States | 702 | NA | Con | E |  |  | A | Adults | Influenza | Pre- | Online | 27.7 | 72.3 | 84 | 0.47 |
| 162 | Otsuka 2013a | RCT | United States | 674 | NA | Con | E |  |  | A | Older adults | Shingles | Pre- | Healthcare | 46.4 | 53.6 | 87.4 | 0.05 |
| 162 | Otsuka 2013b | RCT | United States | 1915 | NA | Con | E |  |  | A | Older adults | Shingles | Pre- | Healthcare | 43.7 | 56.3 | 72.4 | 0.02 |
| 163 | Otsuka-Ono 2019 | RCT | Japan | 175 | NA | Con | E |  |  | A | Young children | Hepatitis B | Pre- | Healthcare | 50.0 | 50.0 | NA | 0.49 |
| 165 | Patel 2014 | CRCT | United States | 365 | 10 | Con | R |  |  | S | Adults | HPV | Pre- | Healthcare | 0.0 | 100.0 | 57 | 0.19 |
| 166 | Patel 2023 | RCT | United States | 74811 | NA | Con | R |  |  | A | Adults | Influenza | Post- | Healthcare | 44.2 | 55.8 | 70.6 | 0.30 |
| 168 | Porter-Jones 2009 | RCT | United Kingdom | 974 | NA | Con | E |  |  | F | Young children | CV | Pre- | Online | 53.3 | 46.7 | NA | 0.88 |
| 169 | Pot 2017 | RCT | Netherlands | 9124 | NA | Con | E |  |  | A | Adolescents | HPV | Pre- | Online | 50.0 | 50.0 | NA | 0.73 |
| 170 | Qin 2023 | RCT | China* | 100 | NA | Con | Aff |  |  | S | Adolescents | HPV | Post- | Healthcare | 16.0 | 84.0 | NA | 0.78 |
| 171 | Quinlivan 2003 | RCT | Australia | 139 | NA | Con | E |  |  | S | Young children | CV | Pre- | Community/Other | 0.0 | 100.0 | NA | 0.72 |
| 173 | Rand 2015 | RCT | United States | 3812 | NA | Con | R |  |  | S | Adolescents | HPV | Pre- | Healthcare | 54.6 | 45.4 | NA | 0.01 |
| 174 | Rand 2017 | RCT | United States | 749 | NA | Con | R | R |  | S | Adolescents | HPV | Pre- | Healthcare | 66.2 | 33.8 | 16.7 | 0.35 |
| 176 | Reiter 2018 | RCT | United States | 150 | NA | Con | ER |  |  | S | Adolescents | HPV | Pre- | Online | 100.0 | 0.0 | 55.4 | 0.03 |
| 175 | Reiter 2023 | RCT | United States | 1227 | NA | Con | ER | ER |  | S | Adolescents | HPV | Post- | Online | 100.0 | 0.0 | 47 | 0.03 |
| 178 | Richman 2014 | RCT | United States | 256 | NA | Con | R |  |  | S | Adolescents | HPV | Pre- | Healthcare | 11.7 | 88.3 | 9.3 | 0.29 |
| 177 | Richman 2016 | RCT | United States | 264 | NA | Con | R |  |  | S | Adolescents | HPV | Pre- | Healthcare | 37.5 | 62.5 | 53.4 | 0.32 |
| 180 | Roca 2012 | RCT | Spain | 2402 | NA | Con | E |  |  | A | Older adults | Influenza | Pre- | Healthcare | 44.3 | 55.7 | 99.1 | 0.37 |
| 182 | Rodriguez 2022 | CRCT | United States | 541 | 7 | Con | E |  |  | A | Adults | COVID-19 | Post- | Healthcare | 58.5 | 41.5 | 36.9 | 0.09 |
| 181 | Rodriguez 2024 | CRCT | United States | 767 | 6 | Con | ER | R |  | A | Adults | Influenza | Post- | Healthcare | 53.5 | 46.5 | 37.3 | 0.15 |
| 184 | Saaksvuori 2022 | CRCT | Finland | 47595 | 34690 | Con | M | M |  | A | Older adults | Influenza | Pre- | Healthcare | 43.8 | 56.2 | NA | 0.34 |
| 185 | Saccardo 2024 | RCT | United States | 386615 | NA | Con | R |  |  | A | Adults | COVID-19 | Post- | Healthcare | 42.2 | 57.8 | NA | 0.12 |
| 187 | Santa Maria 2021 | RCT | United States | 519 | NA | Con | E |  |  | A | Adolescents | HPV | Pre- | Education | 9.7 | 90.3 | 2.7 | 0.60 |
| 188 | Scarinci 2020 | CRCT | United States | 278 | 40 | Con | E |  |  | S | Adolescents | HPV | Pre- | Community/Other | 0.0 | 100.0 | NA | 0.03 |
| 190 | Scott, 2019 | RCT | United States | 402 | NA | Con | E | E |  | A | Children | Influenza | Pre- | Healthcare | 4.8 | 95.2 | NA | 0.65 |
| 191 | Shegog 2022 | CRCT | United States | 512 | 51 | Con | ER |  |  | F | Adolescents | HPV | Pre- | Healthcare | 4.8 | 95.2 | 55.9 | 0.32 |
| 192 | Shen, 2024 | RCT | China* | 720 | NA | Con | Aff |  |  | A | Adults | Influenza | Post- | Healthcare | 46.4 | 53.6 | NA | 0.37 |
| 193 | Shourie 2013 | CRCT | United Kingdom | 220 | 50 | Con | E | E |  | F | Young children | CV | Post- | Online | 50.0 | 50.0 | 91.4 | 0.99 |
| 194 | Si 2022 | CRCT | China* | 3968 | NA | Con | E |  |  | A | Adults | HPV | Post- | Education | 0.0 | 100.0 | NA | 0.00 |
| 195 | Sitler 2018 | RCT | United States | 129 | NA | Con | Ac |  |  | A | Children | Influenza | Pre- | Healthcare | 2.4 | 97.6 | NA | 0.16 |
| 199 | Stockwell 2012a | RCT | United States | 9213 | NA | Con | ER |  |  | A | Children | Influenza | Pre- | Healthcare | 49.3 | 50.7 | 1.4 | 0.18 |
| 199 | Stockwell 2012b | RCT | United States | 361 | NA | Con | R |  |  | A | Adolescents | CV, MenACWY | Pre- | Healthcare | 42.1 | 57.9 | 3 | 0.40 |
| 202 | Stockwell 2014 | RCT | United States | 1187 | NA | Con | E |  |  | A | Pregnant women | Influenza | Pre- | Healthcare | 0.0 | 100.0 | NA | 0.47 |
| 198 | Stockwell 2015 | RCT | United States | 662 | NA | Con | R | R |  | A | Children | Influenza | Pre- | Healthcare | 50.5 | 49.5 | 0.3 | 0.57 |
| 201 | Stockwell 2022 | RCT | United States | 2086 | NA | Con | ER |  |  | S | Children | Influenza | Pre- | Healthcare | 11.2 | 88.8 | 43.4 | 0.81 |
| 203 | Stolpe 2019 | RCT | United States | 22301 | NA | Con | E |  |  | A | Adults | Pneumo | Pre- | Healthcare | 42.7 | 57.3 | NA | 0.01 |
| 204 | Strathdee 2023 | RCT | United States | 150 | NA | Con | E |  |  | A | Adults | COVID-19 | Post- | Community/Other | 63.0 | 37.0 | NA | 0.17 |
| 205 | Stuck 2015 | RCT | Switzerland | 2284 | NA | Con | E |  |  | A | Older adults | Influenza | Pre- | Healthcare | 43.4 | 56.6 | NA | 0.59 |
| 206 | Suh 2012 | RCT | United States | 1600 | NA | Con | ER |  |  | A | Adolescents | CV, HPV | Pre- | Healthcare | 39.1 | 60.9 | NA | 0.35 |
| 207 | Suzuki, 2022 | RCT | Japan | 2175 | NA | Con | E |  |  | A | Adolescents | HPV | Post- | Online | 58.2 | 41.8 | NA | 0.09 |
| 208 | Sweeney 2014 | RCT | United States | 82 | NA | Con | E |  |  | A | Adolescents | HPV | Pre- | Education | 0.0 | 100.0 | 56.1 | 0.05 |
| 217 | Szilagyi 2006 | RCT | United States | 3006 | NA | Con | R |  |  | A | Adolescents | CV | Pre- | Healthcare | 50.8 | 49.2 | NA | 0.26 |
| 216 | Szilagyi 2011 | RCT | United States | 7546 | NA | Con | M |  |  | S | Adolescents | HPV | Pre- | Healthcare | 50.3 | 49.7 | 12.3 | 0.24 |
| 211 | Szilagyi 2013 | CRCT | United States | 7404 | 5559 | Con | R | R |  | S | Adolescents | HPV | Pre- | Healthcare | 50.5 | 49.5 | NA | 0.04 |
| 218 | Szilagyi 2018 | CRCT | United States | 18921 | 42 | Con | Ac |  |  | A | Children | Influenza | Pre- | Education | 50.0 | 50.0 | NA | 0.48 |
| 219 | Szilagyi 2019 | CRCT | United States | 15768 | NA | Con | R |  |  | A | Children | Influenza | Pre- | Education | 50.0 | 50.0 | NA | 0.40 |
| 209 | Szilagyi 2020a | RCT | United States | 62118 | NA | Con | ER | ER | ER | S | Adolescents | HPV | Pre- | Healthcare | 53.0 | 47.0 | NA | 0.37 |
| 209 | Szilagyi 2020b | RCT | United States | 164205 | NA | Con | E | E | E | A | Adults | Influenza | Pre- | Healthcare | 41.7 | 58.3 | 57.3 | 0.28 |
| 209 | Szilagyi 2020c | RCT | United States | 85776 | NA | Con | R | R | R | A | Children | Influenza | Pre- | Healthcare | 50.0 | 50.0 | NA | 0.28 |
| 215 | Szilagyi 2024a | RCT | United States | 24681 | NA | Con | R | R |  | A | Children | Influenza | Post- | Healthcare | 50.0 | 50.0 | NA | 0.52 |
| 215 | Szilagyi, 2024b | RCT | United States | 79955 | NA | Con | R | R |  | A | Adults | Influenza | Post- | Healthcare | 50.0 | 50.0 | NA | 0.43 |
| 215 | Szilagyi, 2024c | RCT | United States | 31044 | NA | Con | R | R |  | A | Older adults | Influenza | Post- | Healthcare | 50.0 | 50.0 | NA | 0.61 |
| 220 | Tentori 2022 | RCT | Italy | 1957 | NA | R | M |  |  | A | Adults | COVID-19 | Post- | Healthcare | 53.0 | 47.0 | NA | NA |
| 221 | Terrell-Perica 2001 | RCT | United States | 6528 | NA | Con | R | R |  | A | Adults | Influenza, Pneumo | Pre- | Healthcare | 44.0 | 56.0 | 25 | 0.02 |
| 222 | Thilly, 2024 | CRCT | France | 14822 | 91 | Con | M | E |  | A | Adolescents | HPV | Post- | Education | 50.0 | 50.0 | NA | 0.20 |
| 223 | Tiro 2015 | RCT | United States | 875 | NA | ER | R |  |  | S | Adolescents | HPV | Pre- | Healthcare | 50.0 | 50.0 | NA | NA |
| 224 | Topp 2013 | RCT | Australia | 201 | NA | Con | Aff |  |  | S | Adults | HBV | Pre- | Healthcare | 77.0 | 23.0 | NA | 0.66 |
| 225 | Tubiana 2021 | CRCT | France; Monaco | 1475 | 18 | ER | E |  |  | A | Older adults | Influenza, Pneumo | Pre- | Healthcare | 50.1 | 49.9 | NA | NA |
| 226 | Tull 2019 | RCT | Australia | 4386 | NA | Con | ER | R |  | A | Adolescents | HPV | Pre- | Education | 49.9 | 50.1 | NA | 0.86 |
| 227 | Ueberroth 2022 | RCT | United States | 16728 | NA | Con | R | R |  | A | Adults | Influenza | Post- | Healthcare | 47.6 | 52.4 | NA | 0.14 |
| 229 | Usami 2009 | CRCT | Japan | 1863 | 84 | Con | E |  |  | A | Older adults | Influenza | Pre- | Healthcare | 32.0 | 68.0 | NA | 0.65 |
| 230 | Vanderpool 2013 | RCT | United States | 344 | NA | Con | ER |  |  | S | Adolescents | HPV | Pre- | Community/Other | 0.0 | 100.0 | 94 | 0.32 |
| 231 | Viver 2000 | RCT | United States | 264 | NA | Con | R | R | R | S | Young children | CV | Pre- | Healthcare | 50.0 | 50.0 | NA | 0.03 |
| 234 | Wang 2021 | RCT | Hong Kong SAR, China | 624 | NA | Con | E | E |  | S | Adults | HPV | Pre- | Community/Other | 100.0 | 0.0 | NA | 0.07 |
| 233 | Wang 2023 | RCT | Hong Kong SAR, China | 396 | NA | Con | E |  |  | A | Older adults | Influenza | Post- | Online | 37.1 | 62.9 | NA | 0.35 |
| 235 | Weaver 2014 | CRCT | United Kingdom | 210 | 12 | Con | Aff | Aff |  | S | Adults | HBV | Pre- | Healthcare | 79.5 | 20.5 | 75 | 0.21 |
| 236 | Wijesundara 2020a | RCT | United States | 39011 | NA | Con | E |  |  | S | Adults | Influenza | Pre- | Healthcare | 37.2 | 62.8 | 72.8 | 0.27 |
| 236 | Wijesundara 2020b | RCT | United States | 58596 | NA | Con | E |  |  | S | Adults | Influenza | Pre- | Healthcare | 50.9 | 49.1 | 64.2 | 0.08 |
| 238 | Winston 2007 | RCT | United States | 2395 | NA | Con | R |  |  | A | Older adults | Pneumo | Pre- | Healthcare | 40.2 | 59.8 | NA | 0.08 |
| 239 | Wiseman 2016 | RCT | United States | 136 | NA | Con | ER |  |  | A | Children | Influenza | Pre- | Healthcare | 5.9 | 94.1 | 2.2 | 0.45 |
| 240 | Wong 2016 | RCT | Hong Kong SAR, China | 321 | NA | Con | E |  |  | A | Pregnant women | Influenza | Pre- | Healthcare | 0.0 | 100.0 | NA | 0.10 |
| 242 | Wouters, 2007 | RCT | Belgium | 615 | NA | Con | Ac |  |  | S | Adults | HBV | Post- | Healthcare | 6.7 | 93.3 | NA | 0.54 |
| 243 | Wright 2012 | CRCT | United States | 3979 | 11 | Con | R |  |  | A | Adults | Influenza | Pre- | Healthcare | 39.8 | 60.2 | 87.1 | 0.14 |
| 244 | Wynn 2021 | RCT | United States | 956 | NA | R | ER |  |  | S | Adolescents | HPV | Pre- | Healthcare | 50.0 | 50.0 | NA | NA |
| 245 | Xu 2022 | RCT | China* | 246 | NA | Con | ER |  |  | U | Young children | CV | Post- | Community/Other | 15.3 | 84.7 | NA | 0.24 |
| 246 | Yeung 2018 | RCT | Hong Kong SAR, China | 833 | NA | Con | M |  |  | A | Children | Influenza | Pre- | Healthcare | 0.0 | 100.0 | NA | 0.13 |
| 247 | Yokum 2018 | RCT | United States | 227955 | NA | Con |  |  |  | A | Older adults | Influenza | Pre- | Healthcare | 45.0 | 55.0 | NA | 0.26 |
| 248 | Yudin 2016 | RCT | Canada | 317 | NA | Con | ER |  |  | A | Pregnant women | Influenza | Pre- | Healthcare | 0.0 | 100.0 | 50 | 0.27 |
| 250 | Zhang 2018b | RCT | Hong Kong SAR, China | 312 | NA | Con | E | E |  | A | Older adults | Influenza | Pre- | Community/Other | 16.7 | 83.3 | NA | 0.64 |
| 252 | Zhang 2022 | RCT | China* | 946 | NA | Con | E |  |  | A | Adults | HPV | Post- | Education | 0.0 | 100.0 | NA | 0.02 |
| 253 | Zuniga de Nuncio 2003 | RCT | United States | 348 | NA | Con | ER |  |  | U | Young children | CV | Pre- | Healthcare | 0.0 | 100.0 | NA | 0.93 |

**Footnotes**

Key: C: Cluster RCT, I Individually randomised RCT

A: Any, F: First, S: Series completion, U: Up-to-date

Pre-: before 2020; Post-: 2020 onwards

CV: Childhood vaccines, C-19: COVID-19, Flu: Influenza, HPV: Human papilloma vaccine, HBV: HBV vaccine

Con: Control, Ac: Access, Af: Affordability, E: Education, ER: Education and reminder, R: Reminder, M: Multicomponent

NA: Not Applicable

*Denotes upper-middle income countries

Control proportion vaccinated: Where studies included a control arm we present proportion vaccinated to give an approximation of baseline vaccination levels. The median proportion, across all control arms was 0.28 (min = 0, max = 0.99); Inter Quartile Range = 0.43 (upper quartile = 0.50, lower quartile = 0.07).

## **L: Socio-demographic and socio-economic characteristics of study participants**

| **Study** | **Religion** | **Education level** | **Socio-economic data** |
| --- | --- | --- | --- |
| Abroms 2023 | Not reported | (N = 478): High school or less 104 (21.8%), Associate’s degree/some college 234 (49.0%), and Bachelor’s/graduate degree 140 (29.3%). | Income: Less than US$20,000: N = 82 (17.2%), US$20,000 to US$49,999: N = 193 (40.4%), More than US$50,000: N = 198 (41.4%) and Prefer not to answer: N = 5 (1.0%). Employment: Working: N = 341 (71.3%) |
| Alonge 2023 | Not reported | Not reported | (n=4296): Has public health insurance = 2502 (58.2%) |
| Alsan, 2024 | Not reported | Completed high school: M = 0.88 (SD = 0.32) | Low household income: M = 0.53 (SD = 0.50) - Low household income is a binary variable equal to 1 if the respondent’s self-reported household income is less than or equal to the median income of Black respondents in the sample (= $30k) |
| Anraad 2023 | Affiliation with religion (1 = no affiliation – 7 = strong affiliation----> Mean (standard deviation)): control=2.13 (1.33) and intervention=2.06 (1.29) | Highest education completed (n=1236): low (less than secondary or vocational education)= 6 (0.5), intermediate (secondary and vocational education)= 265 (21.4%), and high (higher or university education)= 965 (78.1%) | Not reported |
| Aref 2022 | Not reported | N=121: High school diploma=111 (91.7), Two-year undergraduate degree=6 (5.0), Bachelor’s degree=3 (2.5), and other= 1(0.8%) | Immediate family’s total annual household income ($): Less than 20,000 per year: N = 9 (7.5%), 20,000-69,999: N = 33 (27%), 70,000-139,999: N = 51 (42.5%), and 140,000 or more per year: N = 27 (22.5%) |
| Arnold 2022 | Not reported | Not reported | Not reported |
| Arthur 2002 | Not reported | Not reported | Not reported |
| Bartos 2022 | Not reported | Full sample used (n = 2,101).: primary 4.6%, lower secondary 27.7%, upper secondary 36.3%, and university 31.5% | Household income: Up to 10,000 CZK= 1.4%, 10,001 - 15,000 CZK =6.5%, 15,001 - 20,000 CZK =9.5%, 20,001 - 25,000 CZK =7.5%, 25,001 - 30,000 CZK =10.8%, 30,001 - 35,000 CZK =12.3%, 35,001 - 40,000 CZK =10.9%, 40,001 - 50,000 CZK =12.2%, 50,001 - 60,000 CZK =9.0%, Over 60,000 CZK= 8.5%, and I don't know / Don't want to say 11.5%. Economic status: Employee 48.0%, Entrepreneur 4.6%, Student 3.5%, Parental leave 3.9%, Retired 34.8%, Unemployed 3.6%, Other 1.6% |
| Bartu 2006 | Not reported | Education: High school not completed: 41% (63/152); High school completed: 15% (23/152); Technical college: 18% (27/152); University 2% (3/152); Other (trade, apprenticeship, professional registration): 21% (32/152) | Total income in previous year (n = 147): 70% (103/147) Less than $A20 000, 26% (38/147) $A20 000–40 000, 4% (6/147) More than $A40 000; Employed (n=147): 22% (32/147) Full time, 30% (45/147) part time/causal, and 48% (70/147) not employed |
| Baskin 2018 | Not reported | All participants in this study (n = 30,748) were students, faculty and staff at a large university in the United States; 18% undergraduate students, 23% were graduate and professional students, and rest is various employees from high school degree to being close to retirement | Not reported |
| Bastani 2022 | Not reported | Grade school N = 88 (37%), High school diploma N = 89 (37.4%), college N = 55 (23.1%), Post college N = 6 (2.5%) | Annual household income (n=238): <$12,000: N = 64 (26.9%), $12,000–<$24,000: N = 123 (51.7%), $24,000–$36,000: N = 35 (14.7%), $36,000: N = 13 (5.5%), Other: N = 1 (0.4%), and Do not know/refuse to answer: N = 2 (0.8%). Insurance status: Medi-Cal or Healthy Families: N = 196 (82.3%), Other insurance: N = 14 (5.9%), and Uninsured: N =28 (11.8%) |
| Bennett 2015 | Not reported | Student standing (n=661): Undergraduate 445 (67.3%), Graduate 182 (27.5%), and Professional 34 (5.1%) | Employment: Full-time: N = 55 (8.3%), Part-time: N = 323 (48.9%), Unemployed: N = 280 (42.4%), and Missing: N = 3 (0.5%). Health insurance: UM SHIP or GradCare: N = 106 (16.0%), Parent’s private: N = 438 (66.3%), Medicaid: N = 10(1.5), Other insurance: N = 41 (6.2%), None: N = 43 (6.5%), and Don’t know/missing: N = 23 (3.5%) |
| Berg 2004 | Not reported | Not reported | Not reported |
| Berg 2008 | Not reported | Not reported | Not reported |
| Berkhout 2018 | Not reported | Not reported | Not reported |
| Bernard-Genest 2021 | Not reported | n=127: High school diploma 30(23.6%), college 39(30.7%), Bachelor’s degree 36(28.3%), Master’s degree 21 (16.5%), and PhD 1(0.8%) | Not reported |
| Berset 2022 | Not reported | Not reported | Insurance (n=945): Public (i.e., Medicaid): N = 807 (85.4%): Private: N = 112 (11.9%), and self-pay: N = 26 (2.8%). The study population included predominantly non-Hispanic Black, low-income children (age, 6-17 years) |
| Berset 2023 | Not reported | Not reported | Insurance (n=1312): Public (i.e., Medicaid): N =1140 (86.9%), Private: N = 50 (3.8%), Self-pay: N = 119 (9.1%), and Missing data: N = 3 (0.2%). The study was conducted in academic primary care practices serving low-income, predominantly Black patients |
| Bethke 2024 | Not reported | Students (n=6512): 1424 (21.9%) attended high schools, 1714 (26.3%) integrated secondary schools, and 3374 (51.8%) vocational schools. Education mother (n=4537): No education degree = 236 (5.2%), Elementary – Primary school degree= 342(7.5%), Secondary school degree= 1558(34.3%), High school degree= 1049(23.1%), and University degree = 1352 (29.8%). Education father (4154): No education degree= 230(5.5%), Elementary – Primary school degree=341 (8.2%), Secondary school degree=1323 (31.8%), High school degree = 848(20.4%), and University degree=1412 (34%) | Employment status of parents (N = 6310): Both unemployed: N = 451 (7.1%), One employed: N = 1775 (28.1%), and Both employed: N = 4084 (64.7%) |
| Bian 2023 | Not reported | College students (n=202): Junior college student/ undergraduate student 177 (87.6%) and Graduate student 25 (12.4%). Grandparent (n=375): Junior high school and below 320 (85.3%) and High school and above 55 (14.7%) | Not reported |
| Borg 2018 | Not reported | Not reported | There are data for socio-economic index for areas (SEIFA) quintile for each groups, but not possible to extract the overall percentage: Around 30% of children in each group lived in the most disadvantaged socio-economic quintile area of Victoria |
| Bourgeois 2008 | Not reported | Not reported | All participants were employees |
| Bowman, 2014 | Not reported | Not reported | Not reported |
| Brigham 2012 | Not reported | Not reported | Insurance type (n=420): - Private: N = 220 (52.4%), Public: N = 190 (45.2%), None: N = 10 (2.4%) |
| Bronchetti 2015 | Not reported | Not reported | Zip code median income (Means by treatment group): 9.015 (3.560) for control, 8.964 (3.621) for incentive, 9.836 (3.521) for peer, and 9.095 (3.627) for coughing. Note: Sample sizes for means for zip code median income are 2168, 2171, 2172, and 2154, respectively, because zip codes are missing for international students |
| Burkhardt 2022 | Not reported | Not reported | Insurance (n=1235): Public (i.e., Medicaid): N = 1090 (88.3%), Private: N =121 (9.8%), self-pay: N =24 (1.9%). The study took place at 3 academic paediatric primary care practices. These practices serve a predominantly non-Hispanic Black, low-income population |
| Burkhardt 2023 | Not reported | Not reported | Insurance (n=1235): Public (i.e., Medicaid): N = 1090 (88.3%), Private: N =121 (9.8%), self pay: N =24(1.9%). The study took place at 3 academic paediatric primary care practices. These practices serve a predominantly non-Hispanic Black, low-income population |
| Buttenheim 2022 | Not reported | Not reported | Not reported |
| Cameron, 2013 | Not reported | N= 105: <High school 8.6%, High school graduate 19.0%, Some college 27.6%, and >=College 43.8%. | Household income (USD): <$20,000 per year 19.0%, $20,001–60,000 per year 27.6%, $60,001–100,000 per year 16.2%, >$100,000 per year 25.7% |
| Campos-Mercade 2021 | Not reported | Education (n=8286): 2% Elementary School or Lower, 30% High-school,13% Professional Training, 7% In College, 46% College Degree, and 2% PhD | Average monthly income (SEK) = 24,847: 3% Income 0-5000kr, 5% Income 5001-10000kr, 11% Income 10001-15000kr,11% Income 15001-20000kr, 22% Income 20001-25000kr, 20% Income 25001-30000kr, 13% Income 30001-35000kr, 7% Income 35001-40000kr,4% Income 35001-40000kr, Income 45001-50000kr 2%, 1% Income 50000kr-55000kr, and 1% Income more than 55000kr. Occupation: 81% Employed, 4% Unemployed, 11 % in college, 1% Retired, and 4% Other Professional situations |
| Cataldi 2024 | Not reported | Not reported | Not reported |
| Centers for Disease Control and Prevention 2012 | Not reported | Not reported. | Not reported |
| Chai 2013 | Not reported | Total (N=1992): Primary school or below: 42 (2.1%), Junior high: 288 (14.5%), Senior high or secondary school: 788 (39.6%), Junior or full college degree: 842 (42.3%), Graduate degree and above: 32 (1.6%) | Not reported |
| Chang 2023 | Not reported | Not reported | Insurance (N = 57,893): Commercial (2.7%), medical (74.7%), Medicare (12.8%), other (5.7%), and Uninsured (4.1%). The group targeted by the study was a racially/ethnically diverse, primarily low-income adult population |
| Chao 2015 | Not reported | Not reported | Not reported |
| Chodick 2021 | Not reported | Not reported | Socioeconomic level (SES): 23% (5013/21979) Lowest, 32% (6996/21979) Q2, 18% (4099/21979 ) Q3, and 27% (5871/21979) Highest; Median (IQR) socio-economic status [tool used to measure SES not stated]: 6 (4-11) |
| Clayton 2021 | Not reported | Wave 1 (n=2332): Less than high school diploma 1.7%, High school diploma 11.9%, Some college 13.8%, Associate’s degree 7.4%, Bachelor’s degree 26.4%, Some professional or graduate school but no degree 5.8%, and Professional or graduate degree 32.9%. Less than high school diploma: 7 (1%), High school diploma: 74 (10.9%), Some college: 105 (15.5%), Associate’s degree: 55 (8.1%), Bachelor’s degree: 181 (26.7%), Some professional or graduate school but no degree: 49 (7.2%), Professional or graduate degree: 207 (30.5%). | Medicaid: Yes 33.4%, No 66.6%. SCHP: Yes 38.9%, No 61.1%. |
| Coley 2018 | Not reported | Not reported | Not reported |
| Conner 2017 | Not reported | Not reported | Sample mainly lived in areas of low deprivation (Townsend score M = -1.47, SD = 2.93) |
| Cox 2012 | Not reported | High school diploma or more= 889 (76.5%) | Employed=624/1175 (53.1%). Income < $10K= 501/1175 (42.6%) |
| Cutrona 2018 | Not reported | Not reported | Not reported |
| Dai 2021 | Not reported | Not reported | Not reported |
| Dalby 2000 | Not reported | Not reported | Not reported |
| Daley 2002 | Not reported | Not reported | Not reported |
| Daley 2014 | Not reported | All are primary school students (sample of kindergarten to eighth-grade schools). | Percent of student body eligible for free or reduced lunch in intervention schools (min, max): median=67% (17, 91); in control schools median= 61% (5, 93). |
| Daly, 2023 | Not reported | Not reported | Not reported |
| Daniels 2007 | Not specifically reported, however study was conducted in a community church setting. | Not reported | Income less or equal to $30,000: n=92 (65%). Income more than or equal to $30,000: n=50 (35%). Health Insurance Status: Insured: n=127 (71%); Uninsured: n=53 (29%) |
| Dapp 2011 | Not reported | Not reported | Not reported |
| Davies 2017 | Not reported | All are secondary school students. | Not reported |
| DeCamp 2020 | Not reported | Maternal education (n=157): Eighth grade or less= 64 (40.8%), Some high school=41 (26.1%), and High school or greater= 52(33.1%). | Annual family income: <$20 000: N = 67 (42.7%), $20 000–$30 000: N = 38 (24.2%), >$30 000: N = 12 (7.6%), and Did not report or unknown: N = 40 (25.5%) |
| Dempsey 2019 | Not reported | Not reported | Not reported |
| DiClemente 2015 | Not reported | Education: Less than 8th grade N = 13 (6.0%), 8th grade N = 25 (11.6%), 9th grade N = 44 (20.4), 10th grade N = 30 (13.9%), 11th grade N = 42 (19.4%), 12th grade N = 28 (13.0%), High school grad or GED N = 34 (15.7%) | **Received Public Assistance**: No N = 113 (52.3%), Welfare (TANF, SSI) N = 18 (8.3%), Food stamps N = 99 (45.8%), WIC N = 26 (12.0%), Section 8 housing N = 10 (4.6%); Currently Employed N= 34(15.7%); **Health Insurance**: Private N= 19(8.8%), Medical N=97 (44.9%), GA CHIP N=3 (1.4%), No insurance N= 38 (17.6%), and Don't know N= 59 (27.3%) |
| Dini 2000 | Not reported | Not reported | Not reported |
| Dombkowski 2014 | Not reported | Not reported | Not reported |
| Dombkowski 2017 | Not reported | Not reported | Medicaid enrolment status (n=1497): Never enrolled 997(66.6%), Previously enrolled 121(8.1%), Currently enrolled 313(20.9%), and Unknown enrolment status 66(4.4%) |
| Domek 2019 | Not reported | Mother’s education (n=720): No education 10 (1.4%), Completed or some primary education 184 (25.6%), Completed or some secondary education 173 (24.0%), Completed or some higher education 353(49.0%). Father’s education (n=720): No education 9 (1.5%), Completed or some primary education 103 (17.1%), Completed or some secondary education 144 (24.0%), Completed or some higher education 345 (57.4%). | Family monthly income level: <=Q1000: N = 207 (28.8%), Q1001-2000: N =155 (21.5%), Q2001-3000: N = 166 (23.1%), Q3001-4000: N = 103 (14.3%), >=Q4001: N = 89 (12.4%) |
| Doyle 2015 | Not reported | Low education = 75/205 (36.6%) | Employed = 78 (38%) |
| El-Mohandes, 2003 | Not reported | Less than high school N=128 (44.8%), High School N=126 (44.1%), Above high school N=13 (8.9%) | **Below poverty level**: N=172 (60.1%), At least 1 household member receiving Medicaid: N=227 (79.4%), At least 1 household member receiving: WIC N=119 (41.6%), and **Employment** (working at time of pregnancy): N=114 (39.9%). WIC indicates the Special Supplemental Nutrition Program for Women, Infants, and Children. |
| Ernsting 2013 | Not reported | Not reported | All are employed but didn't specify their socio economic data |
| Esposito 2018 | Not reported | Not reported | Not reported |
| Fernandez 2022 | Not reported | Less than high school 374 (24.2%), High school or GED 706 (45.6), Post high school 468 (30.2%). | Income: <$10,000 N = 717 (48.6%), $10,000-$20,000: N = 537 (36.4%), and >=$20,000: N = 220 (14.9%). Insurance: No insurance (or CHIP only): N = 850 (54.8%), Public and/or private insurance: N = 700 (45.2%) |
| Ferreira 2022 | 47.5% of the CG considered themselves Catholic and 43.2% of the IG reported being Evangelical. Christian: 104 (65.4%), Other: 54 (34.0%) | Not reported | Most participants in both groups had an income of up to USD 363.63 (Control = 84.2%; Intervention = 68.6%) |
| Fiks 2013 | Not reported | Not reported | Insurance status: Private N = 17903 (80%), Nonprivate N= 4583 (20%) |
| Fitzpatrick 2018 | Not reported | High school or below: 128 (23.0%), Technical school: 181 (32.6%), College: 219 (39.4%), Advanced degree: 29 (5.0%) | Monthly income (USD): <220: N = 149 (26.8%), 220-439: N = 131 (23.6%), 440-732: N = 168 (30.2%), 733-1171: N = 78 (14.0%), and >1172: N = 30 (5.4%). Occupation: student: N = 180 (32.4%), Office worker/white collar: N = 123 (22.1%), service/retail: N = 106 (19.1%), and other: N = 147 (26.4%) |
| Frew 2014 | Not reported | Less than high school = 45 (16.92%), High school or GED= 141 (53.01%), and more than high school= 80 (30.80%) | Annual household income: <$20,000: N = 176 (69.84%), $20,000-$40,000: N = 41 (16.27%), $40,001-$80,000: N = 27 (10.71%), and >$80,000: N = 8 (3.17%). Employment status: Employed: N = 105 (38.89%), Unemployed: N = 150 (55.56%), and other: N = 15 (5.56%) |
| Frew 2016 | Not reported | Total sample= 95; Less than high school N=12 (13%), High school graduate or equivalent (GED) N=45 (47%), Technical/vocational or associates N=29 (31%), Bachelor degree N= 8 (8%), and Graduate degree N=1 (1%) | Currently has health insurance: yes N=87/95 (92%) |
| Galasso, 2023 | Not reported | No High School= 6379 (9%), High school 6379 = (52%), and College = 6379 (39%) | Not reported |
| Gerend 2012 | Catholic: N = 168 (23%), Protestant: N = 95 (13%), Jewish: N = 23 (3%), Muslim: N = 4 (<1%), Buddhist: N = 4 (<1%), Mormon: N = 3 (<1%), Other Christian: N = 275 (37%), None: N = 132 (18%), Other: N = 24 (3%), and Not reported: N = 11 (2%) | College freshman 96 (13%) College sophomore 98 (13%), College junior 176 (24%), College senior 272 (37%), College graduate 35 (5%), Graduate student 62 (8%). Most participants were in their junior or senior year of college (61 %) | Annual household income: $0: N = 118 (16%), $1–$5000: N = 330 (45%), $5001–$10,000: N = 142 (19%), >$10,000: N = 142 (9%), Not reported: N = 7 (<1%).The majority of participants (91 %) had some form of health insurance, with over two thirds covered by their parents’ health insurance |
| Gerend 2021 | Not reported | N=147; Some high school/high school degree/GED= 42 (28.6%), Some college or trade school certificate= 56 (38.1%), College degree= 31(21.1%), and Some graduate school/ graduate degree= 18 (12.2%) | Annual family income: <$20,000: N = 25 (18.4%), $20,000–$39,999: N = 35 (25.7%), $40,000–$59,999: N = 24 (17.6%), $60,000–$79,999: N = 25 (18.4%), ≥$80,000: N = 27 (19.8%). Health Insurance: None: N = 21 (14.4%), parents' insurance: N = 62 (42.5%), personal insurance: N = 63 (43.1%) |
| Glanz 2017 | Not reported | Some college or less: 186 (17.0%); College or higher 905 (82.8%) | Household income equal to $ 80,000 or less: N = 440 (40.3%): More than $80,000: N = 597 (54.6) |
| Glanz 2020 | Not reported | Grade school= 6 (0.7%), High school=19 (2.3%), Some college= 92 (11.2%), College=323 (39.2%), Graduate school= 380 (46.1%), Nonresponse= 4 (0.5%) | Household income:< $40 000: N = 59 (7.16%), $40 000–$80 000: N = 222 (26.94%), $81 000–$120 000: N = 299 (36.29%), $121 000–$150 000: N = 83 (10.07%), $150,000: N =129 (15.7%), Nonresponse: N = 32 (3.9%). Employment: Employed full-time: N = 567 (68.81%), Employed part-time: N = 112 (13.59%), Unemployed: N = 13 (1.58%), Stay-at-home parent: N = 123 (14.93%), Student: N = 8 (0.97%), and Nonresponse: N = 1 (0.12%) |
| Goodman 2015 | Not reported | Total Sample= 105--> 52 control and 53 intervention; Some high school 3.8% (~2/52) in control and 1.9% (~1/53) in the intervention= 3/105 (2.9%), High school 3.8% (~2/52) in control and 11.3% (~6/53) in the intervention= 8/105 (7.6%), some college 15.4% (~8/52) in the control and 25.4% (~13/53) in the intervention= 21/105 (20%), college 42.3 (~22/52) in the control and 37.7% (~20/53) in the intervention= 42/105 (40%), and post college 34.6% (~18/52) in the control and 22.6% (~12/53) in the intervention=30/105 (28.6%). | 94% had private medical insurance (N=105) 86.5% in Control (~45/52) and 92.5% (~49/53) in the intervention |
| Goodyear-Smith 2012 | Not reported | Not reported | Not reported |
| Grandahl 2016 | Not reported | Mother Education level: University 54.3% (325/598), Upper secondary school 39.5%(236/598), and elementary school 6.2% (37/598); Father Education level: University 38.1%(212/557), Upper secondary school 51.5% (287/557), and elementary school 10.4% (58//557) | **Main occupation – mother**: Employed: 88.7% (654/737), Unemployed: 11.3% (83/737); **Main occupation - father**: Employed: 94% (649/691), Unemployed: 6% (42/691). Employed includes studying and/or parental leave. Unemployed includes sick leave and similar |
| Green 2020 | Not reported | Less than HS diploma/GED= 869/2727 (31.9%) | Both parents unemployed= 973/2727 (36%). Financial stress= 2136/2727 (78%) |
| Gurfinkel 2021 | Not reported | Not reported | Not reported |
| Haff 2023 | Not reported | Not reported | Not reported |
| Hambidge 2009 | Not reported | Not reported | >99% with public insurance or uninsured). Self pay at delivery 62/807 (7.7%) |
| Hanley 2023 | Not reported | Not reported | Insurance (N=7408): Medicaid: N = 2654 (35.8%), Private(managed care): N = 4063 (54.8%), uninsured: N = 629 (8.5%), and other: N = 62 (0.1%) |
| Hannan 2013 | Not reported | High school education (n = 96, 69.1%) | Sample size (N=139) - an annual income of less than $20,000/year (n = 100, 71.9%). Most mothers were not employed (n = 76, 55.5%) and most were Medicaid recipients or awaiting coverage by Medicaid (n = 120, 76.3%) |
| Harari 2008 | Not reported | Not reported | Not reported |
| Henrikson 2018 | Not reported | Not reported | Not reported |
| Hess 2013 | Not reported | Not reported. | Not reported. |
| Higginbotham 2012 | Not reported | Not reported. | Not reported |
| Ho 2019 | Not reported | Not reported. | Not reported |
| Hofstetter 2015 | Not reported | Not reported | Insurance (n=2054): Public: N = 1721 (83.8%), Private: N = 76 (3.7%), and uninsured: N = 257(12.5%) |
| Hofstetter 2015a | Not reported | Not reported | Insurance: public 88.5% (4833/5462), Private 5.9% (322/5462), uninsured 5.6% (307/5462). Studies subjects are among urban, low-income, minority children who remained unvaccinated in the late fall |
| Howell-Jones 2023 | Religious denomination of the school (n (%)): Catholic 209 (6.98%), Church of England 895 (29.89%), Other Christian Faith 3 (0.10%), Sikh 3 (0.10%), Other 3 (0.10%), and No religion 1881 (62.83%). | Not reported. | Deprivation (n (%)): High: N = 350 (11.69%), Low: N = 2644 (88.31%). % Eligible for free school meals in the school (mean (SD)) 12.04 (9.51%) |
| Hu 2017 | Not reported | Education level (n=1252): Junior high school or less: 119 (9.5%), Senior high school or technical school: 378 (30.2%), College or above: 755 (60.3%) | Socioeconomic development levels. High: N = 411 (32.8%), Middle: N = 416 (33.2%), Low: N = 425 (33.9%). Occupation: N = No job 113 (9.0%), Farmer/worker/businessman: N = 827(66.0%), Civil servants: N = 239(31.3%), and Medical staff: N = 73 (5.8%) |
| Hu 2018 | Not reported | Maternal education level (n=200): ≤ Primary school: 17 (8.5%), Middle school graduated: 65 (32.5%), Vocational or college graduated: 118 (59.0%) | Employed: N = 151 (75.5%), Unemployed: N = 49 (24.5%) |
| Hu 2021 | Not reported | No formal education/primary 153(47.8%), Secondary level 98(30.6%), and Tertiary level 69(21.6%) | Not reported |
| Huf 2024 | Not reported | Not reported | Index of multiple deprivation (IMD) (mean, SD): for trial arm1=4.3, 2.0; for trial arm 2 =4.3, 2.1; and for trial arm 3= 4.3, 2.0 |
| Hull 2002 | Not reported | Not reported | Not reported |
| Humiston 2014 | Not reported | Not reported | Not reported |
| Hurley 2018 | Not reported | Not reported | Insurance (n=47268): Medicaid: N = 25424 (53.8%), Uninsured: N = 8705 (18.4%), Commercial: N = 3705 (7.8%), and Medicare: N = 8303(17.6%) |
| Hurley 2019 | Not reported | Not reported | Not reported |
| Hurtaud 2023 | Not reported | Not reported | Not reported |
| Hutchinson 2018 | Not reported | Not reported | Not reported |
| Hwang 2010 | Not reported | < High school 70 (6%), High school 898 (71%), Some college 292 (23%) | Housing status: Permanent 52 (4%) and Temporary/streets 1208 (96%). |
| Irigoyen 2006 | Not reported | Not reported | Not reported |
| Isrctn, 2021 | Not reported | Not reported | Not reported |
| Jackson 2011 | Not reported | Left school at 16 years 34.5% (49/142), Left school at 18 years 14.1% (20/142), and Achieved Degree or higher 54.4% (73/142) | Mean Low Income Scheme Index score: 14.8% (21/142). Low Income Scheme Index score is based on the percentage of prescribed items exempt from a prescription charge due to low income of the patient |
| Jacobson 2022 | Not reported | 8.3% of respondents have less than a high school degree. | The median self-reported household income was between $20,000 to $29,999 |
| Janitz 2023 | Not reported | Not reported | Not reported |
| Jiang 2022 | Not reported | n=350: Primary school or below 126 (36%), High school 164(46.9%), and College or above 60(17.1%) | Monthly income (Chinese Yuan): ≤1,000=176 (50.3%), 1,000-4,000: N = 116 (33.1%), and ≥4,000: N = 58 (16.6%). Occupation: Retirement: N = 160 (45.7%), Full-time/part-time job: N = 33 (9.4%), and other: N = 157(44.9%) |
| Johansen 2023 | Not reported | Not reported | Not reported |
| Johnson 2003 | Not reported | Not reported | Not reported |
| Jordan 2015 | Not reported | Not reported | Poverty group: <=20% poverty 66.8% (4513/6758), > 20% poverty 33.2% (2245/6758). Poverty group (<20% poverty, >20% poverty) was assigned by matching participant’s ZIP code with ZIP code–level poverty data available through the American Community Survey (2011) |
| Joseph 2016 | Practice religion (n=152), Catholic 19% (36/193), Protestant 43% 84/193), N/A( not available) 21% (40/193), Other 17% (33/193) | Maternal highest level of education: Less than/some high school 21% (41/197), Completed high school 35% (67/193), College 41% (80/193), Master's and beyond 3% (5/193) | Average household income <$20 000: 45% (77/171); $200 000-35 000: 29%(50/171); $35 000-60 000: 19% (33/171); >$60 000: 6% (11/171) |
| Ju, 2024 | Not reported | n= 1258: Junior high school and below 21(1.7%), Senior high school (including technical secondary school) 123(9.8%), University (including junior college) 840(66.8%), and Postgraduate and above 274(21.8%) | Worker: 573 (45.5%) |
| Juon 2016 | Not reported | <High school 31 (13.4%), High school Plus 85 (36.8%), College graduate 67 (29.0%), and Grad school 48 (20.8%) | Employed: N = 157 (67.9%), Not employed: N = 74 (32.1%). Having health insurance: yes: N = 212 (52.8%) and no: N = 108 (47.2%) |
| Juraskova 2011 | N=159: Christian N = 104 (66%) and other N = 52(34%) | Father’s education: <12 years 40(25%), TAFE/diploma 17(11%), and University or higher degree 102 (64%) | Not reported |
| Kasting 2019 | Not reported | Not reported | Not reported |
| Kempe 2001 | Not reported | Not reported | Not reported |
| Kempe 2005 | Not reported | Parent/guardian education (n=5193): High school graduate or less 950(18.3%), Some college 1182(22.8%), and College graduate or more 3060(58.9%) | Annual household income (n=5193): <$50 000: N = 1795 (34.6%), $50 000 to <$100 000: N = 1935 (37.3%), and >=$100 000: N = 1482 (28.5%). Insurance: Private 80.3% in the intervention group and 82.1% in the control group; public: 16.0% in the intervention group and 14.8% in the control group; uninsured: 3.4% in the intervention group and 3.0% in the control group |
| Kempe 2012 | Not reported | All students are six grade. | All schools serve predominately low-income, minority populations |
| Kempe 2016 | Not reported | Not reported | The study population was insured and lived in census tracts with a median family income of $74900 (SD $32420), as assessed by using geocoding to estimate area-based socioeconomic measures |
| Kempe 2020 | Not reported | Not reported | Not reported |
| Kerpelman 2000 | Not reported | Not reported | The study was conducted among low-income preschool children by imposing a sanction on families who failed to provide proof of up-to-date immunization status |
| Khan 2023 | Not reported | Not reported | Not reported |
| Kim 2020 | Religion (n=103): Buddhist N = 4 (3.9%), Catholic N = 31 (30.1%), Protestant N = 47 (45.6%), and none N = 21 (20.4%) | All are College Students | Not reported |
| Kopfer 2012 | Not reported | Year in College (N=404)፡ Junior 30% (122), Senior 24% (95), Sophomore 19% (77), Graduate student 16% (63), First Year 11% (44) | Health Insurance Coverage: Yes 90% (365), No 85 (32), and Didn’t know 1% (4) |
| Krieger 2000 | Not reported | N= 1246 (Intervention group=622, and control=624); less than high school 16.3% in the intervention and 17.1% in the control, high school grad 28.2% in the intervention and 30.0% in the control, some college 24.3% in the intervention and 22.0% in the control, college grad 31.1% in the intervention and 30.9% in the control | Household income: <$10,000 was 15.1% in the intervention and 18.3% in the control |
| Kulle, 2024 | Not reported | Not reported | Not reported |
| Larsen, 2023 | Not reported | Not reported | Not reported |
| Lau 2012 | Not reported | University student = 316 (80.2%) | Not reported |
| LeBaron 2004 | Not reported | Not reported | Not reported |
| Lee 2020 | Not reported | Not reported | There are income data by intervention and control group but difficult to extract the overall percentage: $10K-29K~ 26% in the intervention and 24% in the control; $30K-49K~ 21.5% in the intervention and 20.5% in the control; $50K-69K ~23% in the intervention and 22% in the control, and $>70K ~23.% in the intervention and 23% in the control |
| Lerner 2021 | Not reported | Not reported | Insurance, n (%): Private 20 208 (91.7%), Public 1517 (6.9%), and Other or unknown 321 (1.5%) |
| Leung 2017 | Not reported | Baseline--->Below primary/elementary (Grade 1-6)= 29.5% (156/529), Primary/elementary or above=70.5% (373/529): NB in the regression table---> Tertiary education 8.6% (32/374), Secondary education (middle school/high school/Grades 7–12) 32.1% (120/374), Primary 31.0% (116/374), and below primary 28.3% (106/374) | Not reported |
| Liao 2020 | Not reported | Secondary or below= 124/291 (42.6%) and Tertiary or above= 167/291 (57.4%) | Income US$: <20,000=30/291 (10.3%), 20,000-40,000=119/291 (40.9%), and ≥40,000=152/291 (52.2%) |
| Lieu 2022 | Not reported | Not reported | Neighbourhood Deprivation Index, percentile (N = 8287): <25th (least deprived): N = 1396 (16.8%), 25th-74th: N = 3561 (43.0%), 75th-89th: N = 1566 (18.9%), ≥90th (most deprived): N = 1281 (15.5%), and Unknown: N = 483 (5.8%) |
| Lin 2020 | Not reported | Education (n=180): High school 37 (20.6%), Junior college 23 (12.8%), University 87 (48.3%), and Graduate school 33 (18.3%) | Occupation: Have work: N = 127 (70.6%), No work: N = 40 (22.2%), Stopped working: N = 10 (5.6%), and Other: N = 3 (1.7%) |
| Ma 2018 | Not reported | Education (n=1720): <High school=167 (9.7%), High school graduate=490(28.5%), and >Some college=1063 (61.8%) | Annual household income (n=1527): <$20,000: N = 471 (30.8%), $20,000-$40,000: N = 544 (35.6%), >$40,000: N = 512 (33.5%). Health insurance status (n=1753): No=830 (47.3%), Yes=923 (52.7%). Employment (n=1755): Employed=1026 (58.5%), and Unemployed/retired/homemaker= 729 (41.5%) |
| Ma 2021 | Not reported | Not reported | Not reported |
| Mantzari 2015 | Not reported | Not reported | Data provided on mean and standard deviation (SD) social deprivation (IMD): First time invitees Intervention group = M = 46.3 (13.12), First time invitees control group = M = 45.3 (13.0), Previous nonattenders intervention group = M = 35.3 (21.9), and Previous nonattenders control group= M = 36.2 (22.2). Note: Social deprivation. Area-level social deprivation was measured using participants’ postcodes to calculate English Index of Multiple Deprivation (IMD) scores, which range from 0.37 (least deprived) to 85.46 (most deprived) (Community & Neighbourhoods, 2007). The IMD is a measure of deprivation in England based on area of residence |
| Marra 2014 | Not reported | Elementary school: N = 196 (16.5), High school 614 (51.6), College or university: N = 378 (31.7), Currently a student: N = 3 (0.2) | <$14,999: N = 113 (10.8%), $15,000-$29,999: N = 317 (30.4%), $30,000-$44,999: N = 199 (19.1%), $45,000-$59,999: N = 151 (14.5%), $60,000-$79,999: N = 89 (8.5%), $80,000-$99,999: N = 51 (4.89%), ≥$100,000: N = 37 (3.6%) |
| Marra 2014 | Not reported | Elementary school: N = 169 (8.7%) High school: N =1091 (56.2%), College or university: N = 655 (33.8%) Currently a student: N = 25 (1.3%) | <$14,999: N = 226 (15.3%), $15,000-$29,999: N = 372 (25.2%), $30,000-$44,999: N = 300 (20.3%), $45,000-$59,999: N = 230 (15.6%), $60,000-$79,999: N = 164 (11.1%), $80,000-$99,999: N = 104 (7.0%), ≥$100,000: N = 37 83 (5.5%) |
| Mason 2000 | Not reported | Not reported | Not reported |
| Masson 2013 | Not reported | High school education or above 269/489 (55.0%). | Employed: N = 78 (15.9%). Yearly income < $10 000: N = 298 (60.9%) |
| McCaul 2002 | Not reported | Not reported | Not reported |
| Meharry 2014 | Not reported | N=133: <HS/GED 55 (41.3%), Some college 32 (24.1%), College degree 25 (18.8%), and >Graduate degree 19 (14.3%) | Income: <$25,000 = 44 (33.1%), $25,001–$50,000 = 13 (9.8%), $50,001–$75,000 = 6 (4.5%), >$75,001 = 24 (18.0%), and Did not disclose = 46 (34.6%). Employment: Full-time = 40 (30.1%), Part-time = 28 (21.1%), Not working = 46 (34.6%), and ‘‘Stay-at-home-mom’’ = 16 (12.0%) |
| Mehta 2022 | Not reported | Not reported | Household income: < $35,000 / Unknown: N =4396 (27.4%), $35,000 – 59,999: N = 4050 (25.2%), $60,000 – 76,099: N = 3092 (19.3%), and ≥ $76,100: N = 4507 (28.1%); (median (IQR), $): 57 126 (34 579-76 103). *American Community Survey (2015-2019) Median Household Income at zip code level in 2019 inflation-adjusted dollars. Data were missing for 25 participants.* |
| Menzies 2020 | Not reported | Not reported | Not reported |
| Milkman 2022 | Not reported | Not reported | Median household income (US$): 47,474 for Philadelphia County and 38,808 for all 20 zip codes eligible for treatment. |
| Milkman 2022a | Not reported | Not reported | All participants were Medicare recipients. |
| Moniz 2013 | Not reported | n=204: Less than high school 19 (9.3%), High school or high school equivalency certificate 145 (71.1%), 2-y to 4-y college 39 (19.1%), and Postgraduate degree 1 (0.5%) | Household income: Less than $10,000 = 124 (60.8%), $10,000–40,000 = 55 (27%), $40,000–100,000 = 11 (5.4%), Greater than $100,000 = 4 (2%), Do not know = 10(4.9%). Insurance: None = 31 (15.2%), Medicaid or Medicare = 149 (73%), Private = 22 (10.8%), and Do not know: 2 (1%) |
| Munoz-Miralles 2022 | Not reported | Not reported | Not reported |
| NCT, 2022 | Not reported | Not reported | Not reported |
| NCT05012163 2024 | Not reported | Not reported | Not reported |
| NCT05248399, 2022 | Not reported | Not reported | Not reported |
| NCT05534061 2022 | Not reported | Not reported | Not reported |
| NCT05537441 2022 | Not reported | Not reported | Not reported |
| Nehme 2019 | Not reported | Not reported | Study subjects are members of an Affordable Care Act insurance plan. |
| Nowalk 2010 | Not reported. | Not reported | All study subjects are employees of companies. |
| Nyamathi 2009 | Not reported | N=865: High school graduate 73.9% | Employed 9.4% |
| Nyamathi 2010 | Not reported | N=148: High School Grad 81.5%. | Employed 16.7% |
| O'Grady 2018 | Not reported | N=310: Mothers education status: Tertiary degree 43(13.9%), Diploma/certificate/trade 124(40%), High school 67(21.6%), Did not finish high school 67(21.6%), and Declined/missing/unknown 10 (3.2%). Elementary school (16.5%), High school (51.6%), College or University (31.7%), Currently a student (0.2%) | Total annual household income: $104,000+: N = 55 (17.7%), $78,000–103,999: N =52 (16.8%), $52,000–77,999: N = 49 (15.8%), $0–51,999: N = 81 (26.1%), and Declined/missing/unknown= 73(23.5%) |
| O'Grady 2022 | Not reported | n=310: Mothers education status: Tertiary degree 43(13.9%), Diploma/certificate/trade 124(40%), High school 67(21.6%), Did not finish high school 67(21.6%), and Declined/missing/unknown 10 (3.2%). | Total annual household income: $104,000+: N = 55 (17.7%), $78,000–103,999: N = 52 (16.8%), $52,000–77,999: N = 49 (15.8%), $0–51,999: N = 81 (26.1%), and Declined/missing/unknown: N = 73 (23.5%) |
| O'Leary 2015 | Not reported | Not reported | Not reported |
| O'Leary 2019 | Not reported | High school or less (50.7%), Technical, vocational or 2-year degree (20.8%), 4-year college or more (28.5%) | Not reported |
| Omer 2022 | Not reported | N = 2,092: Doctoral or professional degree 140 (8.1%), Master’s degree 326 (18.8%), Bachelor’s degree 616 (35.5%), Associate’s degree 166 (9.6%), Postsecondary non-degree award 68 (3.9%), Some college, no degree 219 (12.6%),High school diploma or equivalent 182 (10.5%), No formal education credential 12 (0.7%), Prefer not to answer 5 (0.3%), and Missing 358 (17.1%). | Insurance type: Private: N = 1,435 (70.8%), Medicaid/CHP+: N = 476 (23.5%), Medicare: N = 7 (0.4%), Uninsured: N = 36 (1.8%), Other: N = 41 (2.0%), Unknown: N = 33 (1.6%), and Missing: N = 64 (3.0%) |
| Osborne, 2023 | There is religious data but not clear to extract | School year (n=702), 1st =224 (31.9%), 2nd=142 (20.2) , 3rd=170 (24.2) , 4th=120 (17.1%) , 5th+=33 (4.7%), missing= 13 (1.8%) | Not reported |
| Otsuka 2013a | Not reported | Not reported | Insurance (n=674): Private: N = 317(47%), Medicare: N = 339 (50.3%), Medicaid: N = 10 (1.5%), and self-pay/other: N = 8 (1.2%). |
| Otsuka-Ono 2019 | Not reported | Mother’s highest education level completed (n=171): Middle/high school 21 (12.3%), Vocational school 41 (24%), Junior college 40(23.4%), university 65 (38%), Graduate 4 (2.3%) | Annual income (thousand yen): < 2000 = 2 (1.2%), 2000–3999 = 19 (11.1%), 4000–5999 = 66 (38.6%), 6000–7999 = 41 (24%), 8000–9999 = 27 (15.8%), and ≥ 10,000 = 13 (7.6%). Mother job status: Unemployed: 77 (45%), Full-time job: 71 (41.5%), part-time job: 22 (12.9%). Father job status: Full-time job: 165 (96.5%), self-employed: 6 (3.5%) |
| Patel 2012 | Not reported | Student standing (n = 256): Undergraduate 181 (70.7%), Graduate 70 (27.3%), Missing 5 (2.0%) | All students pay a student health insurance fee as part of tuition that covers a broad range of health care services at the university-based clinic (excludes vaccinations). Supplemental health insurance coverage: None: N = 84 (32.8%), Parent’s private insurance: N = 58 (22.7%), Student’s own private insurance: N = 31 (12.1%), Other: N = 70 (27.3%), Missing: N = 13 (5.1%) |
| Patel 2014 | Not reported | N = 365: High school or less 185 (50.7%), Technical-vocational or 2-year degree 76 (20.8%), and 4-year college or more 104 (28.5%) | Health insurance status (N=365): No health insurance: N = 207 (56.7%), Private insurance: N = 100 (27.4%), and Public insurance: N = 58 (15.9%) |
| Patel 2022 | Not reported | Not reported | Not reported |
| Payakachat 2016 | Not reported | Education y (n=279): Less than 12th grade 14.7%, GED or High school graduate 39.1%, Some Technical or Community College 18.3%, and Graduated Technical or Community College or higher 38.9% | Annual household income (n=279): <$20,000 = 64.5%, ≥$20,000 = 35.5%. Insurance status: Health Insurance from job/spouse’s job/parents job = 13.3%, Health Insurance someone else paid for (not job-related) = 4.6%, Medicaid = 73.5%, No Health Insurance = 8.7% |
| Porter-Jones 2009 | Not reported | Not reported | Not reported |
| Pot 2017 | (N=8062): Protestant=1490 (18.48%), Not protestant =6559 (81.36%); N missing=13 (0.16%) | Low=1128 (13.99%), Middle=3471 (43.05), and High=3446 (42.74%); Missing=7 (0.09%). Educational level was classified into low (less than secondary or vocational education), intermediate (secondary through preuniversity education) or high (professional or university education) | Not reported |
| Qin 2023 | Not reported | N=100: Below college= 30(30.0%) and College and above =70(70.0%) | Annual income (US$) : 4539$: N = 20 (20.0%), 4539–12103$: N = 22 (22.0%), 12104–22693$: N = 32 (32.0%), 22694–45386$: N = 17 (17.0%), and 45386$: N = 9 (9.0%) |
| Quinlivan 2003 | Not reported | Not reported | Socioeconomic status score (% low or destitute score) = 117/136 (86.0%), Homeless (yes) = 20/136 (14.7%) |
| Rabb 2022 | Not reported | Not reported | Not reported |
| Rand 2015 | Not reported | Not reported | Insurance (N = 3812): Medicaid: N = 2241 (58.8%), SCHIP N = 1571 (41.2%) |
| Rand 2017 | Not reported. | Not reported | Insurance: Public: N = 602 (80.4%), Private: N = 103 (13.7%), and None: N = 44 (5.9%) |
| Reiter 2018 | Not reported | Education level (n=150): Some college or less 94 (62.7%) and College degree or more 56 (37.3%) | Household income: Less than $50,000: N = 114 (76%) and $50,000 or more: N = 36 (24%). Health insurance: None: N = 27 (18%), On parents’ insurance: N = 67 (44.7%, Insures self: N = 56 (37.3%) |
| Reiter 2023 | Not reported | (n = 1,227): High school or less=380 (31%) and Some college or more= 847 (69%) | Employment status: Employed full time or part time: N = 349 (28%), and Other: N = 878 (72%). Health insurance: Private: N = 768 (63%), public: N = 206 (17%), and None/don’t know: N = 253 (21%) |
| Richman 2014 | Not reported | Not reported | Employed N = 139/250 (56%). Insured N = 176/252 (70%) |
| Richman 2016 | Not reported | Class standing (n=263): First year 75 (28.5%), Sophomore 53 (20.1%), Junior 44(16.7%), Senior 58(22.0%), and Graduate student 33(12.5%) | Not reported |
| Rickert 2015 | Not reported | n = 445: Parental Education: < High school 62 (14.0%), High school or GED 104 (23.5%), and Some or college degree 277(62.5%) | Insurance type: None: N = 75(16.9%), Private: N = 205(46.1%), and Public (Medicaid/CHIPS): N = 164(36.9%). |
| Roca 2012 | Not reported | Not reported | Not reported |
| Rodriguez 2022 | Not reported | Not reported | Health insurance provider: City-based health care service: N = 28 (5.6%), Health maintenance organization/Kaiser: N = 7 (1.4%), Medicaid: N = 173 (34.8%), Medicare: N = 97 (19.6%), None: N = 95 (19.2%), Affordable Care Act: N = 21 (4.2%), Private: N = 103 (20.8%), Other: N = 23 (4.6%), and Veterans Health Administration: N = 5 (1.0%). Housing status: Housed: N = 408 (82.3%), Marginal: N = 19 (3.8%), and Unhoused: N = 68 (13.7%) |
| Rodriguez 2024 | Not reported | Not reported | Self-reported health insurance coverage (Participants could have more than one selection) n=767: City-based health care service: N = 68 (8.9%), HMO/Kaiser: N = 3 (0.4%), Medicaid: N = 193 (25.2%), Medicare: N = 146 (19.0%), None: N = 126 (16.4%), Affordable Care Act: N = 7 (0.9%), Private: N = 289 (37.7%), Other: N = 50 (6.5%), and Veterans Health Administration: N = 16 (2.1%). Housing status: Housed: N = 695 (90.6%), Marginal: N = 22 (2.9%), Unhoused: N = 50 (6.5%). |
| Ronzani 2022 | Not reported | Not reported | Not reported |
| Saaksvuori 2022 | Not reported | Not reported | Not reported |
| Saccardo 2024 | Not reported | Not reported | Not reported |
| Saitoh 2017 | Not reported | n=188: Middle/high school 47 (25%), Junior college 94(50%) , and College/graduate school 47 (25%) | Household annual income (thousands of yen) (n=150): 0–3000: N= 16 (10.7%), 3000–4999: N = 70 (46.7%), 5000–6999: N = 48 (32%), 7000–9999: N = 17 (11.3%), and >=10,000: N =8 (5.3%). Maternal employment status (n=188): Unemployed: N = 133 (70.7%) and Employed: N = 55(29.3%) |
| Santa Maria 2021 | Not reported | Parent: Did Not Finish High School 96 (18.71%), High School Graduate 121 (23.59%), Vocational/Technical 66 (12.87%), Some College 119 (23.20%), and College Graduate 111 (21.64%. Youth attended school yes 498 (99.40%) and their current grade are from 4th to 9th | Parent Insurance Status: None: N = 81 (15.70%), Medicaid: N = 217 (42.05%), Private: N = 168 (32.56%), and Other: N = 50 (9.69%) |
| Scarinci 2020 | Not reported | Mean education (years) – Mother 8.7 (3.5) for the vaccination arm and 8.9 (2.5) for the control arm. | Mean Monthly Income ((USD): 1,775.34 (1068.4) for the intervention arm and 1,549.64 (553.2) for the control arm. Employment Status-Mother (n=278): Full-time: N = 51 (18.3%), part-time: N = 87 (31.3%), homemaker: N = 130(46.8%), and unemployed: N = 10 (3.6%) |
| Schwarz 2008 | Not reported | Caregivers (n=76): Not educated past 11th grade 31 (40.8%) | Caregivers (n=76): Employed: N = 9 (11.8%), Financial assistance: N = 66 (86.8%), and Income ≤0K: N = 47(61.8%) |
| Scott, 2019 | Not reported | Parent education high school or less 202/400 (50.5%) | Child Public insurance= 386/400 (96.5%) |
| Shegog 2022 | Not reported | Parent College degree or more (n=373): No 143 (38.1%), and Yes 232 (61.9%) | Parent Employed (n=2375): No: N = 119 (31.7%), Yes: N = 256 (68.3%). Parent Insurance: Private health insurance (individual or employer provided): N = 287 (76.5%), Medicaid/Medicare/CHIP/S-CHIP: N = 77 (20.5%), Military Health Care (Tricare/VA/CHAMP-VA): N = 2 (0.5%), No insurance: N = 7 (1.9%), and Other: N = 2 (0.5%) |
| Shen, 2024 | Not reported | Education (middle school or higher): 193/720(26.8%) | Income (>CNY 2000): 506/720(70.3%) |
| Shourie 2013 | Not reported | N=220: Up to 18 years 85 (38.6%), beyond 18 years 134 (60.9%), and missing 1 (0.5%) | Employment: Full time: N = 107 (48.6%), part-time: N = 75 (34.1%), other: N = 36 (16.4%), and missing: N = 2 (0.9%) |
| Si 2022 | Not reported | Parental education (n = 3739): Junior high school or below 1682 (44.99%),Senior high school (including vocational high school) 1114 (29.79%), and College (including technical college) and above 943 (25.22%) | Not reported |
| Sitler 2018 | Not reported | Caregiver educations (n=129): HS or less= 71.7%, some college= 21.1%, and college degree 7.2%. | Study population: rural low-income population of children enrolled in a WIC (Women/Infant/ Children) program. Eligibility for enrolment into this program is based upon income with the maximum income allowance of 185% above poverty |
| Skinner 2000 | Not reported | Study was conducted among secondary schools | Living expenses (RMB/month): <1000 Yuan: N = 767 (20.51%), 1000–2000 Yuan: N = 2661 (71.17%), and >2000 Yuan: N = 311 (8.32%) |
| Song 2000 | Not reported | Not reported | Not reported |
| Stockwell 2012 | Not reported | Not reported | Insurance (n=9213): None: N = 622 (6.8%), Medicaid/SCHIP: N = 8112 (88.0%), and private: N = 479 (5.2%). Study subjects are low-income, urban parents |
| Stockwell 2012 | Not reported | Not reported | Insurance status for Text4Health–Adolescents (n=361): Uninsured 41(11.4%), Medicaid/SCHIP 290(80.3%), and private 30 (8.3%). The study populations are an urban, low-income population |
| Stockwell 2012 | Not reported | Not reported | Insurance status for Text4Health–Peds (n=174): Uninsured 29(16.7%), Medicaid/SCHIP 132(75.9%), private 13(7.5%). The study populations are an urban, low-income population |
| Stockwell 2014 | Not reported | Not reported | N = 1153: Insurance at start of pregnancy: Uninsured: N = 360 (31.2%), Medicaid/SCHIP: N = 781 (67.7%), and Private: N = 12 (1.1%). The study population are low-income obstetric population |
| Stockwell 2015 | Not reported | <High school 110 (16.7%), High school 230 (34.8%), and At least some college 320 (48.5%) | Insurance: Medicaid/State Children’s Health Insurance Program: N = 638 (96.7%), Commercial: N = 14 (2.1%), and Uninsured: N = 8 (1.2%). The study populations are low-income, urban, minority population |
| Stockwell 2022 | Not reported | Caregiver education: High school or less 22.3% (320), Vocational school or some college 17.9% (257), Associates or bachelors 36.3% (522), and Masters or doctorate 23.5% (338) | Child insurance type: Commercial insurance 54.5% (N = 1137) and Public insurance or uninsured 45.5% (N = 948) |
| Stolpe 2019 | Not reported | Education in ZIP code, % undergrad or higher (SD) (n=21971; 10962 control and 11009 intervention): 26.8% (12.5) for control and 26.6% (12.3) for the intervention | Median income in ZIP code, mean (SD), $: 67,079 (17,034) for the control and 67,019 (17,135) for the intervention |
| Strathdee 2023 | Not reported | (n=135): Median years of education completed (IQR)=12(10,13) | Monthly income <500 USD: N = 64 (47.4%). Income worse since COVID-19 pandemic began: N = 93 (68.9%). Homeless: N = 99 (73.3%). Lacks health insurance: N = 18 (13.3%) |
| Stuck 2015 | N=2284: Protestant 1196 (52.4%), Catholic 1115 (48.8%), No religious affiliations 48 (2.1%), and other/unknown 105(4.6%) | n=2284: Compulsory education or less (<=9 y) 994(43.5%), Secondary-level education (10–12 y) 1042 (45.6%), Tertiary-level education (>12 y) 194(8.5%), and unknown 54(2.4%) | Socio-economic status- Swiss neighbourhood index: 61.2 ± 7.3 for the intervention group and 60.8 ± 7.4 for the control group. Higher scores denote higher levels of socio-economic status |
| Suh 2012 | Not reported | Not reported | Insurance status (n = 799): Private 89.6% (717), public 8.1% (65), and missing 2.3% (18) |
| Suzuki, 2022 | Not reported | N= 2175: Less than high school graduate 21 (1.4%), High school graduate 355 (22.9%), and More than high school graduate 1174 (75.7%) | Household income (million JPY/year), Mean (SD)= 7.41 (4.68); JPY 110=US $1 USD |
| Sweeney 2014 | Not reported | Participants were undergraduate students. | Not reported |
| Szilagyi 2006 | Not Reported. | Not reported | Insurance (n=3006): Medicaid, fee for service: N = 709 (26.3%), Medicaid, managed care (includes SCHIP): N = 289 (9.6%), Private, fee for service: N = 166 (5.5%), Private, managed care: N = 1302 (43.3%), Uninsured: N = 84 (2.8%), and other or unknown: N = 307 (10.2%) |
| Szilagyi 2011 | Not reported. | Not reported | Insurance type (n=7549): Medicaid managed care: N = 4935 (65.4%), Medicaid fee for service: N = 626 (8.3%), Uninsured: N = 445 (5.9%), SCHIP/State Children’s Health Insurance Program: N = 989 (13.1%), and Commercial: N = 550 (7.3%). Study area population: Almost 80% of adolescents in the city live below the poverty level |
| Szilagyi 2013 | Not reported | Not reported | Insurance (n = 4115): Medicaid managed care: N = 1916 (46.6%) and SCHIP: N = 2199 (53.4%). Study subjects are low-income adolescents |
| Szilagyi 2018 | Not reported | The study subjects are elementary school children | Not reported |
| Szilagyi 2019 | Not reported | The study subjects are elementary school children | Participants are urban (mostly low-income income: ~90% free and reduced lunch) elementary school children |
| Szilagyi 2020 | Not reported | Not reported | Not reported |
| Szilagyi 2020 | Not reported | Not reported | Insurance (n=164,205): Private 85.2%, Public 13.5%, and Other or unknown 1.3%. |
| Szilagyi 2020 | Not reported | Not reported | Not reported |
| Szilagyi 2022 | Not reported | Not reported. | Primary insurer (n=196,486): private: N = 167009 (85%), public: N = 26391 (13.4%), and other/unknown: N = 3086 (1.6%) |
| Szilagyi 2023 | Not reported | Not reported | Insurance (N = 213,773): Private: N = 181,724 (85.0%), Public: N = 28,545 (13.4%), and Other/unknown: N = 3504 (1.6%) |
| Szilagyi 2024 | Not reported | Not reported | Primary insurer: Private 218728 (83.4%), public 39008(14.9%), other or unknown 4349 (1.7%). |
| Tentori 2022 | Not reported | Not reported | Not reported |
| Terrell-Perica 2001 | Not reported | Not reported. | Not reported |
| Thilly, 2024 | Not reported | Not reported | Not reported |
| Tiro 2015 | Not reported | Not reported | Insurance: (n = 814): Public 603 (74.1%), private 16 (2.0%), and No insurance 195 (24.0%) |
| Topp 2013 | Not reported | Completed four or more years of high school (n=139); 60% | Government benefits main source of income = 86%, Unstable accommodation last six months = 37% |
| Tubiana 2021 | Not reported | Not reported | Not reported |
| Tull 2019 | Not reported | All study subjects are year 7 students. | Not reported. |
| Ueberroth 2022 | Not reported | Not reported | Not reported |
| Underwood 2015 | Not reported | N=686: Middle school 436 (63.6%) and High school 250 (36.4%) | Child’s insurance: Private insurance: N = 233 (34.0%), Medicaid: N = 410 (59.8%), and No insurance: N = 42 (6.1%) |
| Usami 2009 | Not reported | Not reported | Not reported |
| Vanderpool 2013 | Not reported | N=345: 48.0% reported some college as their highest level of education | Only one-quarter (25.6%) were employed full-time |
| Viver 2000 | Not reported | Pre-school children | Not reported |
| Wagner 2021 | Not reported | From 6 interviewed caregivers for qualitative: some had college (50% bachelor’s degree, 50% some college or associate’s degree) | Insurance at baseline (n=142): Medicaid: N = 71 (50%), Commercial: N = 64 (45.1%), and Others: N = 7(4.9%). |
| Wang 2021 | Not reported | N = 624: Secondary or below 95 (15.2%) and University or above 529 (84.8%) | **Current employment status:** Full time: N = 492 (78.8%), Part-time/unemployed/retired/students: N = 132 (21.2%). **Personal monthly income** (HK $; US **$)**: <HK $10,000 (US $1290): N = 101(16.2%), HK $10,000-$19,999 (US$1290-$2580): N = 207 (33.2%), HK $20,000-$39,999 (US$2580-$5161): N = 223 (35.7%), ≥HK $40,000 (US $5161): N =90 (14.4%), Refuse to disclose: N = 3 (0.5%). |
| Wang 2023 | Not reported | (N=396): ≤Primary 164 (41.4%), Secondary 189 (47.7%), and ≥Tertiary 43 (10.9%) | Monthly household income, HK$ (US $): <20 000 (2580) 294 (74.2%), ≥20 000 (2580) 52 (13.2%), and Refuse to disclose 50 (12.6%) |
| Weaver 2014 | Not reported | Not reported | **Employment (n=210):**  Unemployed: N = 183 (87%), Employed or student: N = 25 (12%), and other: N = 2 (1%). **Accommodation:** Owner occupied: N = 11 (5%), Rented private: N = 47 (22%), Rented (LA, HA): N = 72 (34%), Living with relatives: N = 17 (8%), Bed and breakfast or hotel: N = 5 (2%), Hostel: N = 21 (10%), NFA: N = 26 (12%), and other: N = 11 (5%). |
| Wijesundara 2020 | Not reported | Not reported | Not reported |
| Wilcox 2001 | Not reported | Mother’s education (n=1752): High school 62.5% and Less than high school 37.5%. | The median household income of the area served by the university nursing center was $23365, and the median income of the area served by the social services agency was $15291. |
| Winston 2007 | Not reported | There is data for educational level but there is not enough information to extract | Not reported |
| Wiseman 2016 | Not reported | parent education (n=136): Elementary 9 (7%), High school 102 (75%), Some college 16 (12%), College/university 5 (3.7%). | Family Income: Less than $10,000: N = 63 (51%), $10,001-25,000: N = 43 (35%), $25,001-$40,000: N = 9 (7%), $40,000 and above: N = 8 (7%). |
| Wong 2016 | Not reported | Maternal education (n=321): Compulsory secondary or below 23 (7.2%), Upper secondary 53 (41.1%), Some post-secondary 30 (9.4%),and University degree or above 136 (42.4%) | Family income: Below median: N = 93 (29.0%) and Above median: N = 228 (71.0%)---->Median household income in HK in 2011 was $20,000 to $24,999 HKD per month (1USD=7.7HKD). |
| Wootton 2022 | Not reported | Not reported | Not reported |
| Wouters, 2007 | Not reported | Not reported | All are commercial sex workers (CSW). Working sector - Street 21% (N = 127), Bar 18% (N = 112), Window 31% (N = 190), Private 26% (N = 157), Missing 5% (N =29) |
| Wright 2012 | Not reported | Not reported | Insurance (n=3,979): private: N = 3369 (84.7%), Medicare: N = 562 (14.15%), Medicaid/free care: N = 14 (0.4%), self-pay/none: N = 31 (0.8%), and unknown: N = 3(0.1%). Lived in higher income neighbourhoods (median: $54,617 versus $52,012) and the Mean median neighbourhood income (SD): was 55,385 (9,748) for the active control arm and 54,024 (10,406) for the intervention arm. |
| Wynn 2021 | Not reported | Parental education (n=956): <High school 219 (22.9%), Finished high school 354 (37.1%), and >High school 382 (40%) | Insurance (n=956): Public: N = 903 (94.5%) |
| Xu 2022 | Not reported | n=196: Primary and below 27(13.8%), Middle or high school 104(53.1%), college 41(20.9%), and Bachelor degree or above 24(12.2%) | Occupation: Teacher: N = 4 (2.0%), Service worker: N = 17(8.7%), Medical worker: N = 8 (4.1%), Worker or Farmer: N = 40 (20.4%), Government employee: N = 12(6.1%), Housework and unemployment: N = 74 (37.8%), and other: N = 41 (20.9%) |
| Yeung 2018 | Not reported | Maternal highest educational level (n=833)፡ Junior secondary or below 72 (8.6%), Senior secondary 308(37%), Post-secondary or matriculation 188 (22.6%), University degree or above 265 (31.8%) Low: N = 1128 (13.99%); Middle: N = 3471 (43.05%); High: N = 3445 (42.74%) | Monthly household income in $ (n=820): <20,000: N =175 (21.3%), 20,000–29,999: N = 188 (22.9%), 30,000–39,999: N = 191 (23.3%), >40,000: N = 266 (32.4%) |
| Yokum 2018 | Not reported | Not reported | Not reported |
| Yudin 2016 | Not reported | Education (N = 277): post-secondary 87.7% | Household income > $100,000.00 [N = 255]: 112 (43.9%). |
| Yue 2020 | Not reported | Below primary education 106 (28%), Secondary education 213 (57%), and Tertiary education 54 (15%). | Housing Type: Public housing (small): N = 98 (26%), Public housing (large): N = 215 (58%), and Private housing: N = 60 (16%). Working status - Yes: N = 114 (31%) and No: N = 259 (69%) |
| Zhang 2018a | Not reported | Not reported | 60.53% spent at least a week in the prior month living on the street. In shelter ≥ 1 week: N = 13.53 |
| Zhang 2018b | Not reported | Highest education (n=311): No school education 70(22.5%), Primary school 123(39.5%), Junior high school 73(23.5%), Senior high school 29(9.3%), college graduation 9(2.9%), University graduation and above 5(1.6%), and other 2(0.6%). | Not reported |
| Zhang 2022 | Not reported | Education of parents: Junior high school or below 345 (35.7%), Senior high school 285 (29.5%), and College and above 337 (34.9%) | Living expenses per month (CNY*): <1,000: N = 201 (20.8%), 1,000~2,000: N = 674 (69.75%), and >2,000: N = 92 (9.5%) |
| Zuniga de Nuncio 2003 | Not reported | Not reported | Employment (n=348): Employed = 65 (18.7%), Not employed = 279 (80.2%), and unknown/missing = 4(1.1%). Overall, prenatal care expenses were covered by MediCal for 69 % of women, 28% paid cash, and only 3% had private insurance |

Footnotes:

All data relate to the individual that the intervention was aimed at. This could be the vaccine recipient themselves, or their parent or caregiver.

Socioeconomic data – Socio-economic details includes socio-economic index data, income data, insurance details, occupation, housing.

## **M: Multicomponent interventions included in the NMA**

| **Ref no.** | **Study** | **Intervention categories included in the intervention** | **Country** | **Age group** | **Vaccine type** | **Description of intervention taken from papers** |
| --- | --- | --- | --- | --- | --- | --- |
| 10 | Bastani 2022 | Education, affordability, reminder | United States | Adolescents | HPV | The multicomponent intervention included brief (5-minute) telephone education, referral to a local HPV vaccine provider, and a mailed brochure. Operators provided basic information about the HPV vaccine and addressed the main vaccine barriers endorsed by the caregiver during the baseline survey (sample telephone education scripts in Supplementary Table S1). Operators also provided caregivers with a customized referral to a local clinic, in a location convenient to the family, where the adolescent could receive the HPV vaccine for free or at a low cost. For adolescents with a usual source of care, operators recommended caregivers return to the usual source of care, often health care facilities outside of the County system such as a community health center, to ensure continuity of care. Operators also accounted for the adolescent’s insurance status and attempted to link adolescents. insurance status, sex of child, and race/ethnicity (e.g., images, risk information). For example, the brochure for caregivers of Latina adolescents mentioned Latinas have higher rates of cervical cancer compared with the U.S. population. The brochure also included information about the clinic to which the operator referred the caregiver for the HPV vaccine. |
| 46 | Daniels 2007 | Access and Education | United States | Older adults | Influenza | Vaccine education session and offered on-site vaccination. During the adult vaccine education session component of the intervention, participants learned about influenza and pneumonia vaccines in group discussions that lasted < 1 hour. Study participants at sites that were randomized for on-site vaccination were also offered the vaccines, which were administered by UCSF (University of California, San Francisco) researchers with medical training. All participants were assessed at baseline and during 3- to 6-month follow-up telephone interviews to assess receipt of vaccination. |
| 246 | Yeung 2018 | Affordability, Education, reminder | Hong Kong SAR, China | Children | Influenza | Subjects in the intervention group received the same standard information about the VSS as the control-subjects. Additional interventions were provided when the child was approximately 6 months old (i.e. eligible to receive influenza vaccine) and at the start of the next VSS (i.e. when influenza vaccine become available for the season and before the arrival of the influenza season). The interventions were: (1) a concise information sheet about the risks of influenza to children and the benefits of influenza vaccination; (2) semi-completed forms required for the VSS, with highlighted guidelines of where to sign the form and a reminder to take the child’s birth certificate to the clinic; and (3) the contact number and address of a specific community health centre registered under the VSS that was in reasonable proximity to their home. The United Christian Nethersole Community Health Service (UCN) is registered under the VSS and has a network of clinics that provides influenza vaccine without any additional cost above the subsidy and without the need for a prior appointment. In 2014/2015, UCN clinics provided trivalent influenza vaccine at no additional cost above the subsidy but quadrivalent influenza vaccine at a cost of HKD20 per dose above the Government’s subsidy. The additional HKD20 per dose of quadrivalent influenza vaccine was compensated by the research funding in terms of supermarket or book coupons at the end of the study to ensure that cost of vaccine was not a barrier to uptake. If there was no UCN clinic near the subjects’ home, a list of other nearby VSS registered clinics that did not charge additional fees was provided. This information package was delivered and explained face-to-face to group 1 intervention-subjects after they were recruited into the RCT. For group 2 intervention-subjects, the package was sent by post and explained over the phone. Both intervention groups received follow-up text message reminders of the contacts of the VSS registered clinics atone week and 1–2 months after the information package was provided. If the mothers had a plan to vaccinate their children with influenza vaccine on a certain date during the discussion of intervention package, a reminder was sent the day before this date. A reminder for the second dose of influenza vaccination was sent if the subject was known to have received the first dose and if this was not known, the planned text reminder was sent 1–2 months after the information package was provided. |
| 25 | Bronchetti 2015 | Access, education. Reminder | United States | Adults | Influenza | Email reminders: Dear Erin: As a fellow Swarthmore student, I want you to know that I think it’s a good idea to get a flu vaccine. By getting vaccinated you will protect yourself, and fellow students, from getting sick. No one wants to get sick, especially at crucial times like midterms or finals. It is easy to get a flu vaccine on campus. It is safe, and now is the right time! You can get the vaccine at Worth Health Center beginning Wednesday, October 3rd. You do not need an appointment! The cost is $20 and can be billed to your student account or paid by cash/check. To help the student health center’s planning, please let them know: Do you plan to come in for a flu vaccine? By responding below (clicking EITHER yes or no), you can enter to win one of five $100 prizes. Prizes will be distributed to winners' student mailboxes at the beginning of finals week. YES, I think I will probably get a flu vaccine: Click here. NO, I do not think I will get a flu vaccine: Click here. It is also possible to make a flu vaccine appointment if you want to, on T/W/Th between 1:30 and 4:00 p.m. This helps minimize wait times. If you have any questions about flu vaccination, please contact the Health Center at 610-328-8058. Stay Healthy! From John Do |
| 216 | Szilagyi 2011 | Affordability and reminders | United States | Adolescents | HPV | The intervention consisted of a tiered protocol. Each step was more intensive and targeted a progressively smaller proportion of adolescents who remained behind in immunizations despite the previous steps. This method, modeled after a childhood program, minimized the intervention needed for each adolescent. The intervention was delivered by trained patient immunization navigators (4.5 full-time equivalents), analogous to chronic disease patient navigators or promotors. The navigators were recruited from the community; 1 fluent Spanish speaking navigator was placed in the practice with the largest Hispanic population. They received formal training on the intervention, use of a database, health promotion, and methods to assist families to navigate the health and social service systems. The navigators were provided a workspace and a computer at each practice, and all were supervised by a social worker (M.S.). Their percentage effort in each practice was determined by their caseload, which varied from 600 to nearly 1000 per full-time equivalent. Step 1: Patient Tracking - Because the study occurred before practices had incorporated the statewide immunization registry for adolescent vaccinations, we created a Web-based database for navigators to track the adolescents, record immunizations and preventive care visits, and document tasks performed. Step 2: Reminders/Recall - Navigators performed reminder/recall for adolescents who were eligible for either a vaccination or a preventive care visit (with a 1-month grace period). They attempted to contact families by telephone (>=2 attempts at different times of day on different days) and mail (2 letters sent 2 weeks apart). The protocol involved 2 telephone calls (>1 week apart) and then 2 letters, and it started with letters if no telephone number was available. The navigators used a patient-centered and partnership-building approach to increase family awareness of preventive health measures and to address barriers to care. They offered transportation assistance (bus tokens and transport by car). If after the reminder/recall parents did not make and keep appointments, vaccination status was not brought up-to-date at the visit, or a subsequent human papillomavirus vaccination was needed, the navigators reinitiated the cycle. After 2 telephone calls and 2 letters, they moved to step 3. Step 3: Home Visits - If adolescents remained unvaccinated despite the previous steps, the navigators performed a home visit to further assess barriers, promote the importance of preventive care, and encourage families to make appointments. The number of home visits was kept low to minimize personnel costs and maximize feasibility and sustainability. Control subjects received standard of care. All the practices routinely sent letter or telephone reminders to families who had upcoming scheduled visits, but none used active reminder/recall based on vaccinations. |
| 158 | O'Grady 2022 | Access and reminder | Australia | Young children | Childhood Vaccines | ESMS±S: At two weeks before and the week of each age milestone due date a more detailed  SMS was sent to the carer’s nominated mobile number (Box 1). Two weeks after the second  SMS had been sent, staff checked immunisation records to determine if the infant had been  vaccinated. If no record was found, vaccination status was confirmed with the primary carer  and, if not vaccinated, they were offered either a home visit or active support for the carer to get  the infant to a healthcare provider for immunisation prior to one month past the milestone  due date. Text states “Dear <carer name>, this is a reminder that <baby’s name> <age in months> baby needles are/were due on <date>. It is important that <he/she> gets these on time so <he/she> doesn’t get sick from the diseases that these immunisations protect against. Please make an appointment with your doctor. Please reply “Yes” if these baby needles have already been given or “No” if not”. All infants not age-appropriately immunised at seven months of age were offered support to the parent/carer, including home visits, to complete outstanding vaccinations. For the simple SMS and ESMS±S groups, whether or not a response was received was recorded at each contact timepoint. |
| 184 | Saaksvuori 2022 | Access, education, reminder | Finland | Older adults | Influenza | The individual-benefits reminder contained basic information about the severity of influenza symptoms, seasonal influenza vaccination, the availability of vaccinations (locations and dates to receive the vaccine), and instructions about how to book an appointment with the vaccine administration... Invitation to Influenza Vaccination! We would like to invite all citizens aged 65 and above living in Coastal Ostrobothnia (Maalahti, Korsnäs, Närpiö, Kaskinen and Kristiinankaupunki) for free influenza immunization. Seasonal influenza is a common and serious disease in the age group of 65 and above. Influenza vaccination is the best way to protect you against the disease. Influenza vaccine will protect you also against many secondary diseases associated with seasonal influenza such as pneumonia. It is recommended to take an influenza vaccine every autumn as the protective effect of these vaccines last for about a year. Influenza viruses continuously change and previously taken vaccines may not provide protection in the following years.  You may receive your influenza vaccine without appointment at following dates and times  Kristiinankaupunki – Children’s health clinic (Address: Lapväärtintie 10)  • Monday 29.10. from 1 p.m. to 5 p.m.  • Monday 05.11. from 1 p.m. to 5 p.m.  • Monday 26.11. from 1 p.m. to 5 p.m.  • Monday 10.12. from 1 p.m. to 5 p.m.  Siipyy – Children’s health clinic (Address: Långvikintie 16)  • Wednesday 31.10. from noon to 3 p.m.  • Wednesday 14.11. from noon to 3 p.m.  You may also book an appointment for the vaccine administration on weekdays by calling 06 221 8480  Please bring you social security card with you. We recommend wearing clothes that enable injection of a vaccine into the shoulder. Please notice that if you receive medical home care or live in a nursing home, you may receive an influenza vaccine directly through your care givers. You do not have to book an appointment and travel to receive your vaccine. Welcome Peter Riddar, Chief physician |
| 153 | Nehme 2019 | Reminder and affordability | United States | Adults | Influenza | Participants in group 2 (electronic messages only) and group 3 (electronic plus postal mail group) were sent a total of 4 electronic messages. A text message sent on September 12 and e-mail on October 9 encouraged members to get their free influenza vaccination and provided information about the $10 incentive. A second e-mail sent on November 13 and second text sent on November 17 reminded members to get their influenza vaccination before the end of the year to be eligible for the incentive. You can get your free flu shot at any in-network pharmacy, including HEB, Randall’s, Target, Wal-Mart, CVS and Walgreens. For a complete list of pharmacies, visit our website at www.senderohealth.com. You can also get a free flu shot at your doctor’s office or health clinic. Be proactive, and protect yourself, your family and community from the flu. To learn more, call Sendero Health Plans at (512) 593-6227 |
| 220 | Tentori 2022 | Reminder and access. | Italy | Adults | COVID-19 | On July 23, all participants were sent, by post, a letter from the prevention department of the APSS reminding them of the importance and availability of COVID-19 vaccination. The letter was identical for the two groups, apart from appointment-scheduling information. For the opt-in group, this section constituted instructions on how to schedule an appointment for the vaccination (via web or by contacting their family doctor). For the optout group, it comprised date, time, and location information for a scheduled vaccination appointment, together with instructions on how to modify the arrangement, if desired. All participants were offered the BNT162b2 Pfizer/BioNTech vaccine |
| 34 | Chang 2023 | Affordability and reminders | United States | Adults | COVID-19 | Individuals in the reminder and reminder with financial incentive arms were sent a message reminding them that they were “due for a COVID-19 booster shot” and should not delay (SI Appendix, section 1). The reminder message provided a link to an easy-to-use vaccine scheduler maintained by the county health system. Those assigned to the financial incentive condition received the same reminder message but were also told that they would receive a $25 gift certificate if they were boosted within the next 2 wk. Reminder messages were sent by the health system on December 8, 2022, when bivalent booster vaccinations were on the decline (SI Appendix, Fig. S1) but in advance of the Christmas holiday. Reminder messages were sent in either English or Spanish, depending on the  preferred language listed on the file. Patients who listed a preferred language other than English or Spanish received a message in English. Message states: CCHS: [FIRST NAME], our records show that you are due for a COVID-19 booster shot. Don’t delay, visit https://covidvaccine.cchealth.org/COVIDVaccine/ and schedule your booster today.  As a special incentive, if you are boosted within the next 2-weeks, you will be eligible for a 25  USD Amazon reward. For more information visit https://cchealth.org/medicalcenter/covid-  vaccine-incentives.php |
| 99 | Hwang 2010 | Access and education | United States | Adults | Hepatitis B | Participants enrolled in even-numbered months followed the accelerated schedule of 0, 1 and 2 months. The HBV vaccination behavioural intervention of four, 15–20 min sessions was based on  brief self-efficacy interventions previously developed for community-based HIV prevention  programs [24]. The purpose was to increase drug users’ acceptance and adherence to HBV  vaccine protocols by increasing self-efficacy, positive outcome expectations, perceived peer group support, and the value attached to HBV vaccination. The intervention provided accurate and salient information about HBV and HBV vaccination, the benefits that could be gained and the losses avoided by being tested and vaccinated for HBV, vicarious experience (discussion, stories, modelling, graduated mastery learning processes), verbal persuasion by peer outreach workers, and positive emotional arousal. Sessions 1 and 2 were delivered at screening and enrolment, after obtaining written informed consent, and Session 3 at the one-month visit (2nd dose). Session 4 was delivered before the 3rd dose of vaccine: for the accelerated schedule, at a 6-week visit with the last dose at 2 months; for the standard schedule, at the 2-month visit with the last dose at 6 months. |
| 148 | NCT05012163 2024 | affordability and reminders | United States | Adults | Influenza | Participants in this arm will receive a message stating that they will receive a PA lottery $1 scratch-off ticket if they get a flu shot at an upcoming appointment. The message will mention that they could win $5,000 (the top prize for the scratch-off game). Note: $1 scratch-off products vary over time; at study implementation, an active game with top prize of $5,000 (or the next-highest top prize) will be selected and will define the prize in the raffle absent upfront odds |
| 151 | NCT05534061 2022 | Education and affordability | United States | Adults | COVID-19 | $10 financial incentive for vaccination and $10 financial incentive for testing plus a brief feedback-based motivational enhancement intervention. Connect2Test: Brief motivational enhancement intervention to improve the likelihood of testing and vaccination among syringe exchange clients  The Connect2Test intervention is designed to be approximately 2-5 minutes and focuses on using  motivational interviewing skills to facilitate uptake of COVID-19 testing and vaccination. Specifically, strategies include asking for permission to share information, using open-ended questions, reinforcing change talk, and using reflections to check for understanding. In Phase II, the syringe exchange staff or volunteer will initiate the conversation using an open-ended question and then have an informal conversation with participants using the skills they learned in MI training with Dr. Anne Marie Mauricio. |
| 222 | Thilly, 2024 | Access and education | France | Adolescents | HPV | Component 1 (adolescents’ education and motivation) was carried out in middle schools.  Parents were first invited by the school to a web conference to receive information on HPV infection  and vaccination (from December 2021 to January 2022). Pupils in grades 8 and 9 (13-14 years of age)  then attended 2 educational group sessions on those topics during school hours (from January to  March 2022). Each session used participatory learning and e-health tools (videos, serious game)  developed by the PrevHPV program. Component 3 (HPV vaccination at school) consisted in offering free vaccination on school premises on specific days (from March to April 2022 [ie, after the educational group sessions had been completed]). Vaccines were administered by health professionals from local vaccination centers to eligible adolescents. |
| 138 | Masson 2013 | Access, Affordability, education | United States | Adults | Hepatitis B | Participants in both groups received individual 2-session manual-guided HIV and viral hepatitis counselling and education administered by research staff using a laptop-based slide presentation. The education and counselling included HIV and viral hepatitis pretest counselling, voluntary testing, post-test counselling, and the provision of test results at the second session. Basic information about HIV and viral hepatitis transmission, prevention, and treatment as well as the benefits of HAV–HBV immunization was provided by research staff. This educational and counselling session constituted a manual-guided intervention reflecting what is recommended by the Centers for Disease Control and Prevention for all drug users and by various public health agencies for drug treatment programs.40 The post-test counselling session took place 1 to 2 weeks following the pretest counselling session. The hepatitis care coordination intervention included the individual 2-session HIV and viral hepatitis pretest and post-test counselling and education provided to the control group, with the difference that it was delivered in a motivational interviewing style.44 The intervention group also received serological testing for HIV and hepatitis viruses, on-site vaccination, and, for a period of 6 months, motivational interviewing–enhanced case management assistance with vaccination and off-site HCV evaluations. Participants who, on the basis of serologic test results, were susceptible to HAV, HBV, or both were offered combination vaccine (Twinrix; GlaxoSmithKline, Rixensart, Belgium) on site at the MMT program. Following the instructions on the package insert, MMT program staff administered the combined HAV–HBV vaccine as closely as possible to the recommended schedule at baseline, 1 month, and 6 months. The investigative team designed case management sessions to facilitate access to needed hepatitis medical care, including HAV–HBV vaccination and HCV clinical evaluations. Case managers coordinated with primary care and hepatology clinics to schedule HCV evaluation appointments. In San Francisco, participants were referred for clinical visits in a building adjacent to the methadone program; in New York City, participants were referred for the same services 9 blocks away. Case managers scheduled initial and follow-up patient appointments, accompanied patients to these appointments, negotiated new appointments when participants failed to attend medical care visits, and provided follow-up reminder phone calls and letters. Case managers also assisted patients in accessing psychiatric services, alcohol treatment, legal assistance, and social service entitlements. Case management sessions were held weekly, lasted an hour or less, and were not compensated, but transportation tokens or bus or subway cards were provided on a case-by-case basis by interventionists if transportation could affect adherence to the intervention. Case manager caseloads included approximately 15 participants throughout the study. Case managers had bachelor’s degrees and a minimum of 1 year of experience working with substance-abusing populations and were supervised by a psychologist. |

## **N: Risk of bias assessment for included studies**

Traffic light plots showing risk of bias assessments for each study. Figure made using Robvis tool: McGuinness, LA, Higgins, JPT. Risk-of-bias Visualization (robvis): An R package and Shiny web app for visualizing risk-of-bias assessments. Res Syn Meth. 2020; 1- 7. <https://doi.org/10.1002/jrsm.1411>

Traffic-light plot showing risk of bias for individually randomised RCTs.


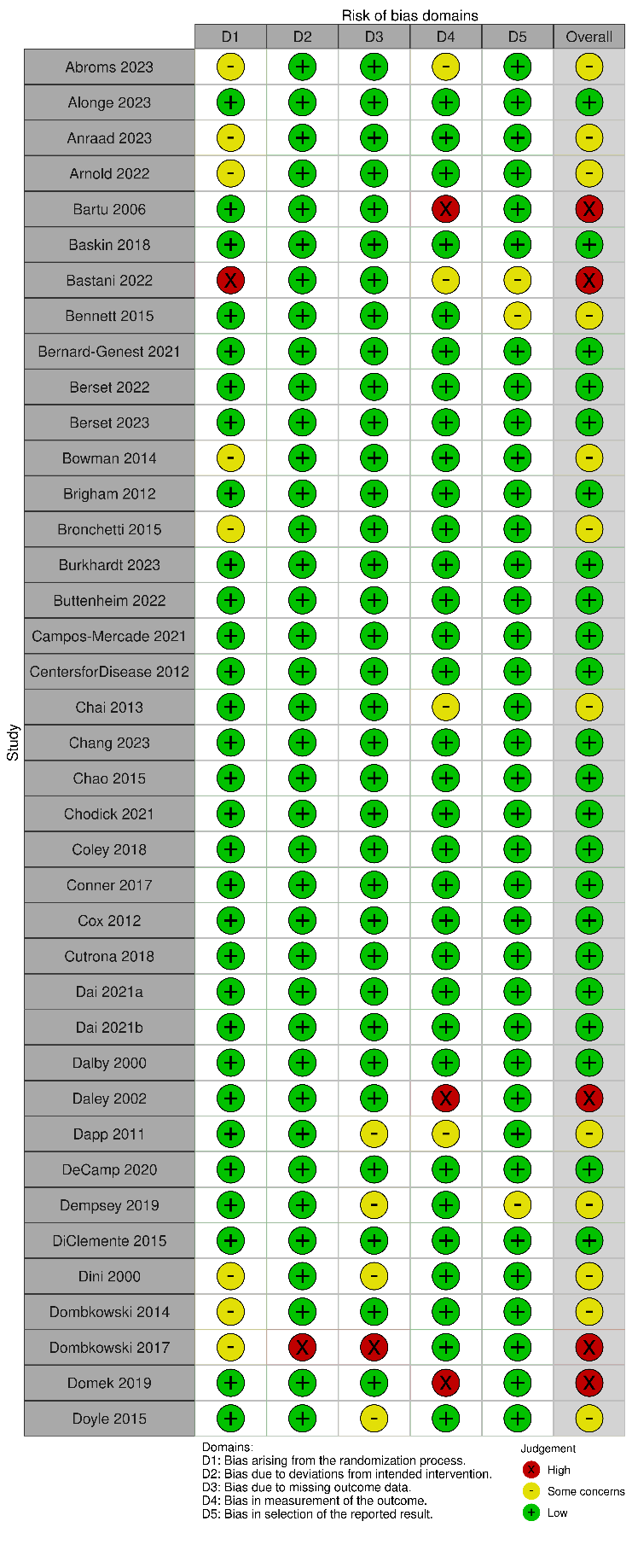


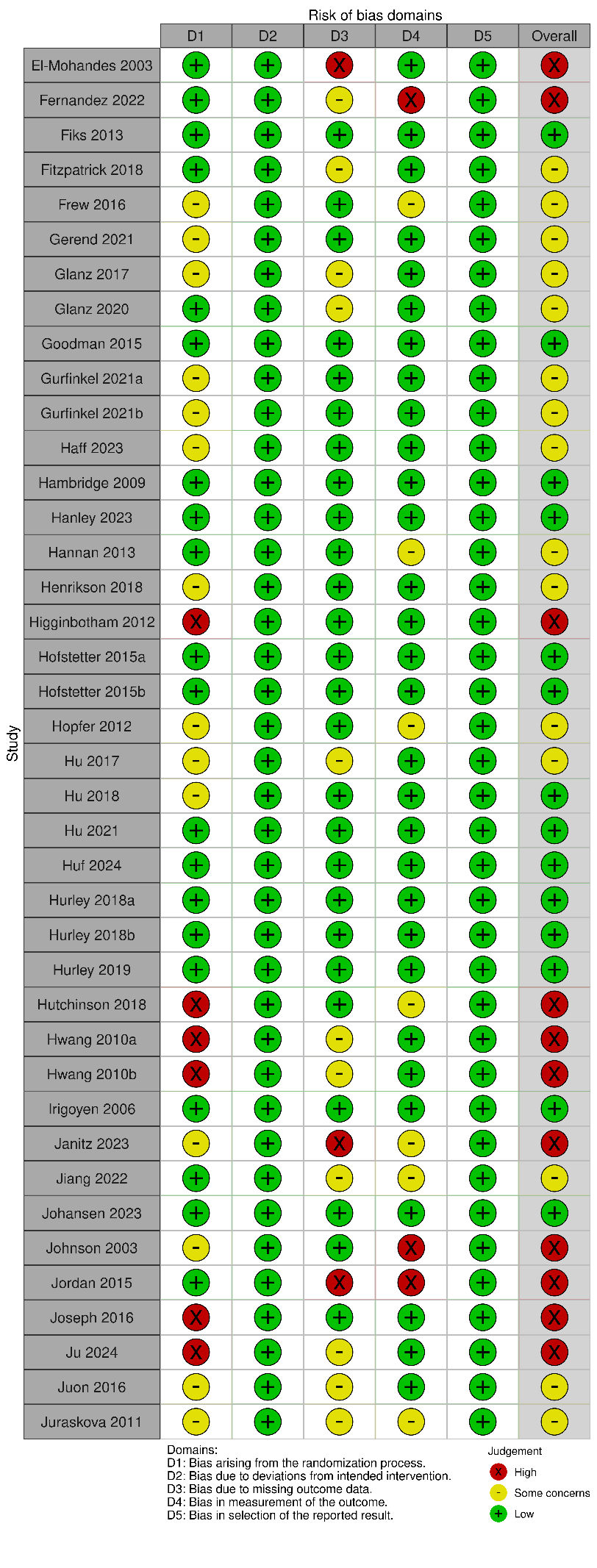


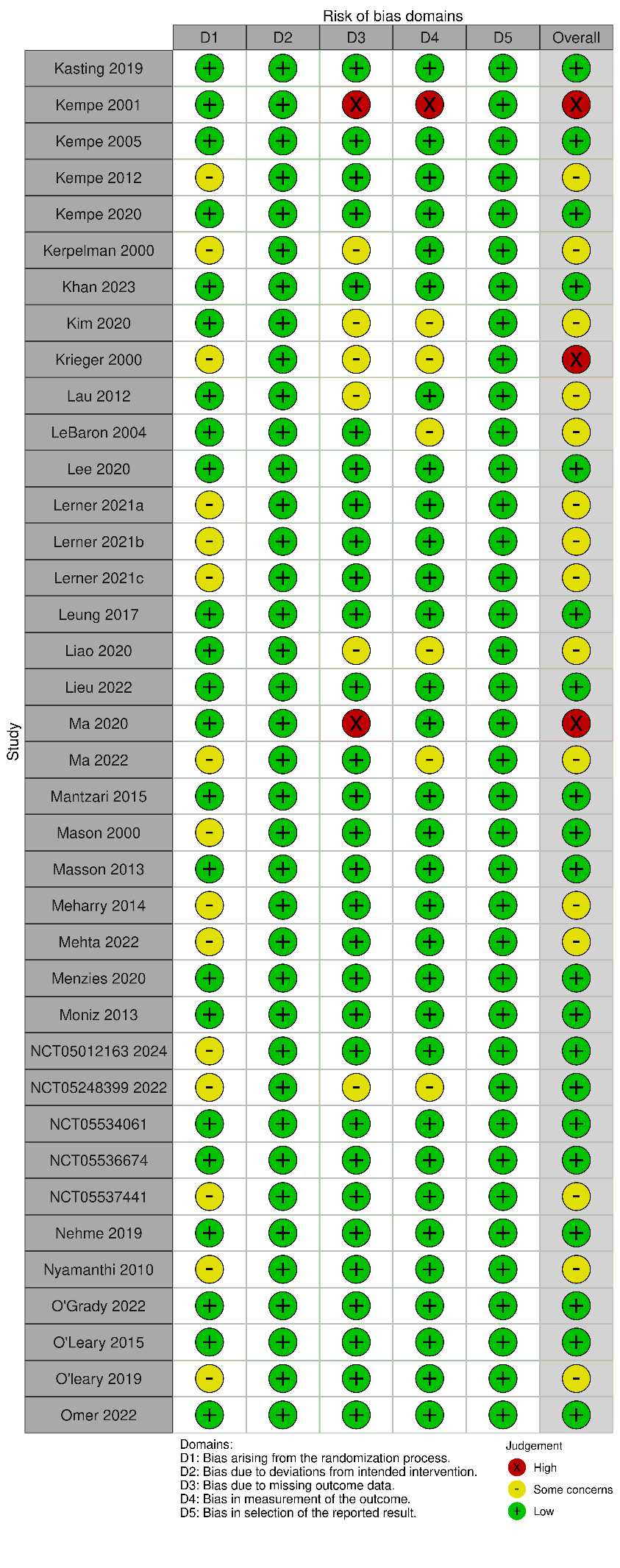


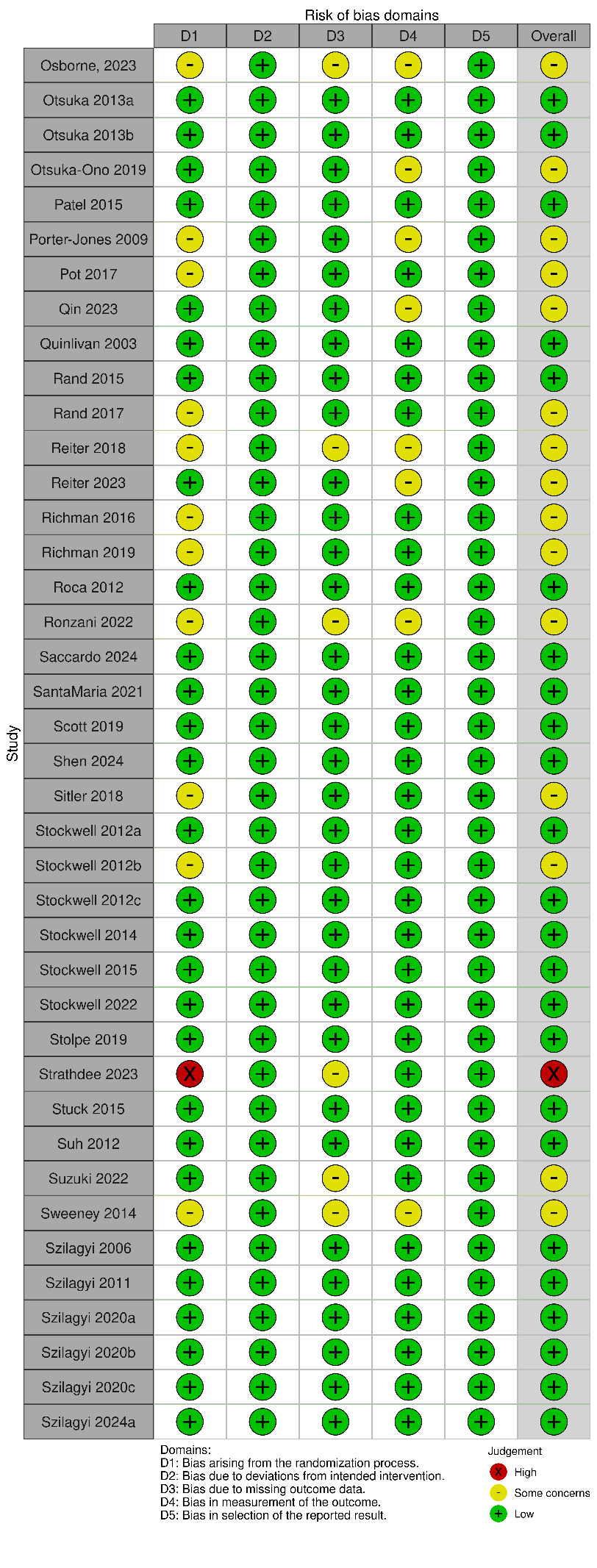


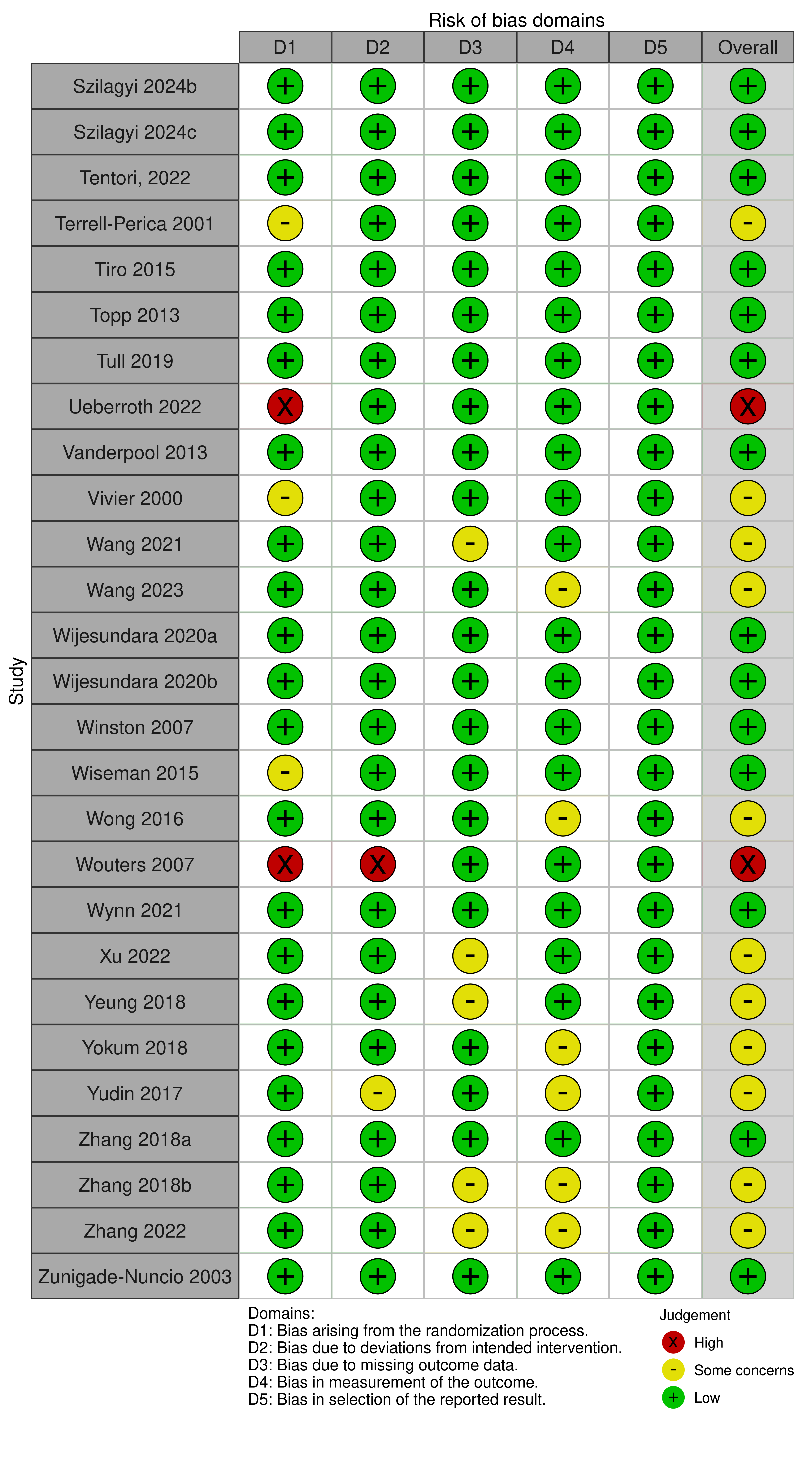


Traffic-light plot showing risk of bias for cluster-RCTs.


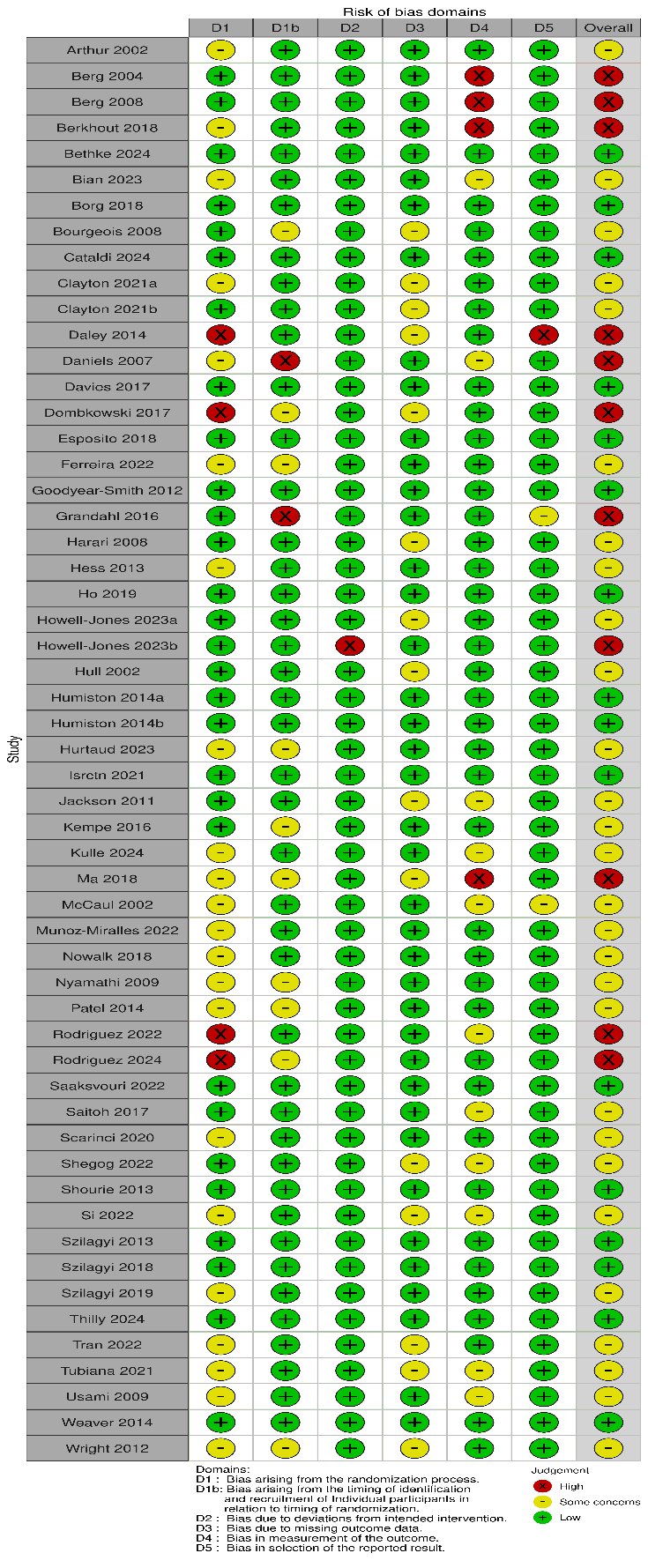


## **O: Confidence in the evidence**

| **Comparison** | **Within-study bias^a^** | **Reporting bias^b^** | **Indirectness^c^** | **Imprecision^d^** | **Heterogeneity^d,e^** | **Incoherence^d^** | **Confidence rating^g^** |
| --- | --- | --- | --- | --- | --- | --- | --- |
| **Access vs. Control** | Some concerns | Low risk | No concerns | No concerns | Some concerns | No concerns | Moderate |
| **Affordability vs. Control** | No concerns | Low risk | No concerns | No concerns | Some concerns | Some concerns | Moderate |
| **Education vs. Control** | Some concerns | Low risk | No concerns | No concerns | Some concerns | Some concerns^f^ | Moderate |
| **Education and Reminder vs Control** | No concerns | Low risk | No concerns | No concerns | Some concerns | No concerns | Moderate |
| **Multicomponent vs Control** | No concerns | Low risk | No concerns | No concerns | Some concerns | No concerns | Moderate |
| **Reminder vs Control** | No concerns | Low risk | No concerns | No concerns | Some concerns | Some concerns | Moderate |

Footnotes:

^a^ Based on average risk of bias for each comparison.

^b^ Although funnel plots for pairwise meta-analyses of each intervention category vs. control (except multicomponent interventions) showed evidence of small-study effects, the asymmetry cannot necessarily be assumed to arise from publication bias since it is consistent with true differences between intervention effects in small versus large studies. Furthermore, the conclusions were similar in sensitivity analyses assuming fixed-effect models, in which the study weights are much higher for the larger studies.

^c^ Studies specifically conducted among socially excluded populations (e.g. people experiencing homelessness, people who use drugs) were considered less representative of the general population and therefore judged to be at 'moderate' indirectness. All other studies were judged to be at 'low' indirectness as they were in the general population. The comparison-level judgments are based on the average contribution of studies (i.e. the average indirectness for all comparisons was 'low' leading to a judgement of 'no concerns').

^d^ Based on a clinically important odds ratio of 1.2.

^e^ Heterogeneity judgements were based on inspection of the results of both our Bayesian analysis and the frequentist CINeMA output. Our Bayesian analysis produced a large tau^2^ (0.176) with consequently wide prediction intervals which extended beyond the clinically important effect in the opposite direction to the effect estimate for all comparisons. The tau^2^ produced by the CINeMA software was much smaller (0.019), with consequently narrower prediction intervals, although the DerSimonian and Laird method which is used to estimate tau^2^ in CINeMA is known to be negatively biased in a number of scenarios including where I^2^ is high, as is the case here. We therefore took a balanced and pragmatic approach, resulting in a judgment of 'some concerns' for all comparisons.

^f^ Whilst the CINeMA software produced an automated judgement of 'major concerns' for the 'Education vs Control' comparison, this was driven by a small number of studies with strongly outlying results (n=5). When these five studies were removed from the dataset uploaded to CINeMA the automated judgement was 'some concerns'. Given that our sensitivity analyses (Appendix R) showed that the findings of the network-meta-analysis remained stable when these strongly outlining studies were removed, we opted for a judgement of 'some concerns'.

^g^ Confidence was rated down by one level for all comparisons, avoiding downgrading more than once for related concerns.

## **P: Checks for inconsistency**

To check for inconsistency, we compared the fit of our NMA model to an unrelated mean effects model. The deviances represent how well each data point fits the model predictions. We present a plot of the deviances below and observe very similar patterns of deviances in the two models indicating no inconsistency.

**
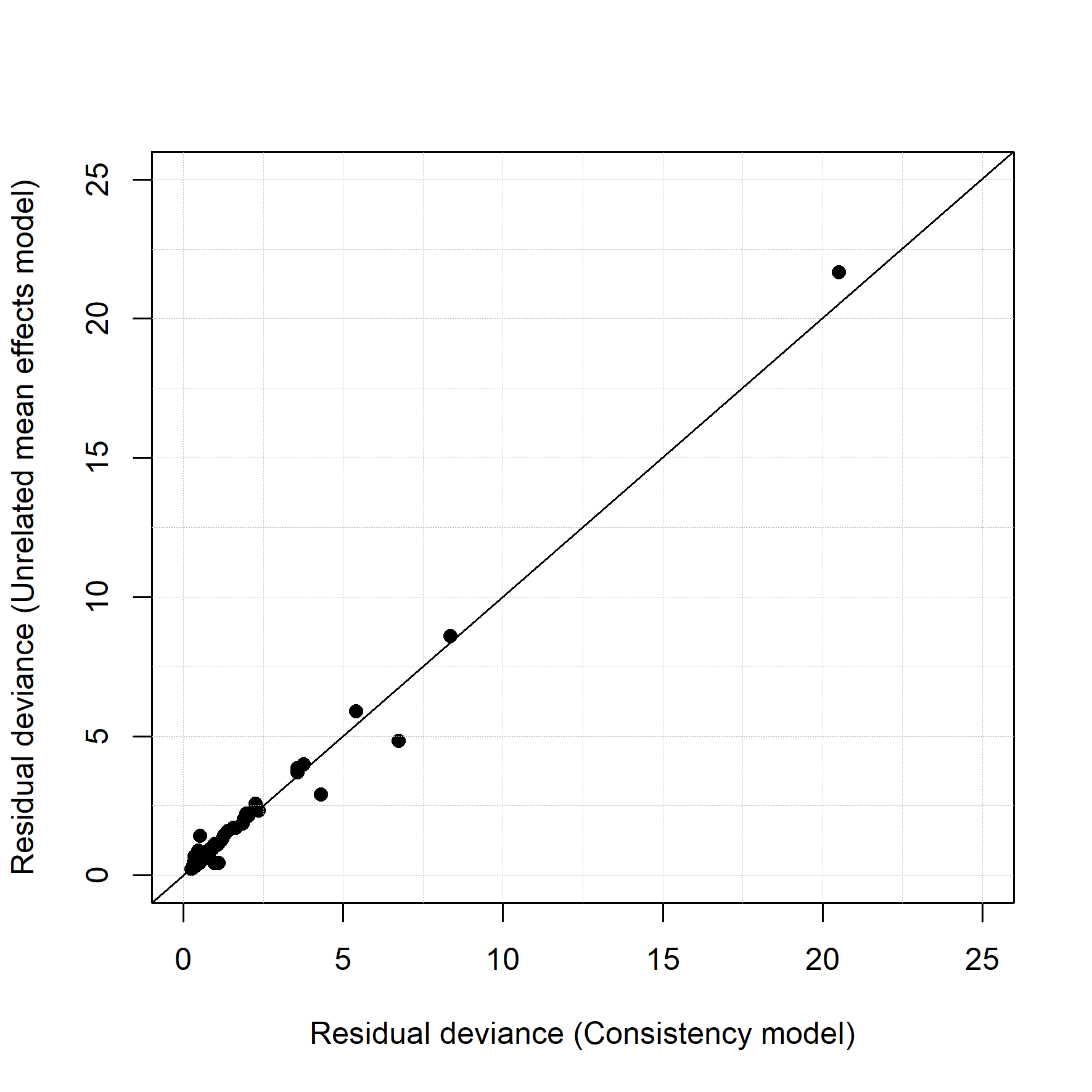
**

**Main: pD =159.9327 DIC= 691.1956**

**UME: pD =157.606 DIC= 693.8314**

## **Q: Pairwise meta-analyses: results**

Pairwise meta-analyses were conducted for all intervention and control and all intervention and intervention comparisons for which direct head-to head evidence was available. We present all forest plots below with effect estimates presented as odds ratios and 95% confidence intervals.

**
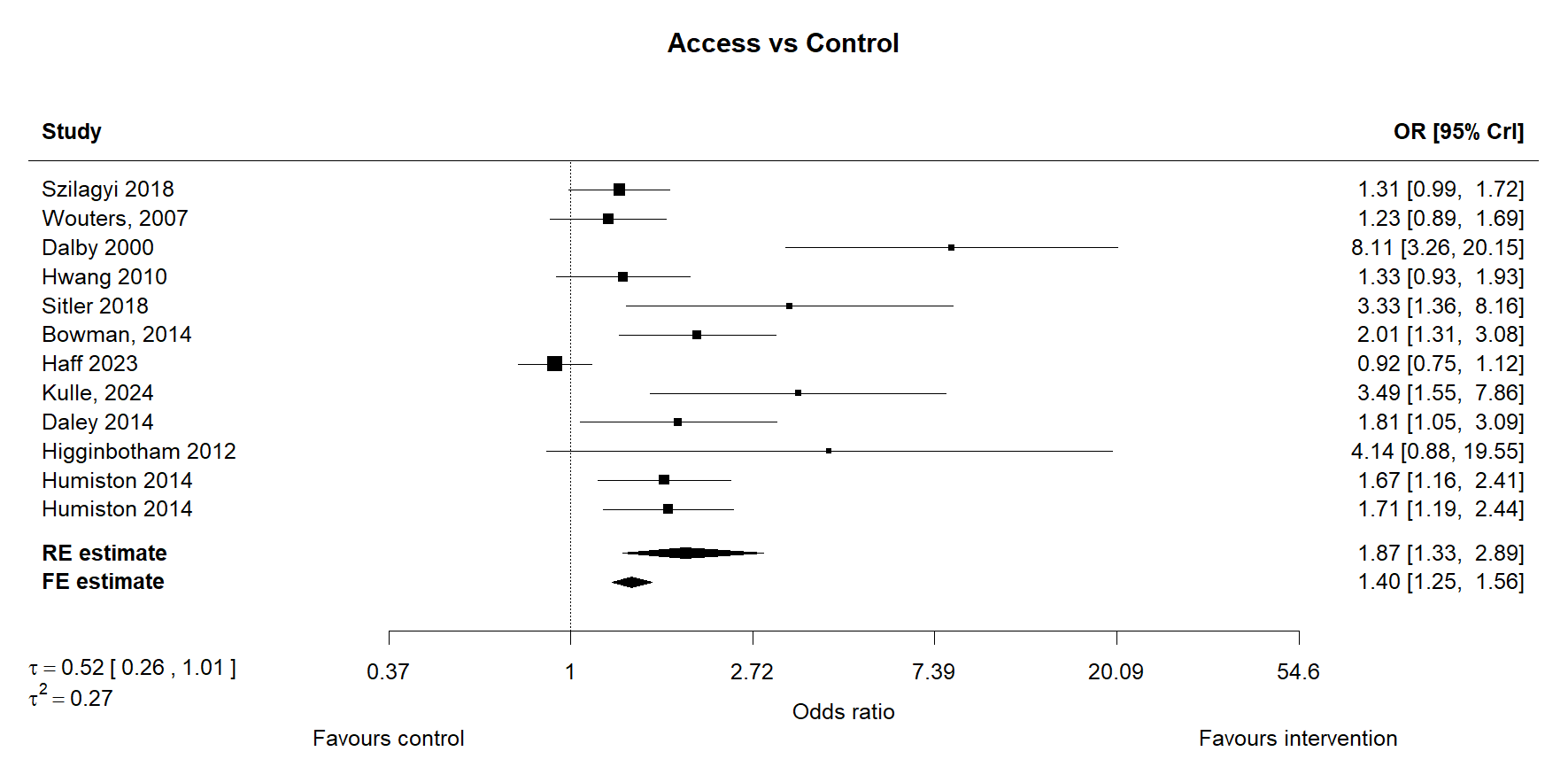

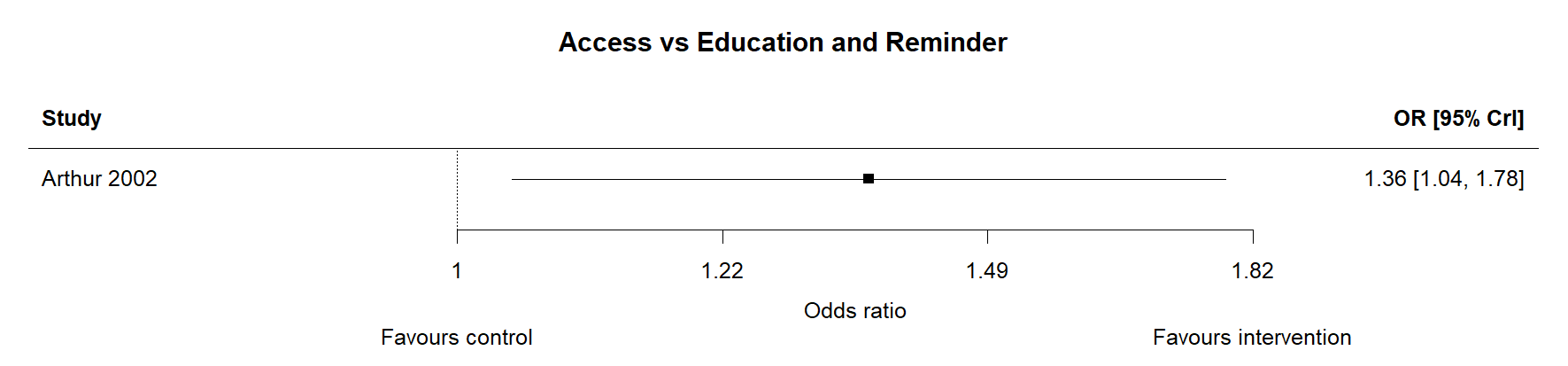

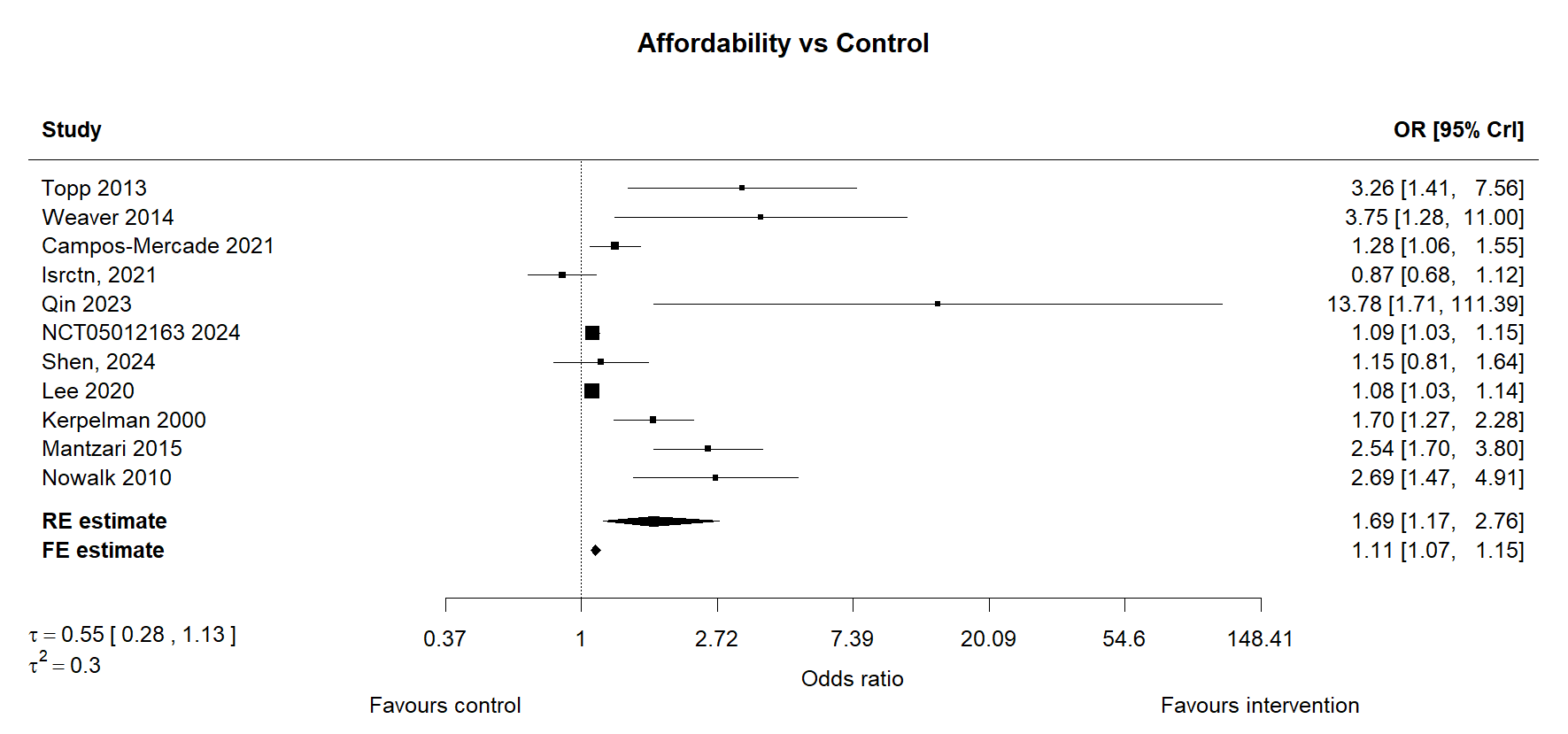

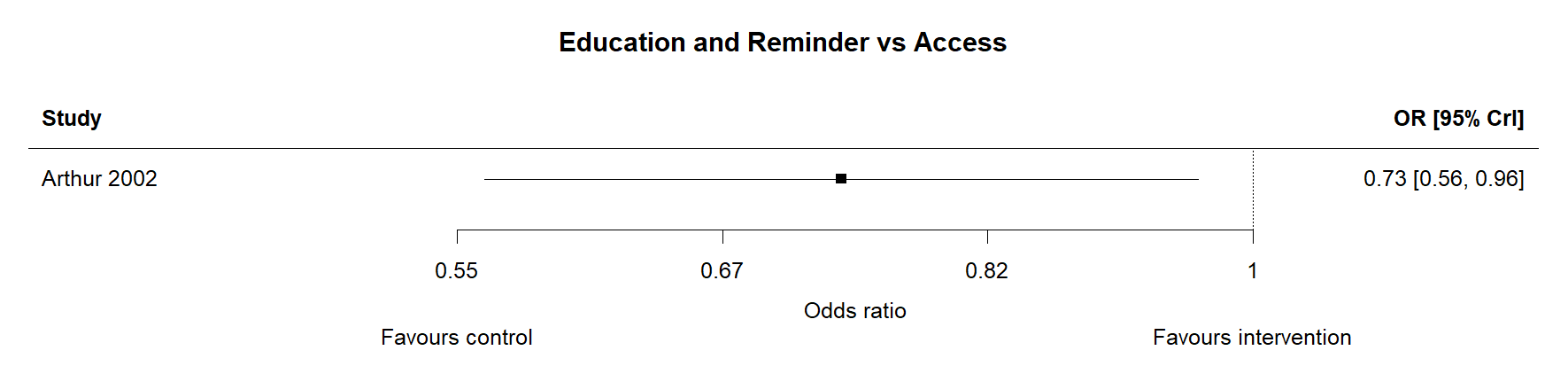

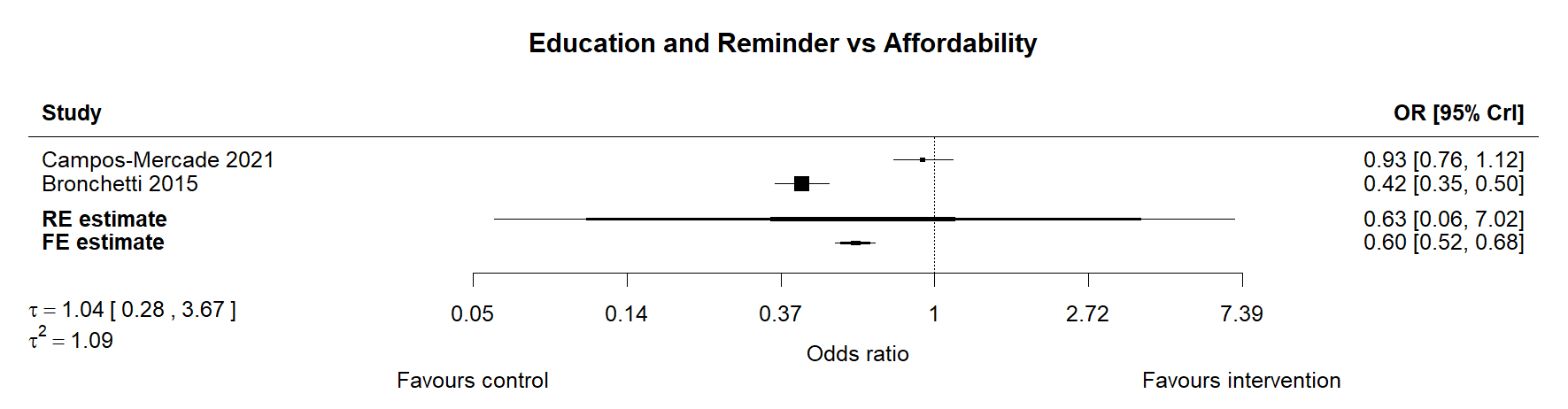

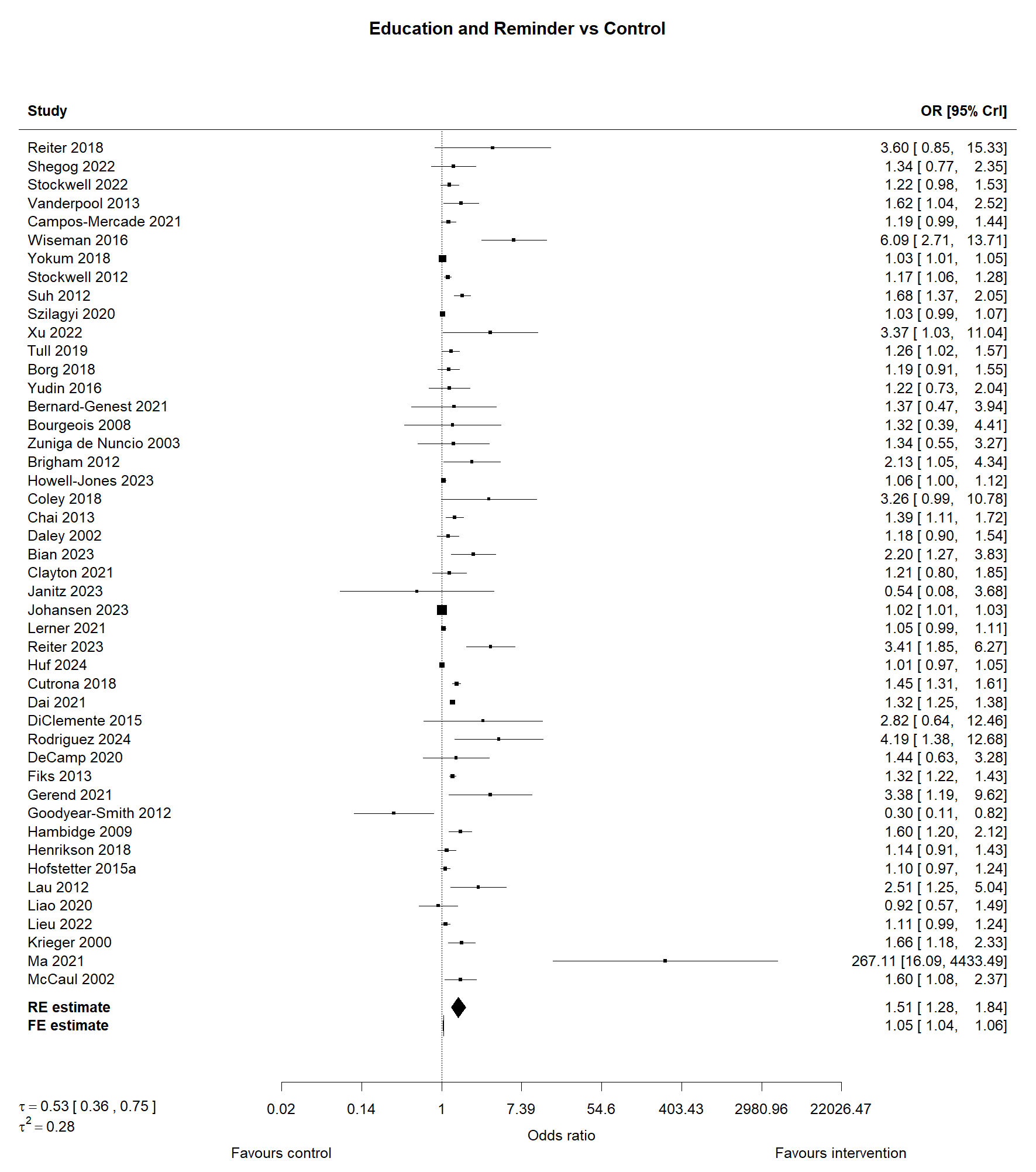

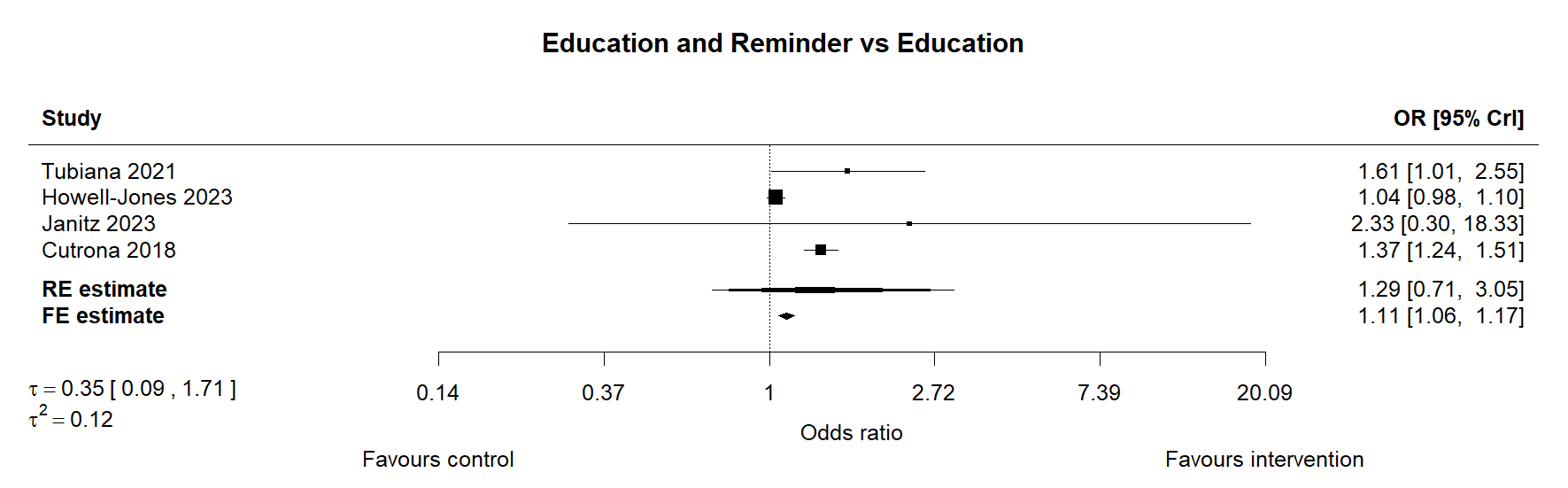

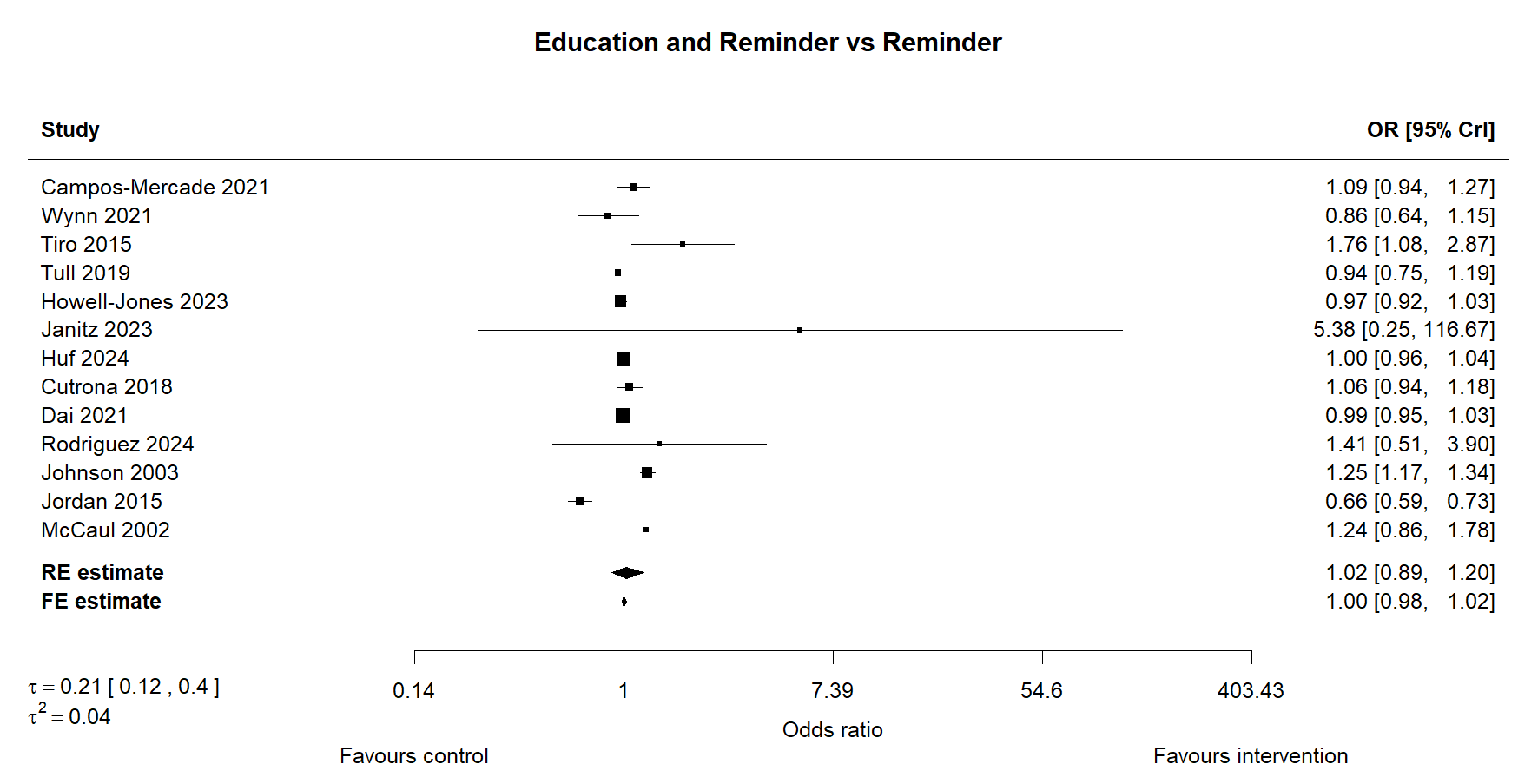

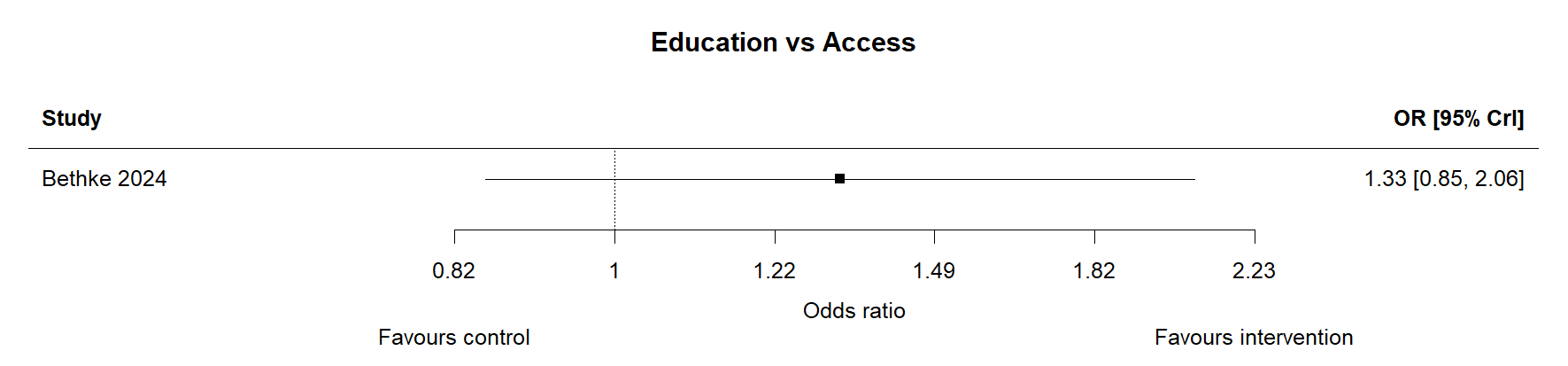

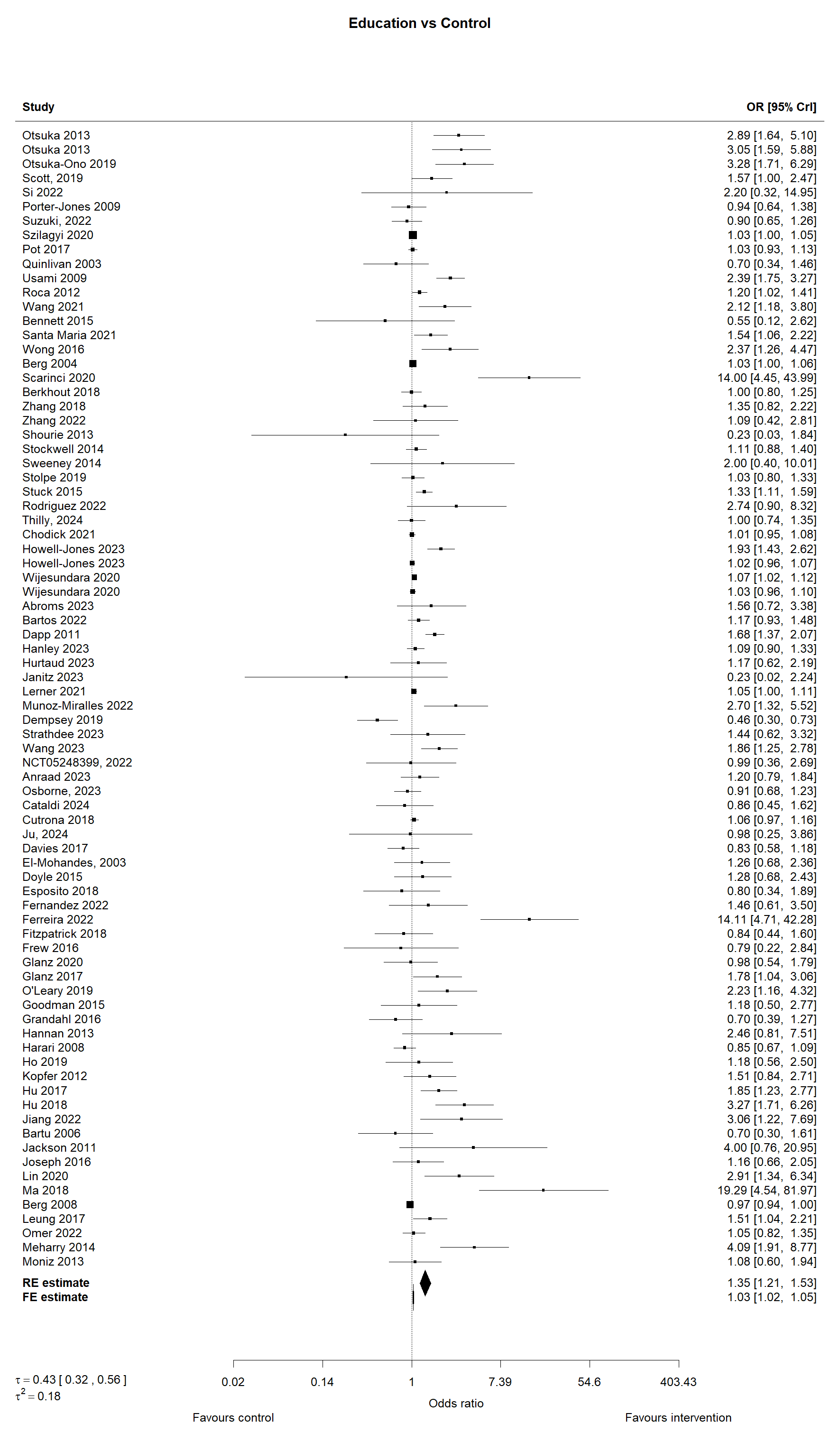

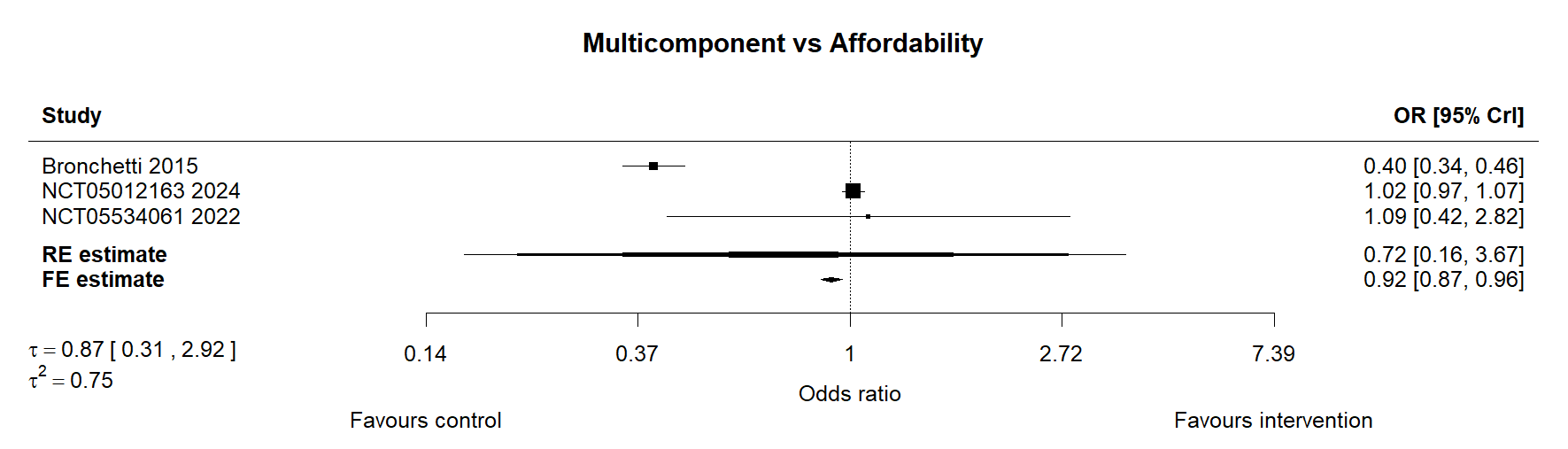

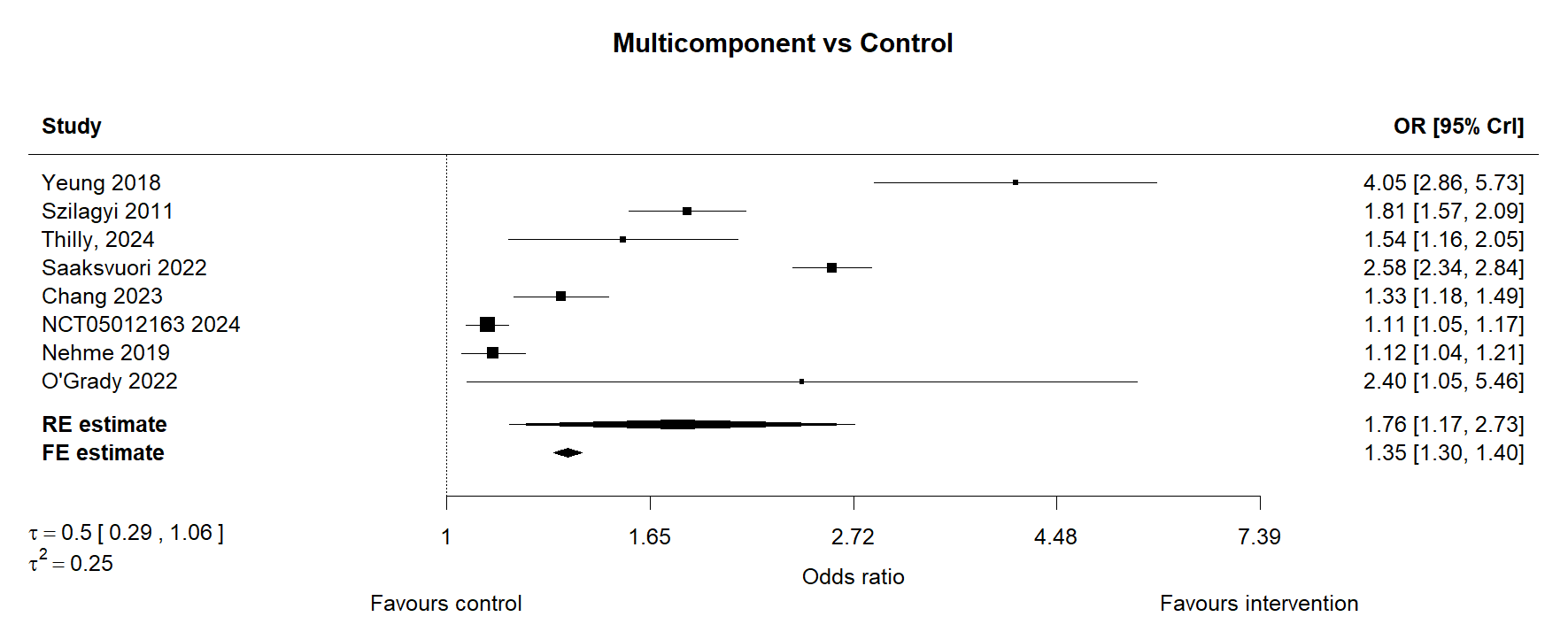

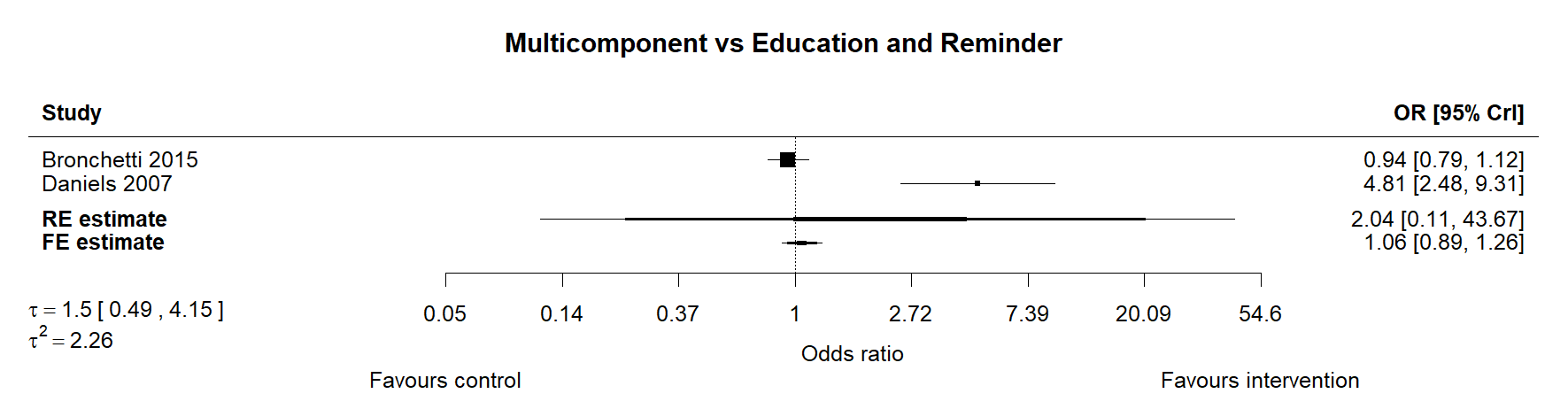

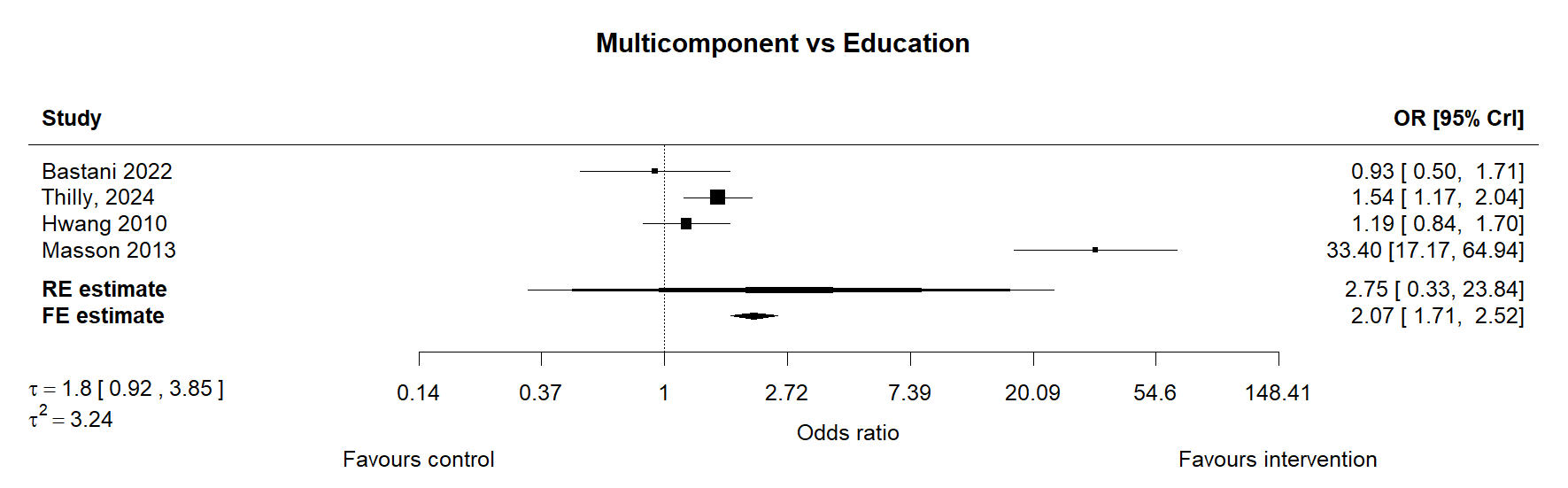

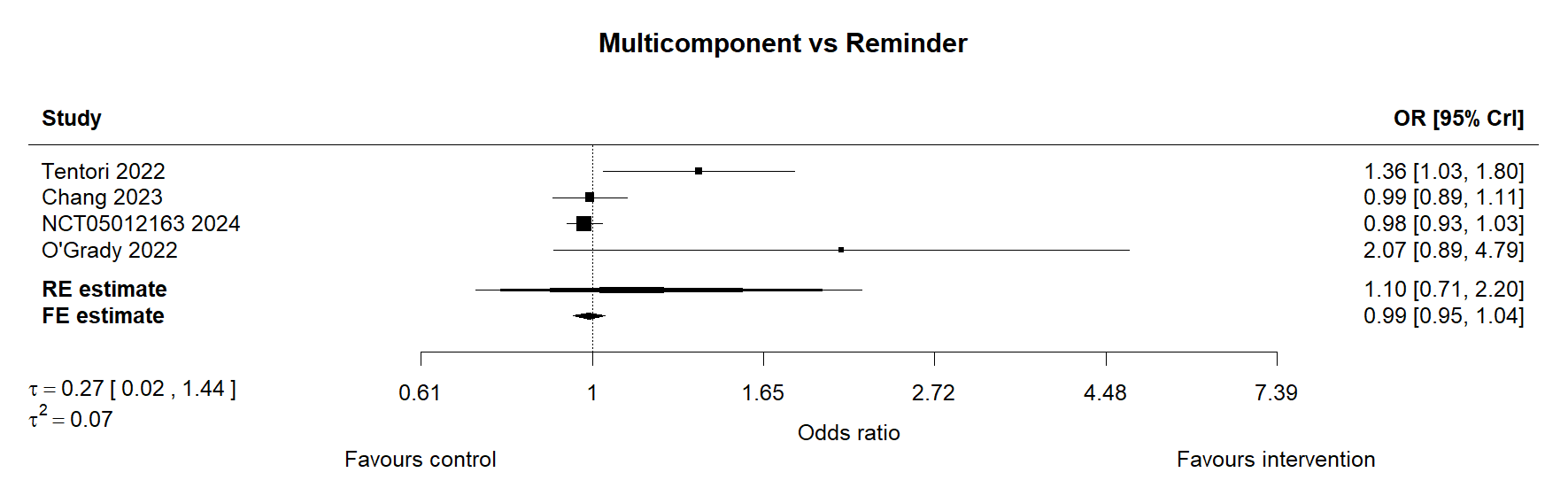

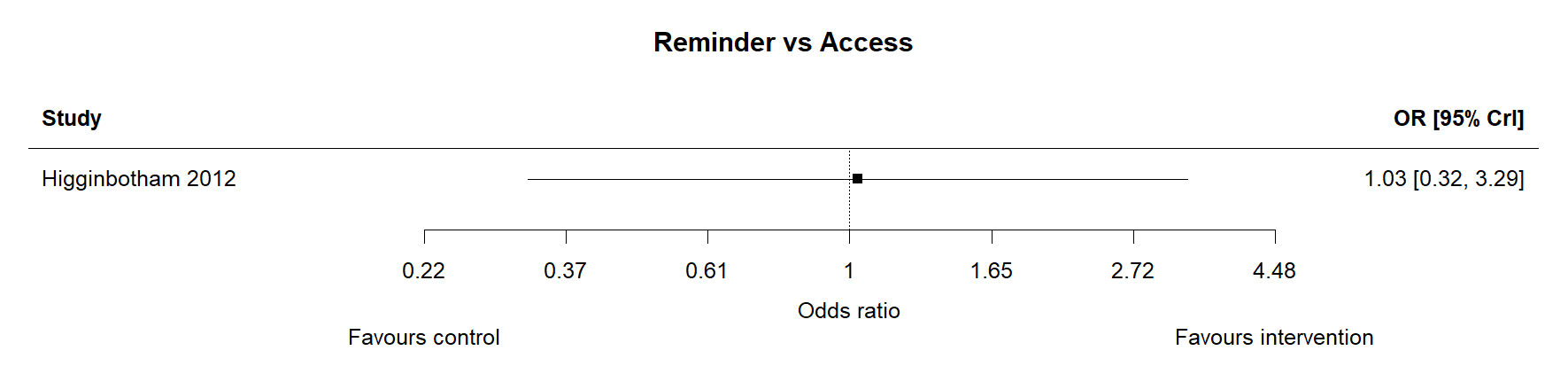

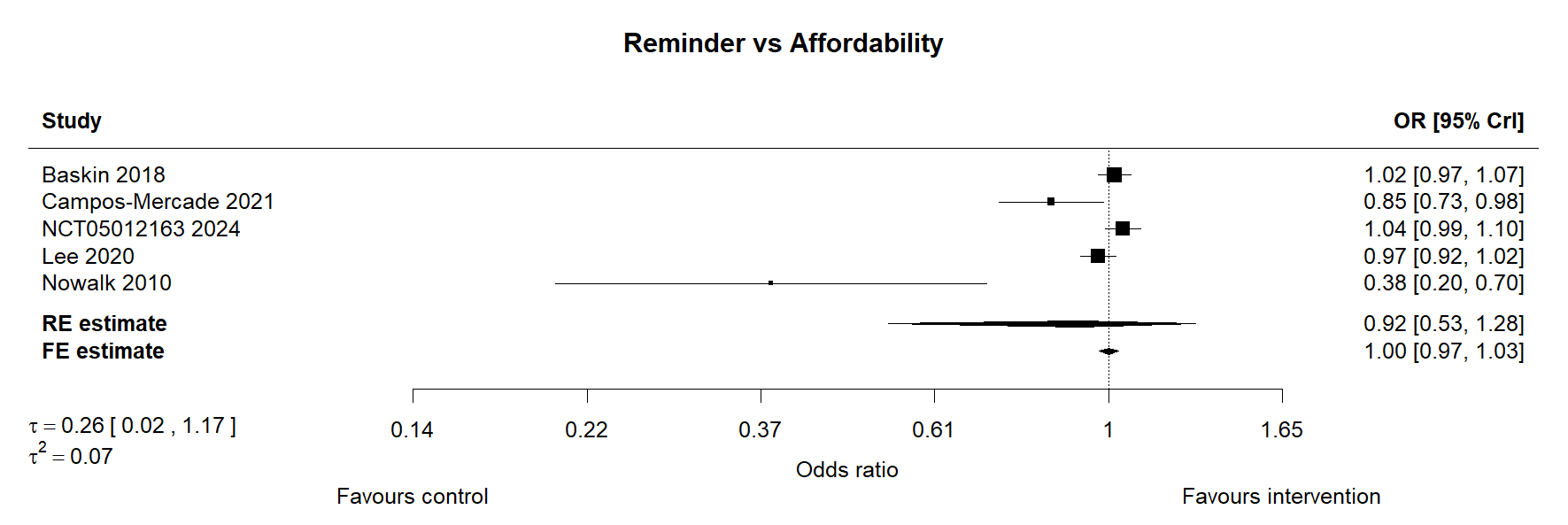

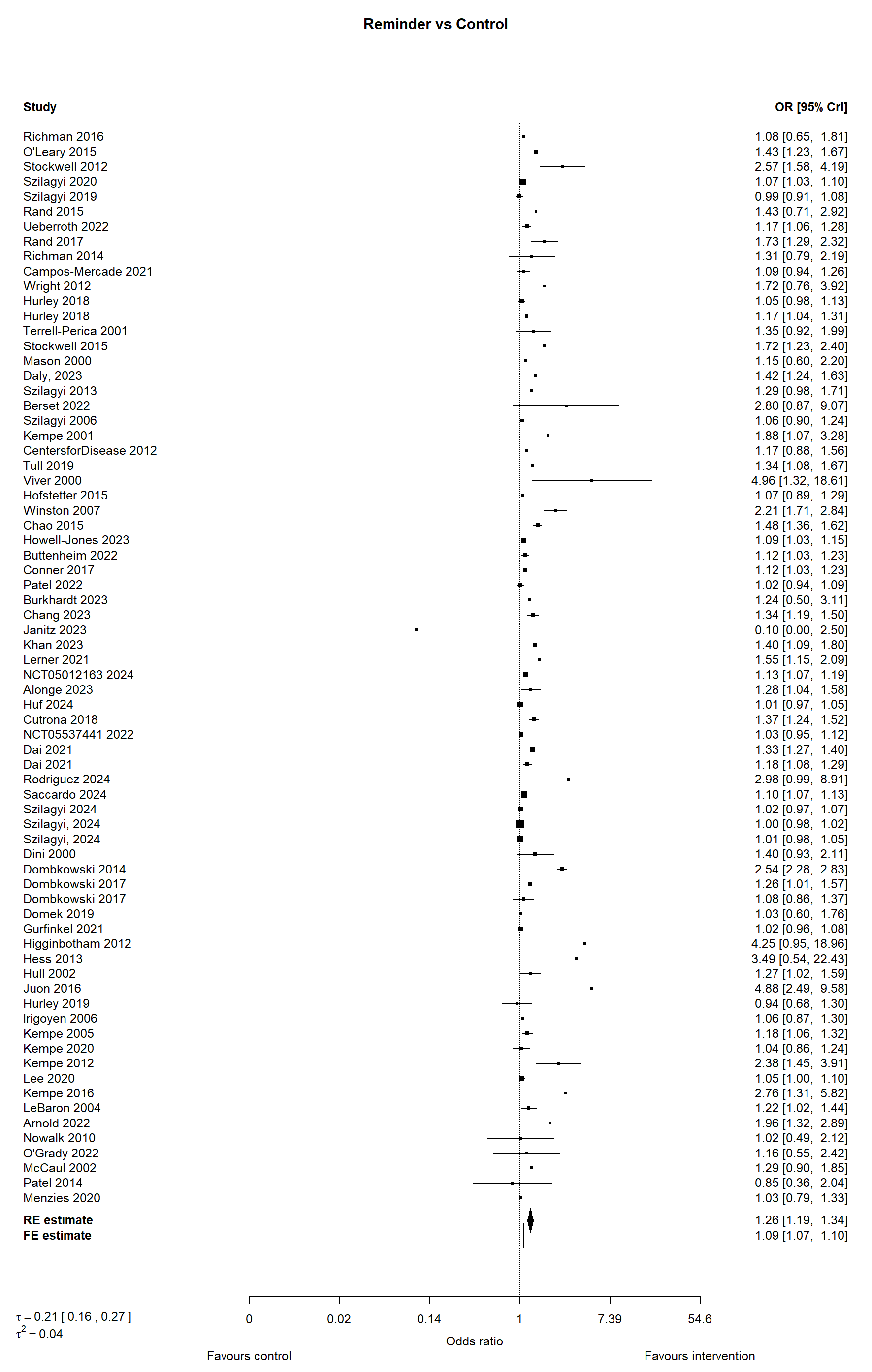

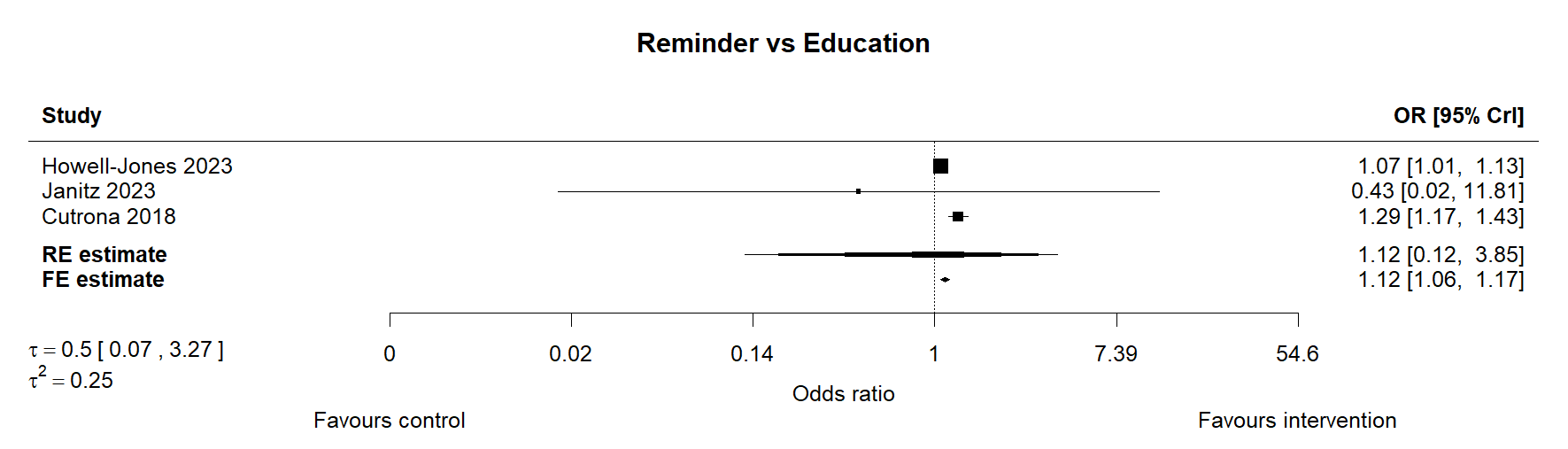
**

## **R: Funnel plots to look for small study effects from pairwise MAs with five or more studies**


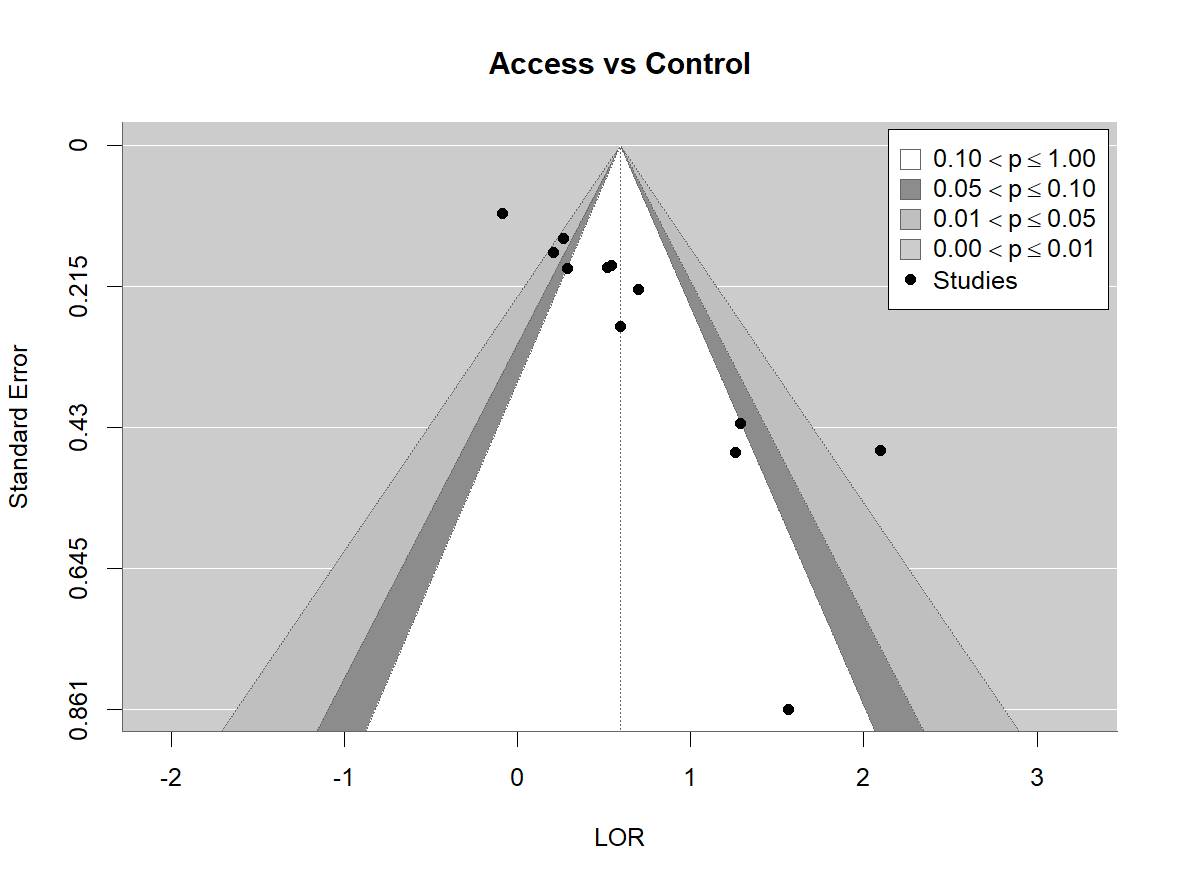


Test for Funnel Plot Asymmetry: z = 6.2128, p < .0001


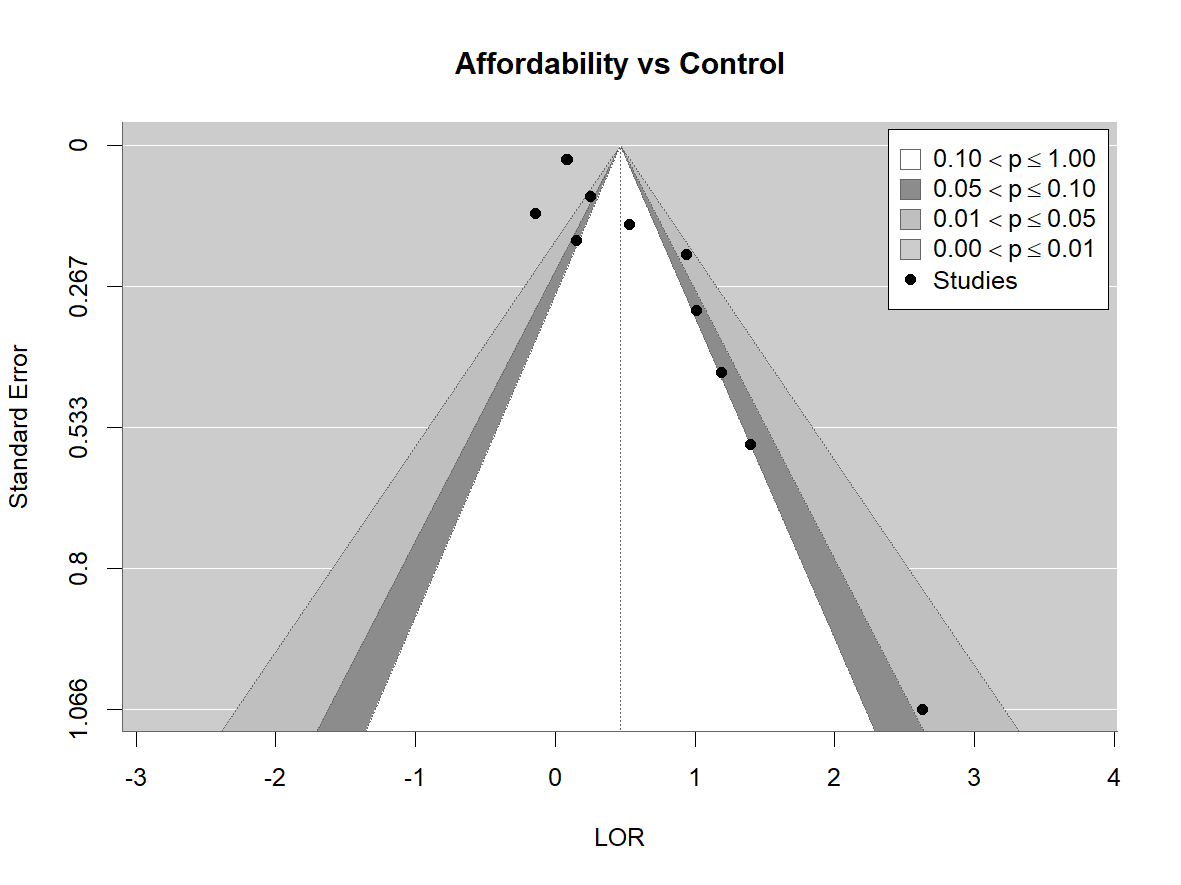


Test for Funnel Plot Asymmetry: z = 5.9947, p < .0001


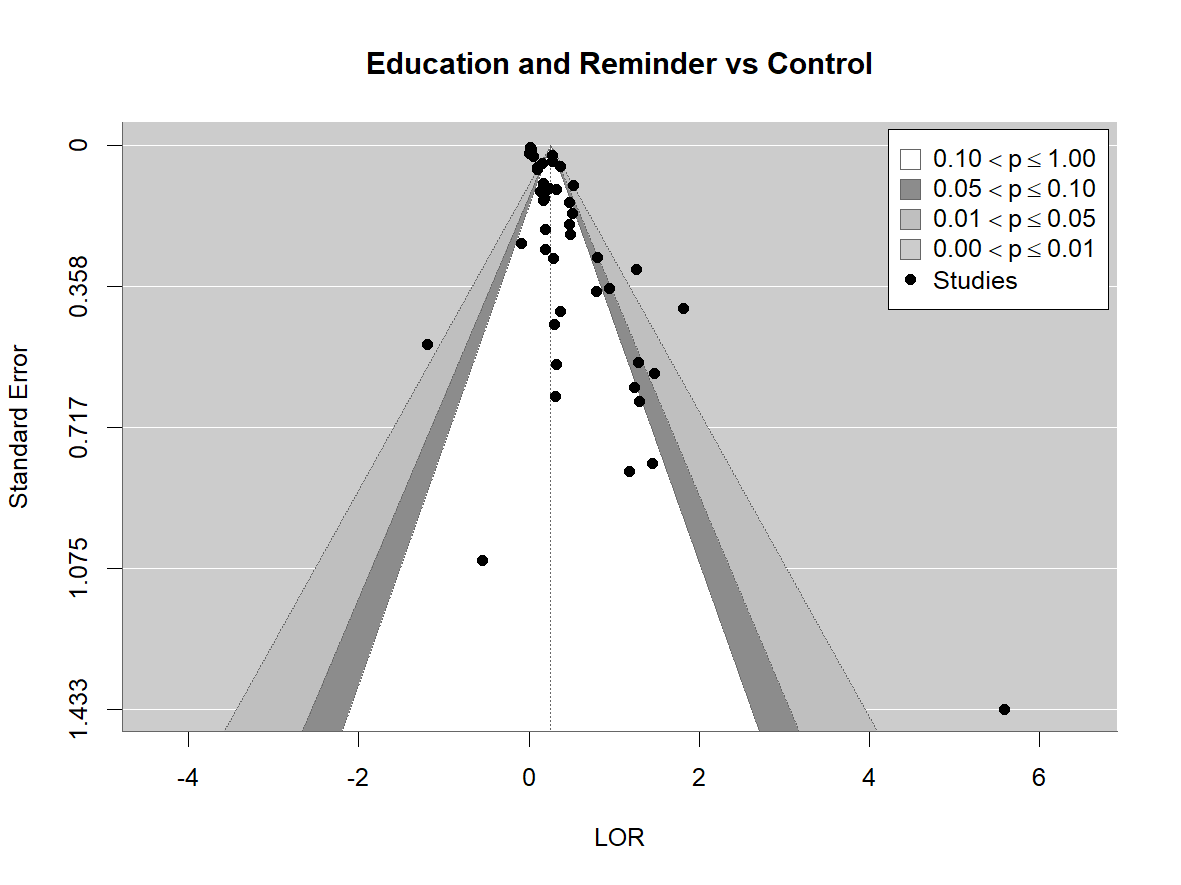


Test for Funnel Plot Asymmetry: z = 6.3171, p < .0001


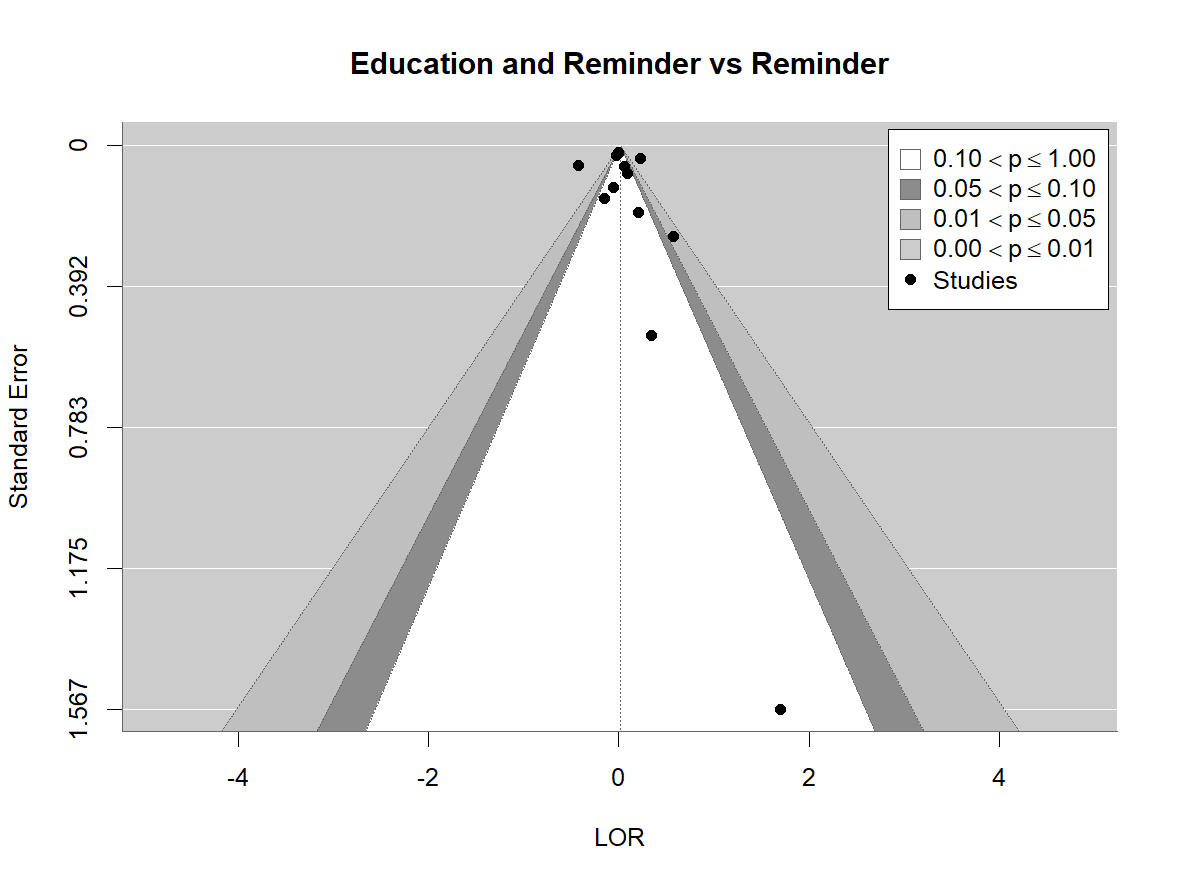


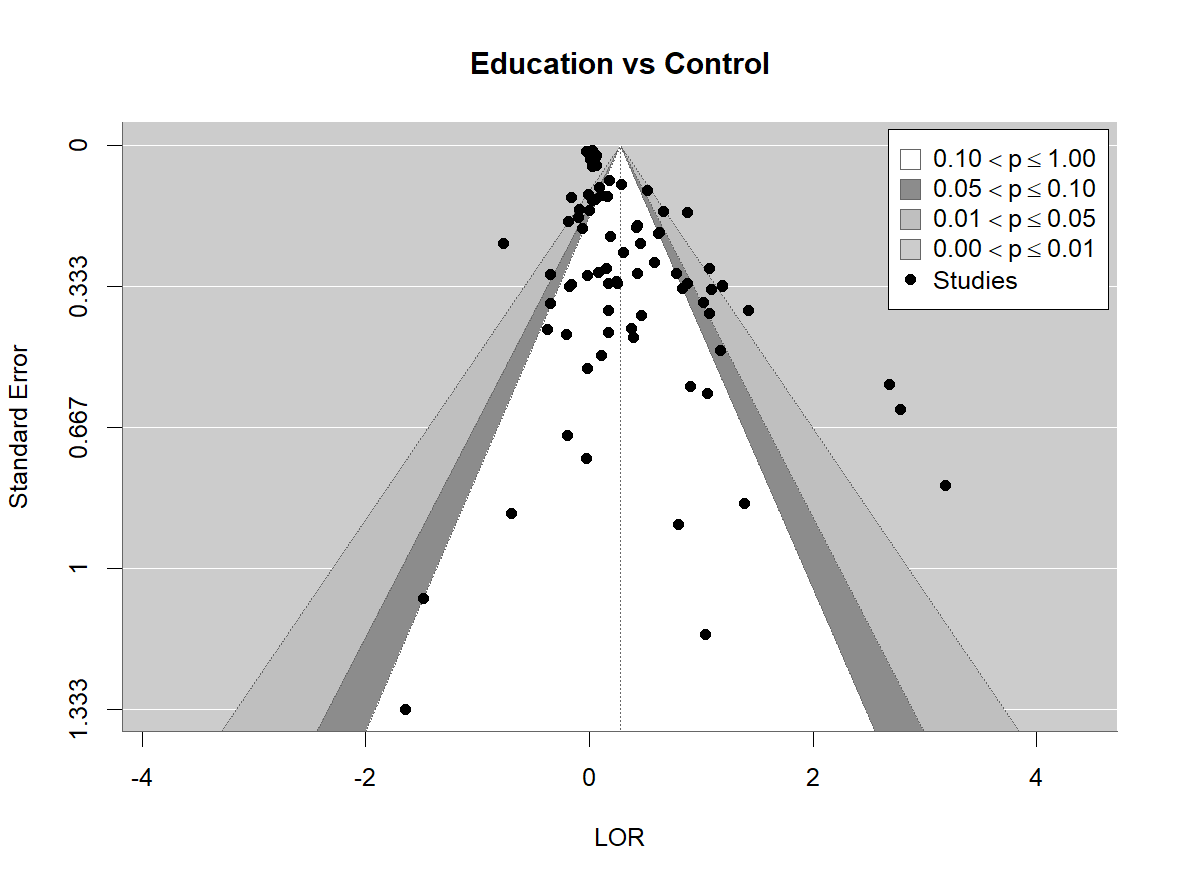


Test for Funnel Plot Asymmetry: z = 4.1816, p < .0001


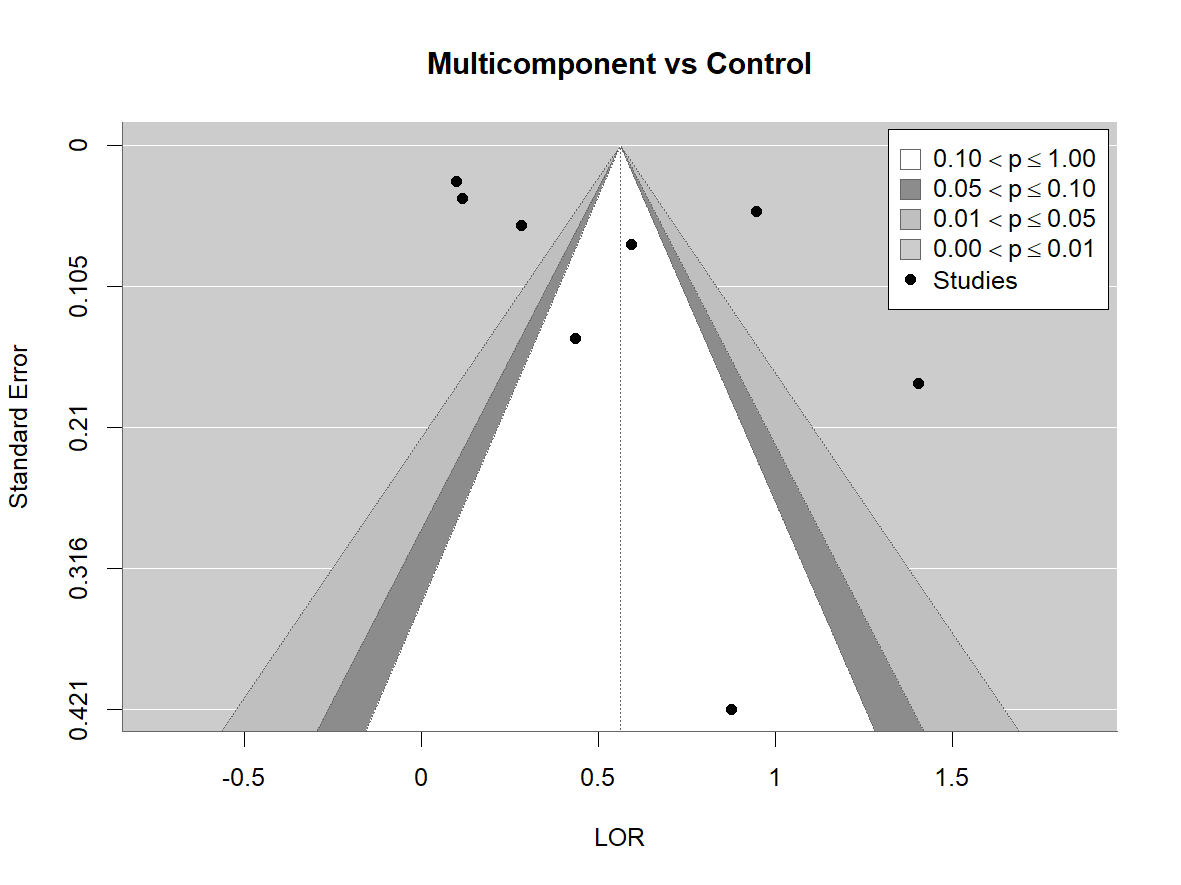


Test for Funnel Plot Asymmetry: z = 1.4376, p = 0.1505


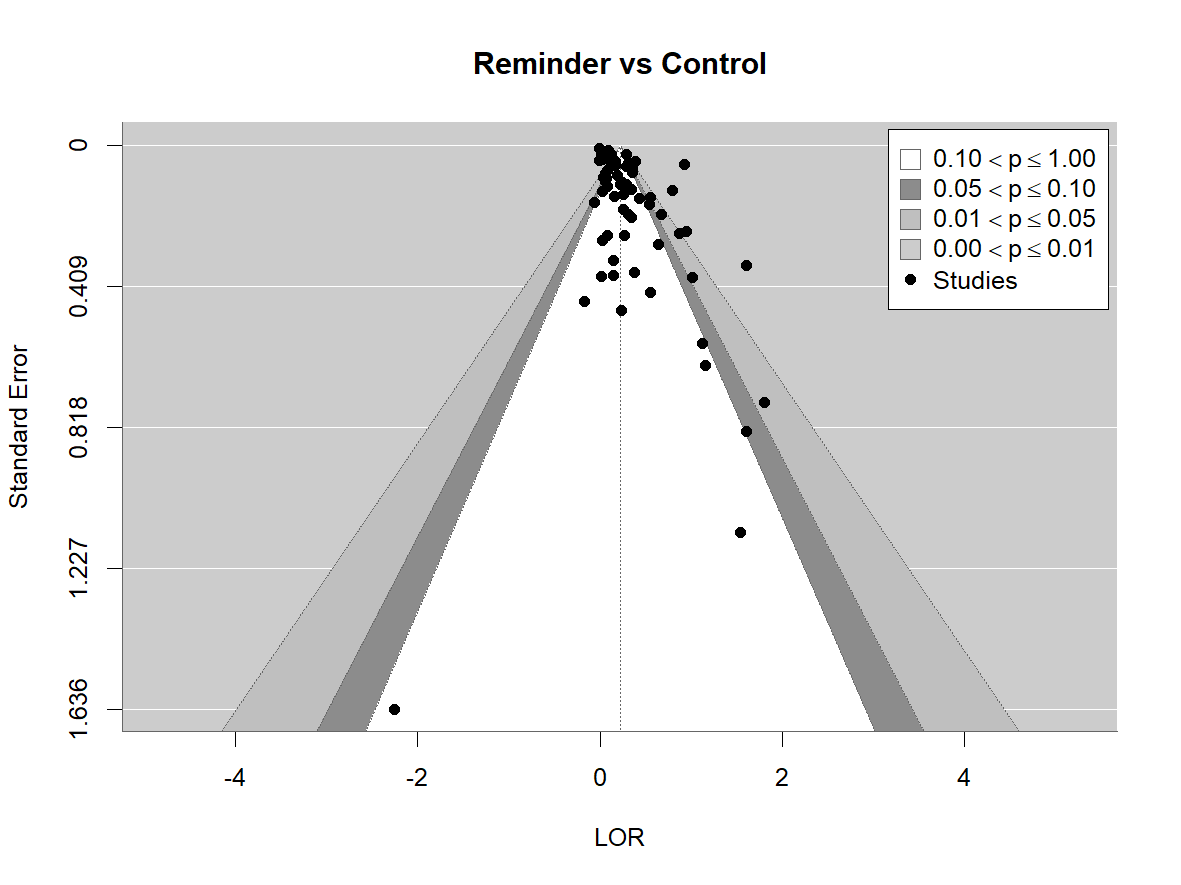


Test for Funnel Plot Asymmetry: z = 4.6575, p < .0001

## **S: Sensitivity analyses**

### *Sensitivity analyses: Fixed effects*


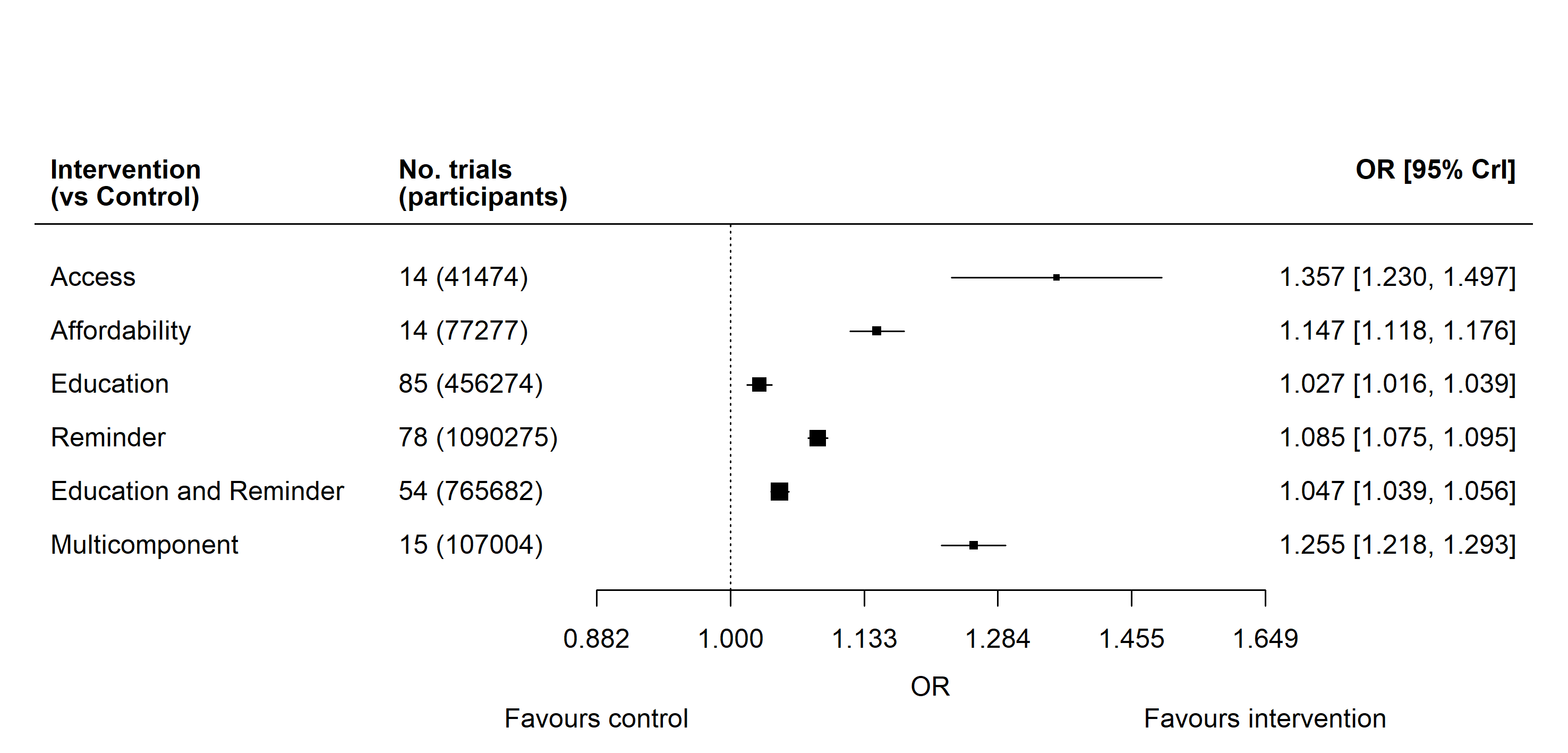


### *Sensitivity analyses: Removal of studies at high risk of bias*
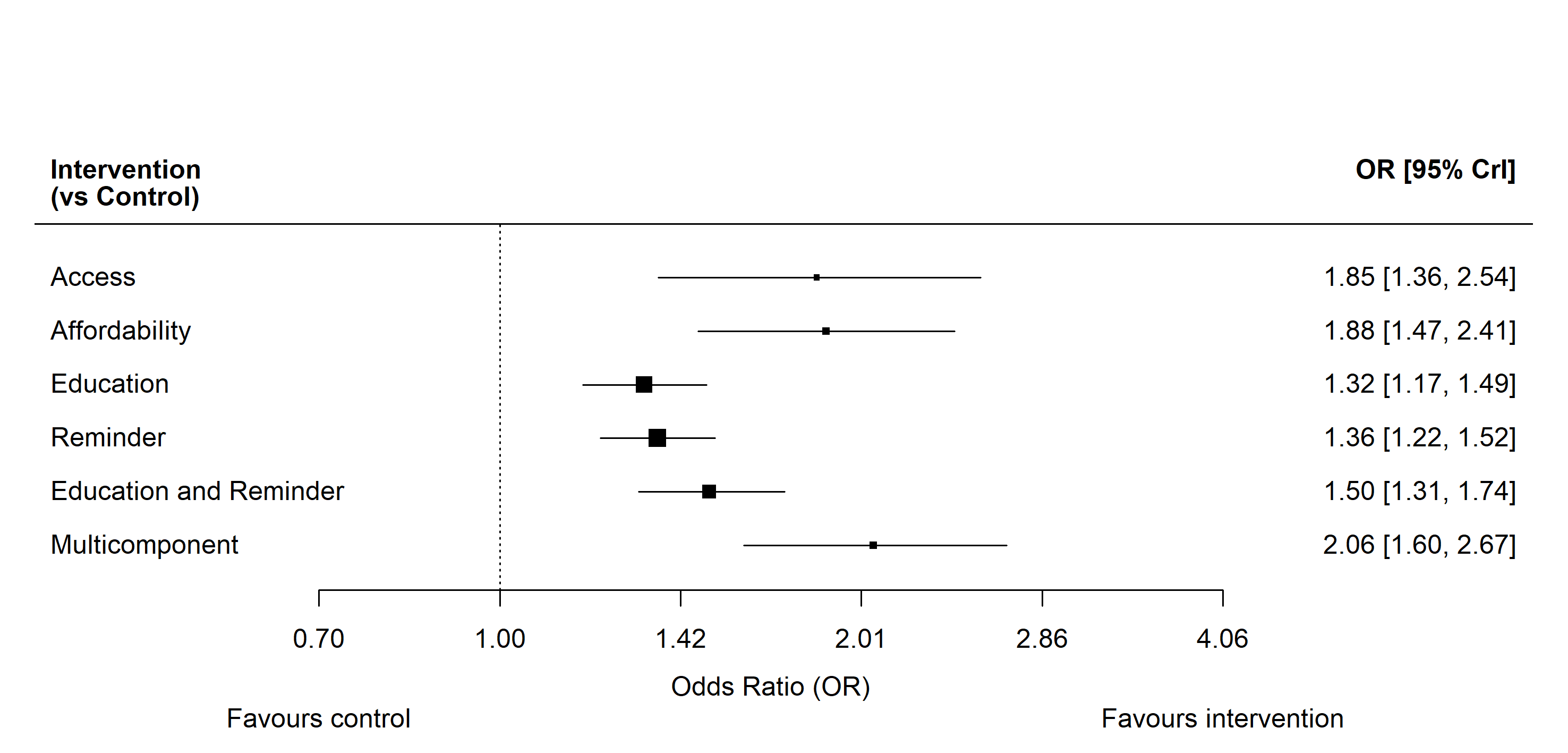


### *Sensitivity analyses: Removal of outliers*

We classified strongly outlying results as those with deviances greater than 2.7. Assuming deviances follow a chi-squared distribution, the probability of a data point having a deviance >2.7 is approximately 10%. Excluding these outliers resulted in the removal of 5 studies (Ferreira 2022, Ma 2018, Ma 2022, Masson 2013, and Scarinci 2020).


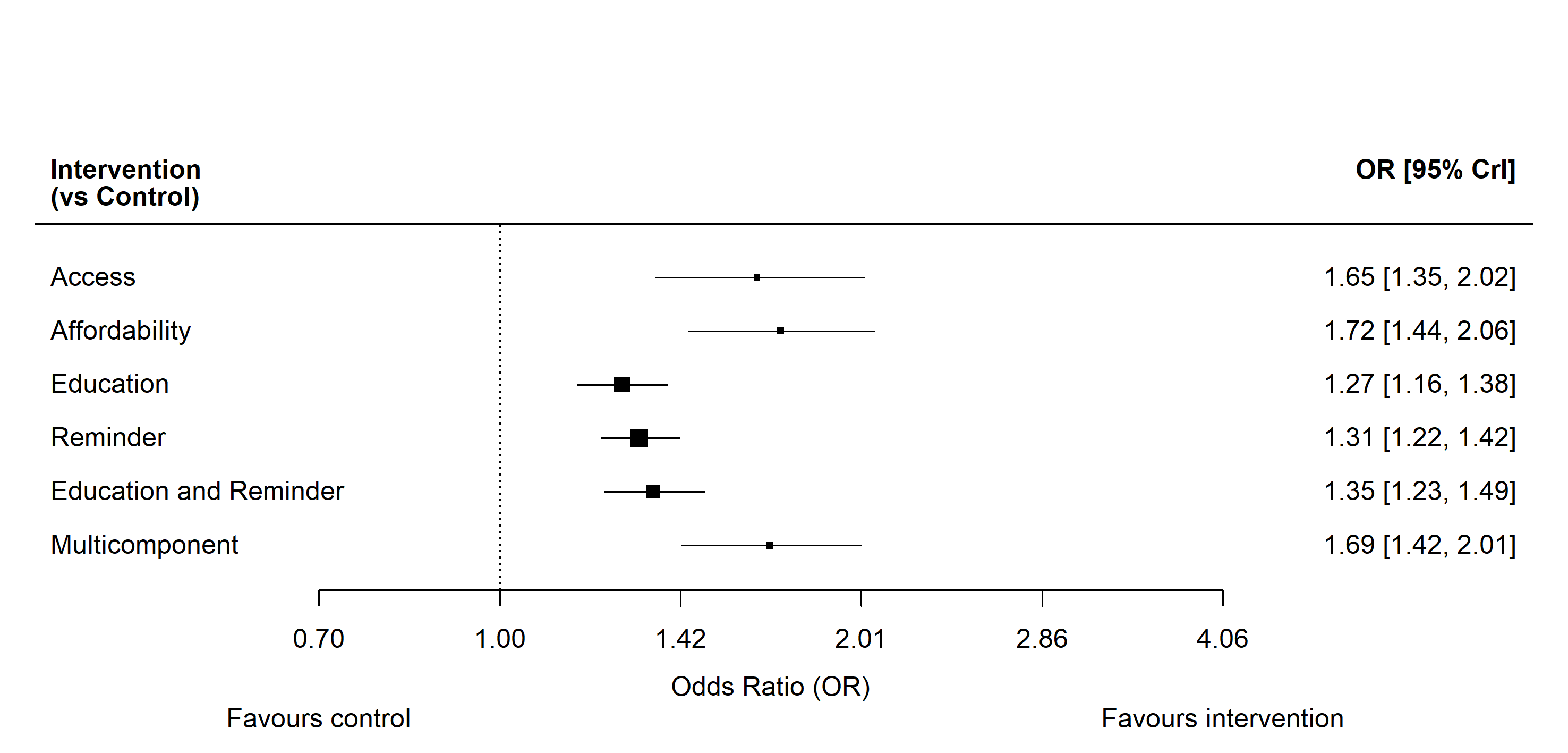


### *Sensitivity analysis: No cluster adjustments*


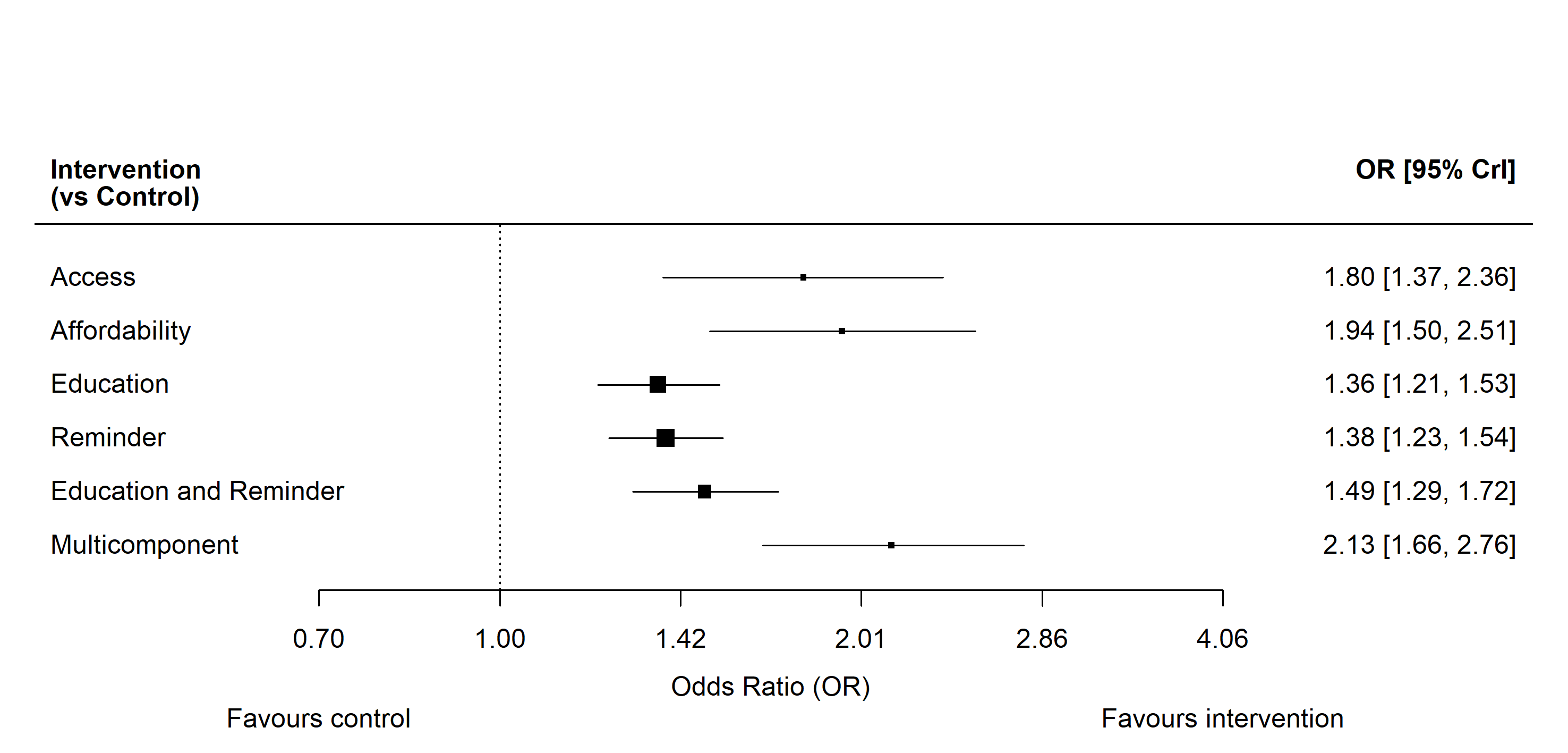


### *Sensitivity analysis: High ICC - larger ICC values (1 for households and 0.3 otherwise)*


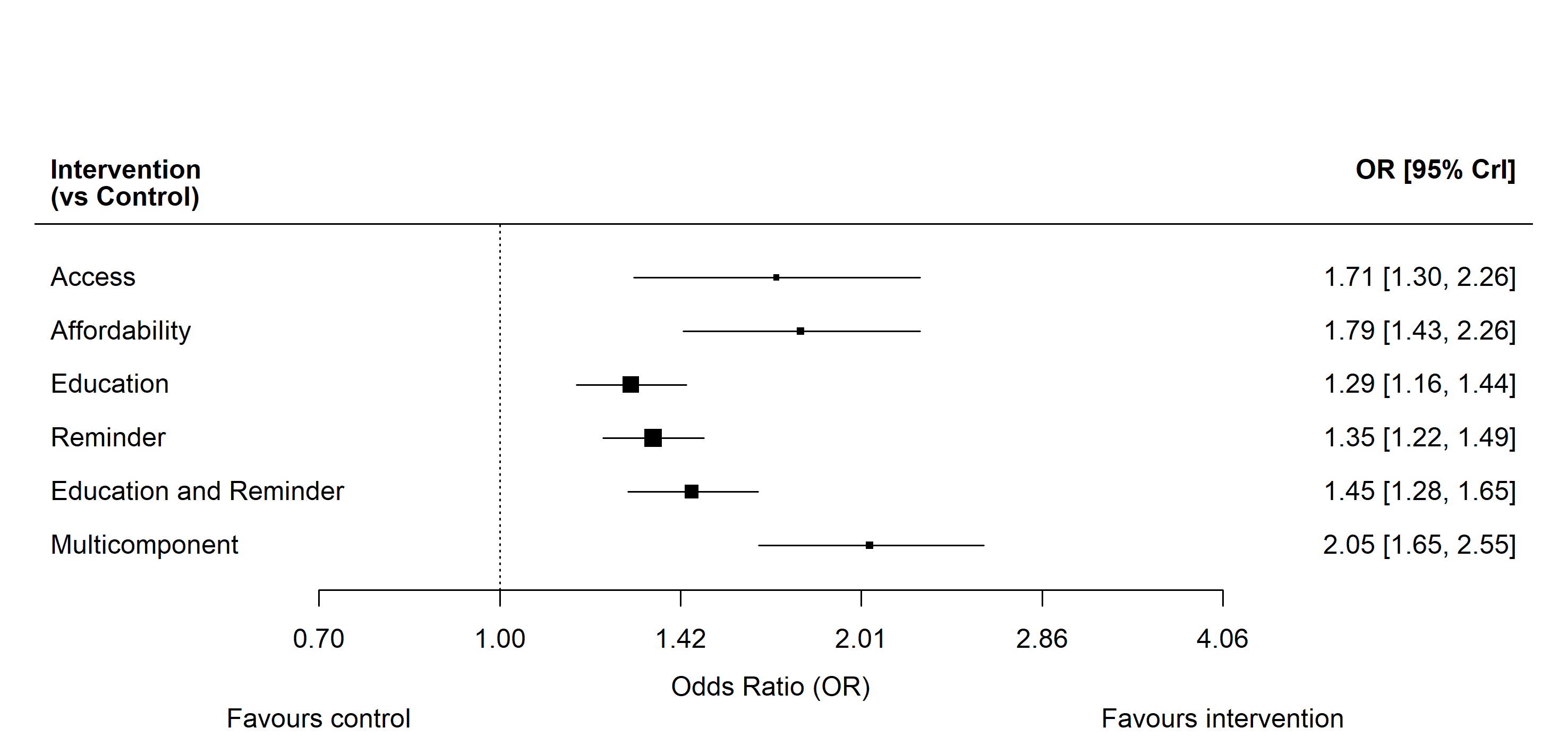


## **T: References to included studies**

1. Abroms LC, Koban D, Krishnan N, et al. Empathic Engagement With the COVID-19 Vaccine Hesitant in Private Facebook Groups: A Randomized Trial. *Health Educ Behav* 2023: 10901981231188313.

2. Alonge OD, Hanson KE, Eggebrecht M, et al. COVID-19 Booster Dose Reminder/Recall for Adolescents: Findings From a Health-Care System in Wisconsin. *J Adolesc Health* 2023; **73**(5): 953-6.

3. Anraad C, van Empelen P, Ruiter RAC, van Keulen H. Effects of an online tailored decision aid to promote informed decision making about maternal pertussis vaccination in the Netherlands: A randomized controlled trial. *Vaccine* 2023; **41**(49): 7348-58.

4. Aref HAT, Westrick S, Chou C, Worthington D, Garza K. How to inform college students about meningitis B vaccine? Comparative effectiveness of an online theory-based text and video intervention. *J Am Coll Health* 2022: 1-10.

5. Arnold JN, Gundlach N, Böckelmann I, Sammito S. Randomised Controlled Study on Measures to Increase Vaccination Rates among German Armed Forces Soldiers. *International journal of environmental research and public health* 2022; **19**(14).

6. Arthur AJ, Matthews RJ, Jagger C, Clarke M, Hipkin A, Bennison DP. Improving uptake of influenza vaccination among older people: a randomised controlled trial. *Br J Gen Pract* 2002; **52**(482): 717-8, 20.

7. Bartos V, Bauer M, Cahlikova J, Chytilova J. C ommunicating doctors' consensus persistently increases COVID-19 vaccinations. *Nature* 2022; **606**(7914): 542-9.

8. Bartu A, Sharp J, Ludlow J, Doherty DA. Postnatal home visiting for illicit drug-using mothers and their infants: a randomised controlled trial. *Aust N Z J Obstet Gynaecol* 2006; **46**(5): 419-26.

9. Baskin E. Increasing influenza vaccination rates via low cost messaging interventions. *PLoS One* 2018; **13**(2): 9.

10. Bastani R, Glenn BA, Singhal R, et al. Increasing HPV Vaccination among Low-Income, Ethnic Minority Adolescents: effects of a Multicomponent System Intervention through a County Health Department Hotline. *Cancer epidemiology, biomarkers & prevention* 2022; **31**(1): 175‐82.

11. Bennett AT, Patel DA, Carlos RC, et al. Human Papillomavirus Vaccine Uptake After a Tailored, Online Educational Intervention for Female University Students: A Randomized Controlled Trial. *J Womens Health (Larchmt)* 2015; **24**(11): 950-7.

12. Berg GD, Silverstein S, Thomas E, Korn AM. Cost and utilization avoidance with mail prompts: a randomized controlled trial. *Am J Manag Care* 2008; **14**(11): 748-54.

13. Berg GD, Thomas E, Silverstein S, Neel CL, Mireles M. Reducing medical service utilization by encouraging vaccines: randomized controlled trial. *Am J Prev Med* 2004; **27**(4): 284-8.

14. Berkhout C, Willefert-Bouche A, Chazard E, et al. Randomized controlled trial on promoting influenza vaccination in general practice waiting rooms. *PLoS ONE* 2018; **13**(2): e0192155.

15. Bernard-Genest MP, Ruel-Laliberte J, Lapointe-Milot K. Effect of educative reminder telephone calls on human papillomavirus immunization rate: A randomized controlled trial. *Womens Health (Lond Engl)* 2021; **17**: 17455065211003821.

16. Berset AE, Burkhardt MC, Xu Y, Mescher A, Brinkman WB. Effect of Electronic Outreach Using Patient Portal Messages on Well Child Care Visit Completion: A Randomized Clinical Trial. *JAMA Network Open* 2022; **5**(11): e2242853-e.

17. Berset AE, Burkhardt MC, Xu Y, Mescher A, Brinkman WB. Effect of Automated and Personalized Outreach Messages on Well-Child Visit Catch Up: A Randomized Clinical Trial. *Acad Pediatr* 2023; **23**: 23.

18. Bethke N, O'Sullivan JL, Keller J, von Bernuth H, Gellert P, Seybold J. Increasing vaccinations through an on-site school-based education and vaccination program: A city-wide cluster randomized controlled trial. *Appl Psychol Health Well Being* 2024; **01**: 01.

19. Bian J, Guo Z, Zhang W, et al. College students' influence on COVID-19 vaccination uptake among seniors in China: a protocol of combined cross-sectional and experimental study. *BMC Public Health* 2023; **23**(1): 1322.

20. Bocquier A, Michel M, Giraudeau B, et al. Impact of a school-based and primary care-based multicomponent intervention on HPV vaccination coverage among French adolescents: a cluster randomised controlled trial protocol (the PrevHPV study). *BMJ Open* 2022; **12**(3): e057943.

21. Borg K, Sutton K, Beasley M, et al. Communication-based interventions for increasing influenza vaccination rates among Aboriginal children: a randomised controlled trial. *Vaccine* 2018; **36**(45): 6790‐5.

22. Bourgeois FT, Simons WW, Olson K, Brownstein JS, Mandl KD. Evaluation of influenza prevention in the workplace using a personally controlled health record: randomized controlled trial. *J Med Internet Res* 2008; **10**(1): e5.

23. Bowman SE. Hepatitis B vaccination at syringe exchange programs in three US cities: Vaccine efficacy for the standard versus accelerated dosing schedules and determinants of completing the vaccination series among active injection drug users. United States -- Connecticut: Yale University; 2010.

24. Brigham KS, Woods ER, Steltz SK, Sandora TJ, Blood EA. Randomized controlled trial of an immunization recall intervention for adolescents. *Pediatrics* 2012; **130(3)**: 507-14.

25. Bronchetti ET, Huffman DB, Magenheim E. Attention, intentions, and follow-through in preventive health behavior: Field experimental evidence on flu vaccination. *Journal of Economic Behavior & Organization* 2015; **116**: 270-91.

26. Burkhardt MC, Berset AE, Xu Y, Mescher A, Brinkman WB. Effect of Outreach Messages on Adolescent Well Child Visits and COVID-19 Vaccine Rates: An RCT. *J Pediatr* 2022; **03**: 03.

27. Burkhardt MC, Berset AE, Xu Y, Mescher A, Brinkman WB. Effect of Outreach Messages on Adolescent Well-Child Visits and Coronavirus Disease 2019 Vaccine Rates: A Randomized, Controlled Trial. *J Pediatr* 2023; **253**: 158-64.e1.

28. Buttenheim A, Milkman KL, Duckworth AL, Gromet DM, Patel M, Chapman G. Effects of Ownership Text Message Wording and Reminders on Receipt of an Influenza Vaccination: A Randomized Clinical Trial. *JAMA netw* 2022; **5**(2): e2143388.

29. Cameron KA, Roloff ME, Friesema EM, et al. Patient knowledge and recall of health information following exposure to "facts and myths" message format variations. *Patient Educ Couns* 2013; **92**(3): 381-7.

30. Campos-Mercade P, Meier AN, Schneider FH, Meier S, Pope D, Wengstrom E. Monetary incentives increase COVID-19 vaccinations. *Science* 2021; **374**(6569): 879-+.

31. Cataldi JR, Suresh K, Brewer SE, et al. Boot Camp Translation using Community-Engaged messaging for adolescent Vaccination: a Cluster-Randomized trial. *Vaccine* 2024; **42**(5): 1078‐86.

32. Centers for Disease C, Prevention. Evaluation of vaccination recall letter system for Medicaid-enrolled children aged 19-23 months--Montana, 2011. *MMWR Morb Mortal Wkly Rep* 2012; **61**(40): 811-5.

33. Chai SJ, Tan F, Ji Y, Wei X, Li R, Frost M. Community-level text messaging for 2009 H1N1 prevention in China. *American journal of preventive medicine* 2013; **45**(2): 190‐6.

34. Chang TY, Jacobson M, Shah M, Kopetsky M, Pramanik R, Shah SB. Reminders, but not monetary incentives, increase COVID-19 booster uptake. *Proc Natl Acad Sci U S A* 2023; **120**(31): e2302725120.

35. Chao C, Preciado M, Slezak J, Xu LF. A Randomized Intervention of Reminder Letter for Human Papillomavirus Vaccine Series Completion. *J Adolesc Health* 2015; **56**(1): 85-90.

36. Chodick G, Teper GR, Levi S, et al. The impact of a Facebook campaign among mothers on HPV vaccine uptake among their daughters: A randomized field study. *Gynecol Oncol* 2021; **160**(1): 106-11.

37. Clayton K, Finley C, Flynn DJ, Graves M, Nyhan B. Evaluating the effects of vaccine messaging on immunization intentions and behavior: Evidence from two randomized controlled trials in Vermont. *Vaccine* 2021; **39**(40): 5909-17.

38. Coley S, Hoefer D, Rausch-Phung E. A population-based reminder intervention to improve human papillomavirus vaccination rates among adolescents at routine vaccination age. *Vaccine* 2018; **36**(32): 4904-9.

39. Conner M, Sandberg T, Nekitsing C, et al. Varying cognitive targets and response rates to enhance the question-behaviour effect: An 8-arm Randomized Controlled Trial on influenza vaccination uptake. *Social Science & Medicine* 2017; **180**: 135-42.

40. Cox AD, Cox D, Cyrier R, Graham-Dotson Y, Zimet GD. Can Self-Prediction Overcome Barriers to Hepatitis B Vaccination? A Randomized Controlled Trial. *Health Psychol* 2012; **31**(1): 97-105.

41. Cutrona SL, Golden JG, Goff SL, et al. Improving Rates of Outpatient Influenza Vaccination Through EHR Portal Messages and Interactive Automated Calls: A Randomized Controlled Trial. *J Gen Intern Med* 2018; **33**(5): 659-67.

42. Dai H, Saccardo S, Han MA, et al. Behavioural nudges increase COVID-19 vaccinations. *Nature* 2021; **597**(7876): 404-9.

43. Dalby DM, Sellors JW, Fraser FD, Fraser C, van Ineveld C, Howard M. Effect of preventive home visits by a nurse on the outcomes of frail elderly people in the community: a randomized controlled trial. *Cmaj* 2000; **162**(4): 497-500.

44. Daley MF, Kempe A, Pyrzanowski J, et al. School-located vaccination of adolescents with insurance billing: cost, reimbursement, and vaccination outcomes. *J Adolesc Health* 2014; **54**(3): 282-8.

45. Daley MF, Steiner JF, Brayden RM, Xu S, Morrison S, Kempe A. Immunization registry-based recall for a new vaccine. *Ambulatory pediatrics* 2002; **2**(6): 438‐43.

46. Daniels NA, Juarbe T, Moreno-John G, Perez-Stable EJ. Effectiveness of adult vaccination programs in faith-based organizations. *Ethn Dis* 2007; **17**(1 Suppl 1): S15-22.

47. Dapp U, Anders JA, von Renteln-Kruse W, et al. A randomized trial of effects of health risk appraisal combined with group sessions or home visits on preventive behaviors in older adults. *J Gerontol A Biol Sci Med Sci* 2011; **66**(5): 591-8.

48. Davies C, Skinner SR, Stoney T, et al. 'Is it like one of those infectious kind of things?' the importance of educating young people about HPV and HPV vaccination at school. *Sex Education* 2017; **17**(3): 256-75.

49. DeCamp LR, Godage SK, Valenzuela Araujo D, et al. A Texting Intervention in Latino Families to Reduce ED Use: A Randomized Trial. *Pediatrics* 2020; **145**(1): 01.

50. Dempsey AF, Maertens J, Sevick C, Jimenez-Zambrano A, Juarez-Colunga E. A randomized, controlled, pragmatic trial of an iPad-based, tailored messaging intervention to increase human papillomavirus vaccination among Latinos. *Human Vaccines Immunother* 2019; **15**(7-8): 1577-84.

51. DiClemente RJ, Murray CC, Graham T, Still J. Overcoming barriers to HPV vaccination: A randomized clinical trial of a culturally-tailored, media intervention among African American girls. *Hum Vaccin Immunother* 2015; **11**(12): 2883-94.

52. Dini EF, Linkins RW, Sigafoos J. The impact of computer-generated messages on childhood immunization coverage. *Am J Prev Med* 2000; **18**(2): 132-9.

53. Dombkowski KJ, Costello LE, Harrington LB, Dong S, Kolasa M, Clark SJ. Age-specific strategies for immunization reminders and recalls: A registry-based randomized trial. *American Journal of Preventive Medicine* 2014; **47(1)**: 1-8.

54. Dombkowski KJ, Cowan AE, Reeves SL, Foley MR, Dempsey AF. The impacts of email reminder/recall on adolescent influenza vaccination. *Vaccine* 2017; **35**(23): 3089-95.

55. Domek GJ, Contreras-Roldan IL, Bull S, et al. Text message reminders to improve infant immunization in Guatemala: A randomized clinical trial. *Vaccine* 2019; **37**(42): 6192-200.

56. Doyle O, Fitzpatrick N, Lovett J, Rawdon C. Early intervention and child physical health: Evidence from a Dublin-based randomized controlled trial. *Econ Hum Biol* 2015; **19**: 224-45.

57. El-Mohandes AA, Katz KS, El-Khorazaty MN, et al. The effect of a parenting education program on the use of preventive pediatric health care services among low-income, minority mothers: a randomized, controlled study. *Pediatrics* 2003; **111**(6 Pt 1): 1324-32.

58. Ernsting A, Schwarzer R, Lippke S, Schneider M. 'I do not need a flu shot because I lead a healthy lifestyle': compensatory health beliefs make vaccination less likely. *J Health Psychol* 2013; **18**(6): 825-36.

59. Esposito S, Bianchini S, Tagliabue C, et al. Impact of a website based educational program for increasing vaccination coverage among adolescents. *Hum Vaccin Immunother* 2018; **14**(4): 961-8.

60. Fernandez ME, Savas LS, Atkinson JS, et al. Evaluation of a 2-1-1 Telephone Navigation Program to Increase Cancer Control Behaviors: Results From a Randomized Controlled Trial. *Am J Health Promot* 2022; **36**(7): 1083-93.

61. Ferreira H, Siqueira CM, Sousa LB, et al. Effect of educational intervention for compliance of school adolescents with the human papillomavirus vaccine. *Revista da Escola de Enfermagem da U S P* 2022; **56**: e20220082.

62. Fiks AG, Grundmeier RW, Mayne S, et al. Effectiveness of decision support for families, clinicians, or both on HPV vaccine receipt. *Pediatrics* 2013; **131(6)**: 1114-24.

63. Fitzpatrick T, Zhou KL, Cheng Y, et al. A crowdsourced intervention to promote hepatitis B and C testing among men who have sex with men in China: study protocol for a nationwide online randomized controlled trial. *BMC Infect Dis* 2018; **18**: 9.

64. Frew PM, Kriss JL, Chamberlain AT, et al. A randomized trial of maternal influenza immunization decision-making: A test of persuasive messaging models. *Human Vaccines and Immunotherapeutics* 2016; **12(8)**: 1989-96.

65. Frew PM, Saint-Victor DS, Owens LE, Omer SB. Socioecological and message framing factors influencing maternal influenza immunization among minority women. *Vaccine* 2014; **32**(15): 1736-44.

66. Galasso V, Pons V, Profeta P, et al. Addressing vaccine hesitancy: experimental evidence from nine high-income countries during the COVID-19 pandemic. *BMJ glob* 2023; **8**(9): 09.

67. Gerend MA, Madkins K, Crosby S, et al. Evaluation of a Text Messaging-Based Human Papillomavirus Vaccination Intervention for Young Sexual Minority Men: Results from a Pilot Randomized Controlled Trial. *Ann Behav Med* 2021; **55**(4): 321-32.

68. Gerend MA, Shepherd JE. Predicting Human Papillomavirus Vaccine Uptake in Young Adult Women: Comparing the Health Belief Model and Theory of Planned Behavior. *Ann Behav Med* 2012; **44**(2): 171-80.

69. Glanz JM, Wagner NM, Narwaney KJ, et al. Web-based Social Media Intervention to Increase Vaccine Acceptance: A Randomized Controlled Trial. *Pediatrics* 2017; **140**(6): 1-9.

70. Glanz JM, Wagner NM, Narwaney KJ, et al. Web-Based Tailored Messaging to Increase Vaccination: A Randomized Clinical Trial. *Pediatrics* 2020; **146**(5): 1-10.

71. Goodman K, Mossad SB, Taksler GB, Emery J, Schramm S, Rothberg MB. Impact of Video Education on Influenza Vaccination in Pregnancy. *J Reprod Med* 2015; **60**(11-12): 471-9.

72. Goodyear-Smith F, Grant C, Poole T, et al. Early connections: effectiveness of a pre-call intervention to improve immunisation coverage and timeliness. *J Prim Health Care* 2012; **4**(3): 189-98.

73. Grandahl M, Rosenblad A, Stenhammar C, et al. School-based intervention for the prevention of HPV among adolescents: a cluster randomised controlled study. *BMJ Open* 2016; **6**(1): e009875.

74. Green B, Sanders MB, Tarte JM. Effects of Home Visiting Program Implementation on Preventive Health Care Access and Utilization: Results from a Randomized Trial of Healthy Families Oregon. *Prev Sci* 2020; **21**(1): 15-24.

75. Gurfinkel D, Kempe A, Albertin C, et al. Centralized reminder/recall for human papillomavirus vaccination: Findings from two states-A randomized clinical trial. *Journal of Adolescent Health* 2021; **69**(4): 579-87.

76. Haff N, Choudhry NK, Bhatkhande G, et al. "How" Versus "Why" Messaging to Increase Uptake of Booster Vaccination Against COVID-19: Results of a Pragmatic Randomized Trial. *J Gen Intern Med* 2023; **06**: 06.

77. Hambidge SJ, Phibbs SL, Chandramouli V, Fairclough D, Steiner JF. A stepped intervention increases well-child care and immunization rates in a disadvantaged population. *Pediatrics* 2009; **124**(2): 455-64.

78. Hanley K, Chung TH, Nguyen LK, et al. Using Electronic Reminders to Improve Human Papillomavirus (HPV) Vaccinations among Primary Care Patients. *Vaccines* 2023; **11(4) (no pagination)**.

79. Hannan J. APN telephone follow up to low-income first time mothers. *J Clin Nurs* 2013; **22**(1-2): 262-70.

80. Harari D, Iliffe S, Kharicha K, et al. Promotion of health in older people: a randomised controlled trial of health risk appraisal in British general practice. *Age Ageing* 2008; **37**(5): 565-71.

81. Henrikson NB, Zhu W, Baba L, et al. Outreach and Reminders to Improve Human Papillomavirus Vaccination in an Integrated Primary Care System. *Clin Pediatr (Phila)* 2018; **57**(13): 1523-31.

82. Hess R. Impact of automated telephone messaging on zoster vaccination rates in community pharmacies. *J Am Pharm Assoc (2003)* 2013; **53**(2): 182-7.

83. Higginbotham S, Stewart A, Pfalzgraf A. Impact of a pharmacist immunizer on adult immunization rates. *J Am Pharm Assoc (2003)* 2012; **52**(3): 367-71.

84. Ho HJ, Tan YR, Cook AR, et al. Increasing Influenza and Pneumococcal Vaccination Uptake in Seniors Using Point-of-Care Informational Interventions in Primary Care in Singapore: A Pragmatic, Cluster-Randomized Crossover Trial. *Am J Public Health* 2019; **109**(12): 1776-83.

85. Hofstetter AM, DuRivage N, Vargas CY, et al. Text message reminders for timely routine MMR vaccination: A randomized controlled trial. *Vaccine* 2015; **33**(43): 5741-6.

86. Hofstetter AM, Vargas CY, Camargo S, et al. Impacting delayed pediatric influenza vaccination: a randomized controlled trial of text message reminders. *American journal of preventive medicine* 2015; **48**(4): 392‐401.

87. Hopfer S. Effects of a narrative HPV vaccination intervention aimed at reaching college women: a randomized controlled trial. *Prev Sci* 2012; **13**(2): 173-82.

88. Howell-Jones R, Gold N, Bowen S, et al. Can uptake of childhood influenza immunisation through schools and GP practices be increased through behaviourally-informed invitation letters and reminders: two pragmatic randomized controlled trials. *BMC Public Health* 2023; **23**(1): 143.

89. Hu PL, Koh EYL, Tay JSH, Chan VX, Goh SSM, Wang SZ. Assessing the impact of educational methods on influenza vaccine uptake and patient knowledge and attitudes: a randomised controlled trial. *Singapore Med J* 2021; **15**: 15.

90. Hu Y, Chen Y, Wang Y, Song Q, Li Q. Prenatal vaccination education intervention improves both the mothers' knowledge and children's vaccination coverage: Evidence from randomized controlled trial from eastern China. *Hum Vaccin Immunother* 2017; **13**(6): 1-8.

91. Hu Y, Li Q, Chen Y. Evaluation of two health education interventions to improve the varicella vaccination: a randomized controlled trial from a province in the east China. *BMC Public Health* 2018; **18**(1): 144.

92. Huf SW, Grailey K, Crespo RF, et al. Testing the impact of differing behavioural science informed text message content in COVID-19 vaccination invitations on vaccine uptake: A randomised clinical trial. *Vaccine* 2024; **28**: 28.

93. Hull S, Hagdrup N, Hart B, Griffiths C, Hennessy E. Boosting uptake of influenza immunisation: a randomised controlled trial of telephone appointing in general practice. *Br J Gen Pract* 2002; **52**(482): 712-6.

94. Humiston SG, Schaffer SJ, Szilagyi PG, et al. Seasonal influenza vaccination at school: a randomized controlled trial. *Am J Prev Med* 2014; **46**(1): 1-9.

95. Hurley LP, Beaty B, Lockhart S, et al. RCT of Centralized Vaccine Reminder/Recall for Adults. *Am J Prev Med* 2018; **55**(2): 231-9.

96. Hurley LP, Beaty B, Lockhart S, et al. Randomized controlled trial of centralized vaccine reminder/recall to improve adult vaccination rates in an accountable care organization setting. *Preventive medicine reports* 2019; **15**.

97. Hurtaud A, Coomans C, Vuillemin B, et al. Impact of a dTcaP booster vaccine awareness campaign initiated by the French national health insurance for adults aged 25 years in 2021. *BMC Health Serv Res* 2023; **23**(1): 903.

98. Hutchison RL, O'Rear J, Olson-Burgess C, Myers AL. Offering the Influenza Vaccine in a Pediatric Hand Surgery Clinic Increases Vaccination Rates. *J Hand Surg [Am]* 2018; **43**(8): 776.e1-.e4.

99. Hwang LY, Grimes CZ, Tran TQ, et al. Accelerated hepatitis B vaccination schedule among drug users: a randomized controlled trial. *J Infect Dis* 2010; **202**(10): 1500-9.

100. Irigoyen MM, Findley S, Wang D, et al. Challenges and Successes of Immunization Registry Reminders at Inner-City Practices. *Ambulatory Pediatrics* 2006; **6(2)**: 100-4.

101. Isrctn. The effects of monetary incentives on COVID-19 vaccination uptake. *https://trialsearchwhoint/Trial2aspx?TrialID=ISRCTN59503725* 2021.

102. Jackson C, Cheater FM, Harrison W, et al. Randomised cluster trial to support informed parental decision-making for the MMR vaccine. *BMC Public Health* 2011; **11**: 11.

103. Jacobson M, Chang TY, Shah M, Pramanik R, Shah SB. Can financial incentives and other nudges increase COVID-19 vaccinations among the vaccine hesitant? A randomized trial. *Vaccine* 2022: 6235‐42.

104. Janitz AE, Neil JM, Bray LA, et al. CATCH-UP vaccines: protocol for a randomized controlled trial using the multiphase optimization strategy (MOST) framework to evaluate education interventions to increase COVID-19 vaccine uptake in Oklahoma. *BMC Public Health* 2023; **23**(1): 1146.

105. Jiang M, Yao X, Li P, et al. Impact of video-led educational intervention on uptake of influenza vaccine among the elderly in western China: a community-based randomized controlled trial. *BMC Public Health* 2022; **22**(1): 1128.

106. Johansen ND, Vaduganathan M, Bhatt AS, et al. Electronic nudges to increase influenza vaccination uptake among patients with heart failure: a prespecified analysis of the NUDGE-FLU trial. *European journal of heart failure* 2023.

107. Johnson EA, Harwell TS, Donahue PM, et al. Promoting pneumococcal immunizations among rural Medicare beneficiaries using multiple strategies. *J Rural Health* 2003; **19**(4): 506-10.

108. Jordan ET, Bushar JA, Kendrick JS, Johnson P, Wang J. Encouraging Influenza Vaccination Among Text4baby Pregnant Women and Mothers. *Am J Prev Med* 2015; **49**(4): 563-72.

109. Joseph NP, Bernstein J, Pelton S, et al. Brief Client-Centered Motivational and Behavioral Intervention to Promote HPV Vaccination in a Hard-to-Reach Population. *Clinical Pediatrics* 2016; **55**(9): 851-9.

110. Ju Q, Xiao H, Peng H, Gan Y. How to Improve People's Intentions Regarding COVID-19 Vaccination in China: A Randomized Controlled Trial. *Int J Behav Med* 2024; **20**: 20.

111. Juon HS, Strong C, Kim F, Park E, Lee S. Lay Health Worker Intervention Improved Compliance with Hepatitis B Vaccination in Asian Americans: Randomized Controlled Trial. *PLoS ONE* 2016; **11**(9): e0162683.

112. Juraskova I, Bari RA, O'Brien MT, McCaffery KJ. HPV Vaccine Promotion: Does Referring to Both Cervical Cancer and Genital Warts Affect Intended and Actual Vaccination Behavior? *Womens Health Iss* 2011; **21**(1): 71-9.

113. Kasting ML, Head KJ, Cox D, Cox AD, Zimet GD. The effects of message framing and healthcare provider recommendation on adult hepatitis B vaccination: A randomized controlled trial. *Preventive Medicine* 2019; **127 (no pagination)**(105798).

114. Kempe A, Barrow J, Stokley S, et al. Effectiveness and cost of immunization recall at school-based health centers. *Pediatrics* 2012; **129**(6): e1446-52.

115. Kempe A, Daley MF, Barrow J, et al. Implementation of universal influenza immunization recommendations for healthy young children: results of a randomized, controlled trial with registry-based recall. *Pediatrics* 2005; **115**(1): 146-54.

116. Kempe A, Lowery NE, Pearson KA, et al. Immunization recall: effectiveness and barriers to success in an urban teaching clinic. *J Pediatr* 2001; **139**(5): 630-5.

117. Kempe A, O'Leary ST, Shoup JA, et al. Parental Choice of Recall Method for HPV Vaccination: A Pragmatic Trial. *Pediatrics* 2016; **137**(3): 26-.

118. Kempe A, Saville AW, Albertin C, et al. Centralized Reminder/Recall to Increase Influenza Vaccination Rates: A Two-State Pragmatic Randomized Trial. *Academic Pediatrics* 2020; **20**(3): 374-83.

119. Kerpelman LC, Connell DB, Gunn WJ. Effect of a monetary sanction on immunization rates of recipients of aid to families with dependent children. *Jama* 2000; **284**(1): 53-9.

120. Khan AA, Tran HN, Lai JA, et al. A Learning Health System Approach to Increasing Human Papillomavirus Immunizations Among Young Adults. *Perm* 2023; **27**(2): 31-6.

121. Kim M, Lee H, Kiang P, et al. A Storytelling Intervention in a Mobile, Web-Based Platform: A Pilot Randomized Controlled Trial to Evaluate the Preliminary Effectiveness to Promote Human Papillomavirus Vaccination in Korean American College Women. *Health Educ Behav* 2020; **47**(2): 258-63.

122. Krieger JW, Castorina JS, Walls ML, Weaver MR, Ciske S. Increasing influenza and pneumococcal immunization rates: a randomized controlled study of a senior center-based intervention. *Am J Prev Med* 2000; **18**(2): 123-31.

123. Kulle A-C, Schumacher S, Bieberstein Fv. Mobile vaccination units substantially increase COVID-19 vaccinations: evidence from a randomized controlled trial. *Journal of Public Health* 2024; **46**(1): 151-7.

124. Larsen BJ, Ryan TJ, Greene S, Hetherington MJ, Maxwell R, Tadelis S. Counter-stereotypical messaging and partisan cues: Moving the needle on vaccines in a polarized United States. *Sci Adv* 2023; **9**(29): eadg9434.

125. Lau AY, Sintchenko V, Crimmins J, Magrabi F, Gallego B, Coiera E. Impact of a web-based personally controlled health management system on influenza vaccination and health services utilization rates: a randomized controlled trial. *J Am Med Inform Assoc* 2012; **19**(5): 719-27.

126. LeBaron CW, Starnes DM, Rask KJ. The impact of reminder-recall interventions on low vaccination coverage in an inner-city population. *Arch Pediatr Adolesc Med* 2004; **158**(3): 255-61.

127. Lee WN, Stuck D, Konty K, et al. Large-scale influenza vaccination promotion on a mobile app platform: A randomized controlled trial. *Vaccine* 2020; **38**(18): 3508-14.

128. Lerner C, Albertin C, Casillas A, et al. Patient portal reminders for pediatric influenza vaccinations: A randomized clinical trial. *Pediatrics* 2021; **148(2) (no pagination)**.

129. Leung KC, Mui C, Chiu WY, et al. Impact of patient education on influenza vaccine uptake among community-dwelling elderly: a randomized controlled trial. *Health Educ Res* 2017; **32**(5): 455-64.

130. Liao Q, Fielding R, Cheung YTD, Lian J, Yuan J, Lam WWT. Effectiveness and Parental Acceptability of Social Networking Interventions for Promoting Seasonal Influenza Vaccination Among Young Children: randomized Controlled Trial. *Journal of medical Internet research* 2020; **22**(2): e16427.

131. Lieu TA, Elkin EP, Escobar PR, et al. Effect of Electronic and Mail Outreach From Primary Care Physicians for COVID-19 Vaccination of Black and Latino Older Adults: A Randomized Clinical Trial. *JAMA netw* 2022; **5**(6): e2217004.

132. Lin SC, Tam KW, Yen JYC, et al. The impact of shared decision making with patient decision aids on the rotavirus vaccination rate in children: A randomized controlled trial. *Preventive Medicine* 2020; **141 (no pagination)**(106244).

133. Ma GX, Lee MM, Tan Y, et al. Efficacy of a community-based participatory and multilevel intervention to enhance hepatitis B virus screening and vaccination in underserved Korean Americans. *Cancer* 2018; **124**(5): 973-82.

134. Ma GX, Zhu L, Tan Y, et al. A Multilevel Intervention to Increase HPV Vaccination among Asian American Adolescents. *J Community Health* 2022; **47**(1): 9-16.

135. Mantzari E, Vogt F, Marteau TM. Financial incentives for increasing uptake of HPV vaccinations: A randomized controlled trial. *Health Psychology* 2015; **34(2)**: 160-71.

136. Marra F, Kaczorowski J, Gastonguay L, Marra CA, Lynd LD, Kendall P. Pharmacy-based Immunization in Rural Communities Strategy (PhICS): A community cluster-randomized trial. *Can Pharm J (Ott)* 2014; **147**(1): 33-44.

137. Mason BW, Donnelly PD. Targeted mailing of information to improve uptake of measles, mumps, and rubella vaccine: a randomised controlled trial. *Commun Dis Public Health* 2000; **3**(1): 67-8.

138. Masson CL, Delucchi KL, McKnight C, et al. A randomized trial of a hepatitis care coordination model in methadone maintenance treatment. *Am J Public Health* 2013; **103**(10): e81-8.

139. McCaul KD, Johnson RJ, Rothman AJ. The effects of framing and action instructions on whether older adults obtain flu shots. *Health Psychol* 2002; **21**(6): 624-8.

140. Meharry PM. Maternal influenza vaccination strategies to improve vaccine uptake in pregnancy. *Dissertation Abstracts International: Section B: The Sciences and Engineering* 2014; **74**(9-B(E)): No-Specified.

141. Mehta SJ, Mallozzi C, Shaw PA, et al. Effect of Text Messaging and Behavioral Interventions on COVID-19 Vaccination Uptake: A Randomized Clinical Trial. *JAMA netw* 2022; **5**(6): e2216649.

142. Menzies R, Heron L, Lampard J, et al. A randomised controlled trial of SMS messaging and calendar reminders to improve vaccination timeliness in infants. *Vaccine* 2020; **38**(15): 3137‐42.

143. Milkman KL, Gandhi L, Ellis SF, et al. A citywide experiment testing the impact of geographically targeted, high-pay-off vaccine lotteries. *Nat Hum Behav* 2022: 13.

144. Milkman KL, Gandhi L, Patel MS, et al. A 680,000-person megastudy of nudges to encourage vaccination in pharmacies. *Proc Natl Acad Sci U S A* 2022; **119**(6): 08.

145. Moniz MH, Hasley S, Meyn LA, Beigi RH. Improving influenza vaccination rates in pregnancy through text messaging: a randomized controlled trial. *Obstet Gynecol* 2013; **121**(4): 734-40.

146. Muñoz-Miralles R, Bonvehí Nadeu S, Sant Masoliver C, et al. Effectiveness of a brief intervention for acceptance of influenza vaccine in reluctant primary care patients. *Gaceta sanitaria* 2022; **36**(5): 446‐51.

147. Nct. Persuasion in Medicine: experimental Evidence on Sender and Signal Effects. *https://clinicaltrialsgov/show/NCT04160975* 2019.

148. Nct. Lottery Incentive Nudges to Increase Influenza Vaccinations. *https://clinicaltrialsgov/show/NCT05012163* 2021.

149. Nct. SMS Reminders to Strengthen Demand for HPV Vaccination in Georgia. *https://clinicaltrialsgov/show/NCT05536674* 2022.

150. Nct. Vale+ Tu Salud: corner-Based Randomized Trial to Test a Latino Day Laborer Program Adapted to Prevent COVID 19. *https://clinicaltrialsgov/show/NCT05248399* 2022.

151. Nct. Motivation, Syringe Exchange, and COVID-19. *https://clinicaltrialsgov/ct2/show/NCT05534061* 2022.

152. Nct. Precision Vaccine Promotion in Underserved Populations. *https://clinicaltrialsgov/ct2/show/NCT05537441* 2022.

153. Nehme EK, Delphia M, Cha EM, Thomas M, Lakey D. Promoting Influenza Vaccination Among an ACA Health Plan Subscriber Population: A Randomized Trial. *Am J Health Promot* 2019; **33**(6): 916-20.

154. Nowalk MP, Lin CJ, Toback SL, et al. Improving influenza vaccination rates in the workplace: a randomized trial. *Am J Prev Med* 2010; **38**(3): 237-46.

155. Nyamathi A, Liu Y, Marfisee M, et al. Effects of a nurse-managed program on hepatitis A and B vaccine completion among homeless adults. *Nurs Res* 2009; **58**(1): 13-22.

156. Nyamathi A, Sinha K, Greengold B, Cohen A, Marfisee M. Predictors of HAV/HBV vaccination completion among methadone maintenance clients. *Res Nurs Health* 2010; **33**(2): 120-32.

157. O'Grady KAF, Kaus M, Jones L, et al. SMS reminders to improve the uptake and timeliness of the primary immunisation series in infants: a multi-centre randomised controlled trial. *Communicable diseases intelligence* 2018; **46**(no pagination).

158. O'Grady KF, Kaus M, Jones L, et al. SMS reminders to improve the uptake and timeliness of the primary immunisation series in infants: a multi-centre randomised controlled trial. *Commun Dis Intell (2018)* 2022; **46**: 19.

159. O'Leary ST, Lee M, Lockhart S, et al. Effectiveness and Cost of Bidirectional Text Messaging for Adolescent Vaccines and Well Care. *Pediatrics* 2015; **136**(5): e1220-7.

160. Omer SB, O'Leary ST, Bednarczyk RA, et al. Multi-tiered intervention to increase maternal immunization coverage: A randomized, controlled trial. *Vaccine* 2022; **40**(34): 4955-63.

161. Osborne MT, Kenah E, Lancaster K, Tien J. Catch the tweet to fight the flu: Using Twitter to promote flu shots on a college campus. *J Am Coll Health* 2023; **71**(8): 2470-84.

162. Otsuka SH, Tayal NH, Porter K, Embi PJ, Beatty SJ. Improving herpes zoster vaccination rates through use of a clinical pharmacist and a personal health record. *Am J Med* 2013; **126**(9): 832.e1-6.

163. Otsuka-Ono H, Hori N, Ohta H, Uemura Y, Kamibeppu K. A childhood immunization education program for parents delivered during late pregnancy and one-month postpartum: a randomized controlled trial. *BMC Health Serv Res* 2019; **19**(1): 798.

164. Patel A, Stern L, Unger Z, et al. Staying on track: a cluster randomized controlled trial of automated reminders aimed at increasing human papillomavirus vaccine completion. *Vaccine* 2014; **32**(21): 2428‐33.

165. Patel DA, Zochowski M, Peterman S, Dempsey AF, Ernst S, Dalton VK. Human papillomavirus vaccine intent and uptake among female college students. *J Am Coll Health* 2012; **60**(2): 151-61.

166. Patel MS, Milkman KL, Gandhi L, et al. A Randomized Trial of Behavioral Nudges Delivered Through Text Messages to Increase Influenza Vaccination Among Patients With an Upcoming Primary Care Visit. *Am J Health Promot* 2023; **37**(3): 324-32.

167. Payakachat N, Hadden KB, Ragland D. Promoting Tdap immunization in pregnancy: Associations between maternal perceptions and vaccination rates. *Vaccine* 2016; **34**(1): 179-86.

168. Porter-Jones G, Williams S, Powell C, Pusey L, Roberts RJ. Impact of a novel way to communicate information about MMR on uptake of MMR vaccine: A randomized controlled trial. *Public Health* 2009; **123**(1): 78-80.

169. Pot M, Paulussen T, Ruiter RAC, et al. Effectiveness of a Web-Based Tailored Intervention With Virtual Assistants Promoting the Acceptability of HPV Vaccination Among Mothers of Invited Girls: Randomized Controlled Trial. *J Med Internet Res* 2017; **19**(9): 18.

170. Qin C, Li Y, Qiu S, et al. Pay-it-forward to increase uptake among 15-18-year-old adolescent girls compared with user-paid vaccination: The pilot results of a two-arm randomized controlled trial in China. *Res Sq* 2023; **25**: 25.

171. Quinlivan JA, Box H, Evans SF. Postnatal home visits in teenage mothers: a randomised controlled trial. *Lancet* 2003; **361**(9361): 893-900.

172. Rabb N, Swindal M, Glick D, et al. Evidence from a statewide vaccination RCT shows the limits of nudges. *Nature* 2022; **604**(7904): E1-E7.

173. Rand CM, Brill H, Albertin C, et al. Effectiveness of centralized text message reminders on human papillomavirus immunization coverage for publicly insured adolescents. *Journal of Adolescent Health* 2015; **56**(5, Suppl): S17-S20.

174. Rand CM, Vincelli P, Goldstein NPN, Blumkin A, Szilagyi PG. Effects of phone and text message reminders on completion of the human papillomavirus vaccine series. *Journal of Adolescent Health* 2017; **60**(1): 113-9.

175. Reiter PL, Gower AL, Kiss DE, et al. Efficacy of the Outsmart HPV Intervention: A Randomized Controlled Trial to Increase HPV Vaccination among Young Gay, Bisexual, and Other Men Who Have Sex with Men. *Cancer Epidemiol Biomarkers Prev* 2023; **32**(6): 760-7.

176. Reiter PL, Katz ML, Bauermeister JA, Shoben AB, Paskett ED, McRee A-L. Increasing Human Papillomavirus Vaccination Among Young Gay and Bisexual Men: A Randomized Pilot Trial of the Outsmart HPV Intervention. *LGBT Health* 2018; **5**(5): 325-9.

177. Richman AR, Torres E, Goldberg EJ. A randomized intervention study to evaluate whether electronic messaging can increase HPV vaccine uptake and knowledge. *Journal of lower genital tract disease* 2016; **20**(2): S28‐.

178. Richman AR, Torres E, Wu Q, et al. Text and Email Messaging for Increasing Human Papillomavirus Vaccine Completion among Uninsured or Medicaid-insured Adolescents in Rural Eastern North Carolina. *Journal of Health Care for the Poor & Underserved* 2019; **30**(4): 1499-517.

179. Rickert VI, Auslander BA, Cox DS, Rosenthal SL, Rupp RE, Zimet GD. School-based HPV immunization of young adolescents: effects of two brief health interventions. *Hum Vaccin Immunother* 2015; **11**(2): 315-21.

180. Roca B, Herrero E, Resino E, Torres V, Penades M, Andreu C. Impact of education program on influenza vaccination rates in Spain. *Am J Manag Care* 2012; **18**(12): e446-52.

181. Rodriguez RM, Eucker SA, Rafique Z, et al. Promotion of Influenza Vaccination in the Emergency Department. *NEJM Evidence* 2024; **3**(4): 1-9.

182. Rodriguez RM, Nichol G, Eucker SA, et al. Effect of COVID-19 Vaccine Messaging Platforms in Emergency Departments on Vaccine Acceptance and Uptake: A Cluster Randomized Clinical Trial. *JAMA Intern Med* 2022; **27**: 27.

183. Ronzani P, Panizza F, Martini C, Savadori L, Motterlini M. Countering vaccine hesitancy through medical expert endorsement. *Vaccine* 2022; **40(32)**: 4635-43.

184. Saaksvuori L, Betsch C, Nohynek H, Salo H, Sivela J, Bohm R. Information nudges for influenza vaccination: Evidence from a large-scale cluster-randomized controlled trial in Finland. *PLoS medicine* 2022; **19(2)**: e1003919.

185. Saccardo S, Dai H, Han MA, Vangala S, Hoo J, Fujimoto J. Field testing the transferability of behavioural science knowledge on promoting vaccinations. *Nat* 2024; **14**: 14.

186. Saitoh A, Sato I, Shinozaki T, Kamiya H, Nagata S. Effect of stepwise perinatal immunization education: A cluster-randomized controlled trial. *Vaccine* 2017; **35**(12): 1645-51.

187. Santa Maria D, Markham C, Misra SM, et al. Effects of a randomized controlled trial of a brief, student-nurse led, parent-based sexual health intervention on parental protective factors and HPV vaccination uptake. *BMC Public Health* 2021; **21**(1): 585.

188. Scarinci IC, Hansen B, Kim YI. HPV vaccine uptake among daughters of Latinx immigrant mothers: Findings from a cluster randomized controlled trial of a community-based, culturally relevant intervention. *Vaccine* 2020; **38**(25): 4125-34.

189. Schwarz K, Garrett B, Lee J, et al. Positive impact of a shelter-based hepatitis B vaccine program in homeless Baltimore children and adolescents. *J Urban Health* 2008; **85**(2): 228-38.

190. Scott VP, Opel DJ, Reifler J, et al. Office-Based Educational Handout for Influenza Vaccination: A Randomized Controlled Trial. *Pediatrics* 2019; **144**(2): 08.

191. Shegog R, Savas LS, Healy CM, et al. AVPCancerFree: impact of a digital behavior change intervention on parental HPV vaccine –related perceptions and behaviors. *Human vaccines and immunotherapeutics* 2022.

192. Shen Y, Wang J, Nicholas S, et al. Effectiveness of financial incentives on influenza vaccination among older adults in China: a randomized clinical trial. *Clin Microbiol Infect* 2024; **08**: 08.

193. Shourie S, Jackson C, Cheater FM, et al. A cluster randomised controlled trial of a web based decision aid to support parents' decisions about their child's Measles Mumps and Rubella (MMR) vaccination. *Vaccine* 2013; **31**(50): 6003-10.

194. Si M, Su X, Jiang Y, et al. Effect of an IMB Model-Based Education on the Acceptability of HPV Vaccination Among College Girls in Mainland China: A Cluster RCT. *Cancer Control* 2022; **29**: 10732748211070719.

195. Sitler LL. The effectiveness of combined appointments and influenza immunization rates in a rural WIC population. *Dissertation Abstracts International: Section B: The Sciences and Engineering* 2018; **78**(11-B(E)): No-Specified.

196. Skinner SR, Imberger A, Nolan T, Lester R, Glover S, Bowes G. Randomised controlled trial of an educational strategy to increase school-based adolescent hepatitis B vaccination. *Aust N Z J Public Health* 2000; **24**(3): 298-304.

197. Song Y, Oh J, Han S, Choi C. Effectiveness of Telephone and Postcard Reminders for the Influenza Vaccination: a Study in the Elderly Who Have Visited a Family Practice Center in a Tertiary Care Hospital. *Korean journal of preventive medicine* 2000; **33**(1): 109‐16.

198. Stockwell MS, Hofstetter AM, DuRivage N, et al. Text message reminders for second dose of influenza vaccine: a randomized controlled trial. *Pediatrics* 2015; **135**(1): e83-91.

199. Stockwell MS, Kharbanda EO, Martinez RA, et al. Text4Health: Impact of Text Message Reminder-Recalls for Pediatric and Adolescent Immunizations. *Am J Public Health* 2012; **102**(2): E15-E21.

200. Stockwell MS, Kharbanda EO, Martinez RA, Vargas CY, Vawdrey DK, Camargo S. Effect of a text messaging intervention on influenza vaccination in an urban, low-income pediatric and adolescent population: a randomized controlled trial. *Jama* 2012; **307**(16): 1702-8.

201. Stockwell MS, Shone LP, Nekrasova E, et al. Text Message Reminders for the Second Dose of Influenza Vaccine for Children: an RCT. *Pediatrics* 2022.

202. Stockwell MS, Westhoff C, Kharbanda EO, et al. Influenza vaccine text message reminders for urban, low-income pregnant women: a randomized controlled trial. *Am J Public Health* 2014; **104 Suppl 1**: e7-12.

203. Stolpe S, Choudhry NK. Effect of Automated Immunization Registry-Based Telephonic Interventions on Adult Vaccination Rates in Community Pharmacies: A Randomized Controlled Trial. *J Manag Care Spec Pharm* 2019; **25**(9): 989-94.

204. Strathdee SA, Abramovitz D, Harvey-Vera AY, et al. A Brief Peer-Led Intervention to Increase COVID-19 Vaccine Uptake Among People Who Inject Drugs in San Diego County: Results From a Pilot Randomized Controlled Trial. *Open forum infect* 2023; **10**(8): ofad392.

205. Stuck AE, Moser A, Morf U, et al. Effect of health risk assessment and counselling on health behaviour and survival in older people: a pragmatic randomised trial. *PLoS Med* 2015; **12**(10): e1001889.

206. Suh CA, Saville A, Daley MF, et al. Effectiveness and net cost of reminder/recall for adolescent immunizations. *Pediatrics* 2012; **129**(6): e1437-45.

207. Suzuki Y, Sukegawa A, Ueda Y, et al. The Effect of a Web-Based Cervical Cancer Survivor's Story on Parents' Behavior and Willingness to Consider Human Papillomavirus Vaccination for Daughters: Randomized Controlled Trial. *JMIR Public Health Surveill* 2022; **8**(5): 15.

208. Sweeney JB. An evaluation of an intervention for hpv risk reduction among college-aged women. *Dissertation Abstracts International: Section B: The Sciences and Engineering* 2014; **75**(1-B(E)): No-Specified.

209. Szilagyi P, Albertin C, Gurfinkel D, et al. Effect of State Immunization Information System Centralized Reminder and Recall on HPV Vaccination Rates. *Pediatrics* 2020; **145**(5): 05.

210. Szilagyi PG, Albertin C, Casillas A, et al. Effect of Patient Portal Reminders Sent by a Health Care System on Influenza Vaccination Rates: A Randomized Clinical Trial. *JAMA Intern Med* 2020; **180**(7): 962-70.

211. Szilagyi PG, Albertin C, Humiston SG, et al. A randomized trial of the effect of centralized reminder/recall on immunizations and preventive care visits for adolescents. *Acad Pediatr* 2013; **13**(3): 204-13.

212. Szilagyi PG, Albertin CS, Casillas A, et al. Effect of Personalized Messages Sent by a Health System's Patient Portal on Influenza Vaccination Rates: a Randomized Clinical Trial. *Journal of general internal medicine* 2022; **37**(3): 615‐23.

213. Szilagyi PG, Albertin CS, Saville AW, et al. Effect of State Immunization Information System Based Reminder/Recall for Influenza Vaccinations: a Randomized Trial of Autodialer, Text, and Mailed Messages. *Journal of pediatrics* 2020; **221**: 123‐31.e4.

214. Szilagyi PG, Casillas A, Duru OK, et al. Evaluation of behavioral economic strategies to raise influenza vaccination rates across a health system: Results from a randomized clinical trial. *Prev Med* 2023; **170**: 107474.

215. Szilagyi PG, Duru OK, Casillas A, et al. Text vs Patient Portal Messaging to Improve Influenza Vaccination Coverage: A Health System-Wide Randomized Clinical Trial. *JAMA Intern Med* 2024; **18**: 18.

216. Szilagyi PG, Humiston SG, Gallivan S, Albertin C, Sandler M, Blumkin A. Effectiveness of a citywide patient immunization navigator program on improving adolescent immunizations and preventive care visit rates. *Arch Pediatr Adolesc Med* 2011; **165**(6): 547-53.

217. Szilagyi PG, Schaffer S, Barth R, et al. Effect of telephone reminder/recall on adolescent immunization and preventive visits: results from a randomized clinical trial. *Arch Pediatr Adolesc Med* 2006; **160**(2): 157-63.

218. Szilagyi PG, Schaffer S, Rand CM, et al. Impact of elementary school-located influenza vaccinations: A stepped wedge trial across a community. *Vaccine* 2018; **36**(20): 2861-9.

219. Szilagyi PG, Schaffer S, Rand CM, et al. Text Message Reminders for Child Influenza Vaccination in the Setting of School-Located Influenza Vaccination: A Randomized Clinical Trial. *Clin Pediatr (Phila)* 2019; **58**(4): 428-36.

220. Tentori K, Pighin S, Giovanazzi G, Grignolio A, Timberlake B, Ferro A. Nudging COVID-19 Vaccine Uptake by Changing the Default: A Randomized Controlled Trial. *Medical Decision Making* 2022; **42**(6): 837-41.

221. Terrell-Perica SM, Effler PV, Houck PM, Lee L, Crosthwaite GH. The effect of a combined influenza/pneumococcal immunization reminder letter. *Am J Prev Med* 2001; **21**(4): 256-60.

222. Thilly N, Michel M, Simon M, et al. Effectiveness of a School- and Primary Care-Based HPV Vaccination Intervention: The PrevHPV Cluster Randomized Trial. *JAMA Netw Open* 2024; **7**(5): e2411938.

223. Tiro JA, Sanders JM, Pruitt SL, et al. Promoting HPV Vaccination in Safety-Net Clinics: A Randomized Trial. *Pediatrics* 2015; **136**(5): 850-9.

224. Topp L, Day CA, Wand H, et al. A randomised controlled trial of financial incentives to increase hepatitis B vaccination completion among people who inject drugs in Australia. *Prev Med* 2013; **57**(4): 297-303.

225. Tubiana S, Labarere J, Levraut J, et al. Effectiveness of a multifaceted informational-based and text message reminders on pneumococcal and influenza vaccinations in hospital emergency departments: A cluster-randomized controlled trial. *Vaccines* 2021; **9(9) (no pagination)**(962).

226. Tull F, Borg K, Knott C, et al. Short Message Service Reminders to Parents for Increasing Adolescent Human Papillomavirus Vaccination Rates in a Secondary School Vaccine Program: A Randomized Control Trial. *J Adolesc Health* 2019; **65**(1): 116-23.

227. Ueberroth BE, Labonte HR, Wallace MR. Impact of Patient Portal Messaging Reminders with Self-Scheduling Option on Influenza Vaccination Rates: a Prospective, Randomized Trial. *Journal of general internal medicine* 2022; **37**(6): 1394‐9.

228. Underwood NL, Weiss P, Gargano LM, et al. Human papillomavirus vaccination among adolescents in Georgia. *Human vaccines & immunotherapeutics* 2015; **11**(7): 1703‐8.

229. Usami T, Hashiguchi M, Kouhara T, Ishii A, Nagata T, Mochizuki M. Impact of community pharmacists advocating immunization on influenza vaccination rates among the elderly. *Yakugaku Zasshi* 2009; **129**(9): 1063-8.

230. Vanderpool RC, Cohen E, Crosby RA, et al. "1-2-3 Pap" Intervention Improves HPV Vaccine Series Completion among Appalachian Women. *J Commun* 2013; **63**(1): 95-115.

231. Vivier PM, Alario AJ, O'Haire C, Dansereau LM, Jakum EB, Peter G. The impact of outreach efforts in reaching underimmunized children in a Medicaid managed care practice. *Arch Pediatr Adolesc Med* 2000; **154**(12): 1243-7.

232. Wagner NM, Dempsey AF, Narwaney KJ, et al. Addressing logistical barriers to childhood vaccination using an automated reminder system and online resource intervention: A randomized controlled trial. *Vaccine* 2021; **39**(29): 3983-90.

233. Wang Z, Chan PS, Fang Y, et al. Chatbot-Delivered Online Intervention to Promote Seasonal Influenza Vaccination During the COVID-19 Pandemic: A Randomized Clinical Trial. *JAMA netw* 2023; **6**(9): e2332568.

234. Wang ZX, Lau JTF, Ip TKM, et al. Two Web-Based and Theory-Based Interventions With and Without Brief Motivational Interviewing in the Promotion of Human Papillomavirus Vaccination Among Chinese Men Who Have Sex With Men: Randomized Controlled Trial. *J Med Internet Res* 2021; **23**(2): 17.

235. Weaver T, Metrebian N, Hellier J, et al. Use of contingency management incentives to improve completion of hepatitis B vaccination in people undergoing treatment for heroin dependence: a cluster randomised trial. *Lancet* 2014; **384**(9938): 153-63.

236. Wijesundara JG, Ito Fukunaga M, Ogarek J, et al. Electronic Health Record Portal Messages and Interactive Voice Response Calls to Improve Rates of Early Season Influenza Vaccination: Randomized Controlled Trial. *Journal of medical Internet research* 2020; **22(9)**: e16373.

237. Wilcox SA, Koepke CP, Levenson R, Thalheimer JC. Registry-driven, community-based immunization outreach: a randomized controlled trial. *Am J Public Health* 2001; **91**(9): 1507-11.

238. Winston CA, Mims AD, Leatherwood KA. Increasing pneumococcal vaccination in managed care through telephone outreach. *Am J Manag Care* 2007; **13**(10): 581-8.

239. Wiseman P. A Study to Determine the Preliminary Effects of a Theory-Based Intervention (SayNo2Flu) Combined with the Use of Mobile Technology on Parents' Influenza Prevention Beliefs and Behaviors in a Primary Care Setting. *Dissertation Abstracts International: Section B: The Sciences and Engineering* 2016; **76**(9-B(E)): No-Specified.

240. Wong VWY, Fong DYT, Lok KYW, et al. Brief education to promote maternal influenza vaccine uptake: A randomized controlled trial. *Vaccine* 2016; **34**(44): 5243-50.

241. Wootton SH, Blackwell SC, Saade G, et al. Randomized Quality Improvement Trial of Opting-In Versus Opting-Out to Increase Influenza Vaccination Rates during Pregnancy. *Am* 2018; **8**(3): e161-e7.

242. Wouters K, Leuridan E, Van Herck K, et al. Compliance and immunogenicity of two hepatitis B vaccination schedules in sex workers in Belgium. *Vaccine* 2007; **25**(10): 1893-900.

243. Wright A, Poon EG, Wald J, et al. Randomized controlled trial of health maintenance reminders provided directly to patients through an electronic PHR. *Journal of General Internal Medicine* 2012; **27**(1): 85-92.

244. Wynn CS, Catallozzi M, Kolff CA, et al. Personalized Reminders for Immunization Using Short Messaging Systems to Improve Human Papillomavirus Vaccination Series Completion: Parallel-Group Randomized Trial. *JMIR Mhealth Uhealth* 2021; **9**(12): e26356.

245. Xu J, Tang W, Qiu W, et al. Effects of mobile APP for immunization on vaccination compliance of migrant children in southwest China: A community trial study. *Hum Vaccin Immunother* 2022; **18**(7): 2135853.

246. Yeung KHT, Tarrant M, Chan KCC, Tam WH, Nelson EAS. Increasing influenza vaccine uptake in children: a randomised controlled trial. *Vaccine* 2018; **36**(37): 5524‐35.

247. Yokum D, Lauffenburger JC, Ghazinouri R, Choudhry NK. Letters designed with behavioural science increase influenza vaccination in Medicare beneficiaries. *Nat* 2018; **2**(10): 743-9.

248. Yudin MH, Mistry N, De Souza LR, et al. Text messages for influenza vaccination among pregnant women: A randomized controlled trial. *Vaccine* 2017; **35**(5): 842-8.

249. Yue M, Wang Y, Low CK, Yoong JSY, Cook AR. Optimal Design of Population-Level Financial Incentives of Influenza Vaccination for the Elderly. *Value Health* 2020; **23**(2): 200-8.

250. Zhang CQ, Zhang R, Chung PK, et al. Promoting influenza prevention for elderly people in Hong Kong using health action process approach: study protocol. *BMC Public Health* 2018; **18**: 9.

251. Zhang SX, Shoptaw S, Reback CJ, Yadav K, Nyamathi AM. Cost-effective way to reduce stimulant-abuse among gay/bisexual men and transgender women: a randomized clinical trial with a cost comparison. *Public Health* 2018; **154**: 151-60.

252. Zhang X, Chen H, Zhou J, Huang Q, Feng XY, Li J. Impact of web-based health education on HPV vaccination uptake among college girl students in Western and Northern China: a follow-up study. *BMC Womens Health* 2022; **22**(1): 46.

253. Zuniga de Nuncio ML, Nader PR, Sawyer MH, De Guire M, Prislin R, Elder JP. A prenatal intervention study to improve timeliness of immunization initiation in Latino infants. *J Community Health* 2003; **28**(2): 151-65.

## **U: List of excluded studies at full text with brief reasons**

Reasons for exclusion at full text screening. Records may have been ineligible for multiple criteria, however we recorded only one reason for each exclusion according to the following hierarchy: wrong study design, wrong population, wrong intervention, wrong outcome, wrong article type [e.g. editorial, commentary, review], <100 participants, not 3+ clusters per arm; published prior to 2000, can't find full text, not a high or upper middle income country, and ongoing trial.

| **Study name** | **Reason for exclusion** |
| --- | --- |
| AbascalMiguel 2024 | Wrong study design |
| Abdel-Qader 2022 | Wrong outcome |
| AbdulRahman 2013 | Wrong study design |
| Abramson 2010 | Wrong population |
| Abramson 2011 | Wrong intervention |
| Abramson 2015 | Wrong intervention |
| Actrn 2010 | Wrong intervention |
| Actrn 2013 | Wrong study design |
| Actrn 2018 | Can't find full text |
| Adams 2012 | Wrong article type [e.g. editorial, commentary, review] |
| Agenor 2020 | Wrong study design |
| Ahmed 2004 | Wrong population |
| Alessandrini 2019 | Wrong study design |
| Alhajji 2023 | Wrong outcome |
| Alzouby 2019 | Can't find full text |
| Andraweera 2023 | Ongoing |
| Anonymous 2004 | Wrong article type [e.g. editorial, commentary, review] |
| Anton 2016 | Wrong study design |
| Anton 2016 | Wrong study design |
| Arslan 2012 | Wrong study design |
| Attwell 2020 | Wrong study design |
| Bailey 2022 | Wrong study design |
| Baker 2014 | Wrong study design |
| Baldwin 2021 | Can't find full text |
| Barbara 2020 | Wrong study design |
| Barbaroux 2021 | Wrong population |
| Bar-Shain 2015 | Wrong study design |
| Bauer 2021 | Wrong study design |
| Beck 2013 | Published prior to 2000 |
| Beleites 2024 | Wrong outcome |
| Belmaker 2006 | Wrong study design |
| Berenbrok 2022 | Wrong study design |
| Berenbrok 2023 | Wrong study design |
| Berenson 2020 | Wrong study design |
| Bernstein 2022 | Wrong study design |
| Berry 2022 | Wrong population |
| Bethke 2022 | Wrong study design |
| Betsch 2018 | Wrong study design |
| Bhatt 2024 | Wrong population |
| Bhatt 2024 | Wrong population |
| Bielecki 2020 | Wrong study design |
| Birchmeier 2002 | Wrong study design |
| Bocquier 2022 | Wrong article type [e.g. editorial, commentary, review] |
| Bocquier 2023 | Wrong outcome |
| Borgey 2019 | Wrong population |
| Bossio 2019 | Wrong study design |
| Bou-Mias 2006 | Wrong study design |
| Bressin 2019 | Wrong study design |
| Brewer 2018 | Wrong intervention |
| Brewer 2020 | Wrong study design |
| Britto 2007 | Wrong study design |
| Brunelli 2022 | Ongoing |
| Bucchiotty 2021 | Wrong study design |
| Budhwani 2023 | Ongoing |
| Buller 2021 | Wrong intervention |
| Buller 2021 | Wrong study design |
| Buller 2024 | Ongoing |
| Burns 2002 | Wrong intervention |
| Bushar 2017 | Wrong study design |
| Buttenheim 2016 | <100 participants |
| Cadena 2011 | Wrong population |
| Cameron 2011 | Wrong outcome |
| Campbell 2007 | Wrong study design |
| Canakis 2019 | Wrong population |
| Carney 2019 | Wrong intervention |
| Carty 2018 | Wrong intervention |
| Casalino 2018 | Wrong study design |
| Casey 2022 | Wrong study design |
| Caskey 2011 | Can't find full text |
| Caskey 2017 | Wrong study design |
| Castellanos 2018 | Can't find full text |
| Cataldi 2020 | Wrong intervention |
| Cates 2018 | Wrong study design |
| Cecinati 2010 | Wrong population |
| Chamberlain 2015 | Wrong intervention |
| Chamberlain 2016 | Wrong outcome |
| Chambers 2015 | Wrong intervention |
| Chamoux 2006 | Wrong population |
| Chan 2015 | Wrong population |
| Chantler 2020 | Wrong study design |
| Chau 2020 | Can't find full text |
| Chen 2010 | Wrong population |
| Chen 2014 | Wrong intervention |
| Chen 2016 | Wrong intervention |
| Cheng 2015 | Wrong study design |
| Cheng 2023 | Wrong population |
| Chetty-Makkan 2022 | Wrong study design |
| Chetty-Makkan 2022 | Wrong study design |
| Clark 2015 | Wrong study design |
| Coenen 2016 | Wrong population |
| Coenen 2016 | Wrong population |
| Coenen 2017 | Wrong population |
| Coffman 2024 | Wrong outcome |
| Coley 2020 | Wrong study design |
| Conner 2011 | Wrong population |
| Cook 2014 | Wrong study design |
| Cory 2019 | Wrong outcome |
| Costantino 2019 | Wrong study design |
| Costantino 2020 | Wrong study design |
| Costantino 2020 | Wrong study design |
| Crawford 2011 | Can't find full text |
| Currat 2020 | Wrong population |
| Currie 2023 | Ongoing |
| Daley 2004 | Wrong study design |
| Daley 2004 | Wrong population |
| Daley 2018 | Wrong outcome |
| Daly 2016 | Wrong study design |
| Darville 2018 | Wrong outcome |
| Davies 2005 | Can't find full text |
| Delamater 2019 | Wrong study design |
| Dempsey 2018 | Wrong intervention |
| Dempsey 2019 | Wrong study design |
| DeSarro 2021 | Wrong study design |
| Deshmukh 2017 | Wrong study design |
| Deshmukh 2018 | Wrong study design |
| Dey 2001 | Wrong population |
| Dick 2014 | Wrong study design |
| Dike 2022 | Wrong outcome |
| Dixon 2019 | Not 3+ clusters per arm |
| Dixson 2021 | Can't find full text |
| Dombkowski 2012 | Wrong population |
| Dombkowski 2014 | Wrong study design |
| DomenechBonilla 2011 | Wrong study design |
| Donahue 2018 | Wrong outcome |
| Doratotaj 2008 | Wrong population |
| Drees 2015 | Wrong study design |
| Dreyer 2022 | Wrong study design |
| Drks 2019 | Wrong population |
| Drozd 2017 | Wrong study design |
| EggersRussell 2018 | Wrong study design |
| Eitze 2021 | Wrong outcome |
| Ekmez 2022 | Wrong outcome |
| El-Halabi 2023 | Wrong study design |
| Epstein 2020 | Wrong article type [e.g. editorial, commentary, review] |
| Esposito 2009 | Wrong population |
| Esteban-Vasallo 2019 | Wrong study design |
| Eubelen 2011 | Not 3+ clusters per arm |
| Fadnes 2011 | Wrong study design |
| Falcone 2020 | Wrong study design |
| Fan 2021 | Wrong study design |
| Fernandez 2017 | Can't find full text |
| FinneyRutten 2018 | Wrong intervention |
| Fishbein 2006 | Wrong study design |
| Focht 2014 | Can't find full text |
| Folz 2015 | Wrong intervention |
| Forster 2017 | Wrong outcome |
| Forster 2018 | Wrong outcome |
| Forster 2018 | Wrong outcome |
| Frank 2004 | Wrong intervention |
| Frank 2021 | Ongoing |
| Fratantoni 2022 | Wrong intervention |
| Friedl 2012 | Wrong study design |
| Gagneur 2018 | Wrong study design |
| Gagneur 2019 | Wrong study design |
| Ganczak 2018 | Wrong study design |
| Gargano 2010 | Wrong study design |
| Gargano 2011 | Wrong study design |
| Gargano 2011 | Wrong study design |
| Gargano 2013 | Wrong article type |
| Gatwood 2023 | Wrong intervention |
| Gentile 2020 | Wrong study design |
| Ghaffarzadegan 2022 | Wrong study design |
| Giles 2021 | Wrong study design |
| Gilkey 2014 | Wrong intervention |
| Gill 2000 | Wrong study design |
| Ginson 2000 | Wrong population |
| Glenn 2022 | Wrong intervention |
| Glik 2004 | Wrong study design |
| Golebiak 2020 | Wrong population |
| Gottlieb 2001 | Wrong study design |
| Grace 2018 | Wrong intervention |
| Grivas 2015 | Wrong study design |
| Grivas 2017 | Wrong study design |
| Guerra 2023 | Wrong population |
| Gurdin 2008 | Wrong intervention |
| Guthmann 2010 | Wrong study design |
| Gutierrez 2017 | Wrong study design |
| Haff 2023 | Wrong article type [e.g. editorial, commentary, review] |
| Hallas 2019 | Wrong study design |
| Hambidge 2000 | Wrong intervention |
| Hambidge 2004 | Wrong intervention |
| Hamper 2012 | Can't find full text |
| Hansen 2018 | Can't find full text |
| Harry 2022 | Wrong intervention |
| Hayles 2015 | Wrong study design |
| Hayward 2006 | Wrong population |
| Hechter 2019 | Wrong study design |
| HeeYun 2016 | Wrong study design |
| Hendrix 2015 | Wrong article type [e.g. editorial, commentary, review] |
| Herbert 2014 | Can't find full text |
| Hernandez-Ray 2016 | Wrong study design |
| Herrett 2014 | Wrong population |
| Herrett 2016 | Wrong population |
| Hicks 2007 | Wrong study design |
| Hirani 2022 | Wrong study design |
| Hofstetter 2017 | Wrong population |
| Hohwu 2012 | Wrong study design |
| Homaira 2020 | <100 participants |
| Honda 2013 | Wrong study design |
| Honda 2013 | Wrong study design |
| Howe 2022 | Wrong study design |
| Hu 2015 | Wrong study design |
| Huang 2018 | Wrong study design |
| Huf 2022 | Wrong study design |
| Hull 2010 | Wrong study design |
| Humiston 2011 | Wrong intervention |
| Hunt 2021 | Wrong study design |
| Huth 2014 | Wrong study design |
| Ibanez-Jimenez 2007 | Can't find full text |
| Ingemi 2014 | Wrong study design |
| Irigoyen 2000 | Wrong study design |
| IrohTam 2009 | Wrong study design |
| Isais 2018 | Wrong study design |
| Isenor 2020 | Wrong study design |
| Isler 2020 | Wrong population |
| Isrctn 2015 | Wrong intervention |
| Isrctn 2016 | Wrong population |
| Isrctn 2021 | Ongoing |
| Jama 2017 | Wrong study design |
| Jama 2018 | Wrong study design |
| Jane 2011 | Wrong outcome |
| Jiang 2021 | Wrong study design |
| Jiang 2021 | Wrong population |
| Johansen 2023 | Wrong population |
| Jprn 2019 | Wrong intervention |
| Kahn 2018 | Wrong study design |
| Kaljee 2015 | Wrong article type [e.g. editorial, commentary, review] |
| Kamath 2017 | Wrong outcome |
| Kamath 2018 | Wrong intervention |
| Kassi 2021 | Wrong outcome |
| Kassi 2022 | Wrong study design |
| Kaul 2019 | Wrong study design |
| Kaushik 2020 | Wrong study design |
| Keeshin 2017 | Wrong study design |
| Kellerman 2000 | Wrong study design |
| Kempe 2013 | Wrong intervention |
| Kempe 2014 | Wrong intervention |
| Kempe 2015 | Wrong intervention |
| Kempe 2017 | Wrong intervention |
| Kemsley 2004 | Wrong study design |
| Kenyon 2012 | Wrong intervention |
| Kenyon 2016 | Wrong intervention |
| Kepka 2021 | Wrong study design |
| Khader 2019 | Wrong study design |
| Kharbanda 2011 | Wrong study design |
| Kharbanda 2011 | Wrong study design |
| Kiderlen 2022 | Wrong study design |
| Kim 2015 | Wrong population |
| Kim 2017 | Wrong study design |
| Kimura 2007 | Wrong population |
| Klassing 2018 | Wrong population |
| Knox 2023 | Wrong population |
| Kobayashi 2023 | Wrong outcome |
| Krawczyk 2012 | Wrong outcome |
| Krelle 2023 | Can't find full text |
| Krelle 2023 | Can't find full text |
| Kreuter 2004 | Wrong study design |
| Kreuter 2016 | Wrong outcome |
| Kumyod 2024 | Wrong study design |
| Kuppuswamy 2016 | Wrong population |
| Lancaster 2018 | Wrong study design |
| Lassen 2023 | Wrong population |
| Lassen 2023 | Wrong population |
| Latessa 2000 | Wrong study design |
| Lau 2020 | Wrong study design |
| Launay 2014 | Wrong intervention |
| Lawton 2021 | Ongoing |
| Leboucher 2012 | Wrong study design |
| Lee 2003 | Wrong intervention |
| Lee 2008 | Wrong study design |
| Lee 2018 | <100 participants |
| Lee 2021 | Wrong study design |
| Lee 2021 | Wrong population |
| Lee 2021 | Wrong population |
| Lee 2022 | Wrong study design |
| Lee 2023 | Wrong outcome |
| Lee 2023 | Wrong outcome |
| Lee 2023 | Wrong outcome |
| Lefevere 2016 | Wrong study design |
| Lehmann 2016 | Wrong population |
| Lemaitre 2019 | Wrong study design |
| Lemstra 2011 | Wrong study design |
| Lennox 2010 | Wrong intervention |
| Levine 2021 | Wrong outcome |
| Lewin 2022 | <100 participants |
| Li 2022 | Wrong study design |
| Liao 2021 | Wrong outcome |
| Link 2014 | Wrong article type [e.g. editorial, commentary, review] |
| Lismidiati 2021 | Wrong outcome |
| Llupia 2013 | Wrong study design |
| Loiacono 2021 | Wrong intervention |
| Loiacono 2021 | Wrong intervention |
| Long 2012 | Wrong article type [e.g. editorial, commentary, review] |
| Long 2022 | Wrong population |
| Long 2022 | Wrong population |
| Long 2023 | Wrong population |
| Loo 2011 | Wrong intervention |
| Looijmans-vandenAkker 2010 | Wrong population |
| Lopes 2008 | Wrong study design |
| Luder 2018 | Wrong intervention |
| Luthi 2002 | Wrong study design |
| Ly 2015 | Wrong intervention |
| Ma 2012 | Wrong study design |
| Macknin 2000 | <100 participants |
| Maljanian 2005 | Wrong intervention |
| Maltz 2024 | Wrong outcome |
| Mantzari 2012 | Wrong article type [e.g. editorial, commentary, review] |
| Margolis 2021 | Wrong outcome |
| Marrero 2006 | Can't find full text |
| Marshall 2019 | Wrong study design |
| Marshall 2022 | Wrong population |
| MartinezVillarreal 2023 | Wrong outcome |
| Mattebo 2021 | Wrong study design |
| McAullay 2016 | Wrong intervention |
| McCarthy 2012 | Wrong study design |
| McCarthy 2015 | Wrong study design |
| McCoy 2014 | Can't find full text |
| McCulloh 2022 | Can't find full text |
| McCulloh 2022 | Can't find full text |
| McGreevy 2020 | Wrong study design |
| McIver 2016 | Wrong study design |
| McLaren 2023 | <100 participants |
| McNally 2018 | Wrong population |
| McPhee 2003 | Wrong study design |
| Meghea 2013 | Wrong intervention |
| Meharry 2012 | Wrong article type [e.g. editorial, commentary, review] |
| Messino 2020 | Wrong study design |
| Meyer 2018 | Wrong study design |
| Minor 2010 | Wrong population |
| Modin 2023 | Wrong population |
| Montejo 2017 | Wrong study design |
| Monzon 2023 | Wrong article type [e.g. editorial, commentary, review] |
| Morales 2023 | Wrong study design |
| Morris 2015 | Wrong study design |
| MuÃ±oz-Miralles 2022 | Wrong population |
| Nace 2007 | Wrong population |
| Nagykaldi 2012 | Wrong intervention |
| NavalonRamon 2019 | Wrong population |
| Nct 2007 | Wrong study design |
| Nct 2009 | Wrong outcome |
| Nct 2010 | Can't find full text |
| nct 2012 | Wrong population |
| Nct 2014 | Wrong population |
| Nct 2015 | Wrong intervention |
| Nct 2015 | Not 3+ clusters per arm |
| Nct 2015 | Wrong intervention |
| Nct 2015 | Wrong intervention |
| Nct 2016 | Wrong population |
| Nct 2018 | Wrong study design |
| Nct 2018 | Can't find full text |
| Nct 2019 | Wrong outcome |
| Nct 2020 | Wrong population |
| Nct 2021 | <100 participants |
| Nct 2022 | Wrong outcome |
| Nct 2022 | Ongoing |
| Nct 2022 | Ongoing |
| Nct 2022 | Ongoing |
| Nct 2022 | Wrong outcome |
| Nct 2022 | Ongoing |
| Nct 2022 | Ongoing |
| Nct 2022 | Ongoing |
| Nct 2022 | Ongoing |
| Nct 2022 | Ongoing |
| Nct 2022 | Ongoing |
| Nct 2022 | Wrong population |
| NCT00244374 2005 | Can't find full text |
| NCT00316303 2006 | Wrong population |
| NCT01276184 2011 | <100 participants |
| NCT02615470 2015 | Wrong intervention |
| NCT03000998 2016 | <100 participants |
| NCT04072159 2019 | <100 participants |
| NCT04323137 2020 | Wrong population |
| NCT04706403 2021 | Wrong outcome |
| NCT04939519 2021 | Ongoing |
| NCT04981392 2021 | Wrong intervention |
| NCT05009251 2021 | Wrong population |
| NCT05052190 2021 | Can't find full text |
| NCT05293392 2022 | Not 3+ clusters per arm |
| NCT05490329 2022 | Ongoing |
| NCT05535777 2022 | Can't find full text |
| NCT05607082 2022 | Ongoing |
| NCT05612360 2022 | Can't find full text |
| NCT05621226 2022 | Can't find full text |
| NCT05713526 2023 | Ongoing |
| NCT05764174 2023 | Can't find full text |
| NCT05875779 2023 | Ongoing |
| NCT05993260 2023 | Ongoing |
| NCT06146361 2023 | Ongoing |
| Norr 2003 | Wrong intervention |
| Nowalk 2005 | Wrong study design |
| Nowalk 2014 | Wrong intervention |
| Nyamathi 2015 | Wrong population |
| Nyamathi 2016 | Wrong population |
| Nyhan 2014 | Wrong outcome |
| Ogburn 2007 | Wrong study design |
| O'Leary 2016 | Wrong intervention |
| O'Leary 2017 | Wrong intervention |
| O'Leary 2019 | Wrong intervention |
| Opel 2020 | Wrong intervention |
| Ortiz 2017 | Wrong outcome |
| Owens 2016 | Can't find full text |
| Ozdemir 2023 | Wrong population |
| Pack 2023 | <100 participants |
| Palmer 2015 | Wrong population |
| Palmore 2009 | Wrong study design |
| Panozzo 2020 | Wrong outcome |
| Pappano 2004 | Wrong population |
| Parry 2004 | Wrong study design |
| Parsons 2022 | Wrong study design |
| Paskett 2016 | Wrong intervention |
| Patel 2022 | Wrong population |
| Patel 2022 | Ongoing |
| Pati 2015 | Wrong study design |
| Perkins 2016 | Wrong study design |
| Perkins 2020 | Wrong intervention |
| Phibbs 2006 | Wrong intervention |
| Pich 2019 | Wrong article type [e.g. editorial, commentary, review] |
| Polgreen 2008 | Wrong population |
| Polinski 2018 | Wrong study design |
| Poparad-Stezar 2021 | Wrong study design |
| Porter 2018 | Wrong outcome |
| Prati 2012 | Wrong outcome |
| Preston 2019 | Wrong study design |
| Qin 2021 | Wrong study design |
| QuerinoSouza 2014 | Wrong study design |
| Quinley 2004 | Wrong intervention |
| Rabin 2023 | Ongoing |
| Rajakumar 2010 | Wrong study design |
| Ramakrishna 2021 | Wrong study design |
| Ramon 2019 | Wrong population |
| Rand 2023 | Wrong intervention |
| Rane 2021 | Wrong study design |
| Reddinger 2022 | Wrong outcome |
| Redfield 2000 | Wrong study design |
| Reich 2019 | Wrong population |
| Reno 2019 | Wrong intervention |
| Reno 2023 | Wrong outcome |
| Reno 2023 | Wrong outcome |
| Riphagen-Dalhuisen 2012 | Wrong population |
| Riphagen-Dalhuisen 2013 | Wrong population |
| Riphagen-Dalhuisen 2013 | Wrong population |
| Ritchie 2018 | Wrong study design |
| Robare 2011 | Wrong intervention |
| Rodriguez 2022 | Wrong study design |
| Rodriguez 2023 | Wrong study design |
| Rodriguez 2023 | Wrong article type [e.g. editorial, commentary, review] |
| Rosenberg 2019 | Wrong intervention |
| Rosentrater 2018 | Wrong study design |
| Roston 2012 | Wrong article type [e.g. editorial, commentary, review] |
| Rothan-Tondeur 2010 | Wrong population |
| Rouhana 2011 | Wrong outcome |
| Ruffin 2015 | Wrong study design |
| Rushton 2015 | Wrong study design |
| Russell 2012 | Can't find full text |
| Sadler 2013 | Wrong intervention |
| Sadlier 2019 | Wrong article type [e.g. editorial, commentary, review] |
| Sahni 2017 | Wrong study design |
| Saitoh 2013 | <100 participants |
| Samson 2020 | Wrong population |
| Sand 2007 | Wrong population |
| Sanderson 2017 | Not 3+ clusters per arm |
| Sansom 2003 | Wrong study design |
| Santos 2021 | Wrong population |
| Satybaldiyeva 2024 | Wrong outcome |
| Saul 2021 | Wrong outcome |
| Saunier 2020 | Wrong population |
| Sawyer 2012 | Wrong study design |
| Schmidtke 2020 | Wrong population |
| Schuh 2023 | Wrong outcome |
| Schulte 2019 | Wrong population |
| Schultze 2014 | Wrong intervention |
| Schwarz 2006 | Wrong article type [e.g. editorial, commentary, review] |
| Sege 2015 | Wrong intervention |
| Seror 2022 | Wrong population |
| Seror 2022 | Wrong population |
| Shah 2008 | Wrong study design |
| Sharma 2015 | Can't find full text |
| Sharma 2021 | Wrong population |
| Sheer 2021 | Wrong intervention |
| Shenson 2001 | Wrong study design |
| Shermohammed 2021 | Wrong population |
| Shin 2022 | Wrong study design |
| Shoup 2015 | Wrong population |
| Silverstein 2004 | Wrong outcome |
| Skaathun 2024 | Ongoing |
| Slaunwhite 2009 | Wrong population |
| Soubrier 2013 | Wrong population |
| Staras 2015 | Wrong study design |
| Staras 2020 | Wrong study design |
| Staras 2021 | Wrong outcome |
| Stephens 2021 | Wrong intervention |
| Stille 2001 | Wrong study design |
| Stubbs 2014 | Wrong study design |
| Sumar 2023 | Ongoing |
| Sun 2022 | Wrong outcome |
| Suppli 2017 | Wrong study design |
| Szilagyi 2019 | Wrong study design |
| Taddio 2021 | Wrong outcome |
| Takagi 2023 | Wrong outcome |
| Takahashi 2023 | Wrong outcome |
| Takamatsu 2022 | Wrong study design |
| Tao 2019 | Wrong population |
| Thirumurthy 2022 | Wrong study design |
| Thomas 2010 | Wrong population |
| Thomas 2019 | Wrong article type [e.g. editorial, commentary, review] |
| Thomas 2022 | Wrong study design |
| Thompson 2018 | Wrong study design |
| Tran 2022 | Not 3+ clusters per arm |
| Tran 2022 | Not 3+ clusters per arm |
| Tran 2022 | Not 3+ clusters per arm |
| Tse 2023 | Can't find full text |
| Tuckerman 2022 | Wrong population |
| Tuckerman 2023 | Wrong intervention |
| Tuckerman 2023 | Wrong population |
| Tuv 2023 | Wrong population |
| Tuv 2023 | Wrong population |
| Ugarte 2022 | Wrong population |
| Urkin 2016 | Can't find full text |
| Valdez 2015 | Wrong outcome |
| Valerio 2021 | Wrong study design |
| Valiente 2010 | Can't find full text |
| Vanderpool 2012 | Wrong article type [e.g. editorial, commentary, review] |
| VanSteenbergen 2002 | Wrong study design |
| Venegas-Murillo 2022 | Wrong study design |
| Vilella 2004 | Wrong study design |
| Vimercati 2019 | Wrong study design |
| Vo 2018 | Wrong study design |
| Walkey 2021 | Wrong study design |
| Walsh 2010 | Wrong outcome |
| Walter 2008 | Wrong study design |
| Wang 2018 | Can't find full text |
| Wang 2022 | Wrong study design |
| Wang 2023 | Wrong study design |
| Wang 2023 | Wrong study design |
| Wang 2024 | Ongoing |
| Washington 2023 | Wrong study design |
| Webb 2022 | Wrong study design |
| Wedel 2016 | Wrong study design |
| Wegwarth 2014 | Wrong study design |
| Wells 2022 | Wrong study design |
| White 2022 | Not 3+ clusters per arm |
| Witus 2022 | Wrong outcome |
| Wong 2022 | Wrong study design |
| Woolard 2006 | Can't find full text |
| Wray 2009 | Wrong outcome |
| Wu 2021 | Wrong study design |
| Wu 2022 | Wrong study design |
| Yao 2022 | Wrong study design |
| You 2023 | Wrong intervention |
| Zeis 2016 | Can't find full text |
| Zhu 2022 | Wrong outcome |
| Zhu 2023 | Wrong article type [e.g. editorial, commentary, review] |
| Zhu 2023 | Wrong article type [e.g. editorial, commentary, review] |
| Zimet 2018 | Wrong intervention |
| Zimet 2020 | Wrong study design |
| Zimmerman 2003 | Wrong study design |
| Zimmerman 2003 | Wrong study design |
| Zimmerman 2004 | Wrong study design |
| Zimmerman 2006 | Wrong study design |
| Zimmerman 2006 | Wrong study design |
| Zimmerman 2014 | Wrong intervention |
| Zimmerman 2017 | Wrong intervention |
| Zimmerman 2017 | Wrong intervention |
| Zwar 2012 | Wrong intervention |
